# Post-translational insertion of boron in proteins to probe and modulate function

**DOI:** 10.1038/s41589-021-00883-7

**Published:** 2021-11-01

**Authors:** Tim A. Mollner, Patrick G. Isenegger, Brian Josephson, Charles Buchanan, Lukas Lercher, Daniel Oehlrich, D. Flemming Hansen, Shabaz Mohammed, Andrew J. Baldwin, Véronique Gouverneur, Benjamin G. Davis

**Affiliations:** 1 Department of Chemistry, Chemistry Research Laboratory, University of Oxford, Oxford, UK; 2 Department of Chemistry, Physical and Theoretical Chemistry Laboratory, University of Oxford, Oxford, UK; 3 Neuroscience Medicinal Chemistry, Janssen Research and Development, Beerse, Belgium; 4 Division of Biosciences, University College London, London, UK; 5 Department of Biochemistry, University of Oxford, Oxford, UK; 6 The Rosalind Franklin Institute, Oxfordshire, Oxford, UK

**Keywords:** Chemical tools, Proteins, NMR spectroscopy, Chemical modification

## Abstract

Boron is absent in proteins, yet is a micronutrient. It possesses unique bonding that could expand biological function including modes of Lewis acidity not available to typical elements of life. Here we show that post-translational Cβ–Bγ bond formation provides mild, direct, site-selective access to the minimally sized residue boronoalanine (Bal) in proteins. Precise anchoring of boron within complex biomolecular systems allows dative bond-mediated, site-dependent protein Lewis acid–base-pairing (LABP) by Bal. Dynamic protein-LABP creates tunable inter- and intramolecular ligand–host interactions, while reactive protein-LABP reveals reactively accessible sites through migratory boron-to-oxygen Cβ–Oγ covalent bond formation. These modes of dative bonding can also generate de novo function, such as control of thermo- and proteolytic stability in a target protein, or observation of transient structural features via chemical exchange. These results indicate that controlled insertion of boron facilitates stability modulation, structure determination, de novo binding activities and redox-responsive ‘mutation’.

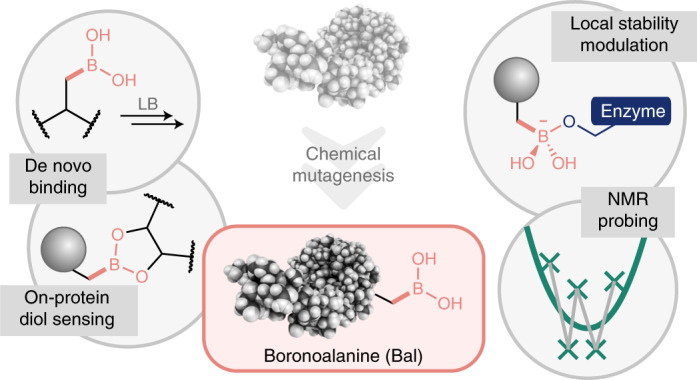

## Abstract (web summary)

Post-translational site-selective formation of boronoalanine in proteins enables applications of boron for binding partner capture, footprinting of interactions with reactive oxygen species, proteolytic control and mapping of transient structures.

## Main

**Fig. 1:**
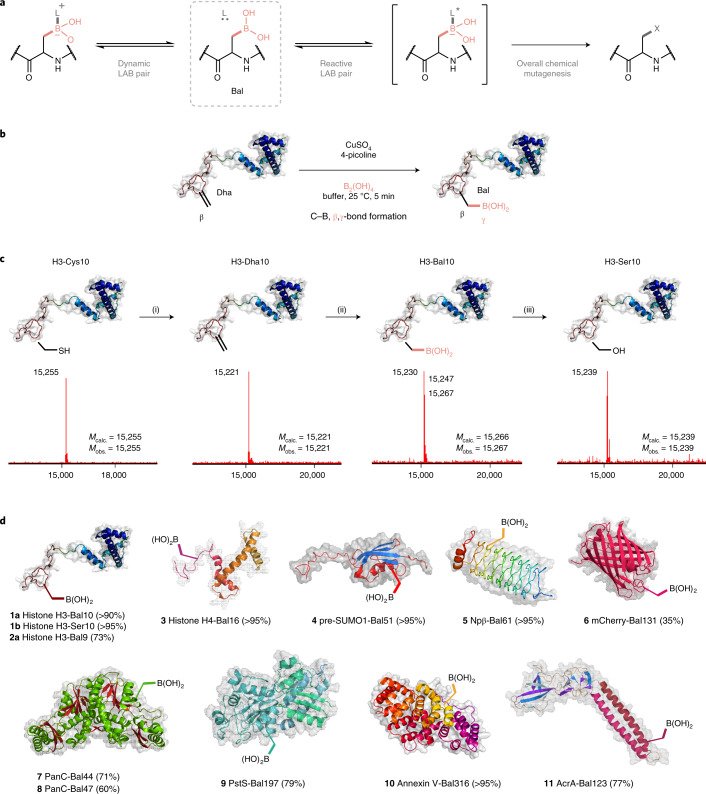
A strategy for borylation and precise exploitation in biomolecule engineering. In this work, we show that such boron–carbon bond formation can create the minimal boronyl amino acid residue boronoalanine (Bal) in a variety of complex protein environments, allowing precise positioning at preselected sites. **a**, This, in turn, enables the engineering of diverse de novo binding functions into proteins that exploit complementary modes of both dynamic and reactive dative, Lewis acid–base (LAB) pair probing. **b**, This uses catalytic on-protein aqueous, Bγ–Cβ bond formation ‘+B(OH)_2_’ borylation chemistry that is rapid, general and benign. **c**, Bγ–Cβ bond-forming borylation to Bal allows for post-translational mutagenesis and can be applied as part of a global sequence from Cys to Ser residues via Dha and Bal. Scheme and LC–electrospray-MS shown for histone H3 protein (Fig. 4, Supplementary Fig. 1 and Extended Data Fig. 1). Reagents and conditions were (i) 2,5-dibromohexanediamide in NaP_i_ buffer (pH 8.0), room temperature to 37 °C, 4 h; (ii) tetrahydroxydiboron (B_2_(OH)_4_), CuSO_4_, 4-methylpyridine/picoline in NaP_i_ buffer (pH 7.0), room temperature, 5 min; and (iii) H_2_O_2_ in NaP_i_ buffer (pH 8.0), room temperature, 10 min. **d**, Protein substrate scope of the Bγ–Cβ bond-forming copper-promoted hydroborylation reaction, encompassing a range of protein types, sites and fold motifs.

**Extended Data Fig. 1:**
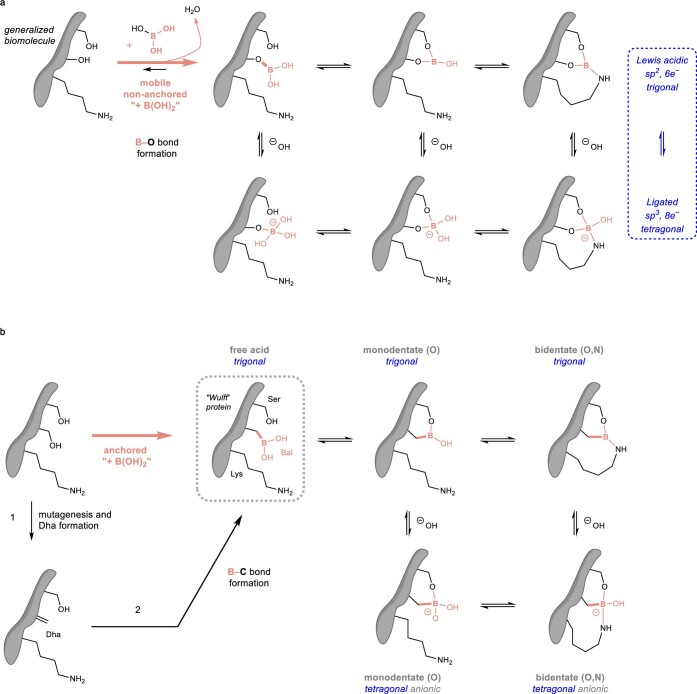
Borylation in Nature and a Strategy for its Precise Exploitation in Biomolecule Engineering. Borylation in Nature and a Strategy for its Precise Exploitation in Biomolecule Engineering: (**a**) In nature, boron is sequestered as an essential or ‘likely-essential’ micronutrient through mobile, non-anchored ‘+B(OH)_2_’ borylation via B–O bond-formation, enabling it seems critical structural motifs. (**b**) Yet, nature has not, to-date, exploited ‘+B(OH)_2_’ B–C bond-formation, which would allow precise, site^−^selective anchoring of such valuable function into biomolecules.

Boron’s electronic proximity to carbon has long drawn comparisons and highlighted their different roles in nature^1^. While both show more extensive speciation^2^ compared to most other elements, boron’s (semi)dynamic interactions find it differentially sequestered across the domains of life. Insertion of B(OH)_n_ structures into nature—that is borylation (Fig. 1 and Extended Data Fig. 1)—appears to generate endogenous functional effects not available through the traditional modes of biological bonding based on the organic chemistry of carbon, hydrogen, nitrogen and oxygen alone. Yet, despite this, it plays only a minor role in current ‘native’ biology.

In many cases, boron’s unique effects arise from the selective, dative engagement of ligand(s) (typically oxygen or nitrogen Lewis bases). It sits at a dynamic cusp between two semistable binding states: Lewis-acidic *sp*^2^ 6e^−^ (electron-deficient) and ligated or datively bonded *sp*^3^ 8e^−^ (electron-rich) (Fig. 1a and Extended Data Fig. 1a), thereby ‘sampling’ ligands via Lewis acid–base pairing (LABP) (Fig. 1a and Extended Data Fig. 1a). Nearby moieties may modulate this Lewis acidity (so-called ‘Wulff-type’^3^ boronates). Notably, ligand binding can be accomplished even in competing Lewis-basic solvents such as water.

Despite its implicated use, the nonanchored (Extended Data Fig. 1a) character of natural borylation prevents explanation of its precise functional roles. In plants, for example, the essential nutritional role of boron drives postbiosynthetic borylation; in muro *cis*-diol engagement of apiosyl sugar residues by borylation (+‘B(OH)_2_’) critically regulates cell-wall strength^4^. However, this nonanchored borylation cannot be predetermined (Extended Data Fig. 1a), resulting in an inability to control or exploit its effects.

**Extended Data Fig. 2:**
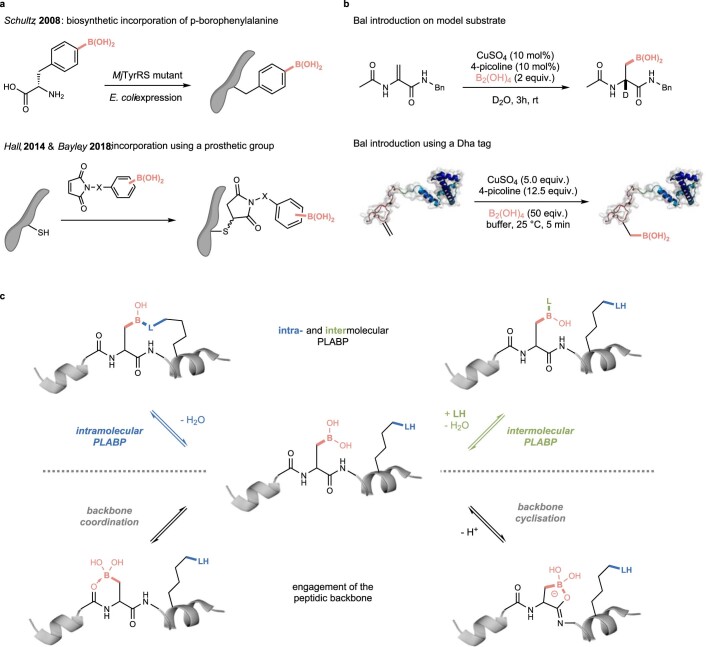
Borylation of proteins allows for Protein Lewis acid•base pair probing (PLABP). Borylation of proteins allows for Lewis acid•base pair probing (PLABP). (**a**) Previously published examples of introduction of boronic acid moieties into proteins are based on either biosynthetic incorporation of prefunctionalized unnatural amino acids^5^ or bioconjugation strategies linking the desired motif via a prosthetic group^6,8^. (**b**) Cu-catalyzed deuteroborylation of a peptidic model substrate under benign aqueous conditions (**top**) led to the development of a method for the chemical posttranslational introduction of Bal residues allowing for direct access to borylated proteins (**bottom**). (**c**) Bal can engage reversibly with Lewis-basic moieties either on the peptidic backbone or via PLABP. PLABP can occur with intramolecular Lewis-bases as well as with intramolecular Lewis-bases (such as Lewis-basic side-chain residues). Backbone interaction leads to backbone coordination or backbone cyclisation.

Methods for introduction of boron-containing moieties into larger biomolecules remain limited. This is made challenging by the many Lewis bases in biomolecules that could sequester them (and hence inhibit installation). Two general methodologies for site-selective introduction of boronic acids into proteins have been reported (Extended Data Fig. 2a): biosynthetic incorporation via tyrosine mimicry^5^, or attachment of a prosthetic group^6^. Biosynthetic incorporation can be limited by reduced expression yields; boronic acids act as inhibitors of protein translation^7^. The alternative use of prosthetic groups necessitates larger, linker-based constructs^6,8^.

We reasoned that boron–carbon bond formation might allow the insertion of a minimal boryl moiety (‘B(OH)_2_’, Extended Data Fig. 1b) with precise control of its site (‘anchored’) and unique functions via direct programmable ‘editing’ into biomolecules. The alkylborono-aminoacid boronoalanine (Bal, Fig. 1a) represents a minimal borylated residue. It is a challenging amino acid to isolate^9^; the boronyl sidechain readily engages its own alpha amino or carboxy moieties^9,10^. Such expected Lewis-acidic coordination hampers typical peptide assembly.

Direct late-stage Cβ–Bγ bond formation, ideally from an unprotected borylation source, might allow ready access to Bal in proteins via a tag-and-modify approach^11^ from the flexible intermediate residue dehydroalanine (Dha) (Fig. 1b). Dehydroalanine residues (Dha) can now be readily introduced into proteins via a variety of chemical and biological methods and therefore provide a versatile ‘tag’ for site-selective protein modification^12^. Given the precedent for useful chemo- and regioselective hydroborylation reactions of terminal alkenes, including in Dha dipeptide models^13^, as well as procedures using aqueous solvent under Cu(II) catalysis with appropriate *N*-ligands on nonpeptidic systems^14,15^, we considered that the terminal olefin of Dha would allow distinct reactivity over other proteinogenic residues, thereby allowing selective carbon–boron bond formation in proteins under potentially benign conditions. Notably, during the final stage of this work elegant, proof-of-principle borylation of longer peptides, ribosomally synthesized and post-translationally modified peptides, was also independently disclosed^16^.

Here we show that mild boron–carbon bond-mediated insertion creates Bal (Fig. 1) and allows exploitation of multiple modes of boron-to-ligand engagement in proteins. In this way dative Lewis acid–base pair interactions (Fig. 1a) may be programmed into and used in biological systems.

## Results

### Chemical introduction of boronoalanine (Bal) into proteins

First, the reactivity of Dha in a small peptidic substrate (compound 1) was tested (Extended Data Fig. 2b). Cu-mediated borylation^14^ conditions using direct, unprotected ‘B(OH)_2_’ source B_2_(OH)_4_ explored the roles of ligand, copper source and base under solely aqueous conditions and revealed that formation of boronoalanine from Dha was feasible with excellent conversions and regioselectivity (Extended Data Fig. 2b and Supplementary Table 1).

Multiple copper sources and additives or ligands were screened on model substrates. Most copper(II) sources (CuSO_4_, Cu(NO_3_)_2_, Cu(OAc)_2_ and Cu(OTf)_2_) gave excellent yields (using 4-picoline as additive). Basic copper carbonate gave lower yield, possibly due to limited solubility. Whereas bidentate ligands (2,2′-bipyridine and 1,8-*bis*(dimethylamino)naphthalene) gave only low yields (6–11%), excellent conversions (96-99%) were generally seen for monodentate, pyridine additives (for example, pyridine, 4-picoline). Monodentate imidazoles (imidazole, l-histidine) also gave good yields (66–88%). While common denaturant guanidine hydrochloride partly facilitated reaction (18%), addition of urea did not. Overall, 4-picoline and copper sulfate proved optimal, affording 2 in 99% yield and with >98% regioselective Cβ–Bγ formation at the Dha residue to give Bal.

Next, having demonstrated reactivity under biocompatible conditions, we turned to full-length biomolecule substrates (Fig. 1c,d). Initially, variation (Supplementary Tables 2 and 3) using model protein substrate histone H3-Dha10 revealed reactivity at a range of pH (pH 6.5–8.5 optimal, Supplementary Table 3, 88–92%). Common denaturants (guanidine hydrochloride, urea) proved beneficial but not essential, consistent with addition of a small, borylation motif. Together this allowed determination of optimal conditions for successful on-protein Cβ–Bγ borylation using tetrahydroxydiboron (50 equiv.), CuSO_4_ (5 equiv.) and 4-picoline (12.5 equiv.) in NaP_i_ buffer (100 mM, 3 M Gdn∙HCl, pH 7.0) with excellent conversions (>90%, Fig. 1c and Supplementary Tables 2 and 3) to yield H3-Bal10.

**Extended Data Fig. 3:**
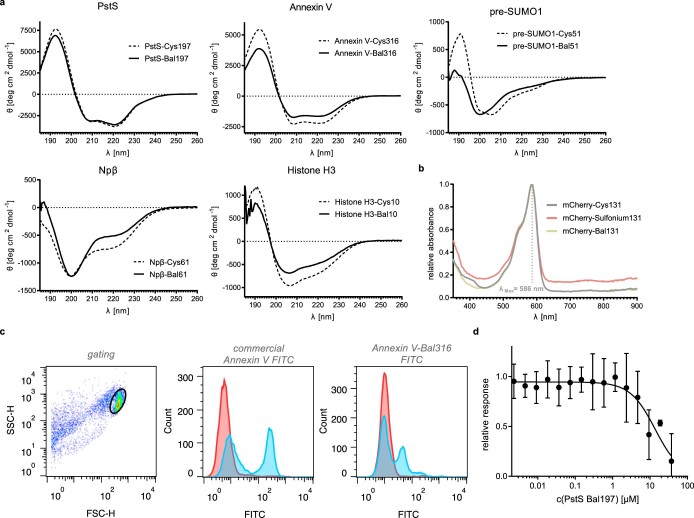
Retention of fold and function after borylation. Retention of fold and function after borylation: (**a**) CD spectra of borylated proteins PstS-Bal197, Annexin V-Bal316, Npβ-Bal61, preSUMO1-Bal51 and Histone H3-Bal10 in comparison with their respective cysteine mutants. (**b**) Absorbance spectra of mCherry-C131, mCherry-Sulfonium131 and mCherry-Bal131 show unaltered absorbance maxima at 586 nm. (**c**) Annexin V-Bal316 binding to apoptotic cells (apoptosis induced using etoposide (ETO) at 25 μM concentration). (**d**) Inhibitor vs. response plot for competition of PstS-Bal197 with phosphate sensor. A stock solution of the commercially available, coumarin-dye labelled PstS variant^27^ ‘Phosphate Sensor’ (ThermoFisher Scientific, USA) and sodium phosphate in TRIS buffer (20 mM, pH 7.6) was prepared and titrated against PstS-Bal197, while the response was measured via fluorescence readout. Values represent mean ± SD. n = 3.

**Extended Data Fig. 4:**
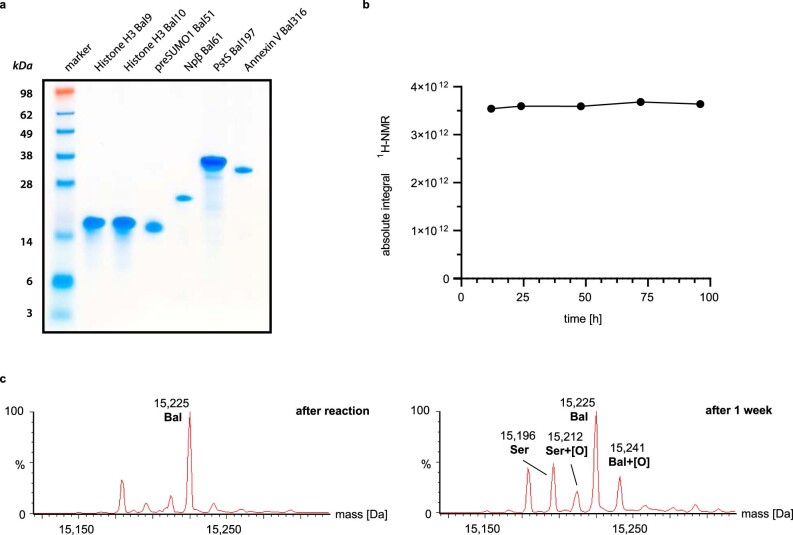
*In vitro* stability determination for boronoalanine (Bal). *In vitro* stability determination for boronoalanine (Bal). (**a**) SDS-PAGE (Coomassie staining) analysis of borylated proteins (marker: Invitrogen SeeBlue™ Plus2 Pre-stained Protein Standard) (**b**) Stability testing of boronoalanine under ambient conditions: No degradation of model Ac-Bal-NHBn could be observed in aqueous buffer (NaP_i_ pH 7.0) via ^1^H-NMR analysis. Absolute integrals were compared for the characteristic C_β_ protons. (**c**) Oxidation could be observed on representative protein Histone H3-Bal9 after prolonged storage under ambient conditions (non-degassed buffer, room temperature) for one week. ~33% of Bal was converted to the corresponding Ser residue at a level seemingly comparable with non-selective oxidation (presumed for example Met-O). Conducting a single experiment was deemed sufficient for qualitative analysis.

**Extended Data Fig. 5:**
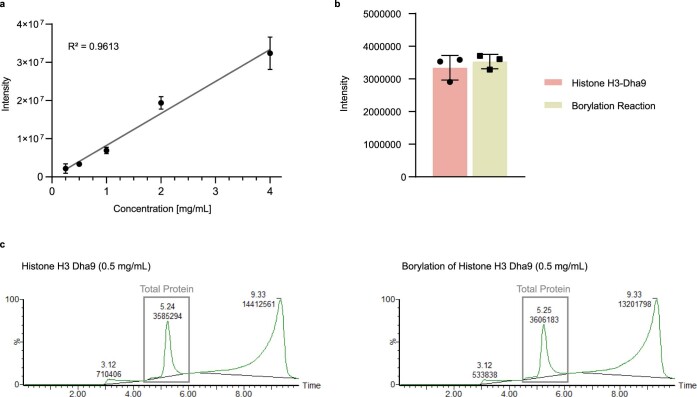
Quantification of borylation yields via LC–MS. Quantification of borylation yields via LC-MS. (**a**) The calibration curve for model protein Histone H3-Dha9 shows linearity over a long range of relevant concentrations (n = 3, error bars depict SD) (**b**) A comparison of integrals derived from the total ion chromatogram (TIC) of Histone H3-Dha9 and Histone H3 Bal9 reveals comparable ionization (0.5 mg/mL, n = 3, error bars depict SD). (**c**) Representative total ion chromatograms for Histone H3-Dha9 and Histone H3 Bal9. The total protein peak used for integration is highlighted (grey box).

Next, a range of proteins differing in size, fold, stability and biological function were tested (Fig. 1d): histones H3 and H4, small α-helical nuclear proteins; pre-SUMO1 (SUMO, small ubiquitin-like modifier), small globular protein containing α-helices and β-sheets; Npβ, β-helical pentapeptide repeat^17^; Annexin V, globular α-helical protein capable of Ca^2+^-dependent phospholipid binding; PanC, enzyme; mCherry, β-barrel oxidation-sensitive fluorescent protein; PstS, protein involved in bacterial phosphate transport^18^ and AcrA, membrane protein. Characterization (including intact protein mass spectrometry (MS), proteolytic/tandem MS (MS/MS) ‘peptide mapping’, circular dichroism and electrophoresis: Supplementary Figs. 1 and 2 and Extended Data Figs. 3–5) confirmed that proteins were successfully and site-selectively converted to their respective Bal variants (Fig. 1d). Moreover, proteins retained a folded state (Extended Data Fig. 3a) and/or function (Extended Data Fig. 3b–d); notably, in some cases this was associated with partial modulation of fold (Supplementary Tables 4 and 5) and/or activity. PstS retained phosphate-binding ability in PstS-Bal197 (Extended Data Fig. 3d), Annexin-Bal316 retained ability to bind apoptotic cells (Extended Data Fig. 3c) and mCherry-Bal131 retained its spectrophotometric properties (Extended Data Fig. 3b). Residual copper levels were <4 ppm (Methods).

### Bal allows intramolecular dynamic Lewis acid–base pairing

Alkyl boronates show Lewis acidity toward hard Lewis bases in pairing (LABP). The extent is not only kinetically dependent^6^ but can be externally modulated (‘Wulff-type’^3^, see also ref. ^19^). Simple, unmodulated alkylboronic acids usually exhibit p*K*_a_ higher than arylboronic acids (for example, p*K*_a_ methylboronic acid 10.7 and p*K*_a_ phenylboronic acid 8.8) rendering them typically^20^ less suitable for binding applications in aqueous media^21^. Titration revealed that Bal displays higher acidity (p*K*_a_ = 8.31, Supplementary Fig. 3) while retaining minimal size.

**Fig. 2:**
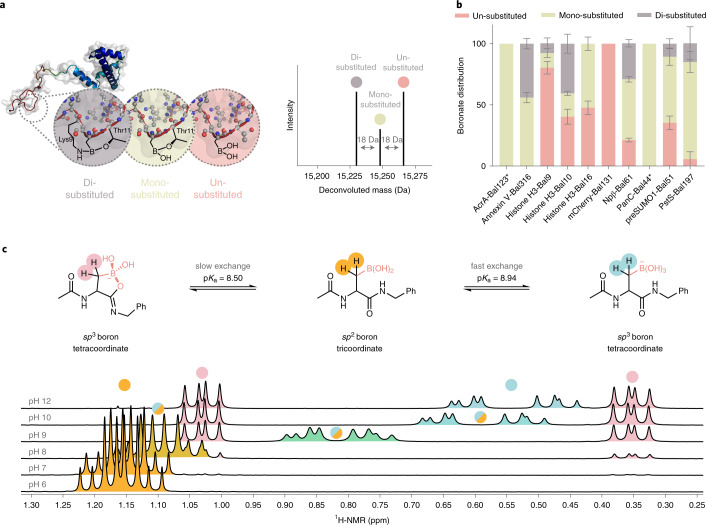
Dynamic dative PLABP. **a**, Total protein MS reveals that, once Lewis acid Bal is inserted into different environments, it engages local Lewis bases to differing extents according to substitution state: un-, mono- or di-substituted, as judged by *m/z* difference of 0, −18 or −36 Da, respectively (right). In each case, possible Lewis-basic sidechains can be identified, for example, at Bal10 in H3-Bal10, Thr13 or Lys9 (left), but it should be emphasized that these data do not allow direct identification of such ligands. **b**, The site-dependent coordination states detected by protein LC–MS. *n* = 3, mean ± s.d. given; *n = 1. **c**, NMR investigations on small-molecule model Ac-Bal-NHBn show pH-dependent boronate formation (turquoise) as well as oxaborolane formation (rose) with the C-terminal amide C = O as one specific mode of PLABP. Fast exchange can be observed for boronate formation, while slow exchange is observed for oxaborolane formation (NaP_i_-buffered D_2_O).

We probed the effect of installation of Bal at different protein sites, folds and environments (exposed versus enclosed) (Fig. 1c and Supplementary Fig. 4). Intact protein mass spectra showed distinct peaks for three different borylated species (Fig. 2a and Supplementary Table 6). These corresponded to free Bal and also different ligated forms: mono- and di-substituted boronyl (Fig. 2a,b). These signatures of Bal’s coordination-state (free, mono-, di-: Fig. 2a,b) therefore probed protein environment through protein-LABP (PLABP).

Analysis of putative ligands suggested (Fig. 2b and Supplementary Table 6) protein-specific boronyl substitution that could be linked with the respective Lewis base environment. Substitution ranged from negligible (for example, in mCherry-Bal131, consistent with the solvent-exposed location of outer barrel site 131), to high amounts of mono-substituted (for example, in AcrA-Bal123, PanC-Bal44, in more structured enclosed regions, presenting internal ligands), to mainly di-substituted boronates (for example, in Histone H3-Bal10, consistent with a flexible N-terminal tail that can provide multiple internal ligands).

**Extended Data Fig. 6:**
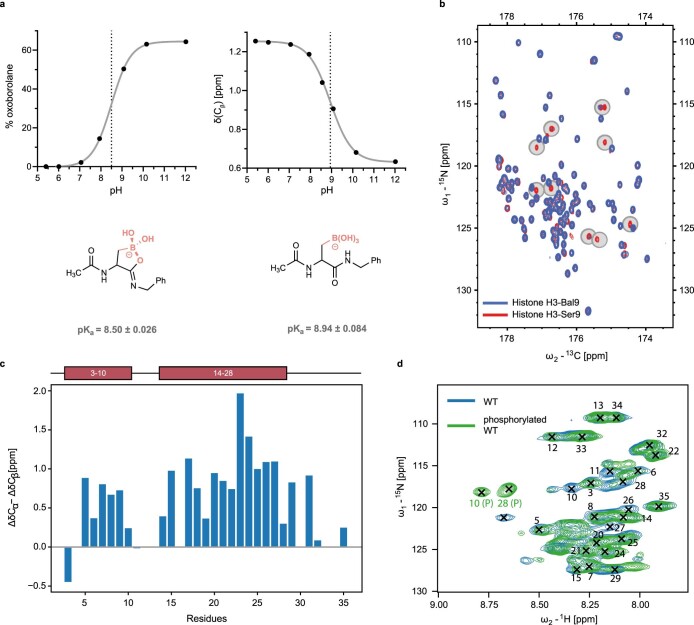
Boronoalanine enables DICE–NMR. Boronoalanine enables DICE NMR. (**a**) ^1^H-NMR allows for pK_a_ determination for oxaborolane (**left**) and boronate (**right**) formation. (**b**) ^13^CO^15^N protein NMR with isotopically (^13^C, ^15^N) labelled Histone H3 allowed more detailed delineation of PLABP. Comparison of Histone H3-Ser9 (**red**) and Histone H3-Bal9 (**blue**) indicates that localized interaction is induced by placing Bal at site 10 leading to the loss of six signals (**grey circles**) as induced by intermediate exchange. The greater shift from slow (in small molecule model Ac-Bal-NHBn) to intermediate exchange (in protein) can be rationalized by the increased local concentration of backbone carbonyls available for PLABP in the case of borylated protein Histone H3-Bal9. (see also Fig. 2b, Supplementary Fig. 4,5 and Supplementary Table 7 for further details including details of amide-specific isotopic labelling revealed pH-dependent intra-residue oxaborolane formation with the C-terminal amide C = O as one specific mode of PLABPs). Protein NMR spectra were recorded in aqueous buffer (50 mM NaPi, 3 M Gdn•HCl, 5% D_2_O, pH 7.0). (**c**) Residual secondary structure determination in the tail region of non-phosphorylated Histone H3 analysing the ΔδCα - ΔδCβ chemical shifts in a nucleosome context indicates the presence of two regions with residual α-helical structure (residues 3-10 and 14-28). (**d**) Overlay of ^1^H-^15^N-HSQC spectra of Histone H3 WT and phosphorylated Histone H3 WT in a nucleosome context.

This observed Bal Lewis acid ligation state was probed further using peptide and protein nuclear magnetic resonance (NMR) (Fig. 2c and Extended Data Fig. 6). In the most diversely coordinated protein system, histone H3-Bal10 (roughly 35% un-, 20% mono- and 45% di-substituted, Fig. 2b), fully ^13^C-^15^N-isotope-labeled protein variants H3-Ser9 and H3-Bal9 were generated and compared via the CON 2D projection of 3D HNCO spectra (Extended Data Fig. 6b). This revealed alteration of specific ^13^C(O)−^15^N crosspeaks on introduction of Bal, consistent with PLABP. Moreover, peptidic ^15^N-labeled isotopologs probed with ^1^H-, ^11^B-, ^13^C-, ^15^N-, ^1^H-^15^N-HMBC and ^1^H-^13^C-HMBC NMR spectroscopy revealed unambiguous pH-dependent Bγ–O engagement of the intraresidue C-terminal backbone amide C=O to form oxaborolane (Fig. 2c, Extended Data Fig. 6a, Supplementary Figs. 4 and 5 and Supplementary Table 7), consistent with the observed protein NMR (Extended Data Fig. 6b), determined p*K*_a_ (Extended Data Fig. 6a and Supplementary Fig. 3) and apparent modulation of function by intramolecular Lewis bases (vide infra). Bal was thus confirmed as a flexible intramolecular binding residue.

### Bal generates de novo intermolecular binding function

This engagement by Bal with internal Lewis bases also suggested potential in binding Lewis-basic moieties in intermolecular partners. Moreover, our observations suggested potential for Wulff-type^3^ and/or competing modulation of substitution. In this way, possible competition between intra- versus intermolecular engagement might provide a mode for higher selectivity than small-molecule boronic acid ‘sensors’ and reduced oligomerization^22^ inside proteins.

**Extended Data Fig. 7:**
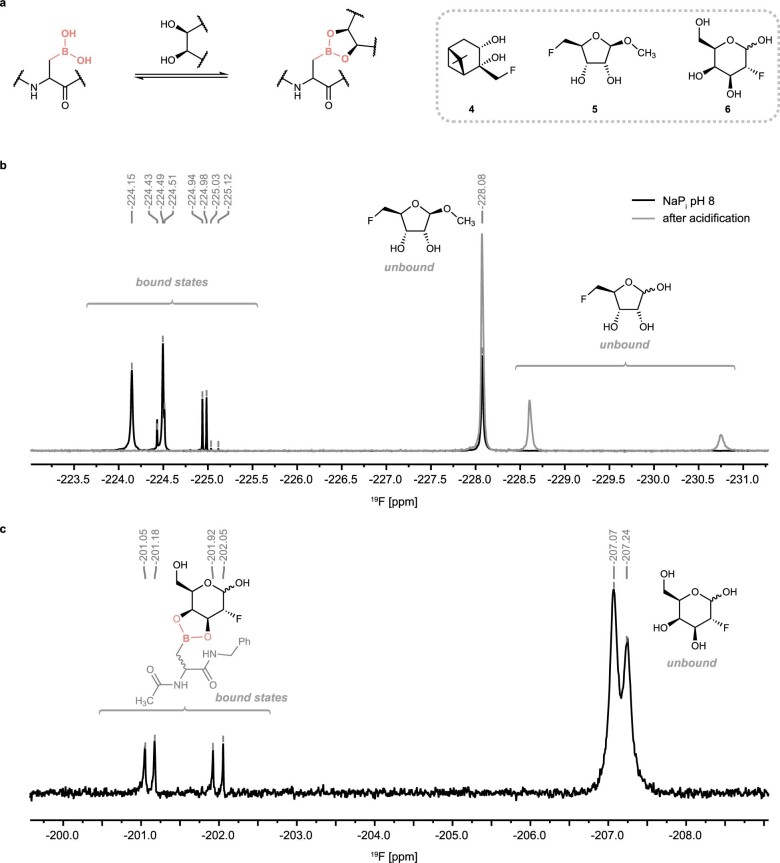
Diol sensing using small molecule model Ac-Bal-NHBn. Diol sensing using small molecule model Ac-Bal-NHBn. (**a**) Fluorinated diols for ^19^F-NMR probing of diol binding on borylated protein (**b**) ^19^F-NMR spectrum of the binding of ribose derivative 5 to Ac-Bal-NHBn under weakly basic conditions (black) reveals a complex mixture comprising of eight distinguishable bound states. Upon acidification with conc. HCl, bound states are no longer observable (grey). Unbound diol can be observed at −228.08 ppm. (**b**) Binding of fluorinated galactose derivative 6 to model compound 2.

We had already established compatibility with existing, endogenous intermolecular binding functions; borylation of PstS and Annexin V did not remove inherent phosphate binding (phosphate and phosphatidylserine, respectively). As an initial test of de novo (‘host–guest’) binding function, H3-Bal10 was surveyed with a range of possible guest ligands (Extended Data Fig. 7 and Supplementary Fig. 7). Wild-type (WT) Histone H3 has no integral function as a host receptor. We tested for intermolecular engagement with biologically derived poly-ols (terpenes, glycans).

**Fig. 3:**
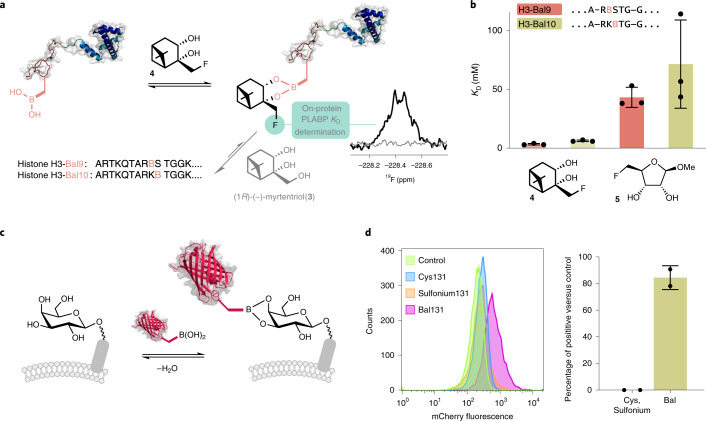
Bal-containing proteins selectively capture biological partners through dative PLABP. **a**, Through the introduction of Bal, de novo function for selective intermolecular binding of terpene and sugar poly-ols (as Lewis bases) is introduced into a previously nonbinding, nonreceptor protein (histone H3). Consistent with differential ‘Wulff-type’ enhancement and modulation by intramolecular Lewis bases already present in the sidechains of residues in the protein (here within the Lewis base-rich pentapeptide motif R_8_KSTG_12_), this binding is sensitive to both ligand type and protein site. **b**, Even movement within that RKSTG register by one residue (site 9 → site 10) causes substantial modulation of *K*_D_. Values represent the mean ± s.d. from *n* = 3 individual experiments. **c**, Exploitation of this de novo function for selective intermolecular poly-ol binding allows the creation of a fluorescent protein reagent mCherry-Bal131. **d**, Through the introduction of Bal into the outer barrel of mCherry, this Bal-fluorescent protein can detect cell-surface glycosylation on mammalian (CHO-WT) cells (84.4 ± 6.3%, left), as shown by flow cytomtery analysis; its nonborylated counterparts show no inherent binding (<5%, right). Percentage of positive versus control (right) was calculated by averaging values obtained when using both the Overton cumulative histogram subtraction algorithm and the Super-Enhanced *D*_max_ Subtraction (SED) algorithm as implemented in FlowJo.

Using suitably labeled ligands we observed (by ^19^F-NMR) ‘capture’ by intermolecular PLABP (Fig. 3a,b) in H3-Bal10. This not only displayed single-site, saturation behavior as a host but also reversible and selective competition by alternative ligands (Fig. 3a).

We not only probed (Fig. 3b) ‘guest’-ligand type (terpene-poly-ol versus sugar-poly-ol) but also site-dependency within the ‘host’ protein. Site variant H3-Bal9 showed consistently enhanced de novo host affinity over H3-Bal10 in its engagement with guest diols (down to low mM *K*_D_). A clear selectivity for diol-type (>11-fold terpene 4 > sugar 5) was also observed (Fig. 3b). In all cases, corresponding nonborylated histone H3 variants showed no measurable affinity for intermolecular guest, confirming the critical role of Bal (Supplementary Fig. 6). Moreover, attempted use of isolated small-molecule model Ac-Bal-NHBn bearing Bal as a host led only to ready oligomerization, precipitation and/or formation of complex mixtures not reflective of direct host–guest binding (Extended Data Fig. 7), confirming the critical role of placing Bal inside a suitable protein environment to exploit this de novo binding function.

This successful creation of de novo diol binding also suggested the potential for diol detection in more complex (for example, cellular) environments where varied diols (for example, cell-surface carbohydrates) are abundant. Representative glycosylated mammalian (CHO) cells were incubated with variants of the red-fluorescent protein mCherry (Fig. 3c). Flow cytometry revealed that while both nonborylated negative controls displayed no substantial cellular binding, site-selectively borylated mCherry-Bal131 showed clear cellular binding (>80% positive for mCherry-Bal131 versus <5% for nonborylated, Fig. 3d). In this way, mCherry, which has no inherent glycan- or cell-binding capacity, was bestowed with de novo cell-surface recognition. While detection systems based on fluorescent proteins as components in FRET-type sensors of small-molecule sugars have been developed^23^, this represents a de novo sugar-detecting fluorescent protein that uses direct binding.

### Reactive PLABP enables footprinting to Ser/d_1_-Ser residues

**Fig. 4:**
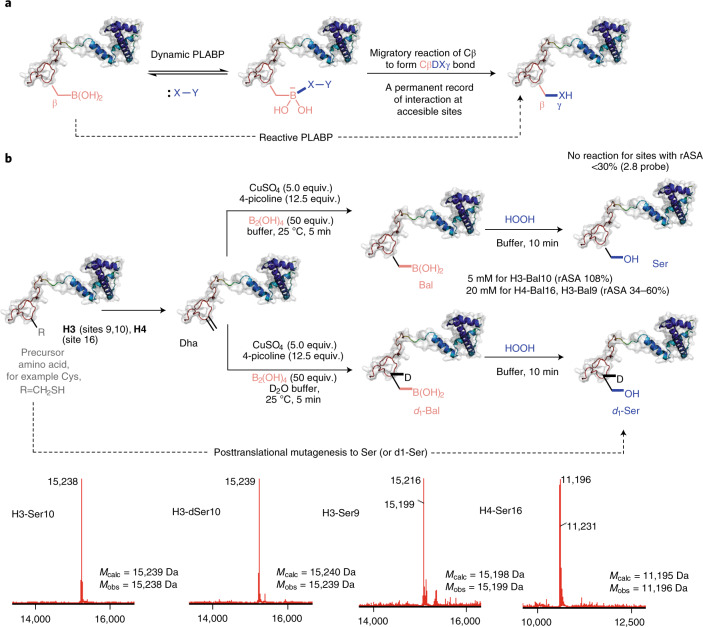
Reactive PLABP allows permanent ‘marking’ of PLABP with suitably reactive Lewis bases and enables post-translational Cβ–Oγ covalent bond formation. **a**, When dynamic PLABP occurs with suitable reactive alpha-nucleophiles (:X–Y), then migratory reaction allows subsequent recording of the interaction as a form of reactive PLABP that complements dynamic PLABP. **b**, When tested with ROS, Lewis base hydrogen peroxide reactivity correlated closely with reactive accessibility (rASA) values with a 2.8 Å probe (Supplementary Table 9). This, in turn, enables not only record of the interaction (footprinting) but also post-translational mutagenesis to Ser and its isotopolog d1-Ser through migratory Cβ–Bγ to Cβ–Oγ bond formation.

These successful uses of Bal in dynamic, reversible PLABP to convey binding function caused us to consider whether Bal could be exploited as both a dynamic and subsequently reactive Lewis acid motif (Figs. 1a and 4). Engagement with suitably reactive Lewis bases could allow reactive Lewis acid–base probe pairing. The permanence of this reactive transformation would then create a ‘footprint’ of LABP.

Several kinds of reactive oxygen species (ROS) are reactive alpha-nucleophiles with the potential to allow the conversion of Bal to Ser via PLABP followed by migratory C–O bond formation (Fig. 4a,b). Not only would this ‘record’ interaction with ROS, it would allow synthetic access to Ser from Dha via Bal, thereby extending post-translational mutagenesis^24^ by providing a method for β,γ-C–O formation (Fig. 4b).

A range of proteins with Bal at sites with different accessibilities (Methods, Supplementary Fig. 4 and Supplementary Table 8) were screened with representative ROS H_2_O_2_. Reactivity correlated with relative accessibility score analysis (rASA, Supplementary Tables 8 and 9); histone variant H3-Bal10 (relative accessibility 71–108% for probes of diameter 1.0–2.8 Å relative to Gly-XXX-Gly, Supplementary Fig. 16 and Supplementary Tables 8 and 9) showed complete reactivity (Fig. 4b) on exposure to only 5 mM H_2_O_2_ (Fig. 4b). Chemically induced Bal → Ser ‘footprinting’ was further confirmed by proteolytic-MS/MS analysis (Supplementary Fig. 2) and generation of an H3-Ser10 that retained the functional ability to be phosphorylated at Ser10 by the cognate histone Aurora B kinase (Supplementary Fig. 8). Less accessible sites in H4-Bal16 (rASA at 2.8 Å = 60%) and H3-Bal9 (rASA at 2.8 Å = 34%) required more concentrated 20 mM H_2_O_2_. Very low accessibility sites Npβ-Bal61 (rASA at 2.8 Å = 25%) or PstS-Bal197 (rASA at 2.8 Å = 0%) were unreactive or decomposed on prolonged exposure. These two methods—dynamic and reactive PLABP—therefore apparently allow determination of complementary information about interactions with intermolecular partners (for example, diols and ROS alpha-nucleophiles, respectively).

The overall reaction sequence Dha → Bal → Ser now allowed access to site-selectively α-deuterated d1-Ser variants (Fig. 4b). Nonexchangeable deuterium labels can act as spectroscopic and mechanistic probes^25,26^. Thus, when H3-Dha10 was borylated in deuterated buffer, site-selectively α-deuterated H3-d1-Bal10 was formed that was then converted to H3-d1-Ser10 (Fig. 4b). This product represents the site-selective isotopolog of WT H3 histone (a H3-Ser10 → d1-Ser10 ‘mutant’), an overall alpha-C-deuteration C–D bond formation at Ser.

Finally, given this demonstrated potential for intended oxidation of the Bal motif, we also probed the longer-term stability of Bal under background, potentially oxidative, conditions (Extended Data Fig. 4b,c). The isolated Bal residue proved stable in buffered solution under ambient conditions up to 100 h (Extended Data Fig. 4b); in a protein context, Bal oxidation in histone H3-Bal9 under ambient conditions in solution after 1 week was essentially comparable to that of background nonspecific oxidation (roughly 30%, Extended Data Fig. 4c).

### PLABP modulates both thermo- and proteolytic stability

Having demonstrated dynamic and reactive PLABP in single proteins, we explored application in more complex systems. Nonanchored borylation (Extended Data Fig. 1a) can play a stabilizing role in nature. This suggested the potential for design of protein stability, perhaps even in a chemoselective manner (for example, OH over SH engagement).

**Fig. 5:**
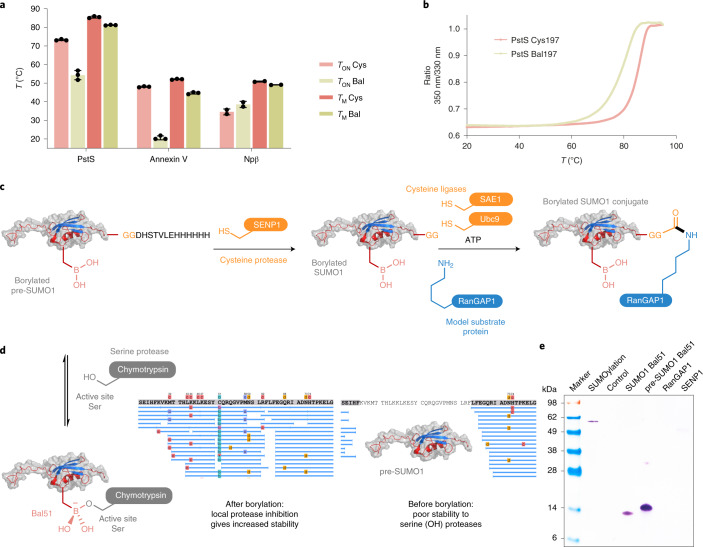
Exploitation of Bal in PLABP to engineer selective protein stability. **a**, Selective introduction of Bal into sites with a defined secondary structure allows engineering of thermostability. In sites with little inherent engagement but with potential for further interaction with nearby motifs, such as site 197 in PstS or site 316 in Annexin V, the introduction of Bal generates a large and positive ΔΔ*T* = *T*_ON_ − *T*_M_ > 0, consistent with local ‘unpicking’ of motifs yet without loss of global fold or function. However, in sites with already well-defined motif-to-motif engagement, such as site 61 in pentapeptide-repeat protein Npβ (which displays tri-valent beta-strand-to-beta-strand hydrogen-bond engagement), Bal apparently tightens interactions and reduces melting range *T*_ON_ − *T*_M_ (ΔΔ*T* < 0) (see Extended Data Fig. 8 for interactions). Values represent the mean ± s.d. from *n* = 3 individual experiments, *n* = 2 for Npβ. **b**, Comparison of representative thermal denaturation melting curves for PstS-Cys197 (red) and PstS-Bal197 (green) shows range widening on borylation. **c**, De novo chemoselective function that exploits recognition of ‘hard’ Lewis bases (for example, active-site Ser) over ‘soft’ ones (for example, active-site Cys) can be introduced into proteins to convey stability toward Ser-mediated proteolysis while retaining Cys-mediated proteolytic maturation. **d**, Borylated pre-SUMO1-Bal51 is locally protected from degradative serine protease (Ser-based catalytic triad) in its most susceptible central core bearing site 51. **e**, Electrophoretic analysis (performed in a qualitative single experiment) showing that pre-SUMO1-Bal51 can be successfully processed in a full SUMOylation cascade mediated by cysteine proteases and ligases, despite its engineered unnatural form.

We first probed the use of PLABP for altering thermostability (Fig. 5a). We chose three proteins with Bal placed at sites within different secondary-structure motifs with varying levels of intramolecular engagement with potential for PLABP-mediated stabilization: partially engaged in PstS-Bal197 (≥80% mono-substituted, start of alpha-helix-197-201, directed toward nearby alpha helices)^27^; strongly engaged in Annexin V-Bal316 (≥40% di-substituted alpha-helix site, end of C-terminal-alpha-helix-20 that lies parallel and interacts (C_316_ = O•••Arg285) with helices 18/19)^28^ and multiply engaged in Npβ-Bal61 (≥20% of all three forms non-, mono-, di-substituted in pentapeptide-beta-strand motif with three strand-to-strand (C = O•••HN)-bonds)^17^.

**Extended Data Fig. 8:**
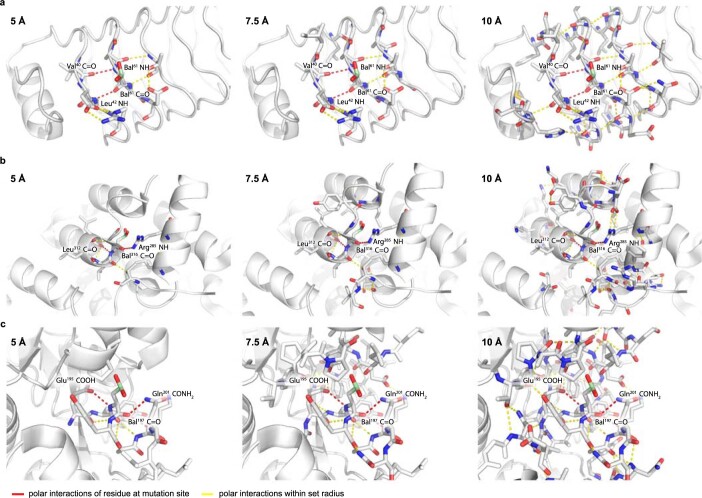
Polar interactions. Polar interactions in Npβ (**a**), Annexin V (**b**) and PstS (**c**) within a specific distance. PDB deposited crystal structures of Npβ (PDB-ID 2J8K), Annexin V (PDB-ID 1HVD) and PstS (PDB-ID 1A40) were mutated to Bal at the relevant site using PyMol. Polar interactions of the residue at the mutation site (red) and within a set radius (red) of 5 Å (left), 7.5 Å (middle) or 10 Å (right) of a putative Bal residue are displayed as dashed lines.

All maintained gross global structure and/or function (Extended Data Fig. 3 and Supplementary Tables 4 and 5). Each location altered stability differently (judged by differential scanning fluorimetry, Fig. 5a,b). Partially and strongly engaged sites PstS-Bal197 (ΔΔ*T* = Δ*T*_ON_ − Δ*T*_M_ = +16 °C) and Annexin V-Bal316 (ΔΔ*T* = +21 °C) saw an expansion in their melting ranges caused by insertion of Bal, whereas multiply engaged site Npβ-Bal61 saw a tightening with minimal change in *T*_M_ (Δ*T*_M_ = −2.0 °C, ΔΔ*T* = −6 °C). Such observed relaxation (ΔΔ*T* > 0) and tightening (ΔΔ*T* < 0) suggested selective disruption or tightening of cooperative structural motifs, respectively^29^, in a manner consistent with local PLABP (Fig. 2a). For example, the most-disrupted Annexin V-Bal316 case uses C_316_ = O as a Lewis-basic hydrogen-bond acceptor (with Arg285)^28^ that would compete with Bal316 intraresidue oxaborolane formation (Extended Data Fig. 8). Such local disruption of an alpha-helix was further supported by measurable loss of α-helical (−15.6 ± 0.9%) and gain of β-sheet (+6.4 ± 2.4%) character (Supplementary Table 5). Together, these data indicate that Bal can engender useful, localized intramolecular stability effects.

Next, we tested PLABP for stability control in multi-protein cascades. Bal’s modulation of thermolytic stability prompted us to explore proteolytic stability. We reasoned that borylation might selectively and locally inhibit (and hence confer resistance to) degradation by serine proteases while maintaining maturation susceptibility by other (for example, activating cysteine proteases) enzyme types. SUMO1, a critical regulator of various cellular processes^30^, is converted from zymogen precursor pre-SUMO1 (Fig. 5c) to matured SUMO1 before attachment to protein substrates. This SUMOylation cascade is mediated by protease SENP1 before SUMO-activating enzyme SAE1 and SUMO-conjugating enzyme Ubc9 transfer this matured SUMO to protein; all three use active-site Cys. We tested Bal in this full SUMOylation cascade (Fig. 5c,d). First, pre-SUMO1 was site-selectively borylated to create pre-SUMO1-Bal51. Next, in the presence of degradative serine protease (chymotrypsin), comprehensive digestion was observed for WT pre-SUMO1, showing poor proteolytic stability (Fig. 5d, right). However, insertion of Bal51 into the most susceptible central region (pre-SUMO1-Bal51) conferred greatly increased stability (Fig. 5d, left). At the same time, full compatibility of Bal with cysteine protease and ligases required for the SUMOylation cascade not only allowed unperturbed maturation of pre-SUMO1-Bal51 (by Cys-protease SENP1) but also use of resulting matured SUMO1-Bal51 in subsequent successful SUMOylation (by SAE1/Ubc9) of protein fragment RanGAP1 (Fig. 5e). In this way, Bal insertion into pre-SUMO1 allowed use of PLABP to convey selective proteolytic protection toward serine protease while allowing full zymogenic processing by cysteine-dependent enzymes. These observations are mechanistically consistent with small-molecule boronyl inhibitors of serine proteases^7^ but represent examples now of de novo engineering of such benefits of chemoselective function into intact proteins to control stability in complex cascades.

### Bal allows dative-contact induced chemical exchange (DICE)

**Fig. 6:**
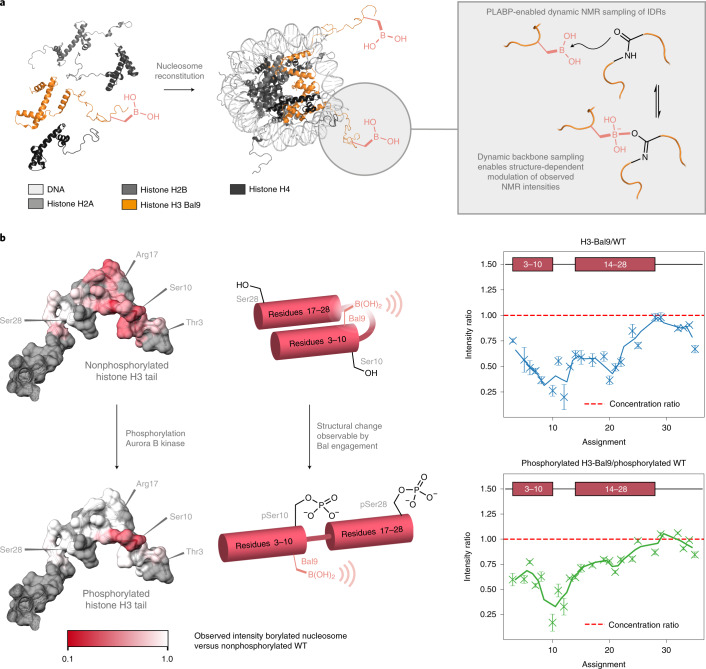
Exploitation of Bal in PLABP using the DICE method as a direct probe of IDRs found during epigenetic modification in nucleosomes. **a**, Assembly of nucleosomes containing histone H3-Bal9 allowed for exploitation of dynamic PLABP for the probing of structure via NMR using DICE. **b**, Nucleosome phosphorylation leads to a structural change in the tail of histone H3 that can be observed as a change in Bal-mediated backbone binding. Bal acts as a direct NMR ‘observer’ sensitive to structural change. Comparison of the NMR intensities of borylated and WT nucleosomes uniquely reveals both short- and long-range structural information. Comparison of intensity modulation after phosphorylation of both Bal-containing nucleosomes and WT nucleosomes reveals no notable differences, confirming Bal’s benign nature as an ‘observer’. Values represent the mean ± s.d. from *n* = 3 measurements of the same sample.

Initial investigations had revealed that Bal can flexibly engage peptidic backbone via PLABP (above and Extended Data Fig. 2c). This suggested that Bal might allow observation of contacts in more complex systems via protein NMR through modulation of signal intensity by PLABP-mediated chemical exchange, specifically DICE. Paramagnetic relaxation enhancement (PRE) measures long-range contacts (for example, <15–24 Å using nitroxide labels) in disordered proteins^31^. There are, however, two drawbacks: lack of quantitative short-range information and incorporation of a spin label-prosthetic that can itself affect residual structure. Generalized, benign, ‘short-and-long’ contact measurement (for example, via DICE) would therefore prove valuable. Notably, a small-molecule model (Fig. 2c) revealed propensity of the Bal-B_γ_ to associate with carbonyls transiently and reversibly, forming dative contacts needed for DICE under physiological conditions. In proteins, increased concentration of backbone-C = O could provide many such contacts. Moreover, since it is dynamic under these conditions, we reasoned that such C = O•••B_γ_ engagement would not alter underlying structural preferences, thereby acting as a benign ‘observer’ (Fig. 6a).

**Extended Data Fig. 9:**
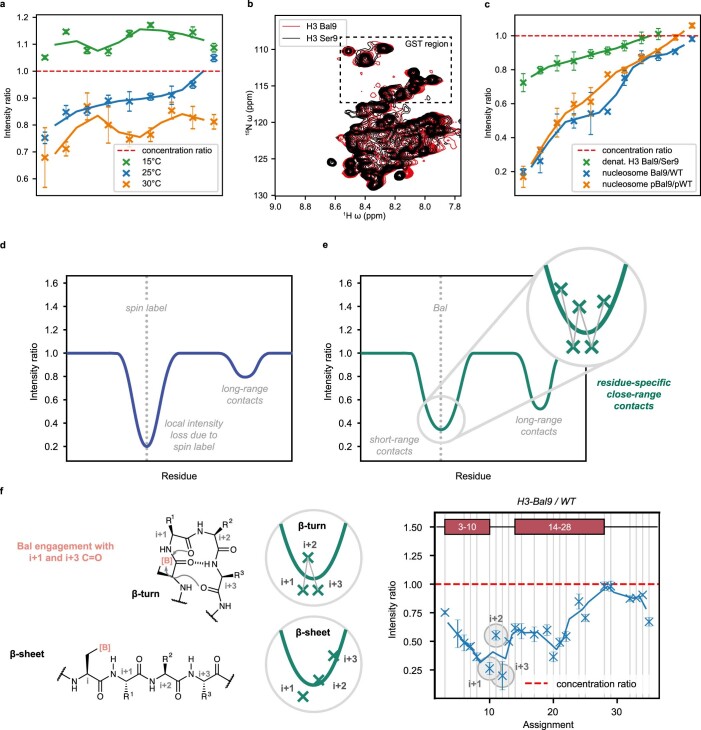
Further insights into DICE NMR. Further insights into DICE NMR. Glycine-Serine-Threonine (GST) residue intensity ratio comparison: (**a**) Intensity ratio comparison at different temperatures for denatured Histone H3 Bal9 and Histone H3 Ser9. (**b**) Contour plot for denatured Histone H3 Bal9 (red) and Histone H3 Ser9 (black) and definition of the GST region. (**c**) Intensity ratio comparison for denatured histone H3 (green, H3 Bal9 vs. H3 Ser9), the nucleosome (blue, H3 Bal9 containing nucleosome vs. WT nucleosome) and the phosphorylated nucleosome (orange, phosphorylated H3 Bal9 containing nucleosome vs. phosphorylated WT nucleosome). (**d**) Schematic representation of the intensity modulation in PRE using a spin label and through Bal-mediated PLABP via protein borylation (**e**). While introduction of a spin label leads to loss of signal intensity at residues close in sequence to the spin label, PLABP-enabled sampling allows for the simultaneous detection of close-range patterns as well as long-range contacts after borylation of a single site of interest. (**f**) The observed close-range contacts are in agreement with a β-turn in Histone H3 in a nucleosome context. Depicted values represent the mean; error bars show SD from n = 3 measurements of the same sample.

To test this, we first characterized Bal as an NMR probe in proteins that form higher-order complexes. First, comparative spectra of denatured H3-Bal9 and H3-Ser9 revealed similar signal intensities for most residues. Next, using ^13^CO-^15^N correlation spectra, six resonances in H3-Bal9 were found to be entirely absent, with no discernable change in the chemical shift of the remaining residues. These observations indicated that transient C = O•••B_γ_ contacts occur in a manner that induces intermediate exchange, thus generating loss of signal intensity. This was further confirmed by similar observations in N–H correlation spectra (Extended Data Fig. 9a); most notably, we observed that the signal loss increased markedly with temperature. Together these observations not only confirmed the benign nature of Bal as an ‘observer’ but also suggested that PLABP could be used to measure residual structure in disordered proteins by simultaneously examining local and remote contacts by using signal loss that correlates with the probability of contact between a backbone site and Bal.

Having established initial viability of Bal as a structural probe, we next tested it in the sampling of transient interactions. These are of special interest in the characterization of intrinsically disordered regions (IDRs) in proteins, which can naturally adopt an ensemble of different states^32^. Typically, PRE is used to detect only long-range contacts in such systems with the drawbacks noted above of label-induced artifacts and local short-range signal broadening (‘bleaching’)^32^. However, by contrast, use of DICE revealed region-selective loss of signal intensity in the manner anticipated (Extended Data Fig. 9) that probed not only transient long-distance contacts (permitting conclusions on residual structure), but also, and in contrast to PRE, DICE allowed simultaneous detection of short-range contacts.

We chose next the nucleosome as a more complex model system to test DICE using Bal as an NMR ‘observer’ of IDRs. Each nucleosome consists of a histone octamer (two each of H2A, H2B, H3, H4) wrapped by DNA^33^. Histones display disordered tails at their termini, which are subjected to a wide range of post-translational modifications (PTMs) suggested to regulate a multitude of cellular functions^33^. Of these, histone H3 phosphorylation at Ser10 by Aurora B kinase has been shown to cause two seemingly contradictory effects: chromatin condensation in mitosis and chromatin relaxation (and so gene expression) in interphase^34–36^. Moreover, such H3-Ser10 phosphorylation also seems to play a crucial role in switching mediated by the key heterochromatin protein 1 (HP1): HP1 is recruited by trimethylation of Lys9 in H3 whereas phosphorylation of Ser10 in H3 leads to its ejection^37^. While the central, structured part of the nucleosome has been described with high resolution^38^, detailed structural descriptions of the intrinsically disordered histone tails and so knowledge of the influence of PTMs (such as phosphorylation of Ser10 of H3) on structure is essentially lacking^33,39,40^.

We constructed a nucleosome containing ^15^N-labeled, borylated, histone H3 (Fig. 6a). Bal was placed strategically next to phosphorylation site Ser10, replacing Lys9 (to create ^15^N-H3-Bal9). Since Lys9 is implicated in Ser10-phosphorylation-dependent HP1 recruitment, we reasoned that this site would allow suitable direct observation of phosphorylation-induced structural change. In agreement with the established intermediate exchange regime, intensity comparison between the ^1^H-^15^N-HSQC spectra obtained for the fully assembled Bal-containing nucleosome (in comparison with its WT variant) immediately revealed a unique, residue-specific intensity modulation ‘double-dip’ pattern (Fig. 6b) corresponding to the presence of a residual structure consisting of two alpha helices (at H3 residues 3–10 and 17–28) connected by a beta-turn. Consistent with induction of tail structure within the intact nucleosome, this also represented a marked increase when compared to isolated, denatured H3 histone (Extended Data Fig. 9c). These observations were further supported by secondary chemical shift (SCS) analyses^41^ (Supplementary Fig. 9) where, in WT nucleosome, we saw strong helical preferences in the same regions identified by Bal as an observer probe.

Next, having used DICE to observe this transient structural motif, we tested the effect of phosphorylation. Notably, on treatment with Aurora B kinase, transient structure was released in favor of increased tail flexibility, leading to a reduction in PLABP (as observed by reduced Bal-induced intensity modulation via DICE, Fig. 6b); again, this was further confirmed by SCS analysis. The ratio of signal intensities ± phosphorylation did not alone reveal the patterns in structural rearrangements from the changes in signal intensities. Moreover, the pattern of signal intensity change was essentially the same for corresponding samples bearing ±Bal as a probe. Together, these combined ±Bal and ±phosphorylation comparisons confirmed that, under these conditions, the presence of Bal caused no perturbation to the relevant underlying structural equilibria. Simple comparison of intensity patterns of spectra obtained ±Bal allowed direct read out of the regions that are making contact via loss in signal intensity using the DICE method. In this way, Bal9 at the end of the first observed helix not only sampled long-range contacts, but also concurrently provided structural information in its direct vicinity (Extended Data Fig. 9e) leading to a mechanistic model where phosphorylation of H3 in the nucleosome opens up transient structure in the tail of H3 with seemingly diverse functional consequences (Fig. 6).

In all cases, control experiments (Fig. 6a and Supplementary Fig. 10) revealed no differences in either induced structures or phosphorylation activity by kinase between an intact WT nucleosome assembly and the H3-Bal9 variant (Fig. 6b), further confirming the benign nature of Bal as a structural probe. Together this highlighted the unique use of Bal as a protein NMR probe of residual structure using the DICE method that exploits PLABP-mediated dynamic, dative sampling of its spatial environment.

## Discussion

Despite being an element essential to many organisms, only a few boron-containing natural products have been described. In all of these, function is endowed by boron as its borate (–O–B–X) rather than boronate (–C–B–X) form. Examples include macrolides boromycin and borophycin^42^, bacterial signaling molecule AI-2 (ref. ^43^) and rhamnogalacturonan II (RG-II)^44^. Our results now indicate that endowing biomolecules with the potential for exploiting the C–B bond (‘anchoring’ of boron) can lead to wider application of the properties of boron as an element in biology.

**Extended Data Fig. 10:**
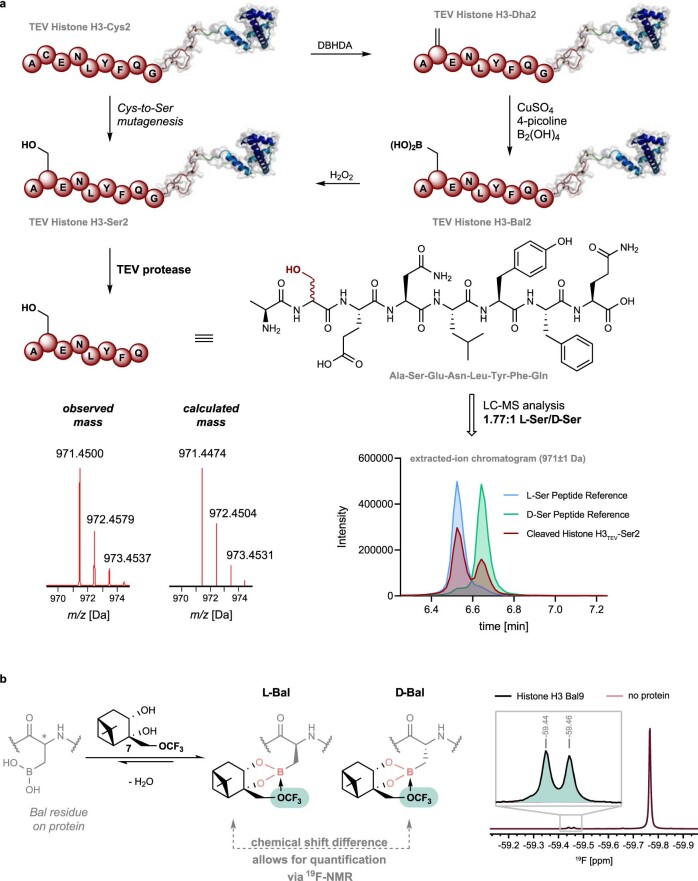
Determination of diastereomeric ratios after borylation. Determination of diastereomeric ratios after borylation. (**a**) Determination of the diastereomeric ratio of protein borylation by LC-MS: Borylation of Histone H3TEV-Dha2 followed by oxidation of the boronic acid to the corresponding H3_TEV_-Ser2 followed by TEV protease-mediated cleavage of and isolation of an eight-residue N-terminal fragment allowed for chromatographic separation of diastereomers and quantification of the diastereomeric ratio by LC-MS. A ratio of 1.77:1 in favor of l-Ser was detected in this specific case using the extracted-ion chromatogram (971 ± 1 Da). The identity of both diastereomers was confirmed by comparison to synthetic references assembled using solid-phase peptide synthesis with both l-Ser or d-Ser (see Methods for more details) (**b**) The diastereomeric ratio obtained upon borylation of Histone H3-Dha9 was determined using a trifluoromethoxy-modified diol as a chiral shift reagent. A diastereomeric ratio of approximately 1:1 was observed indicating minimal stereocontrol through proximal residues and the formation of equimolar d/l epimers.

Site-selective C_β_–B_γ_ bond formation in proteins now generates a minimal borylated residue, Bal, which displays diverse functional traits based on dative bonding. It should be noted that our analyses of diastereoselectivity indicate that in this method Bal (and/or product Ser) are formed as a mixture of l/d-epimers in diastereometric ratios of <2/1 to 1/1 (Extended Data Fig. 10 and Methods). This highlights that not only do the functions we observe arise from site epimers (either together or alone), but also, given the low observed substrate control in diastereometric ratio, highlights the future potential for ligand/catalyst control in stereoselectivity.

Bal now confers on proteins a de novo Lewis-acidic binding function that allows complementary dynamic and even reactive Lewis acid–base pairing with striking selectivity and high biocompatibility toward both intra- and intermolecular partners. Use of appropriately reactive Lewis bases allows migratory conversion of the C_β_–B_γ_ bond to C_β_–O_γ_ bond formation, in turn giving site-selective chemical, post-translational access to Ser and isotopolog d1-Ser. Such reactivity complements approaches in other systems where, for example, peroxynitrite-reactive chromophores containing *p*-boronophenylalanine in fluorescent proteins have been used to generate elegant sensor systems^45^.

Boronic acids are well-known, aqueous-compatible binding motifs that can exploit chelation-enhanced selectivity, such as for poly-ols over mono-ols, allowing recognition even in water^46,47^. Equivalent simple, small-molecule boronate hosts can occupy complex equilibria that prevent precise ‘programmed’ use. Yet the use of Bal in proteins seemingly allows more discrete control, free of oligomerization, precipitation and other confounding effects. Monodentate^48^ as well as more complex bi- and tridentate interactions (O,N and O,O,N) of small-molecule boronic acid-based enzyme inhibitors with Lewis-basic sidechains have been previously described^49^, but are solely based on boronates as external unanchored ligands making use of a predefined protein environment. Future applications of multi-faceted dual- and multi-receptor systems can now be envisaged. For example, both Annexin V-Bal316 and PstS-Bal197 remain active host receptors of their respective natural ligands (phosphatidyl-Ser and phosphate, respectively) while at the same time carrying a Bal residue for putative additional binding. While cooperativity between natural and de novo (Bal) binding was neither designed nor tested here, our observations of independent function suggest promise for de novo ‘dual-mode binding’. Moreover, given the precedent in small-molecule boronates^50^, designed cooperativity between functional groups in multiple unnatural amino acids, for example Bal and formyl-glycine, can also now be envisaged.

Given the advent of boron-based small-molecule drugs (including boron-containing peptides), such site-selective engineering of Bal into proteins could create unique proteins carrying engineered binding sites, tailored responses and selective stability for altered biological function as biotherapeutics^51,52^. Indeed, our creation here of proteins with tailored, selective responses toward thermolysis and proteolysis that still retain their biological functions and compatibility suggests many avenues.

The successful application of Bal in multi-protein systems (SUMOylation cascades or nucleosome phosphorylation) with full retention of natural biological use highlights the residue’s compatibility. For example, installation of Bal9 in place of Lys9 in histone H3 of a nucleosome did not impede native enzymatic phosphorylation (Supplementary Fig. 8) and also permitted detection of H3-pSer10 product using highly specific antibodies (Supplementary Fig. 11).

DICE–NMR via PLABP allowed detection of a transient structural consequences of Aurora B kinase-mediated phosphorylation on the histone H3 tail. This in turn suggests a more complex structural mechanism driving observed HP1 ejection than had previously been supposed^37^ (that is, more than simple occlusion of HP1 from methylated Lys9 by proximal pSer10).

Although the detailed mechanism of DICE–NMR is beyond the scope of the present study, drawing on our current data, Bal-B_γ_ is selectively associating and disassociating with carbonyl oxygen atoms; in the context of many protein C=O bonds, this is N-site chemical exchange. During association, chemical shifts of adjacent atoms will be altered (δω), which modulates signal intensities in a manner that depends on both δω and overall exchange rate (*k*_ex_). In protein-DICE–NMR the effect should be most clear through experiments involving an amide nitrogen or carbonyl oxygen; electron density around the carbonyl carbon is largely unaffected. The effect will be discerned as loss of signal (Extended Data Fig. 6b) and then on backbone HN, where signal intensities are modulated (Fig. 6b). A small population of the boron-associated state (<1%) can, in principle, lead to the substantial intensity changes observed here. Temperature dependence suggests fast-intermediate exchange with *k*_ex_/δω > 1. Overall, every atom transiently modulated by Bal will lead to reduction in signal intensity that reflects chemical accessibility of the site and so, in turn, reveals residual structure in disordered chains. In principle, such Bal associations might alter structural preferences. However, we do not see any substantial change in the chemical shift of observed residues and see similar structural arrangements using both DICE and PREs; there is therefore no indication, in this case, that underlying structural preferences are altered.

Finally, it should also be noted that the chemical roles and reactivities of organoboranes are wide in scope^46^ and so Bal in proteins may now enable further, more diverse applications. For example, while we have demonstrated de novo binding function (dynamic PLABP) toward naturally derived ligands and stoichiometric reactivity (for example, Bal → Ser through reactive PLABP) we can envisage future use of host–guest interactions with nonnatural ligands and even as activatable or de novo catalytic residues^53^.

### Influence of concentration

A stock solution of Ac-Bal-NHBn (30 mg ml^−1^) in NaP_i_ buffer (20 mM, pH 8.5, D_2_O) was prepared and the pH was adjusted to pH 8.5 using 1 M NaOH in D_2_O. A two-fold dilution series was prepared by dilution of the stock solution into the same volume of NaP_i_ buffer (20 mM, pH 8.5, D_2_O). The pH of the resulting solutions was checked before analysis via NMR spectroscopy (Supplementary Table 12).

### NMR studies on [^15^N]-labeled model substrate

NMR studies were conducted on a Bruker AVIII HD 600 equipped with a Prodigy N_2_ broadband cryoprobe. The [^15^N]-labeled model substrate was dissolved in NaP_i_ buffer (20 mM, pH 8.5, D_2_O) and the pH was adjusted to pH 8.5 using 1 M NaOH in D_2_O (Supplementary Figs. 15–18).

### General protocol for protein borylation

Stock solutions of copper(II) sulfate, 4-picoline and tetrahydroxydiboron were prepared in milliQ water directly before the reaction was conducted.

To a solution of dehydroalanine-mutant protein (roughly 1 mg ml^−1^) in borylation buffer (100 mM NaP_i_, 3 M Gdn∙HCl, pH 7.0) were added aliquots of the previously prepared stock solutions of (copper(II) sulfate, 5–60 equiv.; 4-picoline, 12.5–150 equiv.; tetrahydroxydiboron, 50–600 equiv.). The mixture was briefly vortexed and incubated at room temperature for 10 min. Protein samples can be purified by gel filtration or dialysis. Conversions were determined by liquid chromatography–MS (LC–MS) and protein recovery was quantified by absorbance at 280 nm (A_280_) spectrophotometry (see Supplementary Note 3 for details).

### Inductively coupled plasma–MS (ICP–MS) determination of residual copper

#### Sample preparation

Samples (250 µg) were reconstituted in denaturing buffer (NaP_i_ 100 mM, 3 M Gdn∙HCl, pH 7.0) and diluted into 2% HNO_3_ in mQ water to a total volume of 10 ml (40× dilution).

#### Sample analysis

Samples were analyzed on a Perkin Elmer NexION 2000B ICP–MS. Calibration was conducted using dilutions from certified reference material (River Water, SLRS-6). All samples were spiked with 1 ppb Rh and the internal standard was used to adjust for instrument drift. All samples were measured in duplicate.

### Intact protein mass spectrometry

Intact protein LC–MS was performed on a Waters Xevo G2-S quadrupole time of flight (QTOF) spectrometer equipped with a Waters Acquity ultrahigh-performance liquid chromatography (UPLC), on a Waters Xevo G2-XS QTOF spectrometer equipped with a Waters Acquity UPLC or an AB Sciex TripleTOF 6600 spectrometer equipped with a Shimadzu high-performance liquid chromatography (HPLC) system. Separation was achieved using a Thermo Scientific ProSwift RP-2H monolithic column (4.6 × 50 mm) using water + 0.1% formic acid (Solvent A) and acetonitrile + 0.1% formic acid (Solvent B) as mobile phase at a flow rate of 0.3 ml min^−1^ and running a 10 min linear gradient as follows: 5% Solvent B for 1 min, 5 to 95% Solvent B over 6 min, 95 to 5% Solvent B over 1 min, 5% Solvent B for 2 min. Spectra were deconvoluted either using MassLynx (Waters) and the ‘MaxEnt1’ deconvolution algorithm or Analyst (AB Sciex) using the ‘Reconstruct Protein’ algorithm. Conversions were calculated from peak intensities (Waters) or peak areas (AB Sciex) dividing the value for the product by the sum of the values for products and (residual) starting material. Impurities present before the reaction were not considered. For details, see Extended Data Fig. 5 and Supplementary Note 3.

### Protein MS/MS

An aliquot of the protein sample was denatured by addition of 8 M urea solution to a final concentration of 4 M. TCEP reducing agent was added to a final concentration of 10 mm. The sample was diluted fourfold with 50 mm TEAB buffer solution. Digestion buffer (50 mm TEAB, 50 mm TCEP, 2 mm EDTA) was added (one tenth of sample volume). ArgC was added in a ratio 1:20 and the sample was incubated at 37 °C overnight. The sample was analyzed using a standard MS/MS procedure.

LC–MS/MS data were analyzed in PEAKS Studio v.8.5 or PEAKS Studio X (Bioinformatics Solutions Inc.). The data were searched against the known protein sequence. The following settings were used: precursor mass tolerance at 15 ppm; fragment mass tolerance at 0.5 Da; maximum number of missed cleavages, 3 and nonspecific cleavage at one end of the peptide. The following variable PTM were selected in addition to the modification of interest: oxidation (+15.99 Da, Met), deamidation (+0.98 Da, Asn, Gln) and carbamylation (+43.01 Da, Lys). An FDR or 1% on peptide level was applied.

### Bal stability assay

#### Ac-Bal-NHBn stability

Ac-Bal-NHBn (5 mg) was dissolved in deuterated buffer (100 mM NaP_i_ in D_2_O, pH 7.0, 500 µl) and analyzed via quantitative ^1^H-NMR (eight scans, d1 = 60 s) on a Bruker NEO 400 nanobay spectrometer equipped with a 5 mm *z* gradient broadband multinuclear SMART probe. Five different time points were taken (12, 24, 48, 72, 96 h). The absolute integrals of the characteristic C_β_ protons of boronoalanine (1.0–1.3 ppm) were determined using TopSpin 4 (Bruker BioSpin). No change in absolute integrals was detected, confirming that Ac-Bal-NHBn is stable for at least 96 h under the used conditions (Extended Data Fig. 4).

#### Histone H3-Bal9 stability

Lyophilized Histone H3-Bal9 was dissolved in denaturing buffer (NaPi 100 mM, 3 M Gdn∙HCl, pH 7.0) at 1 mg ml^−1^. The solution was incubated under ambient atmosphere at room temperature in a microcentrifuge tube for 1 week. LC–MS analysis revealed the presence of Histone H3-Ser9 (33%) as a result of Bal oxidation. In agreement with ambient oxidation, further oxidized species were observed (M + 16 Da) (Extended Data Fig. 4).

### Determination of stereoselectivity using chiral shift reagent

#### Determination of the enantiomeric ratio of﻿﻿ Ac-Bal-NHBn

Ac-Bal-NHBn was dissolved in binding buffer (40 mM NaP_i_, 5 M urea, pH 7.0, 10% D_2_O) at a final concentration of 500 µM. Then 10 equiv. of chiral shift reagent were added and the sample was vortexed. The sample was transferred to a NMR tube and analyzed on a Bruker AVIII HD 500 MHz spectrometer equipped with BBFO SMART probe (2,060 scans, d1 = 2 s). A ratio of approximately one-to-one was detected after integration of the peaks (Supplementary Fig. 26).

#### Determination of the diastereometric ratio on Histone H3-Bal9

Histone H3-Bal9 was dissolved in binding buffer (40 mM NaP_i_, 5 M urea, pH 7.0, 10% D_2_O) at a final concentration of 144 µM. Then 10 equiv. of chiral shift reagent were added and the sample was vortexed. The sample was transferred to a NMR tube and analyzed on a Bruker AVIII 600 MHz spectrometer equipped with a Prodigy N2 broadband cryoprobe (3,500 scans, d1 = 2 s). A ratio of approximately one-to-one was detected after integration of the peaks (Extended Data Fig. 10).

### Determination of stereoselectivity using proteolytic LC–MS

Cloning, expression and purification of Histone H3_TEV_-Cys2 was done as follows. The plasmid encoding the sequence for expressing the WT protein *Xenopus laevis* Histone H3.3, lacking cysteines was used as a PCR template. A tobacco etch virus (TEV) protease consensus sequence (ENLYFQG) was added between the second and third residues of the Histone sequence (AR(TEV)TKQ…), and the second residue was mutated to Cys (R to C) by including the desired mutations in the forward primer, as well as necessary restriction enzyme sites. The primers used for PCR were:

**Table:** 

**Forward primer**	5′-GGTGGTCCATGGCCTGTGAGAACCTGTACTT CCAGGGCACCAAGCAGACCGCCCGTAAATCC-3′
**Reverse primer**	5′-GGTGGTGGATCCCTAAGCCCTCTCGCCTCGG-3′

After amplification, the PCR product was digested with BamHI and NcoI along with a pET3d vector, ligated into the vector and transformed into XL-10-Gold cells (Agilent). The plasmid and resulting protein sequence were confirmed via Sanger Sequencing, and the H3-Cys2, N-TEV Histone was expressed and purified as described for the other histones.

#### Dha formation, borylation and oxidation

Dha formation, borylation and oxidation were conducted as described for Histone H3 Cys9 and Histone H3 Cys10.

#### TEV digestion

TEV cleavage was conducted on 200 µl of Histone in HEPES buffer (5 mg ml^−1^ Histone H3-(N-terminal TEV)-Ser2 (from Bal), 100 mm HEPES, pH 7.4). Then 25 µl of 10× TEV Protease Reaction Buffer (New England BioLabs, B8035S, Lot 10078918) and 20 µl of TEV Protease (New England BioLabs, P9112S, Lot 10077416) were added and the mixture was incubated overnight at 37 °C shaking at 600 r.p.m. The mixture was used directly for LC–MS analysis.

#### LC–MS analysis

Samples (TEV digest or reference peptides) were analyzed on a Waters Xevo G2-XS QTOF mass spectrometer equipped with a Water Acquity UPLC. Separation was achieved on a ACQUITY UPLC BEH C18 column (Waters) using water + 0.1% formic acid (Solvent A) and MeCN + 0.1% formic acid (Solvent B) as mobile phase at a flow rate of 0.2 ml min^−1^ and using a 10 min linear gradient as follows: 5% Solvent B for 1 min, 5 to 25% over 9 min. The recorded data were processed in MassLynx 4.1 (Waters) by generating extracted-ion chromatograms (971 ± 1 Da) followed by integration of the corresponding peaks. Comparison with authentic peptide references allowed for assignment of the l-Ser and d-Ser epimers.

### Reactive PLABP for the synthesis of Ser and d1-Ser mutants

#### Nondeuterated

To a solution of purified borylated protein in borylation buffer (NaP_i_ 100 mM, pH 7.0, 3 M Gdn∙HCl) was added H_2_O_2_ to a final concentration of 5 to 20 mM and the mixture was incubated at room temperature for 10 min. The concentration of H_2_O_2_ required depends on the specific protein.

#### Deuterated

Dehydroalanine-bearing protein in borylation buffer (100 mM NaP_i_, 3 M Gdn∙HCl, pH 7.0) was lyophilized and redissolved in D_2_O. This procedure was repeated twice. The lyophilized protein was then reconstituted with D_2_O and borylated according to the general protocol to yield a *d1*-Bal mutant. Oxidation to the deuterated serine residue was performed as described above.

### Calculation of residue accessibility

The solvent-accessible surface area was calculated using FreeSASA^54^. The Lee and Richards algorithm^55^ was used on Protein Data Bank (PDB) structures having the site of interest mutated to Cys. The number of slices was set to 100, atom radii from NACCESS were used and different probe sizes (1.00, 1.40 and 2.80 Å) were used. The absolute values obtained for the solvent-accessible surface area of the respective Cys residues were set in relation to a tripeptide Gly-Cys-Gly using the value published by Tien et al.^56^ (Supplementary Fig. 4).

### Determination of oxaborolane and boronate formation by ^1^H-NMR

Small-molecule model compound Ac-Bal-NHBn (2) (see Supplementary Note for synthesis and purification) was dissolved in buffer (20 mM NaP_i_ in D_2_O) at a concentration of 20 mg ml^−1^. The pH was adjusted with concentrated DCl or 1 M NaOH in D_2_O to obtain seven samples having a pH ranging from 5.4 to 12.0. Standard ^1^H-NMR experiments were conducted on each sample and species were quantified by integration of the C_β_-H signals, which are distinctly different for the boronic acid and the oxaborolane. The data were processed with GraphPad Prism v.8.0.0 (GraphPad Software Inc.) and fitting was performed using a sigmoidal standard curve.

### Determination of poly-ol binding to Bal proteins by NMR

Borylated protein was dissolved in NMR binding buffer (NaP_i_ 40 mM, pH 7.0, 5 M urea, 10% D_2_O). Fluorinated poly-ol (see Extended Data Fig. 7a for poly-ols used and Supplementary Note 1 for their synthesis) was added from a stock solution in dimethylsulfoxide (DMSO) (typically 10 equiv.), the mixture was transferred to an NMR tube and analyzed via ^19^F-NMR on a Bruker Avance III HD 600 MHz NMR spectrometer equipped with a Prodigy N_2_ broadband cryoprobe. Peaks for bound poly-ols appeared downfield of the nonbound species. Binding was quantified by peak integration for a known protein, and fluorinated poly-ol concentrations and experiments were conducted in triplicate (Supplementary Fig. 7).

Controls were conducted as follows: competition with nonfluorinated triol 3, Histone H3-Bal9 was incubated with 10 equiv. of diol 4 as described previously and a NMR spectrum was recorded. A second NMR spectrum was recorded in presence of triol 3 (200 equiv.) acting as a competitor (Supplementary Fig. 22). Carr–Purcell–Meiboom–Gill filtered NMR, Histone H3-Bal9 was incubated with 10 equiv. of diol 4 as described previously and a NMR spectrum was recorded. A second NMR spectrum was recorded using a 200 ms Carr–Purcell–Meiboom–Gill filter (Supplementary Fig. 23). ^19^F-NMR titration of Histone H3-Bal9 with diol 4, Histone H3-Dha9 was borylated following the standard procedure. The mixture was purified via dialysis into mQ H_2_O and lyophilized before being resuspended in NMR binding buffer buffer (NaPi 40 mM, pH 7.0, 5 M urea, 10% D_2_O). The protein was titrated with diol 4 (stock solution in DMSO) covering a concentration range from 100 µM to 64 mM and ^19^F-NMR spectra were recorded (d_1_ = 2 s, 3,072 scans) (Supplementary Fig. 24). Binding of FDGal (6) to Ac-Bal-NHBn (2), Ac-Bal-NHBn (2) (31.0 mg, 117 µmol, 10.0 equiv.) and FDGal (6) (2.14 mg, 11.7 µmol, 1.00 equiv.) were dissolved in NaP_i_ buffer (25 mM, pH 8.0, 10% D_2_O) and analyzed via ^19^F-NMR spectrometry (Supplementary Fig. 25).

### Determination of protein melting curves

Melting curves were recorded on a Prometheus NT.48 differential scanning fluorimeter (NanoTemper Technologies). Samples were loaded into high sensitivity quartz capillaries. Unfolding was detected during heating using a linear temperature gradient (20 to 95 °C, 1 °C min^−1^) and an excitation power of 50% (100% for Npβ). Melting curves were recorded at three different concentrations in phosphate buffer (20 mM NaP_i_, 50 mM NaF, pH 7.4). The data were analyzed using PR.Stability Analysis v.1.0.2 software (NanoTemper Technologies) and GraphPad Prism v.8.0.0 (GraphPad Software Inc.).

### Stability in SUMOylation cascade

#### Pre-SUMO1 maturation

Here, 200 µl (0.67 mg ml^−1^) of a solution in TRIS buffer (100 mM TRIS base, pH 7.0) of pre-SUMO1-Bal51 were incubated with 10 µl of SENP1 (0.14 mg ml^−1^) at 37 °C for 4 h. Then 20 µl of fresh Ni-NTA resin was washed with water (2 × 500 µl) and added to the reaction mixture. The mixture was incubated for 15 min and the resin was removed by filtration. The filtrate was concentrated to 50 µl using a VivaSpin concentrator (molecular weight cutoff (MWCO) 5 kDa) and used directly for in vitro SUMOylation.

#### SUMOylation of a model protein

In vitro SUMOylation of RanGAP1 fragment was conducted using an in vitro SUMOylation assay kit (Abcam) on a 20 µl scale using matured SUMO1-Bal51 and following the manufacturer’s general protocol. A control experiment was conducted in the absence of matured SUMO1-Bal51. RanGAP1 SUMOylated with SUMO1-Bal51 was detected via western blot using the supplied primary antibody and a polyclonal goat antirabbit IgG alkaline phosphatase secondary antibody. Controls and reaction were analyzed via SDS–PAGE (10% bis-TRIS gel, 150 V, 45 min, MES buffer). The proteins were transferred to a nitrocellulose membrane. The membrane was blocked for 1 h in TBS-T (pH 7.5) supplemented with 5% BSA. The membrane was incubated with the primary rabbit anti-SUMO1 polyclonal antibody (dilution 1:1,000) for 1 h at room temperature and washed three times for 5 min before the secondary goat antirabbit polyclonal antibody alkaline phosphatase conjugate was added (dilution 1:1,000). The membrane was incubated for another hour, washed three times and SUMO1 was visualized using NBT/BCIP substrate solution (ThermoFisher) (Supplementary Figs. 29 and 30).

### Annexin V binding

#### FITC-labeling of Annexin V-Bal316

A solution of Annexin V-Bal316 in phosphate buffer (500 μl, 0.15 mg ml^−1^, 2.09 nmol, 1.00 equiv.) was exchanged into sodium bicarbonate buffer (100 mM, pH 9.4) and concentrated to 200 μl using VivaSpin 500 concentrators (5 kDa MWCO, Satorius). Then 20 μl (81.6 μg, 209 nmol, 100 equiv.) of a freshly prepared solution of fluorescein 5(6)-isothiocyanate (FITC) (4.1 mg) in DMSO (1,000 μl) was added to the protein solution and the mixture was vortexed briefly. The mixture was shaken (400 r.p.m.) in the dark at 37 °C for 2 h and purified using a PD Minitrap G-25 size exclusion column (GE Healthcare) previously equilibrated with HEPES buffer (10 mM HEPES, 140 mM NaCl, pH 7.4) according to the manufacturer’s instructions. The purified solution of FITC-labeled Annexin V-Bal316 was concentrated using VivaSpin 500 concentrators (5 kDa MWCO, Satorius) and stored on ice until used. The dye-to-protein ratio was determined spectrophotometrically on a NanoDrop 8000 spectrophotometer (Thermo Scientific) measuring at *λ* = 280 nm and *λ* = 494 nm. A dye-to-protein ratio of 0.95 was detected.

#### Flow cytometry

Jurkat cells (5 × 10^5^ cells per ml, 10 ml) were incubated with etoposide at a final concentration of 25 µM (addition of 10 µl of 25 mM stock in DMSO) for 390 min at 37 °C. Approximately 100,000 cells were pelleted and resuspended in 100 µl of Annexin V binding buffer (10 mM HEPES, 150 mM NaCl, 2.5 mM CaCl_2_, pH 7.4). Then 45 µl of Annexin V-Bal316-FITC or 10 µl of commercial Annexin V-FITC (Miltenyi Biotech) were added and the cells were incubated at room temperature for 15 min and stored on ice before being analyzed by flow cytometry.

Flow cytometry was performed on a BD FACSCalibur cell analyzer using BD FACSDiva software. A minimum of 10,000 cells per sample was analyzed using the 488 nm laser and a 530/30 nm bandpass filter.

### mCherry binding

#### Determination of absorption maxima

Absorption spectra of different mCherry mutants were measured on a BMG Labtech SPECTROstar Nano (full spectrum, 350–900 nm, 1 nm interval) using 200 µl of protein solution in a clear 96-well plate at a concentration of approximately 10 µM. Measured optical density (OD) values were used to calculate the relative absorbance of the respective sample. The absorbance maximum was determined to be at 586 nm for all three mutants—mCherry-Cys131, mCherry-Sulfonium131 and mCherry-Bal131.

#### Flow cytometry

To 100 µl of CHO-WT cells (roughly 10^6^ cells) in fluorescence activated cell sorting (FACS) buffer (Dulbecco’s phosphate-buffered saline, pH 8.0, 2% FBS) were added 300 µl of mCherry mutant (0.28 mg ml^−1^, 10 µM) or 300 µl of FACS buffer (control). The cells were shaken on ice for 20 min at 300 r.p.m. The samples were centrifuged at 400*g* for 3 min at 4 °C. The liquid was removed and the cell pellet was resuspended in 1,000 µl of FACS buffer, centrifuged at 400*g* for 3 min at 4 °C and the buffer was removed. The washing step was repeated once before the cell pellet was suspended in 400 µl of FACS buffer. Flow cytometry was performed on a BD LSRFortessa X-20 cell analyzer using BD FACSDiva 8.0 software. A minimum of 10,000 cells per sample was analyzed using the 561 nm laser and a 610/20 nm bandpass filter. The 640 nm laser in combination with a 780/60 nm bandpass filter was used as a reference channel. The data were analyzed using FlowJo v.10 software.

### PstS competition assay

A stock solution of Phosphate Sensor (ThermoFisher Scientific) (1.0 µM) and sodium phosphate (1.0 µM) in TRIS buffer (20 mM, pH 7.6) was prepared. Then 10 µl of this stock solution were mixed with 10 µl of a solution of PstS-Bal197 in TRIS buffer in a black clear bottom 384-well plate in triplicates. Final PstS-Bal197 concentrations of 2.28 nM to 37.4 µM were investigated. Measurements were captured on a BMG Labtech ClarioSTAR plate reader (BMG Labtech) with excitation at 430 nm (8 nm bandwidth) and emission at 450 nm (8 nm bandwidth). The data were fitted to a four-parameter dose response curve using GraphPad Prism v.8.0 (GraphPad Software).

$$\begin{array}{l}{{{{y}}}} = {{{\mathrm{Bottom}}}} + \left( {{{{\mathrm{Top}}}} - {{{\mathrm{Bottom}}}}} \right)\\/\left( {1 + \left( {\left( {{{{{x}}}}^ \wedge {{{\mathrm{HillSlope}}}}} \right)/\left( {{{{\mathrm{IC}_{{50}}}}}^ \wedge {{{\mathrm{HillSlope}}}}} \right)} \right)} \right)\end{array}$$

### Protein CON–NMR

Lyophilized samples were dissolved in 330 µl of NMR buffer (50 mM NaP_i_, 3 M Gdn∙HCl, 5% D_2_O, pH 7.0). NMR experiments on the triple labeled denatured histone H3 samples were performed on a 14.1T Varian Inova spectrometer equipped with a 5 mm *z* axis gradient triple resonance room temperature probe. Three-dimensional HNCO experiments were recorded using a random sparse sampling schedule that contained ^1^H/^13^C/^15^N 1,600/50/30 complex points from a maximum of 1,600/150/50 points. The spectral widths were 7,993/849/1,600 Hz, with eight scans recorded per free induction decay (FID) and an interscan delay of 0.6 s for a total acquisition time of 12 h and 24 min. The spectra were processed in NMRpipe^7^ with the SMILE reconstruction algorithm using linear prediction, a sine-bell window function, zero filling and phase correction (Extended Data Fig. 9).

### Nucleosome NMR study

#### Nucleosome assembly

Octamer reconstitution, nucleosome assembly and 145 bp DNA products were all performed essentially as previously described^57^ using borylated Histone H3 where applicable.

#### Cloning and mutagenesis

The H3-SUMO fusion was created by overlap PCR using primers given in the table below. The fusion protein consisted of an N-terminal His-tagged SUMO for solubility followed by a ‘Tobacco Etch Virus nuclear inclusion A endopeptidase’ (TEV) recognition site and a C-terminal H3 tail (residues 1–44). TEV protease can cleave a variant of its optimal recognition sequence (ENLYFQ\S) in which the C-terminal serine is replaced with alanine (ENLYFQ\A). Digestion of the construct resulted in a WT tail with an N-terminal alanine without any additional amino acids. The WT *Xenopus laevis* histone plasmids were a kind gift from R. Klose (Oxford, Department of Biochemistry). The ‘601’ 145 bp was a kind gift from J. Min.

Primers used for mutagenesis and cloning:

**Table:** 

**H3_F**	GCCCGTACCAAGCAGACCGCC
**H3_R**	AGTGCG CTCGAG CTA GCC GGG CCG GTA ACG GTG AGG
**SUMO_F**	ATGCTC CAT ATG GGC AGC AGC CAT CAT CAT CAT CAT C
**SUMO_R**	GGC GGT CTG CTT GGT ACG GGC TTG GAA GTA CAG GTT TTC CTC GAT ACC ACC

#### Histone expression and purification

WT *Xenopus laevis* core histones H2A, H2B and H4 were expressed and purified in the same manner as that used to produce Histone H3 used for borylation chemistry. Successful expression and purification were validated via intact protein LC–MS (below) and SDS–PAGE analysis. The various ^15^N-labeled Histone H3 proteins were produced via expression in M9 minimal media, followed by borylation at relevant sites, oxidation of Bal10 to give WT mimic Ser10 or phosphorylation with Aurora B kinase.

#### Histone octamer reconstitution

Lyophilized histones were dissolved in Unfolding Buffer (6 M Gdn∙HCl, 5 mM dithiothreitol (DTT), 20 mM Tris∙HCl pH 7.5) in equimolar ratios (20 µM, 72 nmol each) and incubated at room temperature for 1 h. The solution was transferred to a dialysis cassette (ThermoFisher, Slide-A-Lyzer Dialysis Cassette, MWCO 7 kDa) and dialyzed three times against Refolding Buffer (2 M NaCl, 1 mM EDTA, 1 mM DTT, 10 mM Tris∙HCl pH 7.5) for 2 h each time at 4 °C, with the last dialysis taking place overnight. The resulting solution was concentrated (VivaSpin 6, MWCO 5 kDa) to approximately 1 ml and purified via size exclusion chromatography (Cytiva, HiLoad 16/600 Superdex 200 pg), eluting with Refolding Buffer. Fractions containing the octamer and absent of other residual histone species (tetramer, dimer and monomers) were revealed by SDS–PAGE analysis. The purified octamer was concentrated to at least 2 mg ml^−1^ (measured by A_280_ spectrophotometry with the combined histone molecular weight and extinction coefficients) and stored at 4 °C until further use.

#### Widom 601 145 bp large-scale DNA preparation

The plasmid containing the 8 × 145 bp Widom 601 sequence in a pUC vector was transformed into XL-10-Gold Ultracompetent cells (Agilent) following the manufacturer’s instructions and spread on a Luria Bertani (LB) -agar plate containing ampicillin (100 µg ml^−1^) and incubated overnight at 37 °C. The next day, colonies were transferred to four starter culture tubes containing Terrific Broth (Sigma Aldrich) and ampicillin and shaken overnight at 250 r.p.m., 37 °C. The day following that, four 1 l flasks containing the same media were inoculated with the starter cultures and shaken for 24 h at 250 r.p.m., 37 °C. The cells were pelleted (5,000*g*, 8 min, 4 °C), and the pellet resuspended in Alkaline Lysis Solution 1 (50 mM glucose, 10 mM EDTA, 25 mM TRIS∙HCl pH 8.0, 60 ml per liter of media producing the cell pellet). Twice the volume of Alkaline Lysis Solution II (200 mM NaOH, 1% v/v SDS) was added and the mixture shaken vigorously to remove any clumps, before incubation on ice for 20 min with occasional additional shaking. Alkaline Lysis Solution III (4 M NaOAc, 2 M AcOH, 210 ml per liter of media producing the cell pellet) was added and the solution inverted ten times before a further 20 min incubation on ice. The mixture was centrifuged (10,000*g*, 30 min, 4 °C), and the supernatant filtered through Miracloth (Merck Millipore), followed by the addition of 0.52 volumes of isopropanol. After a 15 min incubation at room temperature, the precipitated DNA was pelleted by centrifugation (11,000*g*, 30 min, 20 °C) and allowed to dry open to the air for 1 h. The pellet was fully dissolved in TE 10/50 (10 mM TRIS, 50 mM EDTA pH 8.0, 40 ml) and split between two 50-ml falcon tubes. RNase A (NEB, 10 mg ml^−1^, 120 µl) was added and the solutions incubated overnight at 37 °C. The next day, any precipitant was removed by centrifugation (10,000*g*, 15 min, 20 °C) and the DNA in the supernatant was precipitated by adding 1/5 the original volume of 4 M NaCl and 2/5 the original volume of 40% PEG6000. After mixing at 37 °C for 5 min and incubating on ice for 30 min, the DNA was pelleted by centrifugation (3000 g, 20 min, 4 °C). The pellet was dissolved in TE 10/0.1 (10 mM TRIS, 0.1 mM EDTA pH 8.0, 15 ml) by rocking overnight at 37 °C. To digest the 145 bp inserts out of the plasmid backbone, the following reagents were added: TRIS∙HCl (to 6 mM from a 1 M stock, pH 8.0), MgCl_2_ (6 mM from a 4 M stock), NaCl (150 mM from a 4 M stock) and DTT (1 mM from a 1 M stock). Then, the restriction enzyme EcoRV-HF (10,000 units, NEB) was added and the solution incubated at 37 °C for 20 h. The digest was checked for completion at this stage by analyzing an aliquot via agarose gel electrophoresis. Following digestion, 0.192 volumes of NaCl (4 M) and 0.346 volumes of PEG6000 were added, and the solution incubated on ice for 1 h to precipitate the larger plasmid backbone. The precipitant was pelleted by centrifugation (27,000*g*, 20 min, 4 °C) and the 145 bp insert containing supernatant was poured into ice cold ethanol (125 ml) to induce precipitation with a 20 min incubation on ice. The supernatant/ethanol mixture was divided among four 50 ml centrifuge tubes and centrifuged (27,000*g*, 20 min, 4 °C), and the pellet air dried for 20 min and dissolved in TE 10/0.1 (5 ml). An aliquot of the 145 bp insert containing pellet was analyzed at this stage by agarose gel electrophoresis to validate efficient separation from the larger vector backbone DNA. Next, 2/5 volumes of phenol and 1/5 volumes of chloroform were added to the dissolved insert solution and vortexed to mix. The suspension was then centrifuged (5,000*g*, 10 min, room temperature) to separate phases, and the upper aqueous later extracted with a pipette and mixed 1:1 v/v with chloroform. The mixture was vortexed and centrifuged as before and the aqueous layer subjected to one more round of chloroform (1:1 v/v) purification. The final aqueous layer was extracted by pipette, and a 1/10 volume of NaOAc (3 M, pH 5.2) and 3 volumes of ethanol were added. The solution was vortexed and allowed to precipitate at −20 °C for several days. The precipitant was pelleted by centrifugation (10,000*g*, 30 min, 4 °C), air dried for 30 min and resuspended in water (1 ml, 4.5 mg of 145 bp insert final yield).

#### Nucleosome reconstitution

Histone octamers (1.5 mg, 2 mg ml^−1^ in Refolding Buffer) were mixed with an equimolar amount of 145 bp DNA (2 mg ml^−1^ in 2 M KCl) and dialyzed (4 °C, 2 h per dialysis step) against Reconstitution Buffer (1 mM EDTA, 1 mM DTT, 10 mM TRIS∙HCl pH 7.5) containing 2, 0.85, 0.65 and last 0.25 mM KCl. The resulting solution was then centrifuged (5 min, 10,000 r.p.m., 4 °C) to remove any precipitant, if any, and nucleosome aliquots (10 µl) were heat-shifted (1 h at 4, 37 or 55 °C) to ensure a homogenously populated nucleosome with respect to the position of the DNA on the histone octamer. The incubated aliquots were then analyzed by Native–PAGE (NOVEX TBE 6% in 0.5% TBE buffer, prerun for 90 min at 150 V, 4 °C), to reveal the presence of both DNA (Invitrogen SYBR Safe Staining) and octamer (Coomassie Blue staining) in the nucleosome band. The heat-shifted samples revealed that every reconstituted nucleosome required no further heat shifting beyond 4 °C, as evident by the lack of several close nucleosome bands resolving to one band at higher temperature incubations. Commassie Blue staining revealed that most protein was contained in the nucleosome band, while SYBR Safe staining revealed that in every case, there was some residual free 145 bp DNA remaining in the sample. Any lost octamer was presumed to have crashed out during dialysis, evident by the presence of some precipitant in most cases postdialysis (Supplementary Figs. 31–36).

#### NMR sample preparation

Nucleosomes were prepared for NMR by dialyzing three times (2 h each, last one overnight, 4 °C) into NMR buffer (1 mM EDTA, 10 mM NaP_i_ pH 6.5), the last dialysis was performed with a slightly more concentrated buffer stock that, when 5% v/v D_2_O was added, gave the desired NMR buffer concentration. After dialysis, the nucleosomes were concentrated (VivaSpin 500, MWCO 5 kDa) to 190 µl, and then 10 µl of D_2_O was added. The samples were transferred to 3 mm Bruker Match NMR tubes. After NMR measurement, some nucleosomes were further modified via phosphorylation with Aurora B kinase. These nucleosomes were reevaluated by Native–PAGE (without further heat shifting) to confirm integrity and dialyzed again as described above into the NMR buffer for further NMR measurement.

### Phosphorylation with Aurora B kinase

#### Phosphorylation of histone H3-Ser10

A solution of Histone H3-Ser10 (either native or from oxidized Histone H3-Bal10) was prepared in kinase buffer (10 mM TRIS, 30 mM NaCl, 1 mM DTT, 3 mM MgCl_2_, 2 mM ATP, 10 mM β-glycerophosphate, pH 7.5). To 500 µl of this solution in a microcentrifuge tube were added 0.5 µg of Aurora B kinase (Abcam, ab51435), and the mixture was vortexed. The tube was incubated at 30 °C, 400 r.p.m. for 2 d before the samples were analyzed by SDS–PAGE and western blot.

#### Nucleosome phosphorylation

After NMR measurement, samples were retrieved from the NMR tubes and diluted with 1,800 µl of kinase buffer (10 mM TRIS, 30 mM NaCl, 1 mM DTT, 3 mM MgCl_2_, 2 mM ATP, 10 mM β-glycerolphosphate, pH 7.5). In the case of Bal-containing nucleosomes, dilution was conducted in a nitrogen filled glove box with kinase buffer that was degassed overnight (4 °C). This precaution was taken to prevent oxidation of Bal to Ser, which was observed on Histone H3-Bal9 on prolonged exposure to kinase buffer at elevated temperature (30 °C). Recombinant human Aurora B kinase (3 µg, Abcam, ab51435) was added and the mixture was shaken (400 r.p.m.) at 30 °C for 48 h, after which the samples were again prepared for NMR measurement.

### H3-tail peptide

#### Preparation

The production of the labeled H3-tail peptide was performed as described by Lundstrom et al.^58^ with minor modifications. The plasmid encoding the H3(1–45)-SUMO construct were transformed into BL21(DE3)pLysS chemically competent *Escherichia coli* and grown on LB-agar plates containing ampicillin (100 mg l^−1^) and chloramphenicol (34 mg l^−1^). The next evening, 2–15 ml of LB medium containing the same antibiotics was inoculated with one colony from the plate. These were grown overnight and then centrifuged, and the cells were resuspended in 100 ml of M9 medium (containing ^15^NH_4_Cl and [^1^H, ^12^C] or [^1^H, ^13^C] glucose). These were grown for 4 h at 37 °C and then 50 ml of preculture was added to 700 ml of M9 medium. These cultures were grown at 37 °C until OD_600_ = 0.6–0.8 and then expression was induced by addition of 0.5 mm isopropyl-β-d-thiogalactoside. Expression was continued at 18 °C for 16 h, after which the cells were collected by centrifugation. Cell pellets were stored at −80 °C until purification.

#### Purification

Cells were resuspended in 35 ml of lysis buffer (1-PBS, 400 mM NaCl, 20 mM imidazole, 5 mM beta-meracaptoethanol (BME), Complete protease inhibitor cocktail) and were lysed by sonication. The lysate was then cleared by centrifugation (20 min, 20,000 r.p.m.) and transferred to a 50 ml super-loop. The lysate was loaded onto a 1 ml HisTrap HP column (GE Healthcare; 17-5247-01) equilibrated in loading buffer (25 mM TRIS, 500 mM NaCl, 20 mM imidazole, 5 mM BME, pH 7.5) at 1 ml min^−1^. The protein was eluted using a linear gradient from 0–100% B (loading buffer + 400 mM imidazole) in 20 CV. The fractions containing the desired protein were then concentrated to 1.5 ml and further purified by size exclusion chromatography (Superdex S75 16/60 in 25 mM TRIS, 300 mM NaCl, 1 mM DTT, 1 mM EDTA, pH 7.5). Fractions containing the desired protein were concentrated to 1.5–2 ml, concentration was measured using A_280_ spectrophotometry and TEV protease (1:20 Abs) was added. The cleavage was allowed to proceed overnight (16 h) and the mixture was then purified again by size exclusion (Superdex S75 16/60, 20 mM NaP_i_, 100 mM NaCl, 1 mM EDTA and 5 mM BME, pH 7.0). The fractions containing the H3 fragment were then pooled, concentrated and the buffer was exchanged by VivaSpin (3.5 kDa MWCO) where necessary.

### General NMR acquisition and processing for nonborylated nucleosomes

Measurements were performed on a Bruker Avance 700 MHz spectrometer with TopSpin v.3.2. Data were processed using NMRpipe^59^ and SPARKY^60^. Peak and exponential fitting was performed using FuDa (https://www.ucl.ac.uk/hansen-lab/fuda/). Samples were measured in 3 mm Bruker Match NMR tubes. Unless otherwise stated, spectra were measured in NMR buffer (10 mM NaPi, 1 mM EDTA, complete protease inhibitor cocktail, pH 6.5). Nucleosome 1H-15N HSQC spectra (including ^1^H and ^15^N R_2_) were recorded at 298 K with 1024 (1H)-80 (15 N) complex points and a sweep width of 20 ppm in the indirect dimension. For ^1^H-^15^N HSQCs, the standard Bruker pulse sequence ‘hsqcetfpf3gp’ was used. A recycle delay of 1 s was used. For 32 scans in the direct dimensions the acquisition time was roughly 36 min.

#### ^1^H R_2_ measurements

^1^H R_2_ rate measurements were performed with a custom pulse sequence based on the standard Bruker ^1^H-^15^N HSQC (hsqcetfpf3gp) with the variable delay in the initial Insensitive Nuclei Enhanced by Polarization (INEPT) methods based on a sequence reported by Mal et al.^61^. With 160 scans in the direct dimension the total acquisition time was 18 h.

#### ^15^N R_2_ measurements

^15^N R_2_ rate measurements were performed using the HSQCT2ETF3GPSI standard Bruker pulse sequence. An interscan delay of 5 s was used. With 64 scans in the direct dimension the total acquisition time for four planes was 24 h. The set delay period of 16.96 ms was repeated 1, 5, 10 and 15 times. The separate two-dimensional (2D) planes with different delays were combined and processed using NMRPipe^59^ and peak fitting was performed with FuDa (https://www.ucl.ac.uk/hansen-lab/fuda/).

#### Chemical shift analysis

Random coil chemical shifts were calculated using a webserver at http://spin.niddk.nih.gov/bax/nmrserver/Poulsen_rc_CS, SCS are given by Δ*δ* = *δ*_observed_ − δ_(random coil)_.

### PRE

For the attachment of a spin label, the histone H3 cysteine mutant (H3-Cys36) containing nucleosome was first dialyzed into no-salt buffer containing no DTT for 4 h. Residual DTT was removed by VivaSpin. Then 10 equiv. (1-oxyl-2,2,5,5-tetramethyl-Δ3-pyrroline-3-methyl) methanethiosulfonate (Toronto Research Chemicals O875000; dissolved in MeCN) was added and left to incubate for 1 h at room temperature and then overnight at 4 °C. Subsequently, excess methanethiosulfonate was removed by Vivaspin. After *R*_2_ rates were recorded with the spin label, the spin label was reduced by the addition of 10 mM sodium ascorbate and incubation at 4 °C overnight. PRE rates were calculated from ^1^H *R*_2_ rates using:

$${\Gamma}_2 = R_{2_{{\mathrm{red}}}} - R_{2_{{\mathrm{ox}}}}$$

### NMR acquisition for DICE–NMR

NMR experiments on borylated nucleosome samples were performed on a Bruker Avance III HD 950 MHz spectrometer equipped with a 5 mm TCI Cryoprobe. 2D ^1^H-^15^N bond-selective excitation short transient (BEST)-transverse relaxation optimized spectroscopy experiments from the standard Bruker library were recorded with ^1^H (^15^N) 1024 (128) complex points, spectral widths of 10,416 Hz (3,465 Hz), maximum acquisition times of 98.3 ms (36.0 ms), 256 scans per FID with an interscan delay of 0.2 s for a total acquisition time of 2 h 52 min. To calculate errors, these spectra were recorded three times for the following samples: WT nucleosome, phosphorylated WT nucleosome, Histone H3-Bal9-containing nucleosome, denatured Histone H3-Ser10 (WT) and denatured Histone H3-Bal9. One measurement was made of the phosphorylated Histone H3-Bal9-containing sample, and errors for this sample were calculated by using the average peak noise found in the other spectra. These spectra were processed using a sine-bell window function, zero filling and phase correction in both dimensions. The peaks were then manually identified in SPARKY^60^ by mapping the assignments from Zhou et al.^62^. With the exception of phosphorylated Histone H3-Bal9-containing nucleosome, the intensities were averaged across the three spectra and the standard deviations calculated. All nucleosome samples were measured in NMR buffer (1 mM EDTA, 10 mM NaP_i_ pH 6.5, 5% v/v D_2_O). Denatured histones were measured in denaturing NMR buffer (50 mM NaP_i_, 3 M Gdn∙HCl, 5% v/v D_2_O, pH 7.0).

#### Concentration determination

To quantify the concentration of nucleosomes, ^1^H spectra were recorded with 8,192 complex points and a sweep width of 10,417 Hz for an acquisition time of 786 ms. Then 32 scans per FID were recorded with an interscan delay of 1 s. The spectra were processed in NMRPipe^59^ using a sine-bell window function, zero filling, phase correction and linear baselining and then the methyl regions (0.62 to 0.98 p.p.m.) were extracted for comparison. To quantify the concentration of denatured H3 histones, ^1^H spectra were recorded with 31,248 complex points and a sweep width of 10,417 Hz for an acquisition time of 3 s. Next, 32 scans per FID were recorded with an interscan delay of 1 s. The spectra were processed in NMRPipe^59^ using a sine-bell window function, zero filling, phase correction and linear baselining and then extracted the methyl regions (0.7 to 1.14 p.p.m.) for comparison (Supplementary Fig. 14).

#### Temperature variation of denatured histones

The same experimental protocol was then applied to denatured Histone H3-Ser9 and denatured Histone H3-Bal9 samples. 2D ^1^H-^15^N BEST-transverse relaxation optimized spectra were recorded three times at three temperatures: 288, 298 and 303 K. Processing again involved a sine-bell window function, zero filling and phase correction in both dimensions. In the absence of assignments, isolated peaks in the glycine-serine-threonine region were peak picked in SPARKY^60^. Peak intensities were averaged and standard deviations calculated for errors. Denatured histones were measured in denaturing NMR buffer (50 mM NaP_i_, 3 M Gdn∙HCl, 5% v/v D_2_O, pH 7.0) (Extended Data Fig. 9).

### Reporting Summary

Further information on research design is available in the Nature Research Reporting Summary linked to this article.

## Methods

### Small-molecule chemical synthesis

Detailed synthetic procedures are available in Supplementary Note 1.

### pH dependency

The pH dependency of oxaborolane formation on model compound Ac-Bal-NHBn was observed by ^1^H-NMR using solutions of Ac-Bal-NHBn (20 mg ml^−1^) in NaP_i_ buffer (20 mM in D_2_O). The pH was adjusted with concentrated DCl and 1 M NaOH in D_2_O. Quantification was conducted by integration of the C_β_-H signals, which are distinctly different for the boronic acid and the oxaborolane. The data were processed with GraphPad Prism v.8.0.0 (GraphPad Software Inc.) and fitting was performed using a sigmoidal standard curve (Supplementary Table 11) (where IC_50_ is half-maximum inhibitory concentration):

$${{{{y}}}} = {{{\mathrm{Bottom}}}} + \left( {{{{\mathrm{Top}}}} - {{{\mathrm{Bottom}}}}} \right)/\left( {1 + 10^ \wedge \left( {\left( {{{{\mathrm{logIC}_{{50}}}}} - {{{{x}}}}} \right) \times {{{\mathrm{HillSlope}}}}} \right)} \right).$$

## Online content

Any methods, additional references, Nature Research reporting summaries, source data, extended data, supplementary information, acknowledgements, peer review information; details of author contributions and competing interests; and statements of data and code availability are available at 10.1038/s41589-021-00883-7.

## Supplementary information

Supplementary Information Supplementary Figs. 1–37, Tables 1–13 and Notes 1–3.

Reporting Summary

## Data Availability

Raw protein LC–MS, raw protein MS/MS and raw NMR data (protein and small-molecule), raw nucleosome NMR data and primary numerical data for all graphical plots are deposited in the open-access depositories ORA-data (partial) (https://ora.ox.ac.uk/objects/uuid:ca409cd6-36d0-4788-a3c8-083e32bf0e18) and Zenodo (full) (10.5281/zenodo.4900115). The following publicly available protein structures were used: acrA (PDB ID 2FMA), Annexin V (PDB ID 1HVD), nucleosome, Histone H3 and Histone H4 (PDB ID 1KX5), mCherry (PDB ID 4ZIN), Npβ (PDB ID 2J8K), PanC (PDB ID 1N2E), pre-SUMO1 (PDB ID 1A5R) and PstS (PDB ID 1A40).

## 2-acetylamino-*N*-benzyl-acrylamide

PubChemID: 442107713
InChIKey: OVRHVEWKMXSKAL-UHFFFAOYSA-N
MDL Molfile
Chemdraw file

## (2-acetamido-3-(benzylamino)-3-oxopropyl)boronic acid

PubChemID: 442107714
InChIKey: MXGIKOYISHZQJV-UHFFFAOYSA-N
MDL Molfile
Chemdraw file

## (1*R*)-(–)-myrtentriol

PubChemID: 442107692
InChIKey: RJVDPCROFUUYEV-BDNRQGISSA-N
MDL Molfile
Chemdraw file

## fluorodeoxy-(1*R*)-(–)-myrtenoldiol

PubChemID: 442107693
InChIKey: SEFRNOCYFDMMOH-BDNRQGISSA-N
MDL Molfile
Chemdraw file

## methyl 5-deoxy-5-fluoro-β,D-ribofuranoside

PubChemID: 442107694
InChIKey: QAQHSPJOFQDUID-KVTDHHQDSA-N
MDL Molfile
Chemdraw file

## 2-deoxy-2-fluoro-D-galactopyranose

PubChemID: 442107695
InChIKey: ZCXUVYAZINUVJD-GASJEMHNSA-N
MDL Molfile
Chemdraw file

## trifluoromethoxy-(1*R*)-(–)-myrtenoldiol

PubChemID: 442107696
InChIKey: ZQOWOHJCFXPELW-BDNRQGISSA-N
MDL Molfile
Chemdraw file

## [^15^N]benzamide

PubChemID: 442107697
InChIKey: KXDAEFPNCMNJSK-VJJZLTLGSA-N
MDL Molfile
Chemdraw file

## [^15^N]benzamine

PubChemID: 442107698
InChIKey: WGQKYBSKWIADBV-VJJZLTLGSA-N
MDL Molfile
Chemdraw file

## 2-acetylamino-*N*-benzyl-acryl-[^15^N]-amide

PubChemID: 442107699
InChIKey: OVRHVEWKMXSKAL-KCKQSJSWSA-N
MDL Molfile
Chemdraw file

## (2-acetamido-3-(benzyl-[^15^N]-amino)-3-oxopropyl)boronic acid

PubChemID: 442107700
InChIKey: MXGIKOYISHZQJV-UJKGMGNHSA-N
MDL Molfile
Chemdraw file

## [^15^N]acetamide

PubChemID: 442107701
InChIKey: DLFVBJFMPXGRIB-LBPDFUHNSA-N
MDL Molfile
Chemdraw file

## [^15^N]-acetamidoacrylic acid

PubChemID: 442107702
InChIKey: UFDFFEMHDKXMBG-PTQBSOBMSA-N
MDL Molfile
Chemdraw file

## 2-acetyl-[^15^N]-amino-*N*-benzyl-acrylamide

PubChemID: 442107703
InChIKey: OVRHVEWKMXSKAL-UJKGMGNHSA-N
MDL Molfile
Chemdraw file

## (2-acet-[^15^N]-amido-3-(benzylamino)-3-oxopropyl)boronic acid

PubChemID: 442107704
InChIKey: MXGIKOYISHZQJV-XPOOIHDOSA-N
MDL Molfile
Chemdraw file

## (1*R*)-(–)-myrtenoldiol benzylether

PubChemID: 442107705
InChIKey: DIQICRZKUBAXPR-PNBKFKSVSA-N
MDL Molfile
Chemdraw file

## (1*R*)-(–)-myrtenoldiol benzylether acetonide

PubChemID: 442107706
InChIKey: SLBSOWLONCCMBY-PDOICOKGSA-N
MDL Molfile
Chemdraw file

## (1*R*)-(–)-myrtenoldiol acetonide

PubChemID: 442107707
InChIKey: HVLSOSXWKJANBZ-ORXSELOVSA-N
MDL Molfile
Chemdraw file

## fluorodeoxy-(1*R*)-(–)-myrtenoldiol acetonide

PubChemID: 442107708
InChIKey: DNLVGEIUSXFFJJ-ORXSELOVSA-N
MDL Molfile
Chemdraw file

## 1,3,4,6-tetra-*O*-acetyl-2-deoxy-2-fluoro-D-galactopyranose

PubChemID: 442107709
InChIKey: KIPRSJXOVDEYLX-RQICVUQASA-N
MDL Molfile
Chemdraw file

## methyl 2,3-*O*-isopropylidene-5-*O*-(toluenesulfonyl)-β,D-ribofuranoside

PubChemID: 442107710
InChIKey: IAPMZKRZMYQZSW-KBUPBQIOSA-N
MDL Molfile
Chemdraw file

## methyl 2,3-*O*-isopropylidene-5-deoxy-5-fluoro-β,D-ribofuranoside

PubChemID: 442107711
InChIKey: KYCHVROSCLBCEN-WCTZXXKLSA-N
MDL Molfile
Chemdraw file

## trifluoromethoxy-(1*R*)-(–)-myrtenoldiol acetonide

PubChemID: 442107712
InChIKey: OEYLFECUZRWBDT-ORXSELOVSA-N
MDL Molfile
Chemdraw file

## Extended data

are available for this paper at 10.1038/s41589-021-00883-7.
