# Publisher Correction: Post-translational insertion of boron in proteins to probe and modulate function

**DOI:** 10.1038/s41589-021-00957-6

**Published:** 2021-12-13

**Authors:** Tim A. Mollner, Patrick G. Isenegger, Brian Josephson, Charles Buchanan, Lukas Lercher, Daniel Oehlrich, D. Flemming Hansen, Shabaz Mohammed, Andrew J. Baldwin, Véronique Gouverneur, Benjamin G. Davis

**Affiliations:** 1 Department of Chemistry, Chemistry Research Laboratory, University of Oxford, Oxford, UK; 2 Department of Chemistry, Physical and Theoretical Chemistry Laboratory, University of Oxford, Oxford, UK; 3 Neuroscience Medicinal Chemistry, Janssen Research and Development, Beerse, Belgium; 4 Division of Biosciences, University College London, London, UK; 5 Department of Biochemistry, University of Oxford, Oxford, UK; 6 The Rosalind Franklin Institute, Oxfordshire, Oxford, UK

**Keywords:** Chemical tools, Proteins, NMR spectroscopy, Chemical modification

Correction to: *Nature Chemical Biology* 10.1038/s41589-021-00883-7, published online 1 November 2021.

In the version of this article initially published, there was a composition error in Fig. 5a where, for the first, fifth and ninth columns correlating to “*T*_ON_ Cys” in the key, the pink color dropped out of the columns and top key. The HTML and PDF versions of the article have been corrected online.

