# Supplementary material for: Post-translational insertion of boron in proteins to probe and modulate function
Source: Nat Chem Biol. 2021 Nov 1;17(12):1245–61. doi: 10.1038/s41589-021-00883-7 (PMC8604732; doi:10.1038/s41589-021-00883-7)
Supplement: Supplementary file 1 — Supplementary Figs. 1–37, Tables 1–13 and Notes 1–3. [file 41589_2021_883_MOESM47_ESM.pdf]

nature portfolio

https://doi.org/10.1038/s41589-021-00883-7

Supplementary information

Post-translational insertion of boron in proteins to probe and modulate function

In the format provided by the authors and unedited

Supplementary Information

Post-translational insertion of boron in proteins to probe and modulate function

Table of contents

1 Supplementary Figures 1

2 Supplementary Tables 37

3 Supplementary Notes 51

3.1 Supplementary Note 1: Small Molecule Synthesis 51
3.1.1 General Considerations 51
3.1.2 Chromatography 51
3.1.3 Analytical Techniques 51
3.1.4 Synthetic Procedures 54
3.1.5 NMR Spectra 78

3.2 Supplementary Note 2: Protein Sequences 109
3.2.1 Histone H3-Cys10 109
3.2.2 Histone H3-Cys9 110
3.2.3 Annexin V-Cys316 111
3.2.4 Npβ-Cys61 112
3.2.5 preSUMO1-Cys51 113
3.2.6 mCherry-Cys131 114
3.2.7 PstS-Cys197 115
3.2.8 Histone H4-Cys16 116
3.2.9 AcrA-Cys123 117
3.2.10 panC-Cys44 118
3.2.11 panC-Cys47 119

3.3 Supplementary Note 3: Protein Chemistry 120

3.4 Protein Expression and Purification 120
3.4.1 Histone H3-Cys10 120
3.4.2 Histone H3-Cys9 123
3.4.3 [^{13}C-^{15}N]Histone H3-Cys10 126
3.4.4 [^{15}N]Histone H3-Cys9, [^{13}C-^{15}N] Histone H3-Cys9 and [^{15}N]Histone H3-Cys10 129
3.4.5 Annexin V-Cys316 134
3.4.6 Npβ-Cys61 137
3.4.7 pre-SUMO1-Cys51 140
3.4.8 mCherry-Cys131 143
3.4.9 PstS-Cys197 145
3.4.10 panC 147

3.4.11 AcrA-Cys123 ... 148
3.4.12 Histone H4-Cys16 ... 149
3.4.13 Histone H3-Cys10 ... 149
3.4.14 [^{15}N]Histone H3-Cys10 ... 154
3.4.15 [^{13}C-^{15}N]Histone H3-Cys10 ... 156
3.4.16 Histone H3-Cys9 ... 159
3.4.17 [^{15}N]Histone H3-Cys9 ... 162
3.4.18 Annexin V ... 164
3.4.19 Npβ ... 166
3.4.20 PanC ... 168
3.4.21 pre-SUMO1 ... 171
3.4.22 mCherry ... 173
3.4.23 PstS ... 175
3.4.24 AcrA ... 177
3.4.25 Histone H4 ... 179

4 References ... 181

1 Supplementary Figures

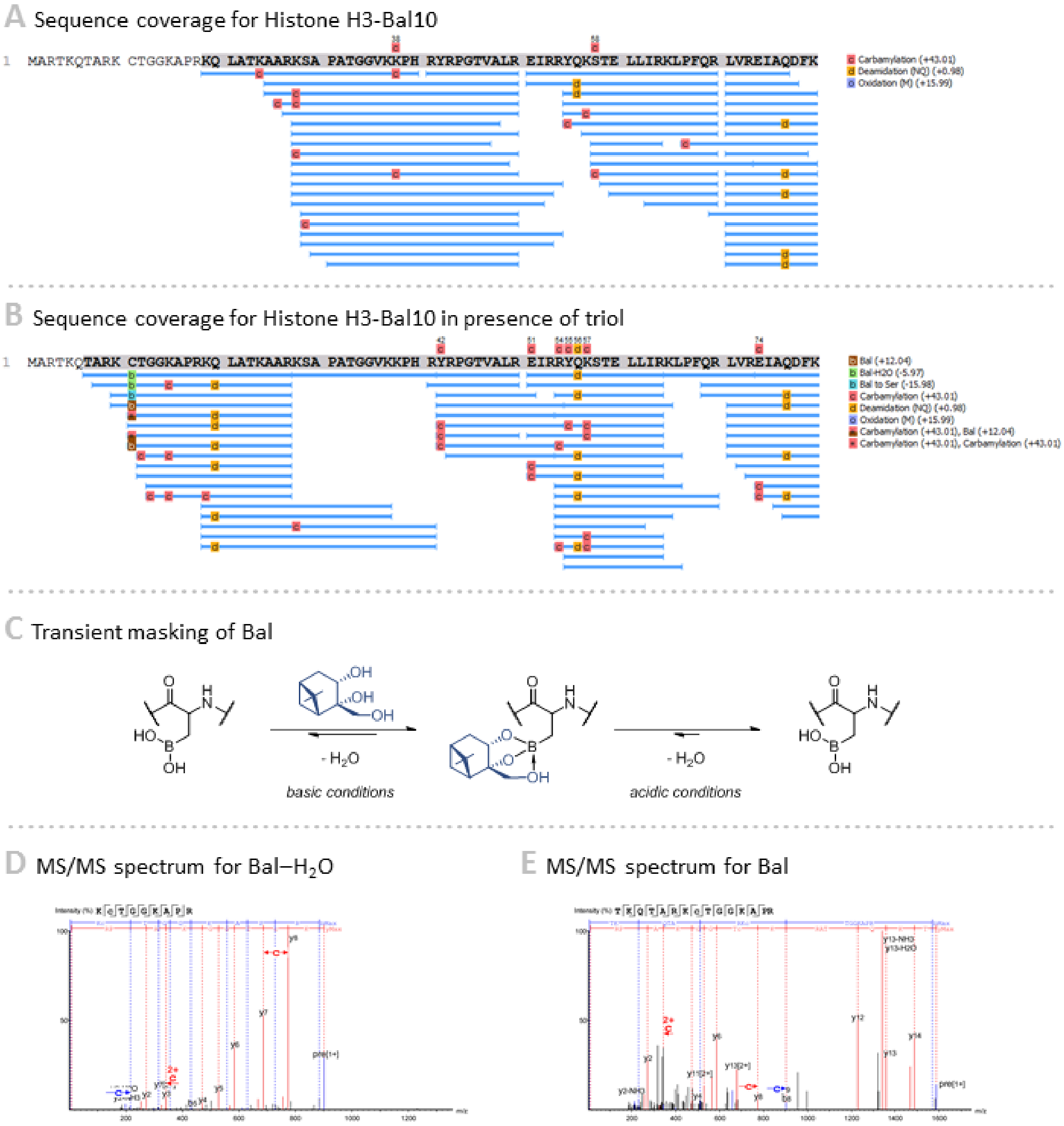

Supplementary Figure 1: MS/MS analysis of Histone H3-Bal10: A) Sequence coverage of Histone H3-Bal10 B) Sequence coverage of Histone H3-Bal10 after addition of triol **3** C) Transient masking of Bal using triol **3** allows for MS/MS analysis of borylated proteins. D) MS/MS spectrum of peptide fragment TARK-**Bal(–H_2O)**-TGGKAPRKQLATKAAR. c depicts the mutated site S10Bal. E) MS/MS spectrum of peptide fragment K-**Bal**-TGGKAPRKQLATKAAR. c depicts the mutated site S10Bal.

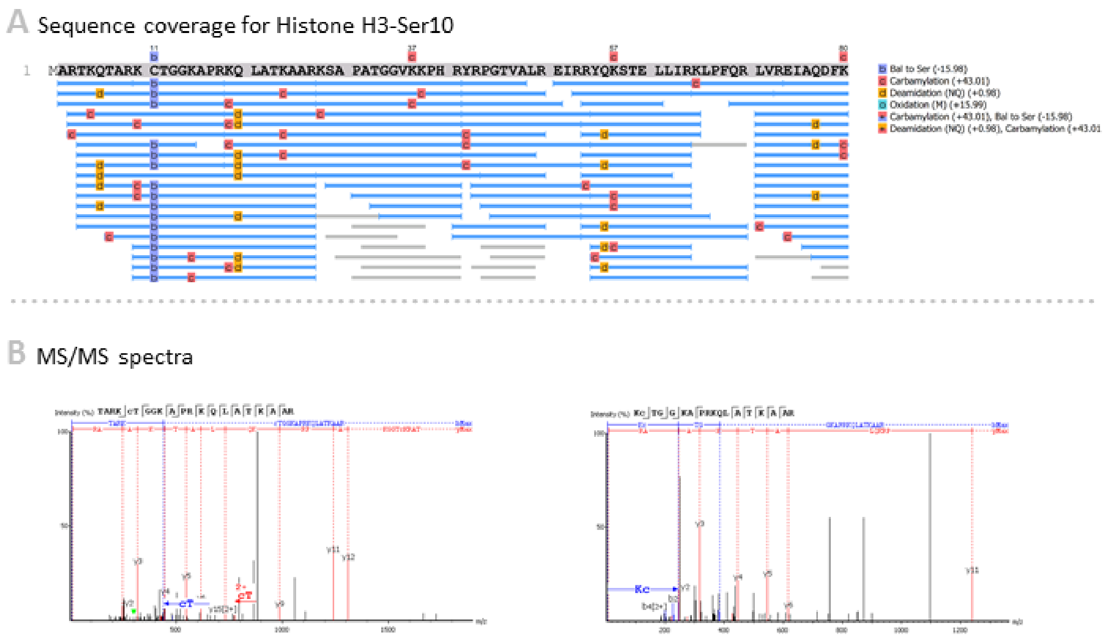

Supplementary Figure 2: MS/MS analysis of Histone H3-Bal10 after oxidation with H_2O_2: A) Sequence coverage of Histone H3-S10 B) MS/MS spectrua of peptide fragments TARK**S**TGGKAPRKQLATKAAR and K**S**TGGKAPRKQLATKAAR. c depicts the mutated site S10.

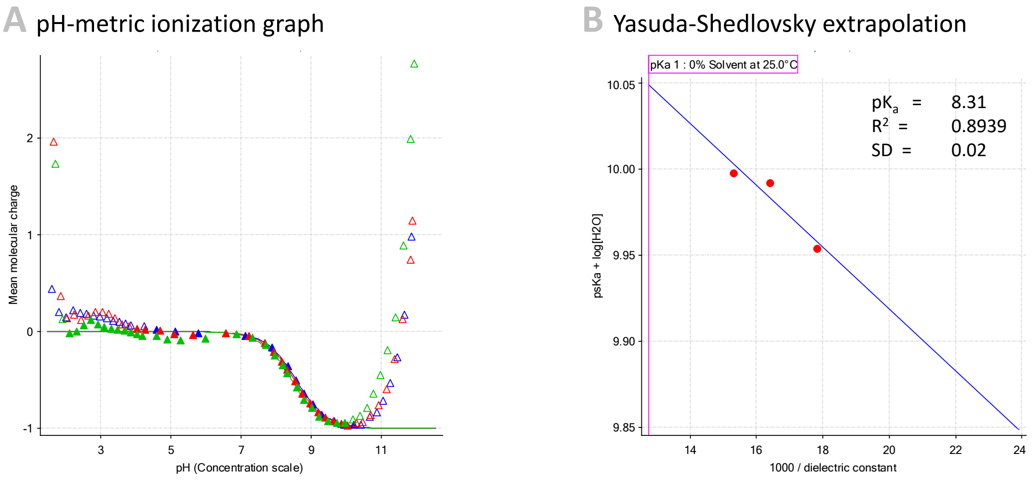

Supplementary Figure 3: Potentiometric pKa determination of model compound Ac-Bal-NHBn (**2**): A) pH-metric pK_a determination at three different organic co-solvent concentrations. B) Yasuda-Shedlovsky extrapolation reveals a pK_a value of 8.31 ± 0.02 (mean±SD) for Ac-Bal-NHBn at 25°C.

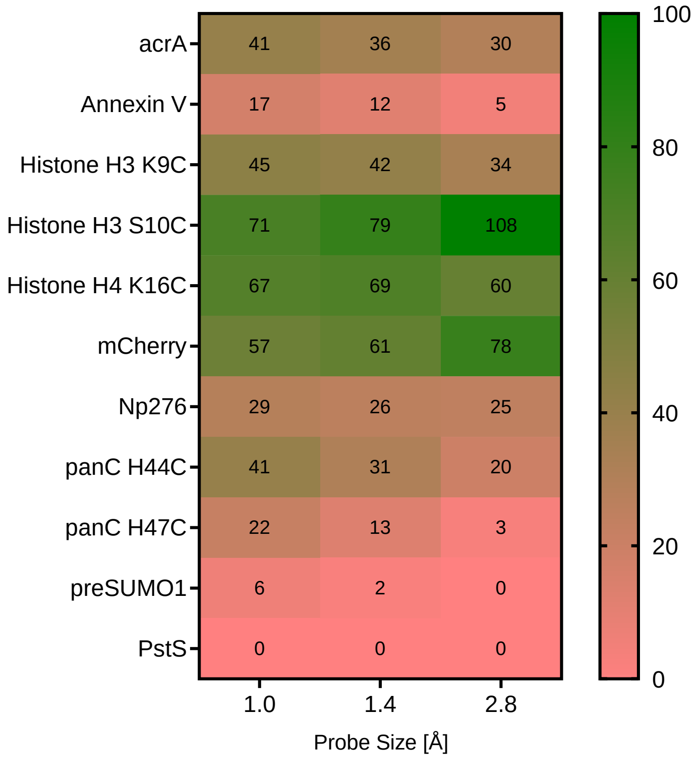

Supplementary Figure 4: Heat map for the solvent-accessible surface area relative to Gly-Cys-Gly for Cys residues in different proteins (in %).

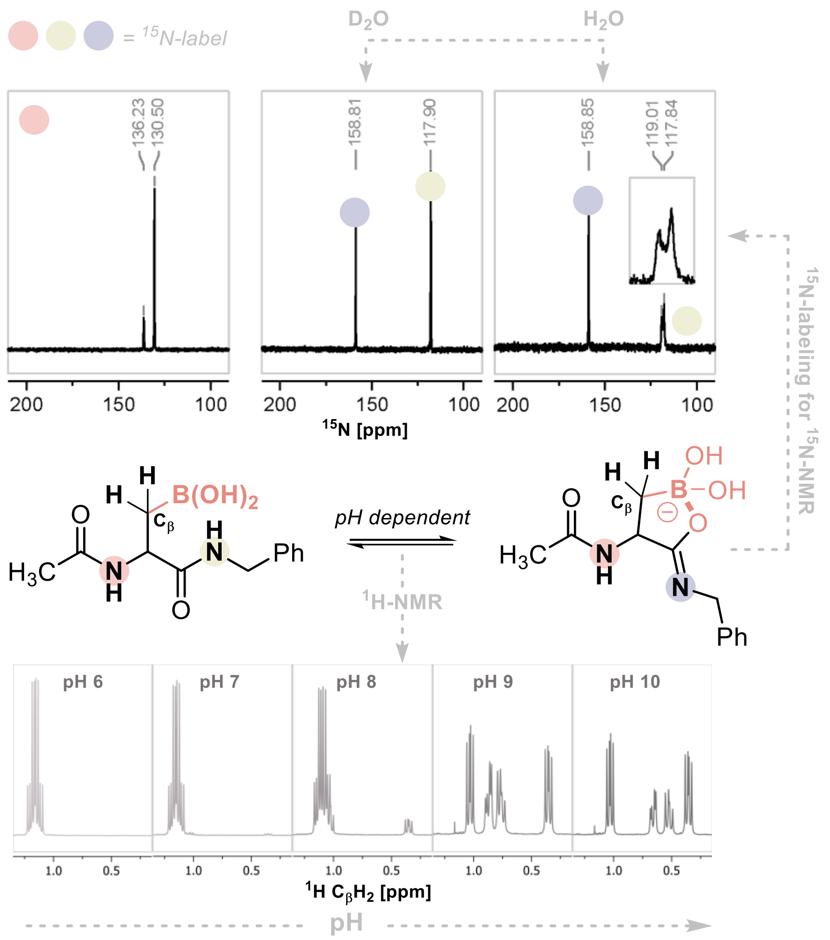

Supplementary Figure 5: NMR analysis of pH dependent oxaborolane formation on small molecule model Ac-Bal-NHBn (**2**) in aqueous solution.

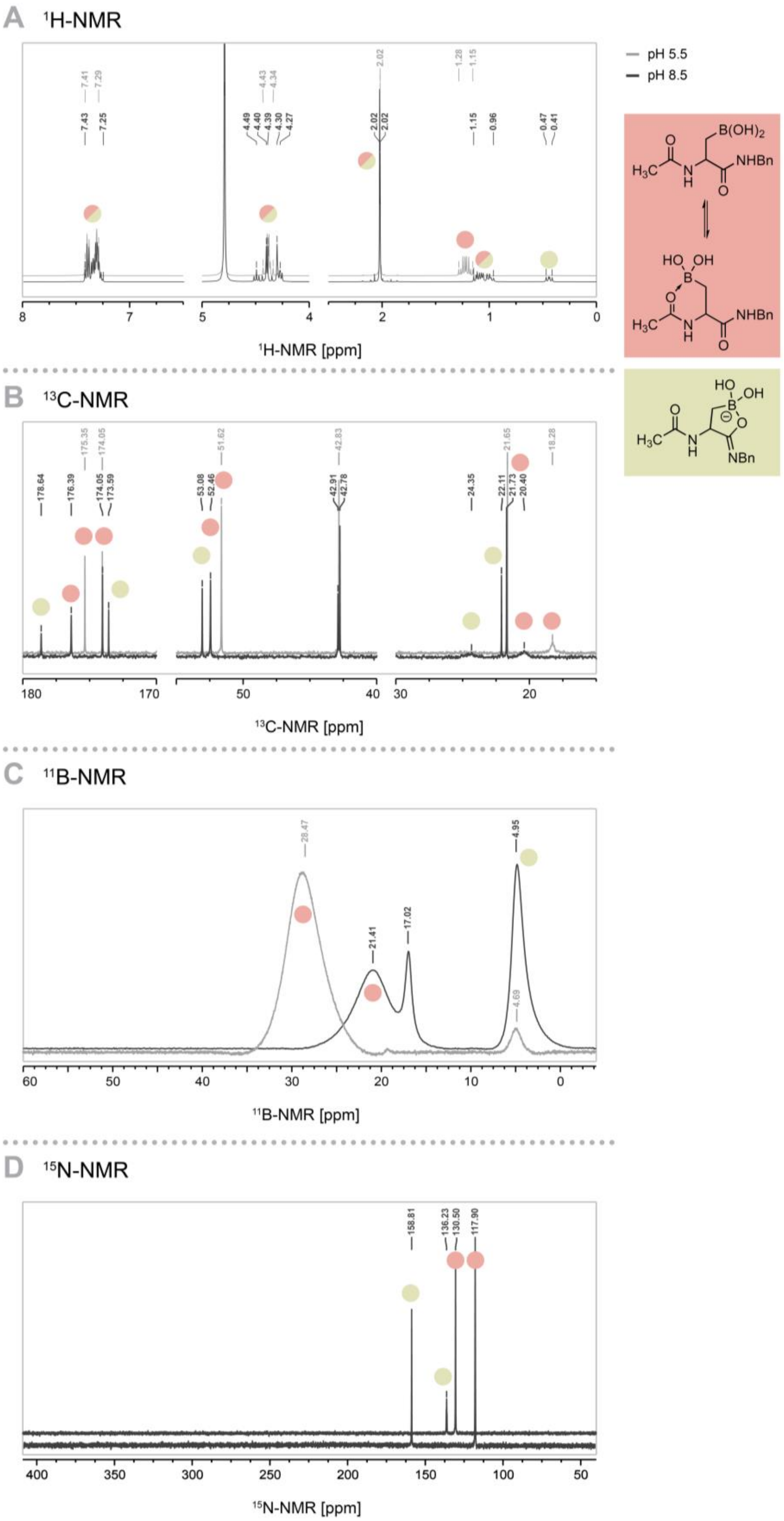

Supplementary Figure 6: Comparison of ^{1}H- (A), ^{13}C- (B), ^{11}B- (C) and ^{15}N- NMR spectra of Ac-Bal-NHBn

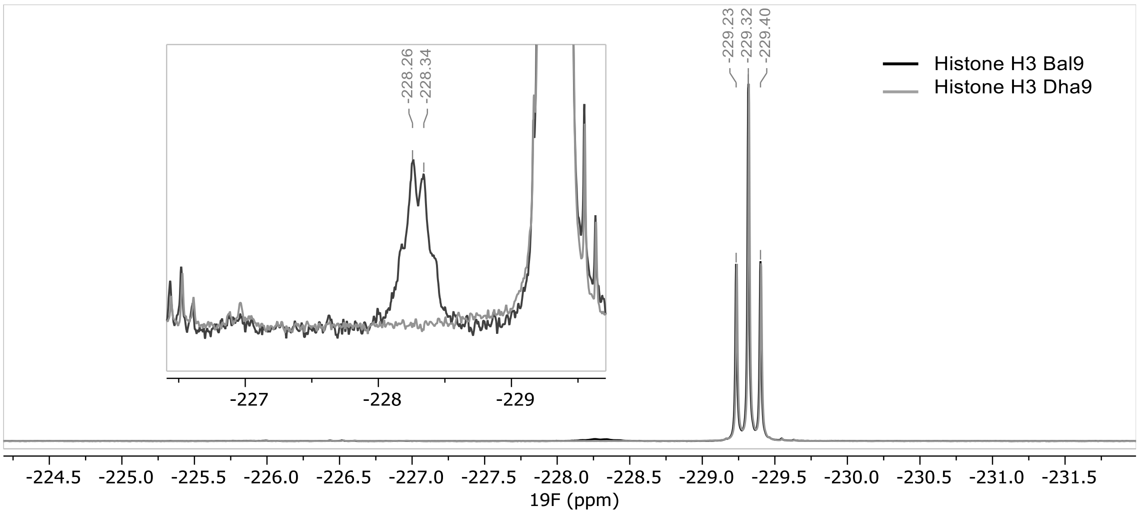

Supplementary Figure 7: ^{19}F-NMR of Histone H3 Bal9 (black) and Histone H3 Dha9 (grey) incubated with 10 equiv. of diol **4**. Histone H3 Bal9 exhibits binding, while no bound species can be detected for Histone H3 Dha9.

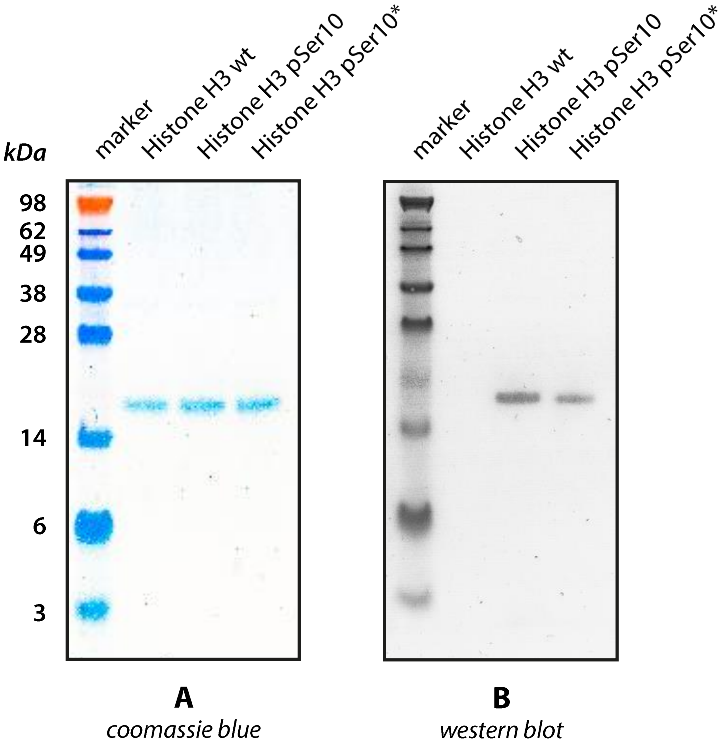

Supplementary Figure 8: Western blot analysis shows that Histone H3-Ser10 derived from Histone H3-Bal10 remains functional in phosphorylation by Aurora B kinase. (**A**) SDS-PAGE analysis and coomassie staining. (**B**) Western blot. Primary antibody: murine anti-Histone H3 pSer10 (dilution 1:1000) (GeneTex, GTX630185); secondary antibody: goat anti-mouse IgG-alkaline phosphatase (dilution 1:1000). Visualization with NBT/BCIP substrate solution (ThermoFisher). Marker = SeeBlue® Plus2 Pre-Stained Protein Standard. * denotes phosphorylated Histone H3 Ser10 derived from Histone H3 Bal10 by oxidation with H_2O_2. Loading: 100 ng. A single experiment was deemed sufficient, as no quantification was required.

A Determination of residual secondary structure in the tail region of histone H3 via secondary chemical shift analysis

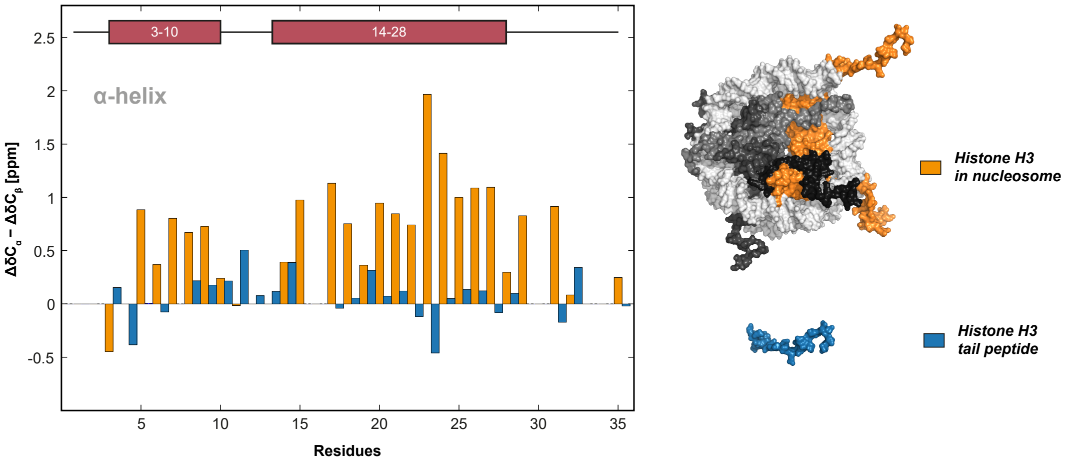

B Comparison of ^{1}H and ^{15}N R_2 relaxation rates for the tail region of histone H3 in the nucleosome and tail peptide

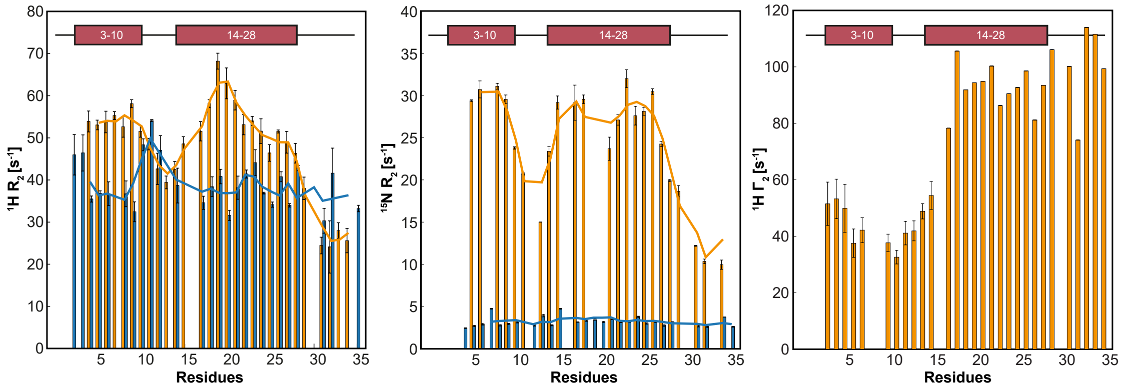

Supplementary Figure 9: NMR analysis reveals two regions (residues 3-10 and residues 14-28) with increased R_2 rates and residual α-helical structure in the histone H3 tail region in a nucleosome context. (**A**) Residual secondary structure determination in the tail region of histone H3 analysing the $\Delta\delta C_\alpha - \Delta\delta C_\beta$ chemical shifts in a nucleosome context (orange) or in the isolated tail peptide (blue) indicates the presence of two regions with residual α-helical structure. (**B**) Comparison of ^{1}H (left) and ^{15}N (middle) R_2 relaxation rates for the tail region of histone H3 in the nucleosome and isolated tail peptide shows two regions with increased R_2 relaxation rates indicating residual structure. Relaxation rates and corresponding uncertainties are derived from fitting intensities from the relevant NMR experiment (see methods) acquired as a function of several various delays, to a single decaying exponential. The most probable relaxation rate and the amplitude of the exponential are obtained from a Levenberg Marquardt assisted least squares optimization, where the uncertainties are derived from the covariance matrix using standard methods. Paramagnetic relaxation enhancement (PRE) (right) using MTSL paramagnetic labeling of the K39C mutant shows a strong PRE effect in the N terminal residues.

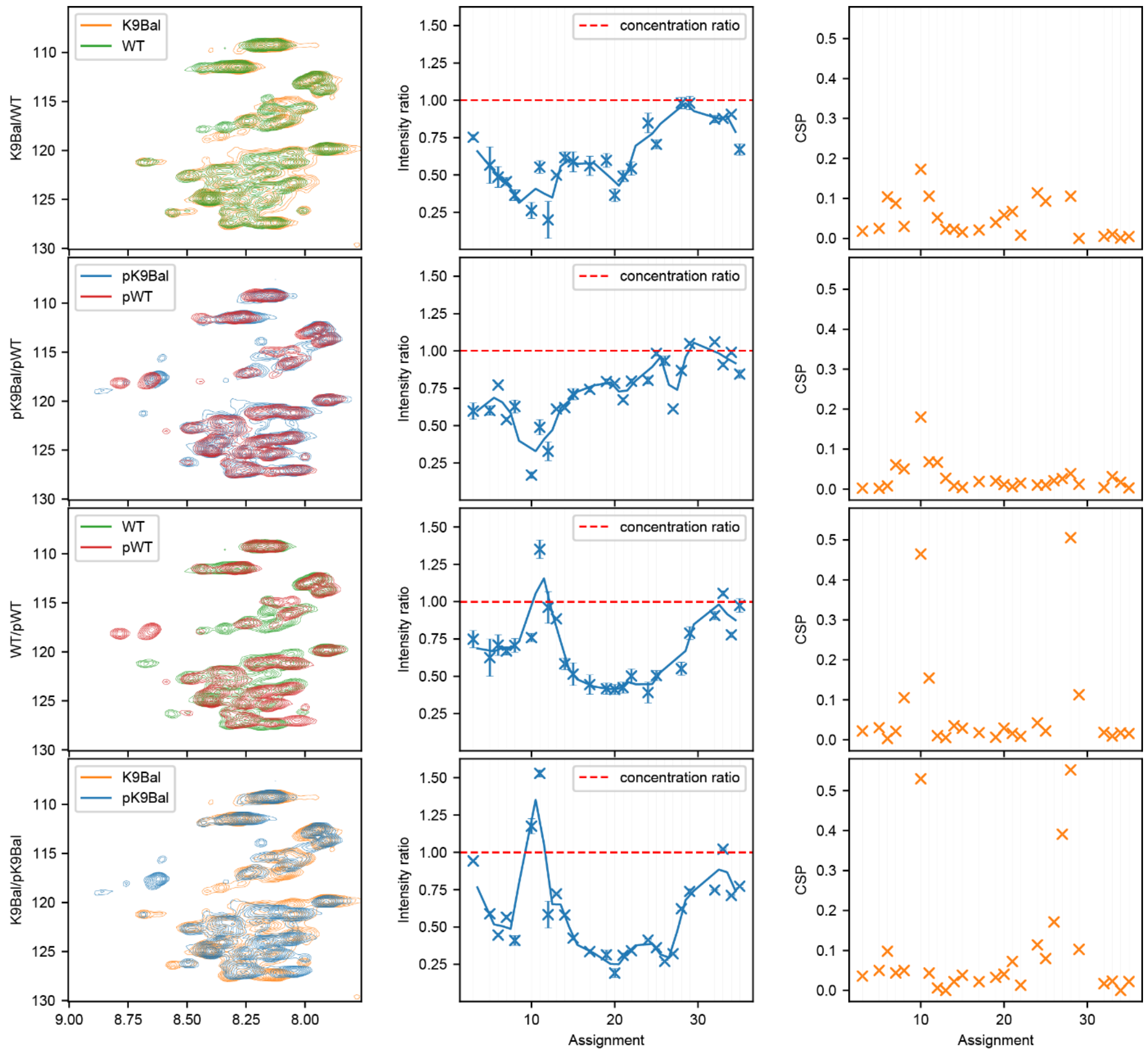

Supplementary Figure 10: Contour plots, intensity ration plots and chemical shift perturbations (CSPs) for Histone H3-Bal9 containing nucleosome vs WT, phosphorylated Histone H3-Bal9 containing nucleosome vs phosphorylated WT, WT nucleosome vs phosphorylated WT and Histone H3-Bal9 containing nucleosome vs phosphorylated Histone H3-Bal9 containing nucleosome. Depicted values represent the mean; error bars show SD from n=3 measurements of the same sample.

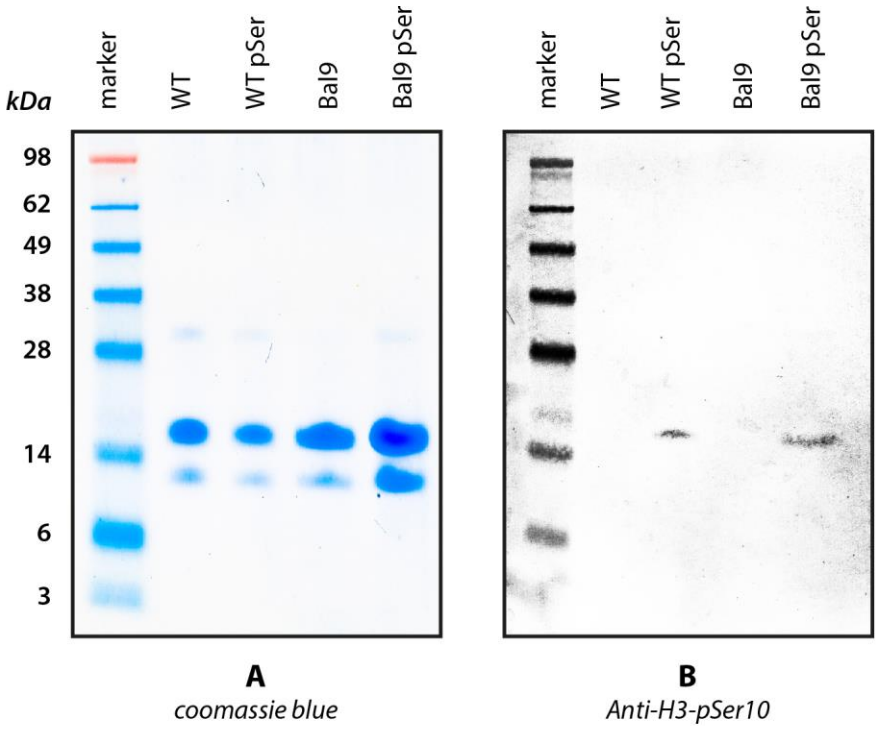

Supplementary Figure 11: SDS-PAGE and Western blot analysis of phosphorylated nucleosomes. 10% bis-TRIS gel, MES buffer, 200 V, 40 min, 4 °C. Primary antibody: murine anti-Histone H3 pSer10 (GeneTex, GTX630185); secondary antibody: goat anti-mouse IgG-alkaline phosphatase. Visualization with NBT/BCIP substrate solution (ThermoFisher). Marker = SeeBlue® Plus2 Pre-Stained Protein Standard, WT = wild-type nucleosome, WT pSer = phosphorylated wild-type nucleosome, Bal9 = Histone H3 Bal9 nucleosome, Bal9 pSer = phosphorylated, Histone H3 Bal9 nucleosome. A single experiment was deemed sufficient, as no quantification was required.

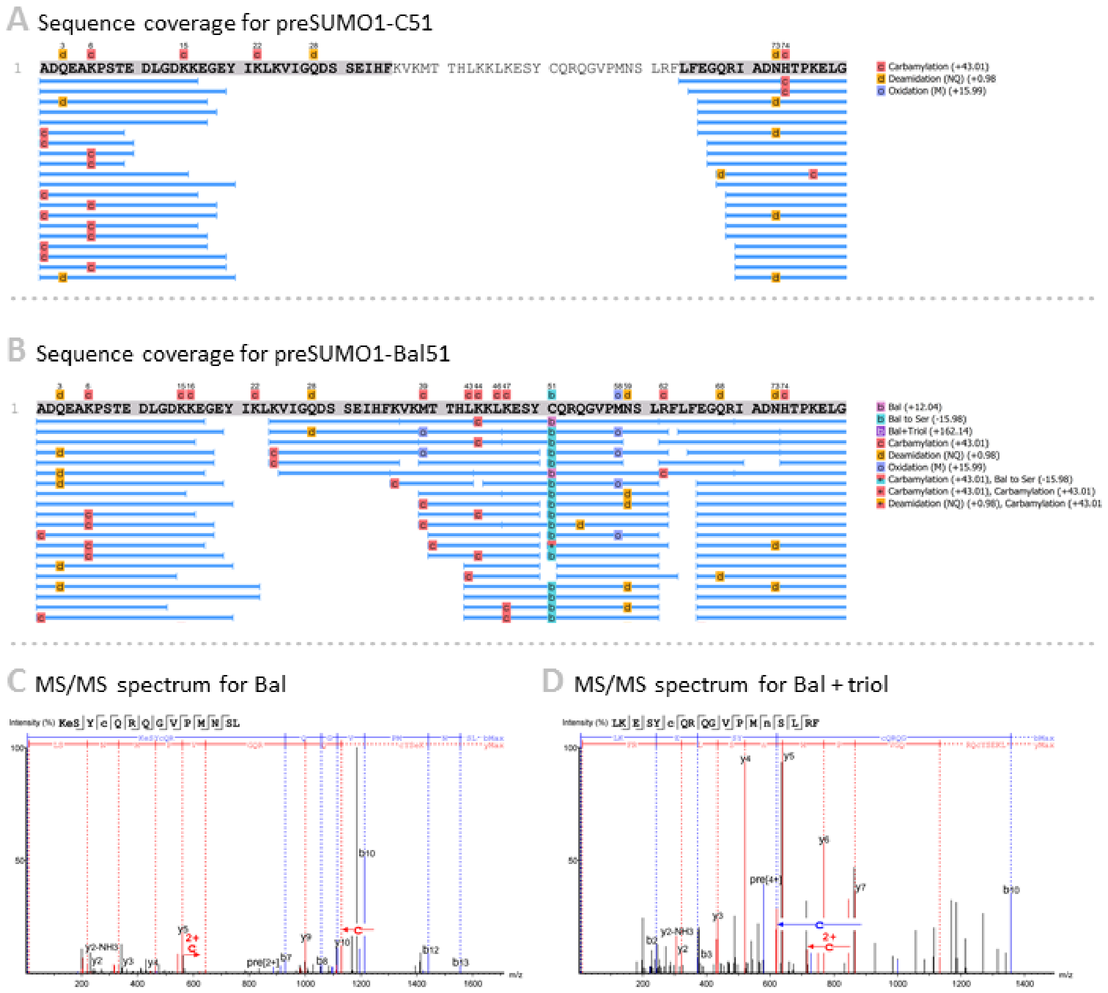

Supplementary Figure 12: MS/MS analysis of preSUMO1 after digestion with chymotrypsin: (**A**) Sequence coverage of preSUMO1-C51 (**B**) Sequence coverage of preSUMO1-Bal51 using transient masking with triol **3** (**C**) MS/MS spectrum of peptide fragment KESY-**Bal**-QRQGVPMNSL. c depicts the mutated site C51Bal. (**D**) MS/MS spectrum of peptide fragment LKESY-**Bal(+triol)**-QRQGVPMNSLRF. c depicts the mutated site C51Bal.

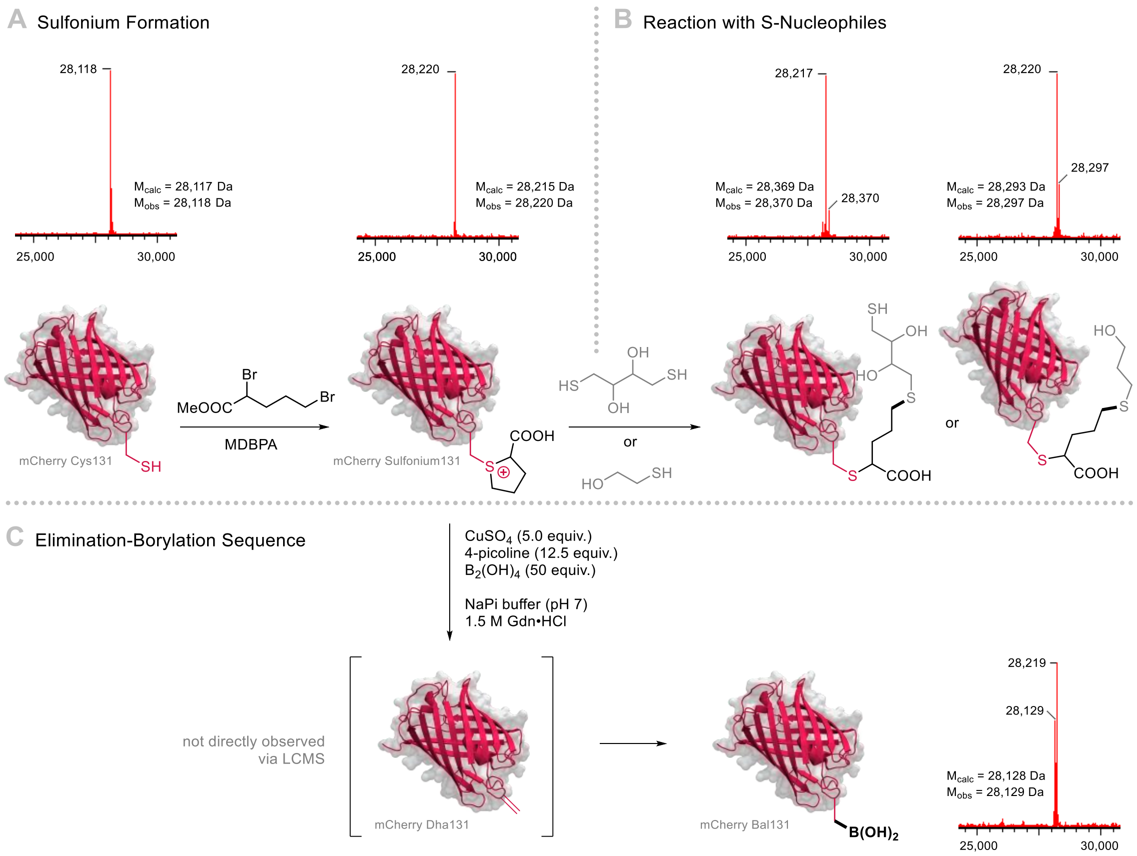

Supplementary Figure 13: Borylation of mCherry. (**A**) Formation of mCherry Sulfonium131 via *bis*-alkylation with MDBPA. (**B**) Confirmation of the sulfonium species via reaction with S-nucleophiles DTT and β-mercaptoethanol. (**C**) One-pot elimination-borylation sequence gives access to mCherry Bal131.

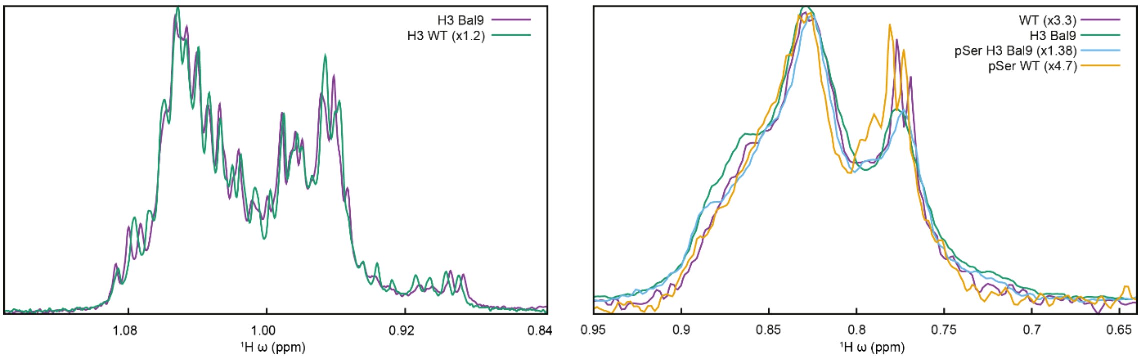

Supplementary Figure 14: Concentration determination via NMR utilizing methyl region.

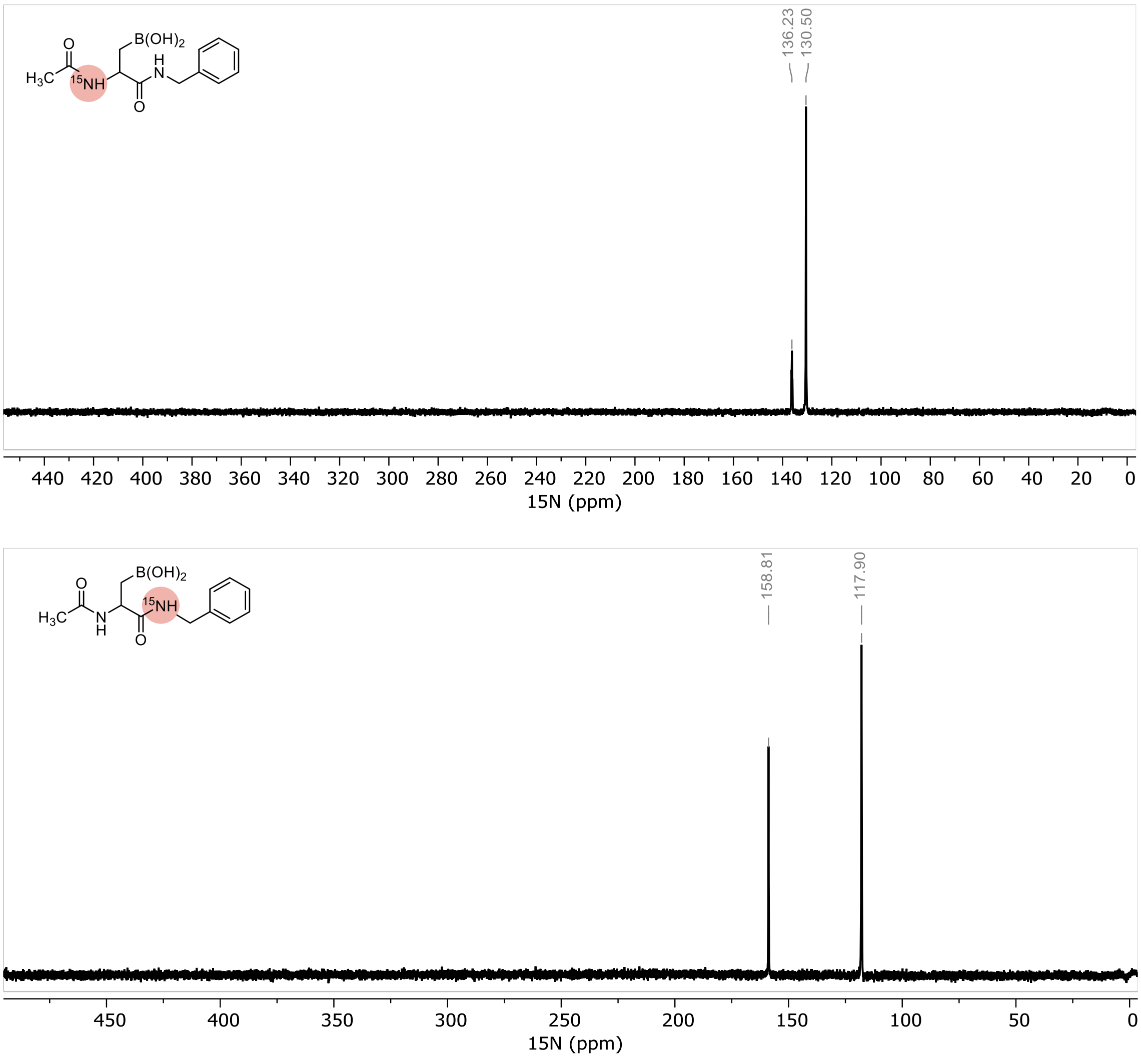

Supplementary Figure 15: ^{15}N-NMR spectra of both ^{15}N-labelled isotopologues of Ac-Bal-NHBn.

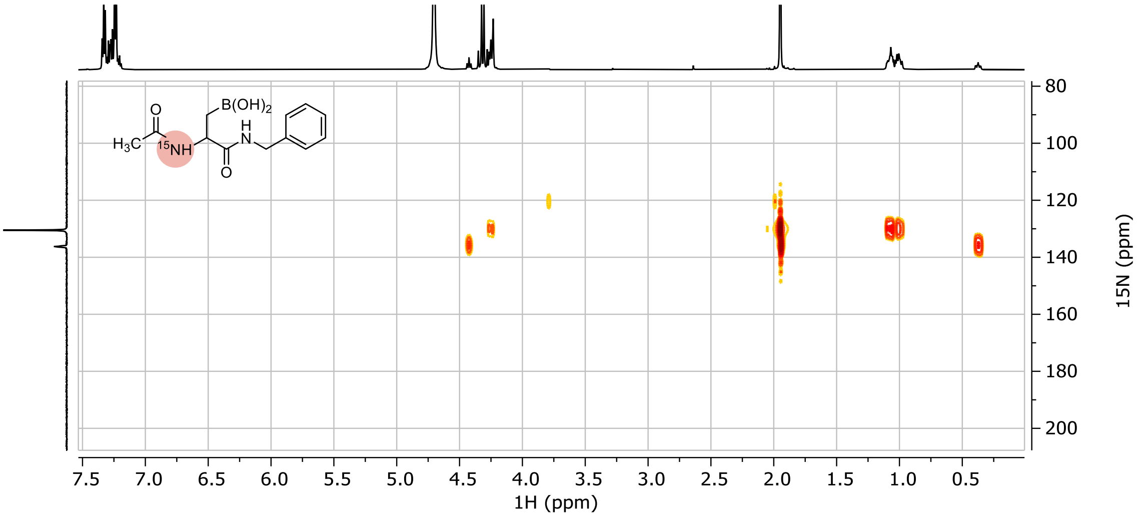

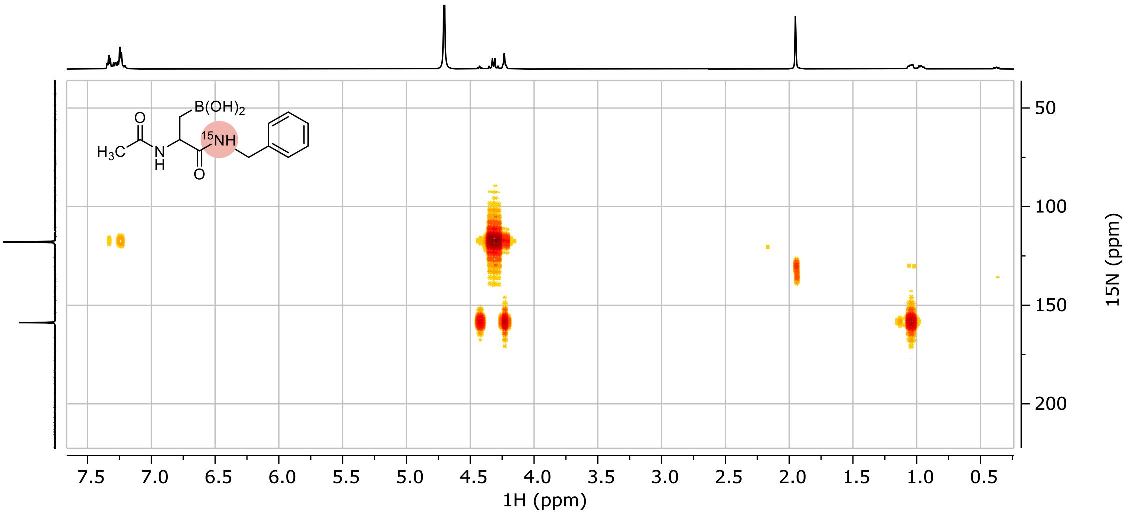

Supplementary Figure 16: ^{1}H-^{15}N-HMBC spectra of both ^{15}N-labelled isotopologues of Ac-Bal-NHBn.

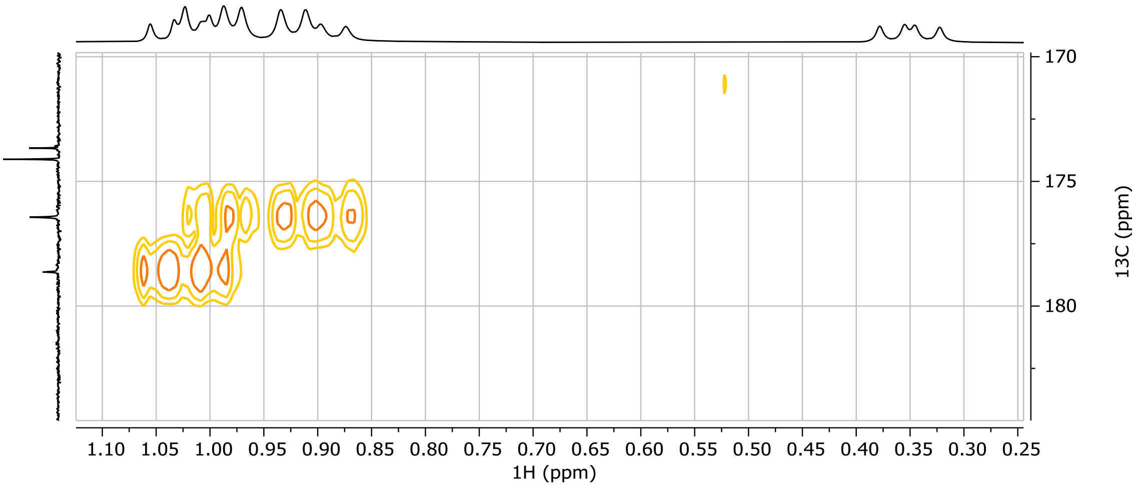

Supplementary Figure 17: ^{1}H-^{13}C-HMBC spectrum of Ac-Bal-NHBn at pH 8.5.

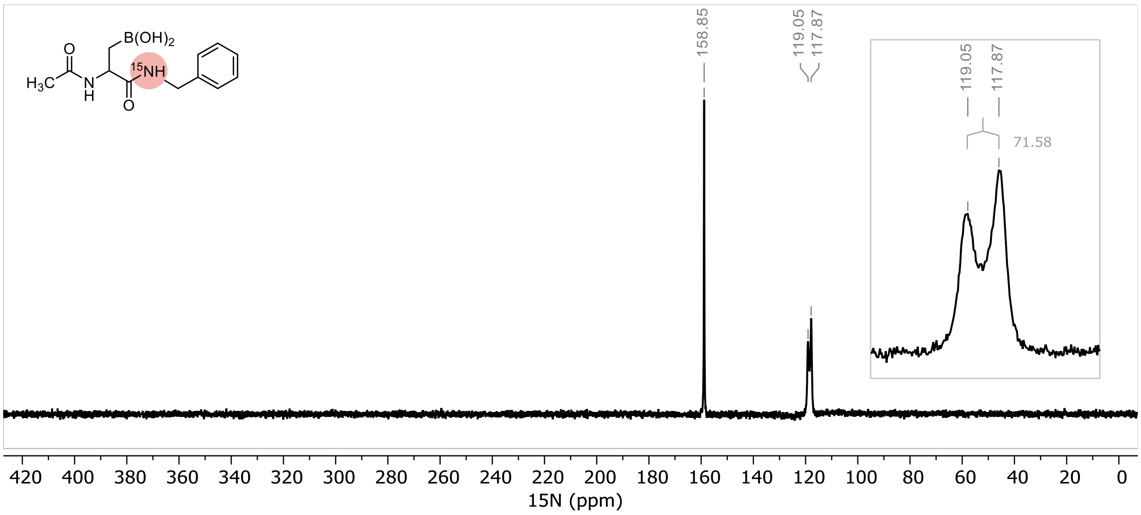

Supplementary Figure 18: ^{15}N-NMR spectrum of C-terminally labelled Ac-Bal-NHBn in H_2O/D_2O (95:5) at pH 8.5.

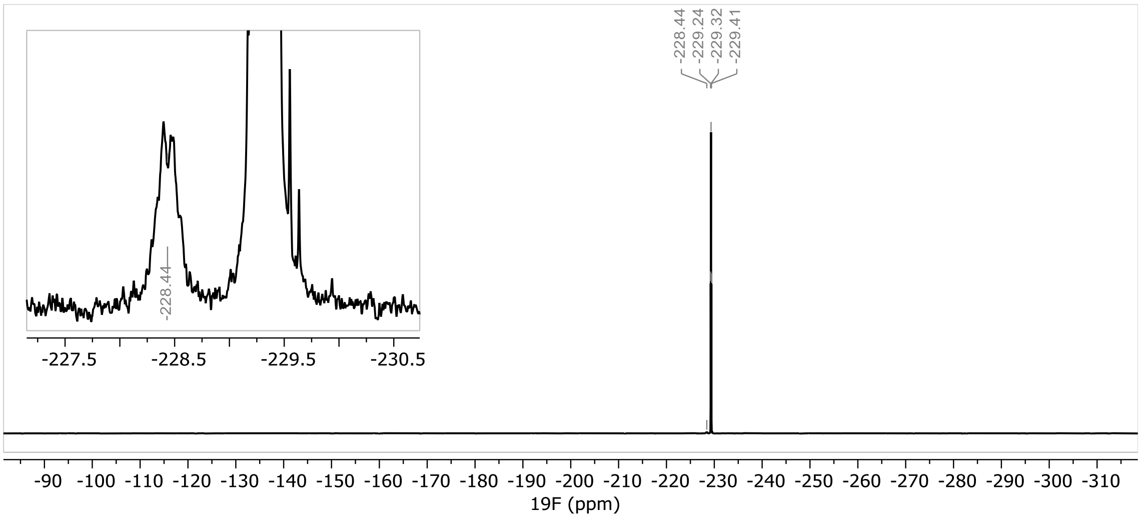

Supplementary Figure 19: Representative ^{19}F-NMR spectrum (d_1 = 2 s, 4096 scans) of diol **4** binding to Histone H3-Bal9. Bound diol leads to a peak at –228.44 ppm.

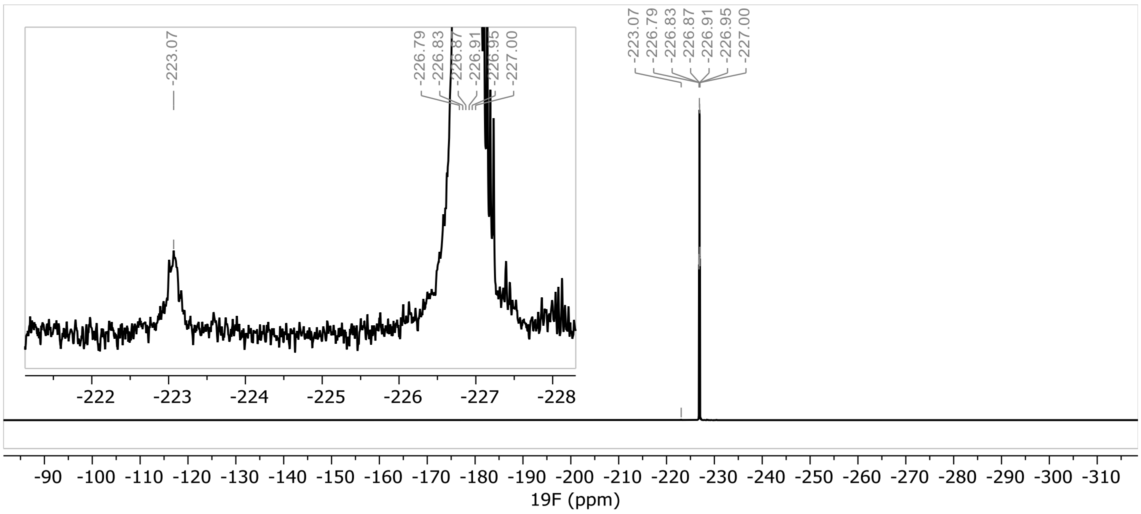

Supplementary Figure 20: Representative ^{19}F-NMR spectrum (d_1 = 2 s, 20480 scans) of FDRibOMe (**5**) binding to Histone H3-Bal9. Bound diol leads to a peak at –223.07 ppm.

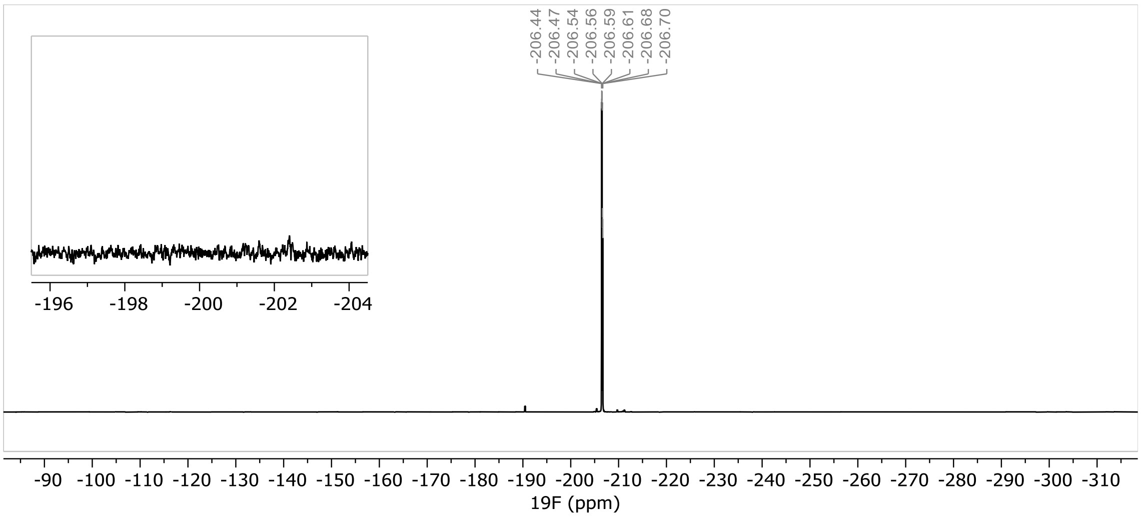

Supplementary Figure 21: Representative ^{19}F-NMR spectrum (d_1 = 2 s, 25395 scans) of attempted binding of FDGal (**6**) to Histone H3-Bal9. No bound diol could be detected.

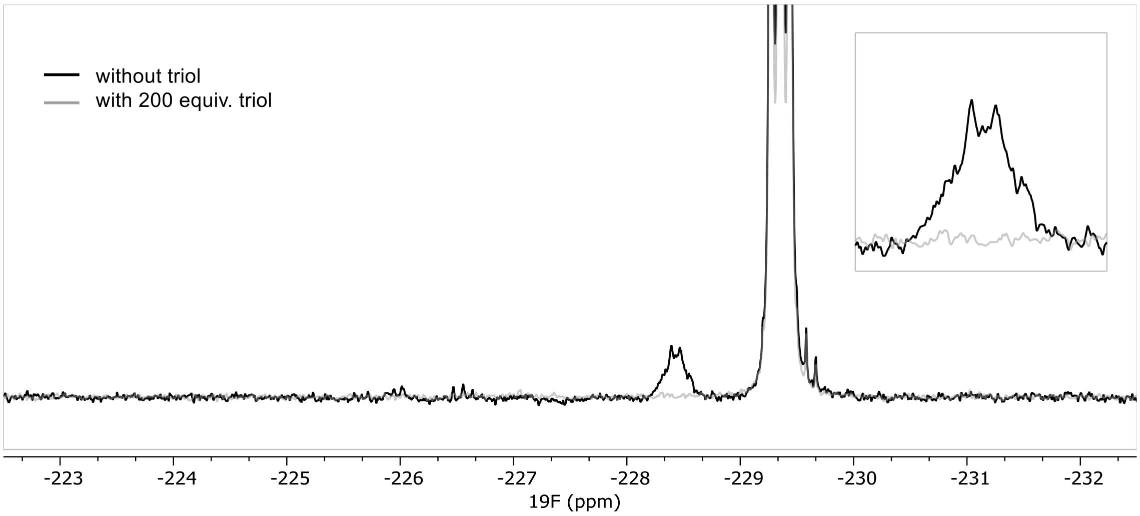

Supplementary Figure 22: ^{19}F-NMR spectra of Histone H3-Bal9 in the presence of diol **4** (black) and after addition of large excess of triol **3** (grey).

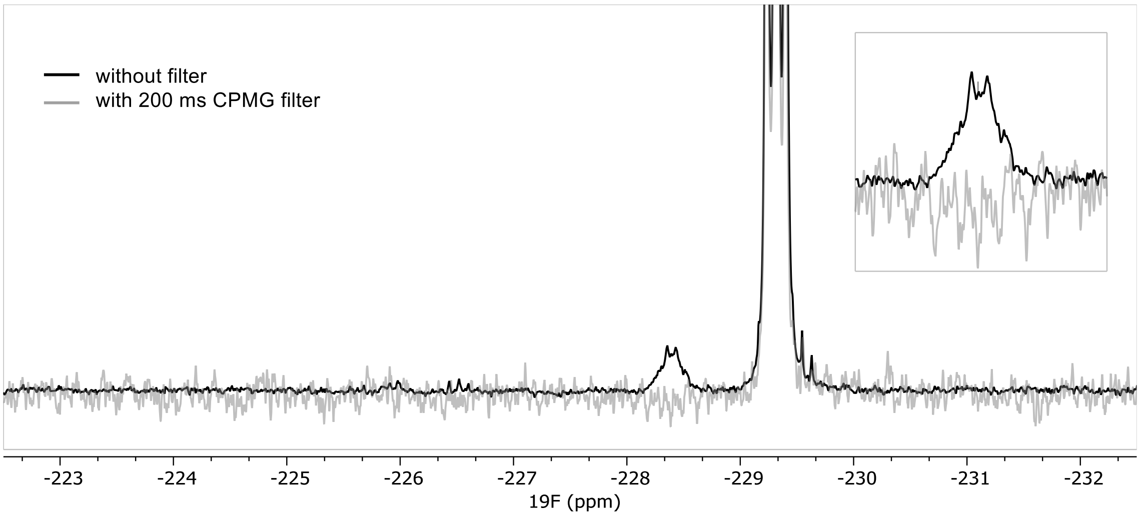

Supplementary Figure 23: ^{19}F-NMR spectra of Histone H3-Bal9 in the presence of diol **4** using a standard pulse sequence (black) and a 200 ms CPMG filter (grey). Peak intensities were normalized based on unbound diol.

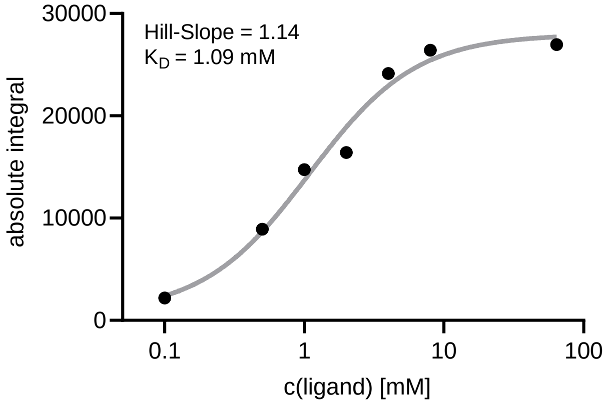

Supplementary Figure 24: Dose-response curve obtained for ^{19}F-NMR titration of Histone H3 Bal9 with fluorinated diol **4**.

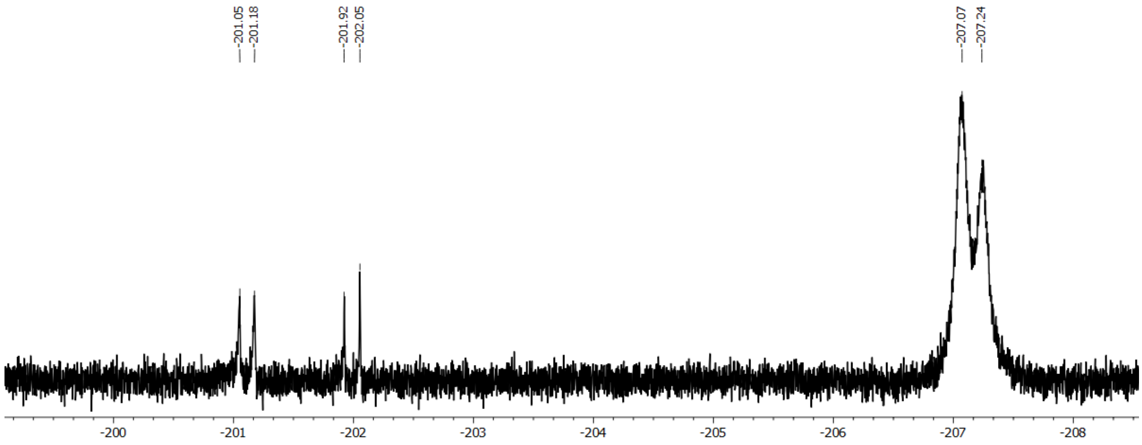

Supplementary Figure 25: $^{19}F-\{^{1}H\}$-NMR spectrum of 2-deoxy-2-fluoro-D-galactose (FDGal) (**6**) binding to borylated model substrate (Ac-Bal-NHBn, **2**). Binding of both, α- and β-anomers, to R and S enantiomers of the model substrate is observed; giving rise to four distinct peaks for bound FDGal.

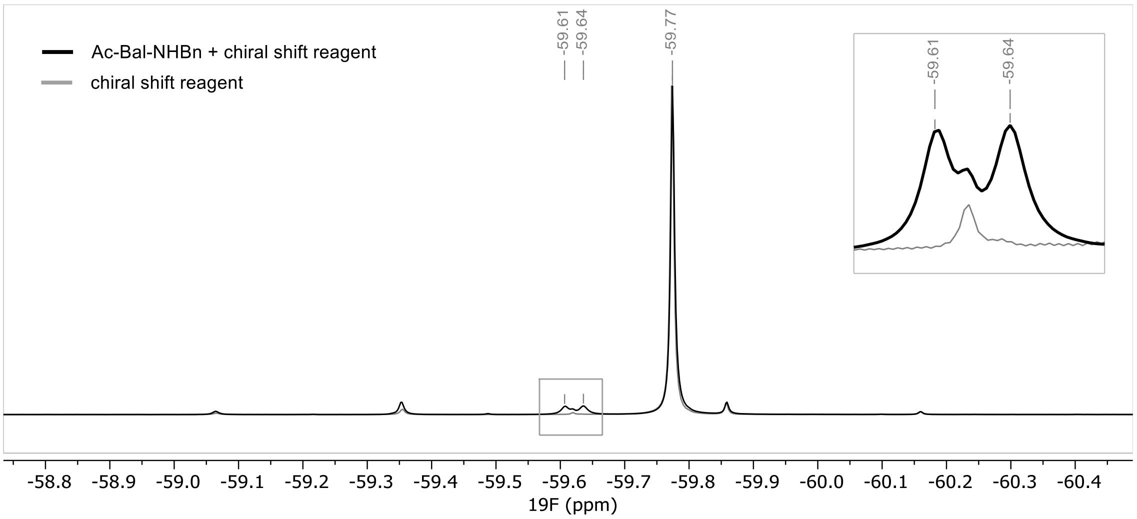

Supplementary Figure 26: ^{19}F-NMR determination of the enantiomeric ratio of Ac-Bal-NHBn trifluoromethoxy-modified diol as a chiral shift revealed a racemic mixture.

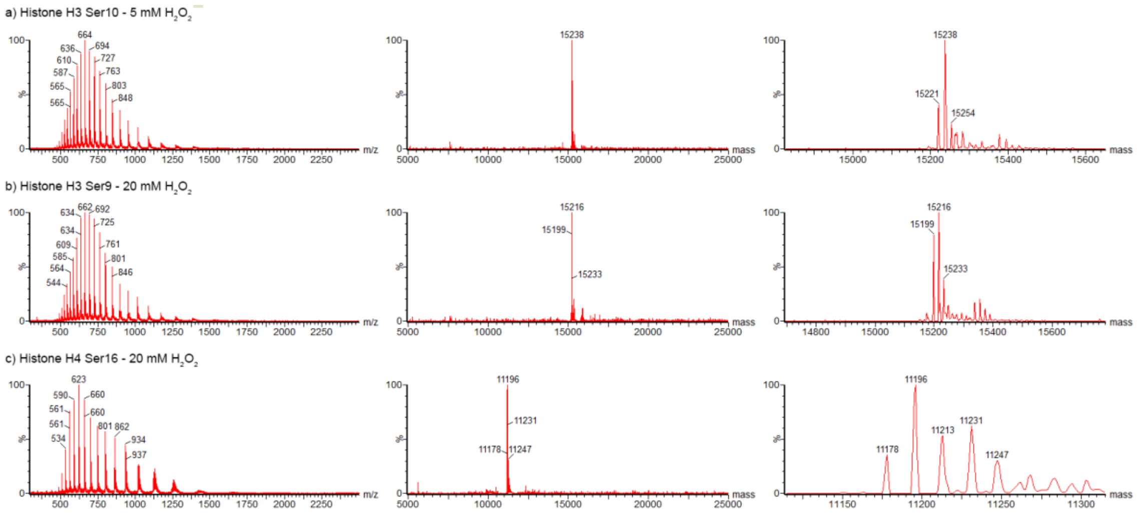

Supplementary Figure 27: Ion series and deconvoluted spectra for oxidation of borylated mutants to Histone H3 Ser10 (M_{calc} = 15,239 Da; M_{obs} = 15,238 Da; 15,254 Da (mono-oxidation)) (**a**), Histone H3 Ser9 (M_{calc} = 15,198 Da; M_{obs} = 15,199 Da; 15,216 Da (mono-oxidation); 15,233 Da (di-oxidation)) (**b**) and Histone H4 Ser16 (M_{calc} = 11,195 Da; M_{obs} = 11,196 Da; 11,213 Da (mono-oxidation); 11,213 Da (di-oxidation); 11,231 Da (tri-oxidation)) (**c**).

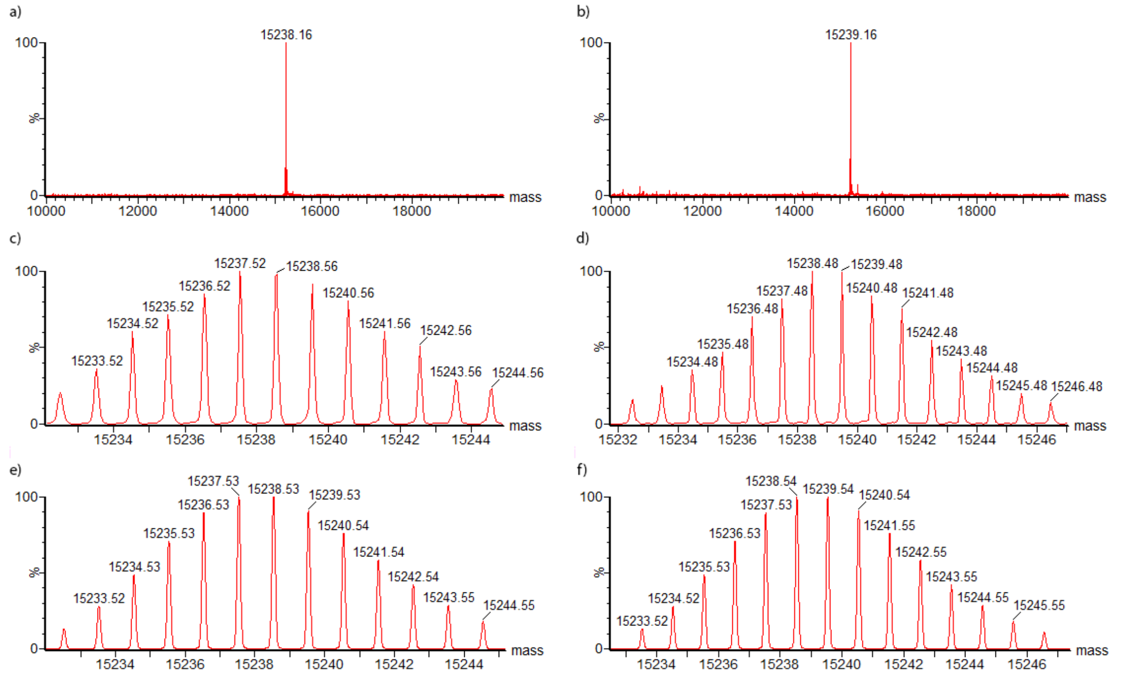

Supplementary Figure 28: Isotopic distribution pattern of Histone H3-Ser10 and Histone H3-dSer10: a) deconvoluted mass spectrum of Histone H3-Ser10 b) deconvoluted mass spectrum of Histone H3-dSer10 c) measured isotopic pattern of Histone H3-Ser10 d) measured isotopic pattern of Histone-dSer10 e) calculated isotopic pattern for Histone H3-Ser10 f) calculated isotopic pattern for Histone H3-dSer10. Measured patterns were obtained by high resolution Qtof mass spectrometry and deconvolution using the MaxEnt1 algorithm (mass resolution: 0.04 Da/channel, uniform gaussian, half height: 0.04 Da, minimum intensity ratios: 33% left and right) preinstalled on MassLynx 4.1 (Waters). Calculated patterns were obtained using the isotope modelling feature (Create Continuum spectrum, peak width at half-height: 0.10, separation: 1.00, min abundance (%): 1.00, full range) preinstalled on MassLynx 4.1 (Waters) using the molecular formula $C_{670}H_{1131}N_{215}O_{186}S_2$ for H3-Ser10 and $C_{670}H_{1130}N_{215}O_{186}S_2D$ for H3-dSer10. The atomic mass of D was defined as 2.014102 Da.

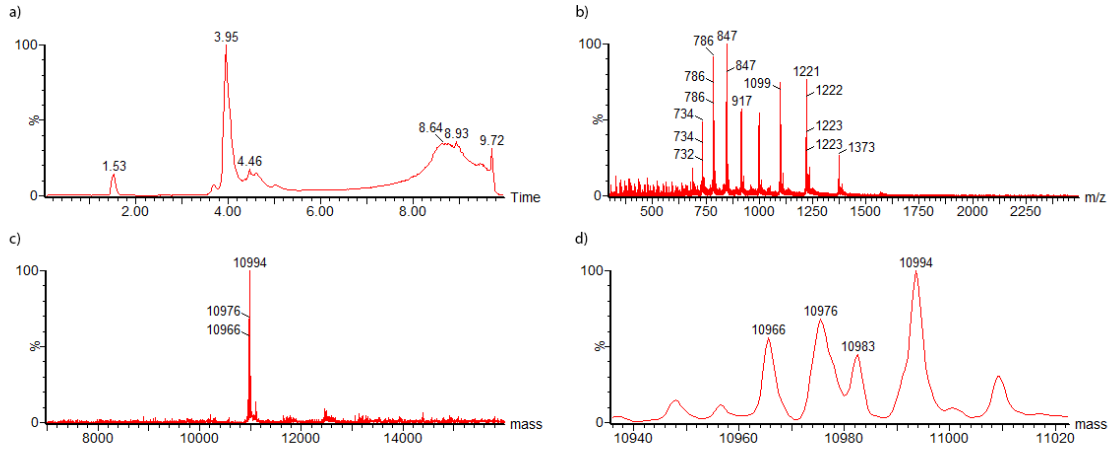

Supplementary Figure 29: LCMS analysis of matured SUMO1-Bal51: a) total ion chromatogram b) ion series c) deconvoluted spectrum d) magnification of the major peak**;** calculated masses: 10997 g/mol (Bal), 10979 g/mol (Bal−H_2O), 10961 g/mol (Bal−2H_2O); observed masses: 10997 g/mol (Bal), 10976 g/mol (Bal−H_2O), 10966 g/mol (Bal−2H_2O).

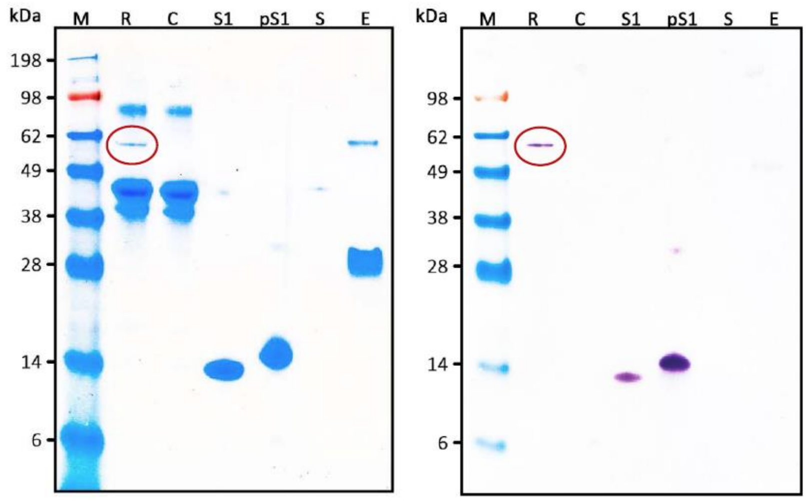

Supplementary Figure 30: Coomassie-stained gel and Western Blot of in-vitro SUMOylation using SUMO1-Bal51. M = marker, R = reaction, C = control, S1 = SUMO1-Bal51, pS1 = pre-SUMO1-Bal51, S = substrate (RanGAP1 fragment), E = SENP1. A single experiment was deemed sufficient, as no quantification was required.

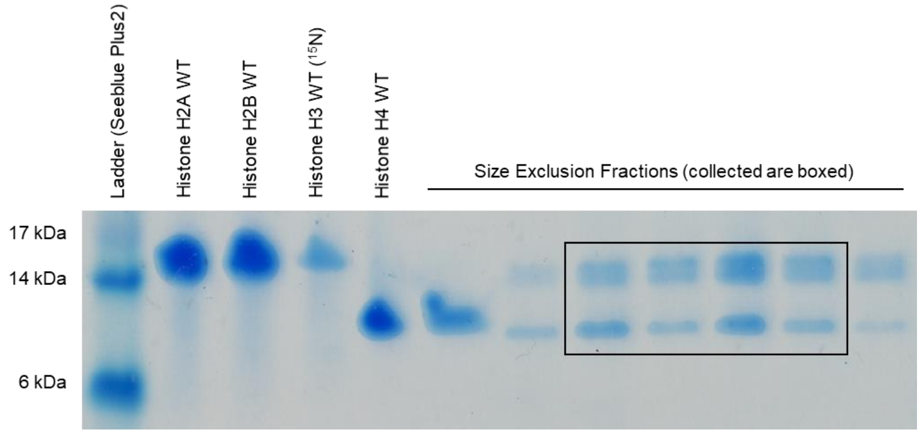

Supplementary Figure 31: Analysis of octamer containing ^{15}N-labelled Histone H3 WT (from oxidation of [^{15}N]Histone H3 Bal10) via SDS-PAGE (10% Bis-TRIS gel, MES buffer, 200 V, 40 min) and coomassie staining (marker = SeeBlue™ Plus2 Pre-stained Protein Standard). A single experiment was deemed sufficient, as no quantification was required.

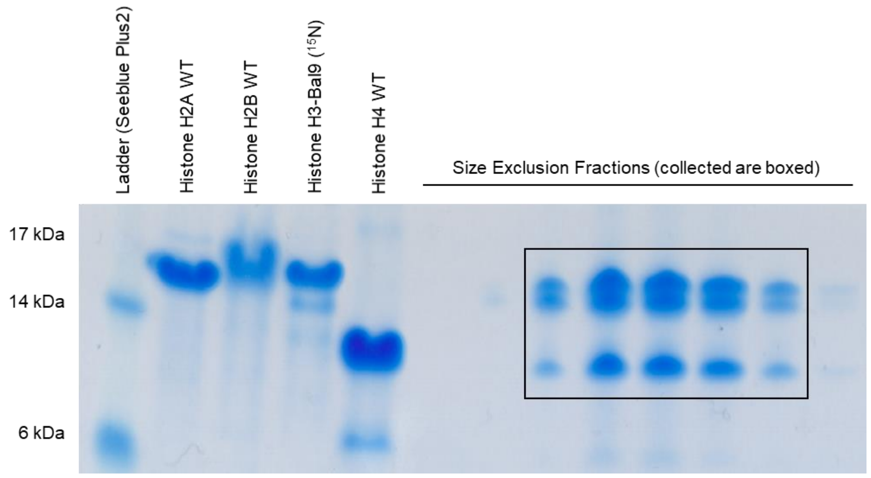

Supplementary Figure 32: Analysis of octamer containing ^{15}N-labelled Histone H3 Bal9 via SDS-PAGE (10% Bis-TRIS gel, MES buffer, 200 V, 40 min) and coomassie staining (marker = SeeBlue™ Plus2 Pre-stained Protein Standard). A single experiment was deemed sufficient, as no quantification was required.

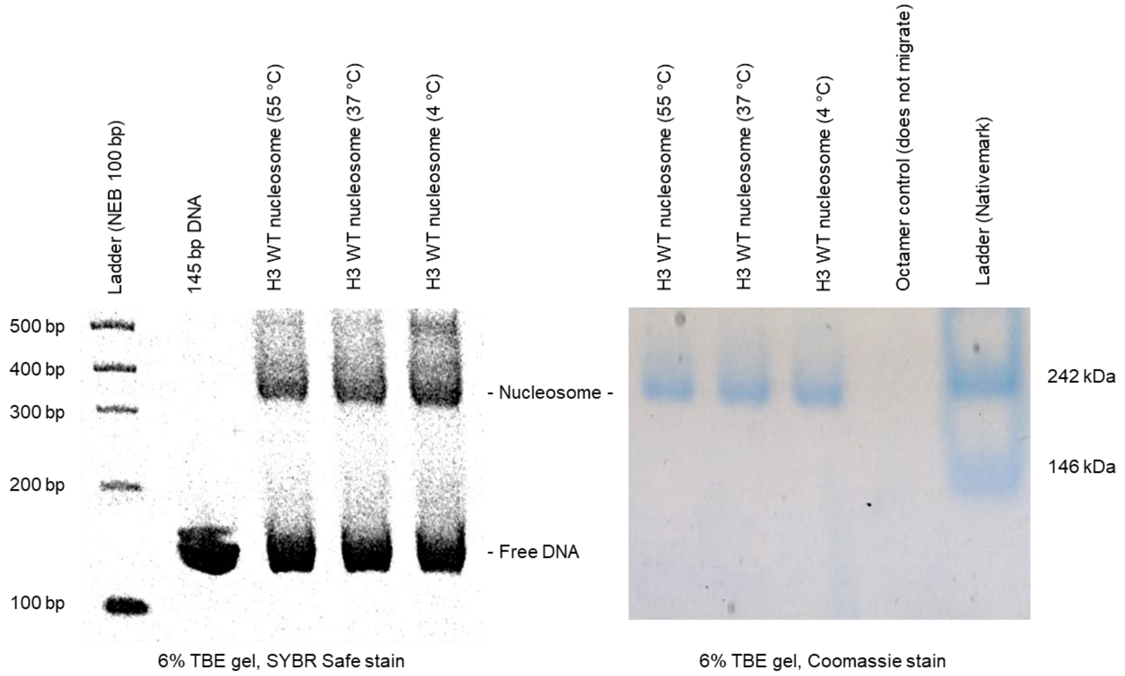

Supplementary Figure 33: Analysis of Nucleosome containing ^{15}N-labelled Histone H3 WT. A single experiment was deemed sufficient, as no quantification was required.

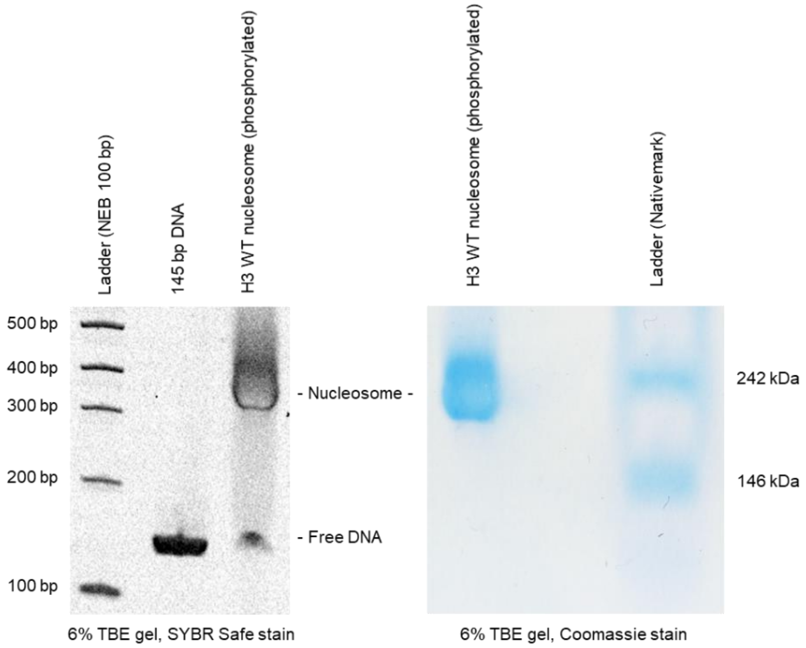

Supplementary Figure 34: Analysis of Nucleosome containing ^{15}N-labelled Histone H3 WT after phosphorylation. A single experiment was deemed sufficient, as no quantification was required.

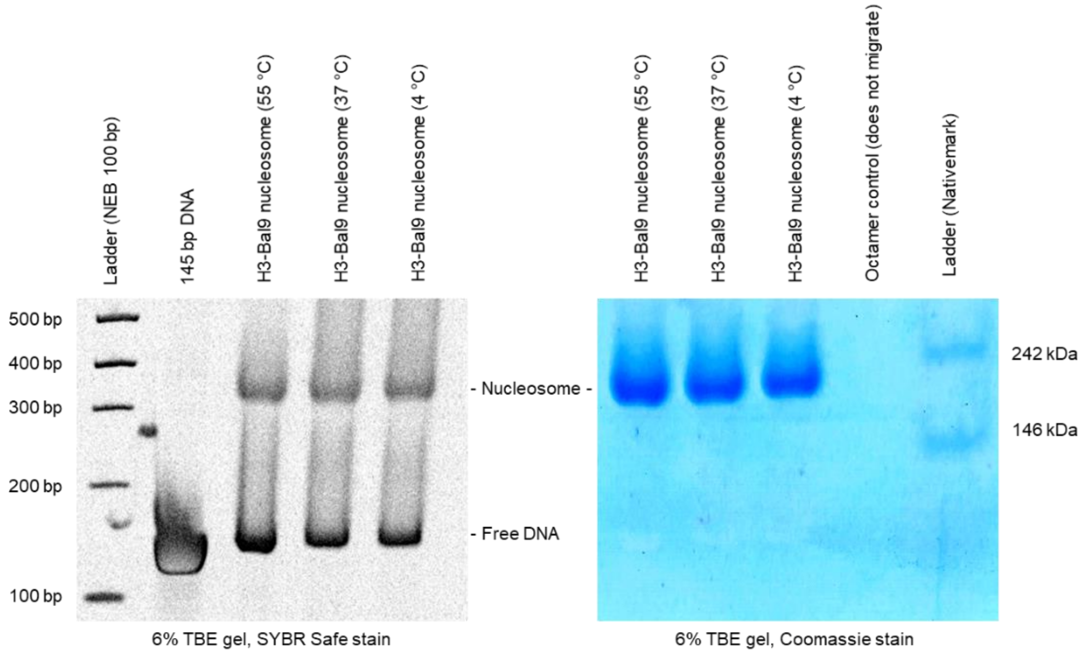

Supplementary Figure 35: Analysis of Nucleosome containing ^{15}N-labelled Histone H3 Bal9. A single experiment was deemed sufficient, as no quantification was required.

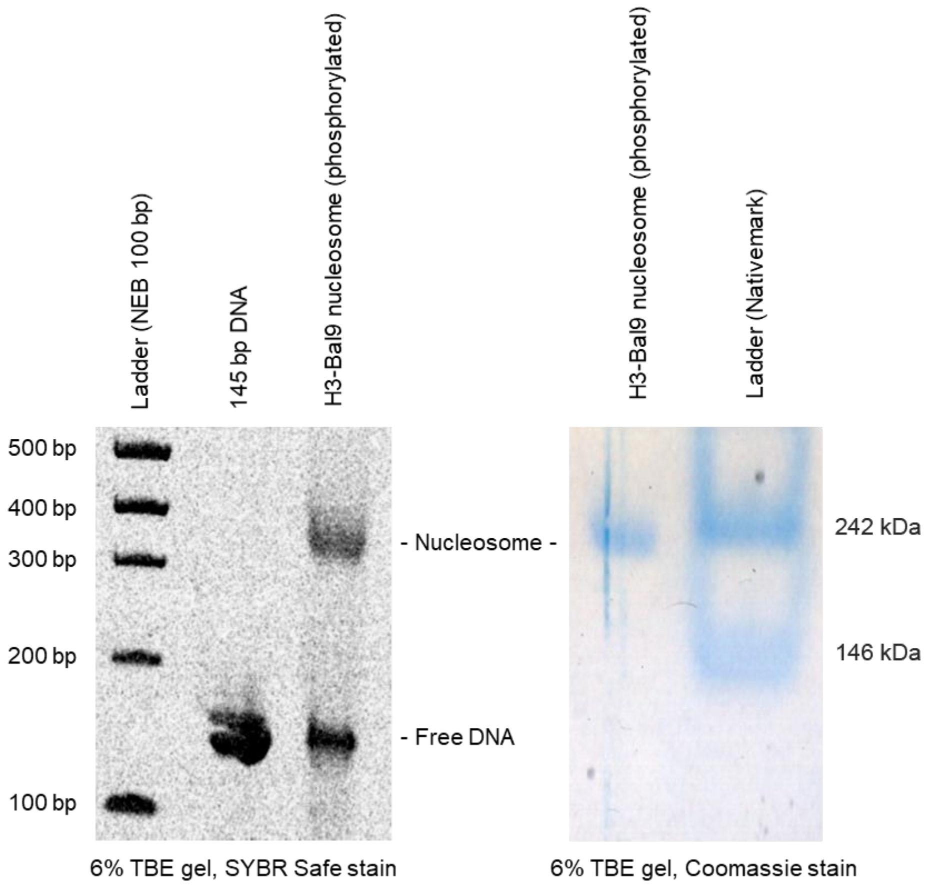

Supplementary Figure 36: Analysis of Nucleosome containing ^{15}N-labelled Histone H3 Bal9 after phosphorylation. A single experiment was deemed sufficient, as no quantification was required.

2 Supplementary Tables

Supplementary Table 1: Optimization of Bal formation on peptidic model substrate **1**.

Entry	Variable[#]	NMR-yield
Influence of ligand / base (using $CuSO_4$ as copper source)		
**1**	pyridine	96%
**2**	4-picoline	99%
**3**	2,2'-bipyridine	6%
**4**	DMAP	25%
**5**	proton sponge	11%
**6**	urea	3%
**7**	imidazole	88%
**8**	Gdn·HCl	n.d.
**9**	Gdn·HCl, Na_2HPO_4 (1.0 equiv.)	18%
**10**	Na_2HPO_4 (1.0 equiv.)	5%
**11**	none	2%
Using amino acids as ligands (using $CuSO_4$ as copper source)		
**12**	L-histidine	66%
**13**	L-tryptophan	4%
**14**	L-proline	0%
**15**	glycine	3%
Influence of the copper source (using 4-picoline as ligand)		
**16**	$CuSO_4$	99%
**17**	$Cu(NO_3)_2$	95%
**18**	$Cu(OAc)_2$	99%
**19**	$Cu_2(OH)_2CO_3$	59%
**20**	$Cu(OTf)_2$	99%
**21**	none	0%

[#]If not specified otherwise, standard conditions are used. Standard conditions: 2-acetylamino-*N*-benzyl-acrylamide (S4) (10.0 mg, 45.8 µmol, 1.00 equiv.) was dissolved in D_2O (1.0 mL). Copper source (4.58 µmol Cu(II), 0.10 equiv.) and base (11.5 µmol, 0.25 equiv.) were added and the mixture was stirred at room temperature for 3 hours.

Supplementary Table 2: Optimization of Bal formation on model protein Histone H3-Dha10.

Entry	Variable[#]	Yield
Influence of base (equiv.)		
**1**	2,2'-bipyridine (12.5)	72%
**2**	DMAP (12.5)	62%
**3**	proton sponge (12.5)	0%
**4**	4-picoline (12.5)	92%
**5**	4-picoline (50.0)	87%
**6**	none	82%
Influence of temperature		
**7**	room temperature	92%
**8**	0°C	79%
**9**	37°C	85%
Influence of boron source		
**10**	10 equiv. $B_2(OH)_4$	16%
**11**	50 equiv. $B_2(OH)_4$	92%
**12**	250 equiv. $B_2(OH)_4$	87%
**13**	4 × 15 equiv. $B_2(OH)_4$	85%
Influence of stoichiometry		
**14**	1.0 equiv. $CuSO_4$	86%
**15**	5.0 equiv. $CuSO_4$, 12.5 equiv. 4-picoline	92%
**16**	5.0 equiv. $CuSO_4$, 50 equiv. 4-picoline	87%
**17**	50 equiv. $CuSO_4$, 50 equiv. 4-picoline	62%
Influence of addition sequence		
**18**	$B_2(OH)_4$ → $CuSO_4$ → 4-picoline	85%
**19**	4-picoline → $B_2(OH)_4$ → $CuSO_4$	87%
**20**	4-picoline → $CuSO_4$ → $B_2(OH)_4$	92%
Influence of urea as denaturant		
**21**	1 M Urea	75%
**22**	3 M Urea	82%
**23**	5 M Urea	84%
**24**	7 M Urea	89%
Use of common buffer systems		
**25**	50 mM NH_4OAc, 3 M Gdn·HCl, pH 7.0	91%

**26**	50 mM TRIS base, 3 M Gdn·HCl, pH 7.0	92%
**27**	50 mM HEPES (sodium salt), 3 M Gdn·HCl, pH 7.0	87%

#If not specified otherwise, standard conditions are used. Standard conditions: Histone H3-Dha10 in NaP_i buffer (100 mM, pH 7.0) at 1 mg/mL, 5 equiv. $CuSO_4$, 12.5 equiv. 4-picoline, 50 equiv. $B_2(OH)_4$, 40 µL reaction volume. Reactions were conducted at room temperature and conversions were determined after 30 min.

Supplementary Table 3: Optimization of Bal formation on model protein Histone H3-Dha10: Dependency of conversions from pH and denaturant concentration.

conditions / pH	6.0	6.5	7.0	7.5	8.0	8.5	9.0	9.5
**A (0 M)**	38	69	67	69	67	65	56	51
**B (3 M)**	74	90	92	88	90	88	80	64
**C (5 M)**	57	91	89	88	87	87	81	79

Numbers in % conversion. Conditions: Histone H3-Dha10 in NaP_i buffer (100 mM) at specified pH and 1 mg/mL, 5 equiv. $CuSO_4$, 12.5 equiv. 4-picoline, 50 equiv. $B_2(OH)_4$, 40 µL reaction volume. Reactions were conducted at room temperature and conversions were determined after 30 min. A: no addition of Gdn·HCl; B: 3 M Gdn·HCl; C: 5 M Gdn·HCl.

Supplementary Table 4: Protein secondary structure content estimated from CD spectra for Cys and Bal mutants and comparison to theoretical secondary structure content derived from crystal structures.

**Entry**	**Protein**	**α-helix [%]**			**β-sheet [%]**		
		Cys	*Bal*	*calc*	*Cys*	*Bal*	*calc*
**1**	Annexin V site316	**65.3±2.9**	**48.5±3.3**	72.1±4.9	2.6±1.9	9.0±4.3	1.5±1.9
**2**	Histone H3 site10	66.9±24.9	65.7±26.7	52.1±5.0	7.8±7.3	8.3±7.8	2.4±4.1
**3**	Npβ site61	13.5±5.6	9.9±5.0	5.4±0.5	26.0±2.9	23.4±4.9	20.8±31.1
**4**	preSUMO1 site51	17.2±12.4	9.5±3.9	16.0±6.3	21.3±6.8	19.1±5.0	31.7±5.4
**5**	PstS site197	77.4±8.9	76.2±5.3	41.5±4.3	6.7±4.1	5.3±3.0	24±6.3

Protein secondary structures were estimated from CD spectra using K2D3[1], CDSSTR[2–4] and BeStSel[5]. Reference values were determined from the corresponding crystal structures deposited on PDB using the algorithms DSSP_CONT, P-SEA, PALSSE, STICKS, STRIDE and XTLSSTR implemented in 2Struc.[6] The single values obtained from the different algorithms were averaged and the standard deviation was calculated.

Supplementary Table 5: Change in protein secondary structure content upon borylation as determined via CD spectroscopy for selected borylated proteins.

Entry	Protein	Δ α-helix [%]	Δ β-sheet [%]
**1**	Annexin V site316	−15.6 ± 0.9	6.4 ± 2.4
**2**	Histone H3 site10	−1.2 ± 1.8	0.5 ± 0.5
**3**	Npβ site61	−3.7 ± 3.8	−2.6 ± 2.7
**4**	preSUMO1 site51	−7.7 ± 12.4	−2.2 ± 7.4
**5**	PstS site197	−1.2 ± 3.8	−1.4 ± 1.1

Protein secondary structures were estimated from CD spectra using K2D3[1], CDSSTR[2–4] and BeStSel[5]. Changes in secondary structure were calculated as Δα-helix = $\alpha\text{-helix}_{Bal}$ – $\alpha\text{-helix}_{Cys}$ and Δβ-sheet = $\beta\text{-sheet}_{Bal}$ – $\beta\text{-sheet}_{Cys}$. The single values obtained from the different algorithms were averaged and the standard deviation was calculated.

Supplementary Table 6: Distribution of Bal-substitution amongst a range of different borylated proteins reveals high protein specificity.

Protein	Distribution of Bal-substitution[#] [%]		
	un-substituted	*mono-substituted*	*di-substituted*
**acrA Bal127***	0	100	0
**Annexin V Bal316**	0	56±3	44±3
**Histone H3 Bal9**	80±4	12±3	8±4
**Histone H3 Bal10**	40±5	19±1	41±6
**Histone H4 Bal16**	48±4	52±4	0
**mCherry Bal131**	100±0	0	0
**Npβ Bal61**	21±1	50±2	29±2
**panC Bal44***	0	100	0
**preSUMO1 Bal51**	35±4	54±6	11±3
**PstS Bal197**	6±5	79±7	15±11

[#] Bal-substitution distribution was determined via LCMS. Unless stated otherwise, samples were measured in triplicate. * n = 1.

Supplementary Table 7: Observed and calculated ^{15}N-, ^{1}H-, ^{13}C- and ^{11}B-NMR shifts for possible species of Ac-Bal-NHBn.

No.	Species#	^{15}N-NMR [ppm] N^1	^{15}N-NMR [ppm] N^2	^{1}H-NMR [ppm] CαH	^{1}H-NMR [ppm] CβH$_2$	^{13}C-NMR [ppm] Cα	^{13}C-NMR [ppm] Cβ	^{11}B-NMR [ppm]	CSP (major)	CSP (minor)
**1**	*observed (major)*	*130.5*	*117.9*	*4.28*	*0.94-1.18*	*52.6*	*20.4*	*21.4*		
**2**	*observed (minor)*	*136.2*	*158.8*	*4.49*	*0.44*<br>*0.94-1.18*	*53.2*	*24.5*	*4.9*		
**3**	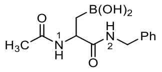	**123**	**109**	**3.30**	**0.54**<br>**2.51**	**55.3**	**19.1**	**26.3**	**1.31**	5.61
**4**	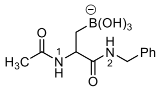	128	104	3.79	0.27<br>0.46	61.7	23.3	2.2	2.57	5.61
**5**	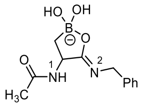	**136**	**167**	**3.92**	**0.34**<br>**1.00**	**57.1**	**26.2**	**8.3**	5.16	**0.99**
**6**	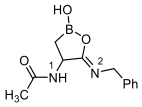	122	233	3.39	1.26<br>1.89	55.7	15.7	32.2	11.61	8.09
**7**	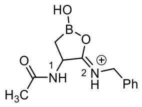	116	228	3.53	0.92<br>1.97	59.1	12.3	31.3	11.20	7.80
**8**	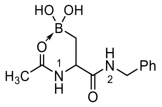	**132**	**114**	**3.74**	**0.63**<br>**1.21**	**61.4**	**15.9**	**9.9**	**1.57**	4.68
**9**	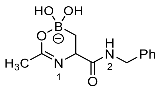	202	113	3.68	−0.34<br>0.95	62.6	22.7	2.1	7.49	8.08
**10**	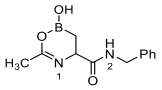	228	120	4.14	0.61<br>1.74	57.7	10.1	28.7	9.85	10.36
**11**	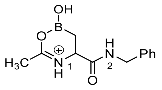	83	138	3.56	0.91<br>0.98	60.4	17.0	11.4	5.32	5.84

#Isotropic chemical shifts were calculated using the Gaussian09.[7] Three dimensional models were created and their geometry was first optimised, before calculation of the shielding tensor from which the isotropic chemical shift can be calculated. All DFT calculations were carried out using the B3LYP density functional with the 6-31G(d) basis set, a combination known to give values in excellent agreement with experimental data.[8,9] Data for observed species was collected on ^{15}N-labelled Ac-Bal-NHBn in D_2O (20 mM NaP_i, pH 8.5). CSP was calculated as $CSP = \sqrt{\sum_{i,n}(\omega_A - \omega_B)^2 \times \frac{\gamma_H^2}{10^2 * \gamma_n^2}}$ with calculated chemical shift ω_A, observed chemical shift ω_B, $\gamma_H = 267 \times 10^6$ rad $T^{-1}s^{-1}$, $\gamma_C = 67.28 \times 10^6$ rad $T^{-1}s^{-1}$, $\gamma_N = -27.12 \times 10^6$ rad $T^{-1}s^{-1}$ and $\gamma_B = 85.84 \times 10^6$ rad $T^{-1}s^{-1}$.

Supplementary Table 8: Solvent accessible surface area calculated for the mutation site in different proteins.

Protein	Site	PDB ID	solvent-accessible surface area [Å^2]			rel. solvent-accessible surface area [%]		
			1.0 Å	1.4 Å	2.8 Å	1.0 Å	1.4 Å	2.8 Å
acrA	C127	2FM1	68.45	60.18	50.03	41	36	30
Annexin V	C316	1HVD	28.09	19.56	8.05	17	12	5
Histone H3	C9	1KX5	74.88	70.47	56.28	45	42	34
Histone H3	C10	1KX5	118.13	131.56	179.69	71	79	108
Histone H4	C16	1KX5	112.36	115.57	100.63	67	69	60
mCherry	C131	4ZIN	95.41	102.49	129.64	57	61	78
Npβ	C61	2J8K	47.71	44.15	42.00	29	26	25
panC	C44	1N2E	68.73	52.23	33.42	41	31	20
panC	C47	1N2E	36.45	21.19	4.61	22	13	3
preSUMO1	C51	1A5R	9.26	3.79	0.00	6	2	0
PstS	C197	1A40	0.81	0.00	0.00	0	0	0

Supplementary Table 9: Reactive PLABP: Concentration of H_2O_2 required for oxidation of Bal to Ser correlates with rASA at the site of interest.

Entry	Protein	rASA# [%]	c(H_2O_2) (full conversion to Ser)*
**1**	Histone H3 Bal10	108	5 mM
**3**	Histone H4 Bal16	60	20 mM
**2**	Histone H3 Bal9	34	20 mM
**4**	Npβ Bal61	25	decomposition at 20 mM
**5**	PstS Bal197	0	no oxidation at 20 mM

#solvent-accessible surface area of the Cys mutant relative to Gly-Cys-Gly calculated with a probe size of 2.8 Å. *Reaction in NaPi (100 mM, pH 7.0, 3 M Gdn·HCl) for 10 min.; H_2O_2 concentrations tested: 1 mM, 5 mM, 20 mM. Full conversion to Ser is considered achieved once no masses belonging to Bal can be detected.

Supplementary Table 10: ^{19}F-NMR based K_D determination of different protein and diol combinations at pH 7.0.

Entry	Protein and Diol	K_D [mM]
**1**	Histone H3 Bal9 + diol **4**	3.31±0.49
**2**	Histone H3 Bal9 + FDRibOMe (**5)**	43.2±7.0
**3**	Histone H3 Bal10 + diol **4**	6.22±0.55
**4**	Histone H3 Bal10 + FDRibOMe (**5)**	71.4±30.6
**5**	Histone H3 Bal9 + FDGal (**6**)	not detected

Supplementary Table 11: Oxaborolane formation observed via NMR at varying pH.

Entry	pH	% oxaborolane
1	5.40	0
2	6.01	0
3	7.06	2
4	7.94	14
5	9.08	50
6	10.16	63
7	12.02	64

Supplementary Table 12: Oxaborolane formation observed via NMR at various concentrations.

**c [mg/mL]**	**c [mM]**	**^{1}H-NMR**		**^{11}B-NMR**	**^{13}C-NMR**	
		Cβ-H	CH_3		Cβ	Cα
30	113.6	32.9	36.9	34.5	33.6	33.5
15	56.8	33.0	34.7	36.5	33.3	33.0
7.5	28.4	33.0	35.4	37.5	31.9	33.6
3.75	14.2	33.2	35.3	nd	nd	nd
1.875	7.1	33.1	37.4	nd	nd	nd

Supplementary Table 13: Residual Cu-content as determined by ICP-MS.

Entry	Sample	Cu-content [ppb]
**1**	Buffer blank	0.48±0.05
**2**	Histone H3-Bal10 (3×dialysed)	92.68±0.14

3 Supplementary Notes

3.1 Supplementary Note 1: Small Molecule Synthesis

3.1.1 General Considerations

For air and moisture sensitive reactions all used glassware has been dried by heating in high vacuum and purging with nitrogen. This procedure has been repeated three times prior to use. Syringes have been purged with nitrogen and airtight septa have been used for the addition of liquids to reactions performed under nitrogen atmosphere. Solid substances were added using nitrogen countercurrent where needed. Solvents were removed on a rotary evaporator where applicable. Chemical reagents were obtained from commercial suppliers (Alfa Aesar, Combi-Blocks, Fluorochem, Fisher Scientific, Sigma Aldrich, VWR) and used without further purification unless noted otherwise.

3.1.2 Chromatography

Chromatographic separations were performed by flash chromatography on CombiFlash instruments (Teledyne ISCO, USA). Analytical thin-layer chromatography (TLC) was performed on Merck aluminium backed sheets coated with silica gel 60 F_{254}. UV-active substances were visualized by short wavelength (254 nm) ultraviolet light. Other substances were visualized using a $KMnO_4$ staining solution and heating the TLC plate after application.

3.1.3 Analytical Techniques

3.1.3.1 Nuclear magnetic resonance spectroscopy (NMR spectroscopy)

Synthesized compounds were characterized by NMR spectroscopy. ^{1}H and ^{13}C NMR spectra were recorded using Bruker Advance-III HD 400 MHz, Bruker Advance-III HD 500 MHz and Bruker Advance-III HD 600 MHz spectrometers. ^{1}H- and ^{13}C-NMR spectra were referenced to the NMR solvent shift or to TMS. Chemical shifts are given in parts per million (ppm) and coupling constants are declared in Hertz (Hz). The multiplicities of the signals are reported as singlet (s), doublet (d), triplet (t), quartet (q),

pentet (pent), multiplet (m), broad (br) or any combination of these. Signals showing unexpected multiplicities are indicated by the prefix “app”.

3.1.3.2 Mass spectrometry

Protein mass spectra were recorded on a Waters Xevo G2-S QTof spectrometer, on a Waters Xevo G2-XS QTof spectrometer or an AB Sciex TripleTOF 6600 spectrometer.

Spectra were deconvoluted either using MassLynx (Waters) and the “MaxEnt1” deconvolution algorithm or Analyst (AB Sciex) using the “Reconstruct Protein” algorithm. Conversions were calculated from peak intensities (Waters) or peak areas (AB Sciex) dividing the value for the product by the sum of the values for products and (residual) starting material. Impurities present prior to the reaction were not considered.

3.1.3.3 IR spectrometry

IR spectra were recorded on a Bruker Tensor 27 Fourier Transform spectrophotometer equipped with an ATR probe or a Thermo Nexus 670 equipped with a silicon probe. Samples were loaded directly onto the probe.

3.1.3.4 Melting Points

Melting points were measured via differential scanning calorimetry (DSC) using a Mettler Toledo DSC 1 STAR^{e} system.

3.1.3.5 Optical Rotations

Optical rotations were recorded on a Schmidt+Haensch Unipol polarimeter using a cell with a length of 10 cm. Background measurements were performed before loading of the sample.

3.1.3.6 CD spectroscopy

CD spectra were recorded on a Chirascan device at 25ºC. Experiments were performed using 300 µL of protein solution in a 1 mm cuvette in buffer. Spectra were collected from 200 to 260 nm (0.5 nm step, 1 s per point) as a smoothened average of three scans. Buffer solution was used as a blank sample.

3.1.3.7 pK_a Determination

pKa values were determined using a Sirius T3 automated titrator (Pion Inc., United Kingdom). A stock solution of compound in DMSO was prepared and titrated from pH 2.0 to pH 12.0 in the presence of three varying concentrations of MeOH. HCl was used for acidification and KOH was used for titration. The pK_a value in a solely aqueous system was extrapolated from the measured pK_a values for the three different concentrations of organic co-solvent using Yasuda-Shedlovsky extrapolation.

3.1.4 Synthetic Procedures

3.1.4.1 Model Substrate

2-Acetylamino-*N*-benzyl-acrylamide (1)

The title compound was synthesized following a literature procedure.[10]

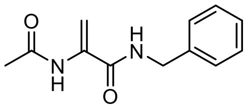

To a stirred solution of 2-acetamidoacrylic acid (1.29 g, 10.0 mmol, 1.00 equiv.) and 4-methylmorpholine (1.21 mL, 11.0 mmol, 1.10 equiv.) in THF (100 mL) were added subsequently isobutyl chloroformate (1.43 mL, 11 mmol, 1.10 equiv.) and benzylamine (1.20 mL, 11.0 mmol, 1.10 equiv.). The mixture was stirred at room temperature for 2 h, before it was filtered and the solvent was evaporated. The residue was purified by flash chromatography (*n*-heptane / EtOAc; 10-100% EtOAc) yielding compound **1** as a white solid (1.62 g, 7.43 mmol, 74%).

^{1}H-NMR (400 MHz, DMSO-d_6, 300 K): δ (ppm) = 9.08 (s, 1H, CON*H*), 8.86 (t, J = 6.1 Hz, 1H, CON*H*), 7.35 – 7.20 (m, 5H, 5 × Ar-*H*), 6.03 (s, 1H, C=CH_a), 5.44 (s, 1H, C=CH_b), 4.36 (d, J = 6.1 Hz, 2H, CH_2), 2.00 (s, 3H, CH_3).

^{13}C-NMR (101 MHz, DMSO-d_6, 300 K): δ (ppm) = 169.2 (*C*=O), 164.1 (*C*=O), 139.3 (C_{Ar}), 136.2 (*C*=CH_2), 128.2 (C_{Ar}), 127.2 (C_{Ar}), 126.8 (C_{Ar}), 103.0 (C=CH_2), 42.5 (CH_2), 23.8 (CH_3).

IR (silicone, neat): $\tilde{\nu}$ (cm^{-1}) = 3404, 3295, 3033, 2941, 1723, 1666, 1648, 1622, 1533, 1497, 1423, 1401, 1371, 1346, 1322, 1283, 1267, 1232, 1215, 1190, 1125, 1100, 1070, 1025, 1017, 1001, 990, 968, 954, 939, 923, 907, 867, 850, 839, 827, 806, 752, 726, 707, 694, 660.

m.p.: 126.8°C

HRMS (ESI+): *m/z* for $C_{12}H_{15}O_2N_2$ [M+H]$^+$: calculated: 219.1128; found: 219.1129.

(2-acetamido-3-(benzylamino)-3-oxopropyl)boronic acid (2)

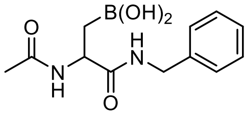

To a stirred suspension of 2-Acetylamino-N-benzyl-acrylamide (1.00 g, 4.58 mmol, 1.00 equiv.) and bis(pinacolato)diboron (1.28 g, 5.04 mmol, 1.10 equiv.) in 30 mL of water were added $CuSO_4 \cdot 5H_2O$ (57.2 mg, 0.23 mmol, 0.05 equiv.) and 4-methylpyridine (44.6 µL, 0.46 mmol, 0.10 equiv.). The reaction mixture was stirred overnight at room temperature before being filterted and lyophillized. The residue was dissolved in a mixture of 100 mL of THF and 100 mL of Et_2O and filtered. Diethanolamine (0.58 g, 5.50 mmol, 1.20 equiv.) was added and the mixture was stirred for 3 days. The precipitate was then filtered off, washed with Et_2O (3 × 50 mL) and dried in vacuo. The white solid was dissolved in 60 mL of 0.2 M HCl and stirred for 1 h. The solution was lyophilized and dissolved in 18 mL H_2O for purification. A purification was performed via preparative HPLC (Stationary phase: RP XBridge Prep C18 OBD-10µm, 50 × 250 mm, mobile phase: 0.25% NH_4HCO_3 solution in water, MeCN). The title compound **2** was afforded after lyophilisation as a white solid (485 mg, 1.84 mmol, 40%).

^{1}H-NMR (400 MHz, NaP_i (20 mM, pH 1.5, D_2O), 300 K): δ (ppm) = 7.47 – 7.24 (m, 5H, Ar-*H*), 4.45 – 4.33 (m, 3H, C*H* + CH_2NHBn), 2.02 (s, 3H, CH_3), 1.23 (app qd, J = 15.4, 8.0 Hz, 2H, CH_2).

^{13}C-NMR (101 MHz, NaP_i (20 mM, pH 1.5, D_2O), 300 K): δ (ppm) = 175.3 (*C*=O), 174.1 (*C*=O), 137.8 (C_{Ar}), 128.7 (C_{Ar}), 127.4 (C_{Ar}), 127.0 (C_{Ar}), 51.6 (*C*H), 42.8 (*C*H_2), 21.7 (*C*H_3), 18.2 (br s, CH_2).

^{11}B-NMR (128 MHz, NaP_i (20 mM, pH 1.5, D_2O), 300 K): δ (ppm) = 29.7 (br s, *B*$(OH)_2$).

IR (silicone, neat): $\tilde{\nu}$ (cm^{-1}) = 3282, 3078, 2933, 1631, 1531, 1498, 1454, 1420, 1374, 1259, 1080, 1029, 897, 734, 699.

m.p.: not determined due to decomposition

HRMS (ESI+): *m/z* for $C_{12}H_{17}O_4N_2{}^{11}BNa$ $[M+Na]^+$: calculated: 287.1174; found: 287.1175.

pK_a (aqueous solution, 25 °C): 8.31 ± 0.02

3.1.4.2 [^{15}N]-labelled Model Substrate

[^{15}N]Benzamide (8)

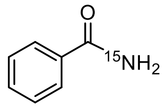

[^{15}N]Benzamide was synthesized following a reported procedure.[11] [^{15}N]NH_4Cl (98% ^{15}N, 1.00 g, 18.4 mmol, 1.00 equiv.) was dissolved in water (15 mL) and toluene (2 mL) was added. The mixture was cooled to 0 °C. NaOH (4 M, 10 mL, 2.20 equiv., 40.4 mmol) was added followed by a solution of benzoyl chloride (2.2 mL, 1.02 equiv., 18.7 mmol) in toluene (80 mL) was added slowly. After addition, the mixture was allowed to warm to room temperature and stirred overnight. The white precipitate was collected by filtration, washed with ice cold water (3 × 10 mL) and dried *in vacuo* to yield the title compound as a white solid (1.70 g, 13.9 mmol, 76%).

^{1}H-NMR (400 MHz, $CDCl_3$, 295 K): δ (ppm) = 7.84 – 7.79 (m, 2H, Ar-*H*), 7.55 – 7.49 (m, 1H, Ar-*H*), 7.47 – 7.41 (m, 2H, Ar-*H*), 6.24 (d, *J* = 88.7 Hz, 2H, CON*H*$_2$).

^{13}C-NMR (101 MHz, $CDCl_3$, 295 K): δ (ppm) = 169.8 (d, *J* = 15.4 Hz, *C*ONH$_2$), 133.5 (d, *J* = 8.1 Hz, C_{Ar}), 132.1 (C_{Ar}), 128.7 (C_{Ar}), 127.5 (C_{Ar}).

HRMS (ESI+): *m/z* for $C_7H_8{}^{15}NO$ $[M+H]^+$: calculated: 123.0571; found: 123.0571.

The analytical data are in agreement with the literature.[11]

[15N]Benzylamine (9)

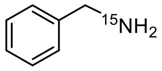

[15N]Benzylamine was synthesized following a reported procedure.[11] [15N]Benzylamide (1.00 g, 8.25 mmol, 1.00 equiv.) was dissolved in dry THF and added to a suspension of $LiAlH_4$ (934 mg, 24.8 mmol, 3.00 equiv.) in THF (40 mL) at 0 °C. The solution was warmed to room temperature and refluxed for 2.5 h. After cooling back to room temperature, the resulting mixture was slowly added to ice water (200 mL) and filtered through a plug of celite. NaOH (4 M, 10 mL) was added and the aqueous solution was extracted with EtOAc (3 × 100 mL). The combined organic extracts were dried over Na2SO4 and the solvent was evaporated to afford [15N]benzylamine as a yellow oil (784 mg, 8.19 mmol, 89%).

^{1}H-NMR (400 MHz, $CDCl_3$, 295 K): δ (ppm) = 7.39 – 7.22 (m, 5H, Ar-*H*), 3.89 (s, 2H, CH_2), 1.55 (br s, 2H, NH_2).

^{13}C-NMR (101 MHz, $CDCl_3$, 295 K): δ (ppm) = 143.5 (C_{Ar}), 128.7 (C_{Ar}), 127.2 (C_{Ar}), 126.9 (C_{Ar}), 46.65 (d, *J* = 3.9 Hz, CH2).

HRMS (ESI+): *m/z* for $C_7H_9{}^{15}N$ $[M+H]^+$: calculated: 109.0778; found: 109.0780.

The analytical data are in agreement with the literature.[11]

2-Acetylamino-*N*-benzyl-acryl-[^{15}N]-amide (10)

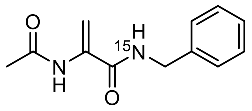

2-Acetamidoacrylic acid (837 mg, 6.49 mmol, 1.10 equiv.) was suspended in THF (40 mL). *N*-methylmorpholine (713 µL, 6.49 mmol, 1.10 equiv.), isobutyl chloroformate (841 µL, 6.49 mmol, 1.10 equiv.) and [^{15}N]benzylamine (650 µL, 5.90 mmol, 1.00 equiv.) were added subsequently. The mixture was stirred overnight at room temperature, filtered through a plug of celite and purified by flash chromatography (0-100% EtOAc in petroleum ether) and recrystallized from EtOH/Et_2O yielding the title compound as a white solid (715 mg, 3.26 mmol, 55%).

^{1}H-NMR (400 MHz, DMSO-*d6*, 295 K): δ (ppm) = 9.08 (br s, 1H, CON*H*), 8.86 (dt, *J* = 92.8, 6.1 Hz, 1H, CO^{15}N*H*), 7.36 – 7.19 (m, 5H, Ar-*H*), 6.03 (s, 1H, C=C*H*), 5.43 (s, 1H, C=C*H*), 4.36 (d, *J* = 5.6 Hz, 2H, CH_2), 2.00 (s, 3H, CH_3).

^{13}C-NMR (101 MHz, DMSO-*d6*, 295 K): δ (ppm) = 169.2 (*C*ONH), 164.1 (d, *J* = 17.1 Hz, *C*O^{15}NH), 139.3 (C_{Ar}), 136.1 (d, *J* = 9.2 Hz, *C*=CH_2), 128.2 (C_{Ar}), 127.2 (C_{Ar}), 126.8 (C_{Ar}), 102.9 (C=*C*H), 42.5 (d, *J* = 10.7 Hz, CH_2), 23.8 (CH_3).

HRMS (ESI+): *m/z* for $C_{12}H_{14}N^{15}NO_2Na$ $[M+Na]^+$: calculated: 242.0918; found: 242.0918.

(2-acetamido-3-(benzyl-[^{15}N]-amino)-3-oxopropyl)boronic acid (11)

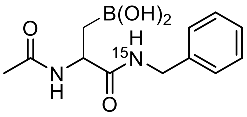

2-Acetylamino-*N*-benzyl-acryl-[^{15}N]-amide (300 mg, 1.37 mmol, 1.00 equiv.), $B_2(OH)_4$ (245 mg, 2.74 mmol, 2.00 equiv.) and $CuSO_4 \cdot 5H_2O$ (34 mg, 0.14 mmol, 10 mol-%) were suspended in distilled water (30 mL). 4-picoline was added (33 µL, 0.34 mmol, 25 mol-%) and the mixture was stirred vigorously open to air at room temperature for 3 h. Another portion of $B_2(OH)_4$ (245 mg, 2.74 mmol, 2.00 equiv.) was added and stirring was continued overnight. The solution was lyophilized and dissolved in 18 mL H_2O for purification. Purification was performed via preparative HPLC (stationary phase: RP XBridge Prep C18 OBD-10 µm, 50 × 250 mm, mobile phase: 0.25% NH_4HCO_3 solution in water, MeCN). The title compound was afforded after lyophilisation as an off-white solid (155 mg, 0.58 mmol, 43%).

^{1}H-NMR (400 MHz, D_2O (5% conc. DCl), 295 K): δ (ppm) = 7.21 – 7.06 (m, 5H, Ar-*H*), 4.25 – 4.10 (m, 3H, C*H* + CH_2NHBn), 1.89 (s, 1H, CH_3), 1.05 – 0.81 (m, 2H, CH_2).

^{13}C-NMR (101 MHz, D_2O (5% conc. DCl), 295 K): δ (ppm) = 174.5 (d, *J* = 16.7 Hz, *C*=O), 174.4 (*C*=O), 137.5 (C_{Ar}), 128.6 (C_{Ar}), 127.3 (C_{Ar}), 127.0 (C_{Ar}), 52.9 – 51.7 (m, C_α), 42.9 (dd, *J* = 9.9, 4.9 Hz), 21.3 (CH_3), 17.2 (CH_3).

^{11}B-NMR (128 MHz, D_2O (5% conc. DCl), 295 K): δ (ppm) = 21.66 (br s).

HRMS (ESI+): *m/z* for $C_{12}H_{17}O_4N^{15}N^{11}BNa$ $[M+Na]^+$: calculated: 288.1144; found: 288.1147.

[15N]-Acetamide (12)

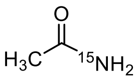

To a solution of [^{15}N]NH_4Cl (98% ^{15}N, 1.00 g, 18.4 mmol, 1.00 equiv.) in NaOH (10 M, 4.1 mL, 41.1 mmol, 2.20 equiv.) and toluene (40 mL) was added acetyl chloride (1.5 mL, 20.6 mmol, 1.10 equiv.) at 0 °C. The solution was allowed to warm to room temperature and vigorously stirred overnight. The solvent was evaporated and the solid residue was extracted with boiling acetone (3 × 50 mL). The combined extracts were evaporated to dryness to yield [^{15}N]-acetamide as white needles (724 mg, 18.7 mmol, 66%).

^{1}H-NMR (400 MHz, $CDCl_3$, 295 K): δ (ppm) = 5.75 (dd, *J* = 88.3, 3.1 Hz, 1H, CON*H*), 5.59 (dd, *J* = 88.9, 3.2 Hz, 1H, CON*H*), 2.00 (d, *J* = 1.2 Hz, 3H, CH_3).

^{13}C-NMR (101 MHz, $CDCl_3$, 295 K): δ (ppm) = 173.0 (d, *J* = 14.1 Hz), 22.8 (d, *J* = 9.0 Hz).

HRMS (ESI+): *m/z* for $C_2H_6{}^{15}NO$ [M+H]$^+$: calculated: 61.0414; found: 61.0420.

The spectroscopic data is in agreement with the literature.[12]

[^{15}N]-Acetamidoacrylic acid (13)

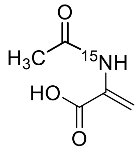

Following a literature procedure,[13] a solution of [^{15}N]-acetamide (600 mg, 10.0 mmol, 1.00 equiv.) and pyruvic acid (1.5 mL, 22.0 mmol, 2.20 equiv.) in toluene (80 mL) were refluxed under Dean-Stark conditions overnight. The solvent was evaporated and the solid residue was titurated with ether to afford the title compound as light brown solid containing impurities (667 mg). The compound was used without further purification.

^{1}H-NMR (400 MHz, DMSO-*d6*, 295 K): δ (ppm) = 13.27 (br s, 1H, COO*H*), 9.09 (dd, *J* = 91.9, 1.1 Hz, 1H, CO^{15}N*H*), 6.23 (t, *J* = 1.2 Hz, 1H, C=C*H*), 5.65 (dt, *J* = 4.3, 1.1 Hz, 1H, C=C*H*), 2.02 (d, *J* = 1.4 Hz, 3H, CH_3).

^{13}C-NMR (101 MHz, DMSO-*d6*, 295 K): δ (ppm) = 169.7 (d, *J* = 12.8 Hz, *C*ONH), 165.5 (d, *J* = 2.7 Hz, *C*OOH), 133.8 (d, *J* = 15.7 Hz, *C*=CH_2), 109.0 (C=CH_2), 24.3 (d, *J* = 9.4 Hz, CH_3).

HRMS (ESI+): *m/z* for $C_5H_8{}^{15}NO_3$ $[M+H]^+$: calculated: 131.0469; found: 131.0469.

2-Acetyl-[^{15}N]-amino-*N*-benzyl-acrylamide (14)

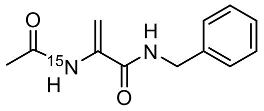

[^{15}N]-2-Acetamidoacrylic acid (400 mg, 3.07 mmol, 1.00 equiv.) was suspended in THF (30 mL). *N*-methylmorpholine (372 µL, 3.38 mmol, 1.10 equiv.), isobutyl chloroformate (440 µL, 3.38 mmol, 1.10 equiv.) and benzylamine (369 µL, 3.38 mmol, 1.10 equiv.) were added subsequently. The mixture was stirred at room temperature for 1 h, filtered through a plug of celite and purified by flash chromatography (0-100% EtOAc in petroleum ether) affording the title compound as a pale yellow solid (252 mg, 1.15 mmol, 37%).

^{1}H-NMR (400 MHz, DMSO-*d6*, 295 K): δ (ppm) = 9.08 (d, *J* = 91.5 Hz, 1H, CO^{15}N*H*), 8.86 (t, *J* = 6.1 Hz, 1H, CON*H*), 7.36 – 7.19 (m, 5H, Ar-*H*), 6.03 (d, *J* = 1.4 Hz, 1H, C=C*H*), 5.43 (d, *J* = 4.4 Hz, 1H, C=C*H*), 4.36 (d, *J* = 6.1 Hz, 2H, CH_2), 2.00 (d, *J* = 1.3 Hz, 3H, CH_3).

^{13}C-NMR (101 MHz, DMSO-*d6*, 295 K): δ (ppm) = 169.2 (d, *J* = 12.9 Hz, *C*O^{15}NH), 164.1 (d, *J* = 2.5 Hz, *C*ONH), 139.3 (C_{Ar}), 136.2 (d, *J* = 15.6 Hz, *C*=CH_2), 102.9 (C=CH_2), 42.5 (CH_2), 23.8 (d, *J* = 9.4 Hz, CH_3).

HRMS (ESI+): *m/z* for $C_{12}H_{14}N^{15}NO_2Na$ $[M+Na]^+$: calculated: 242.0918; found: 242.0918.

(2-acet-[^{15}N]-amido-3-(benzylamino)-3-oxopropyl)boronic acid (15)

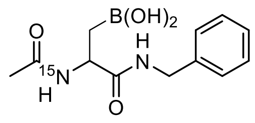

2-Acetyl-[^{15}N]-amino-*N*-benzyl-acrylamide (150 mg, 0.68 mmol, 1.00 equiv.), $B_2(OH)_4$ (123 mg, 1.37 mmol, 2.00 equiv.) and $CuSO_4 \cdot 5H_2O$ (17 mg, 0.07 mmol, 10 mol-%) were suspended in distilled water (15 mL). 4-picoline was added (16.6 µL, 0.17 mmol, 25 mol-%) and the mixture was stirred vigorously open to air at room temperature for 3 h. Another portion of $B_2(OH)_4$ (123 mg, 1.37 mmol, 2.00 equiv.) was added and stirring was continued overnight. The solution was lyophilized and dissolved in 18 mL H_2O for purification. Purification was performed via preparative HPLC (stationary phase: RP XBridge Prep C18 OBD-10 µm, 50 × 250 mm, mobile phase: 0.25% NH_4HCO_3 solution in water, MeCN). The title compound was afforded after lyophilisation as an off-white solid (101 mg, 0.38 mmol, 56%).

^{1}H-NMR (400 MHz, D_2O (5% conc. DCl), 295 K): δ (ppm) = 7.27 – 7.02 (m, 5H, Ar-*H*), 4.24 – 4.09 (m, 3H, C*H* + C*H*$_2$NHBn), 1.89 (d, *J* = 1.5 Hz, 3H, CH_3), 1.04 – 0.80 (m, 2H, CH_2).

^{13}C-NMR (101 MHz, D_2O (5% conc. DCl), 295 K): δ (ppm) = 174.5 (*C*=O), 174.4 (d, *J* = 15.6 Hz, *C*=O), 137.5 (C_{Ar}), 128.6 (C_{Ar}), 127.3 (C_{Ar}), 127.0 (C_{Ar}), 52.3 (dd, *J* = 9.9, 5.1 Hz, C_{quart}), 42.9 (app t, *J* = 4.7 Hz, CH_2), 21.3 (d, *J* = 6.2 Hz, CH_3), 17.2 (CH_2).

^{11}B-NMR (128 MHz, D_2O (5% conc. DCl), 295 K): δ (ppm) = 20.65 (br s).

HRMS (ESI+): *m/z* for $C_{12}H_{17}O_4N^{15}N^{11}BNa$ $[M+Na]^+$: calculated: 288.1144; found: 288.1145.

3.1.4.3 Fluorinated Diol Probe

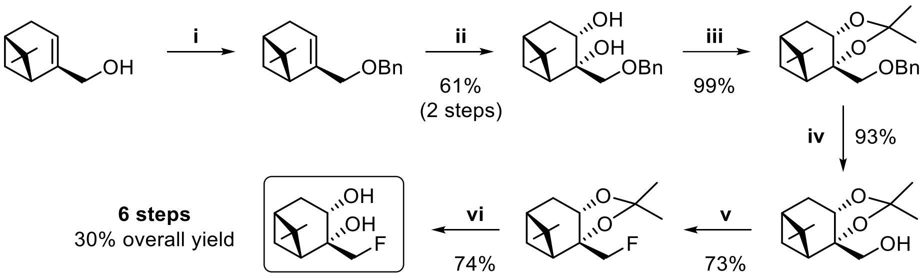

Synthesis of ^{19}F-NMR diol probe **4**: (i) NaH, BnBr, DMF; (ii) K_2OsO_4, NMO, 4-picoline, iPrOH; (iii) 2,2-dimethoxypropane, *p*TsOH·Py, acetone; (iv) H_2, Pd/C, MeOH; (v) DAST, Et_3N, toluene; (vi) Amberlyst 15, EtOH, H_2O.

(1R)-(−)-Myrtenoldiol benzylether (16)

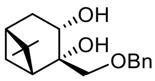

To an ice cold solution of (1R)-(−)-Myrtenol (4.0 mL, 25.2 mmol, 1.00 equiv.) in DMF (100 mL) was added NaH (60% in mineral oil) (1.5 g, 37.7 mmol, 1.50 equiv.) in small portions. The mixture was stirred for 30 min before benzyl bromide (3.6 mL, 30.2 mmol, 1.20 equiv.) was added. The mixture was allowed to warm to room temperature and stirred overnight. The reaction mixture was cooled to 0°C, 300 mL of H_2O and 100 mL of brine were added and the mixture was extracted with EtOAc (4 × 100 mL). The combined organic extracts were washed with brine (3 × 100 mL), dried over Na_2SO_4 and concentrated *in vacuo*. The residual oil was dissolved in 125 mL of iPrOH and 4-picoline (3.2 mL, 32.7 mmol, 1.30 equiv.), a solution of 4-Methylmorpholine *N*-oxide monohydrate (4.42 g, 32.7 mmol, 1.30 equiv.) in 5 mL of H_2O and $K_2OsO_4·2H_2O$ (185 mg, 0.50 mmol, 2 mol-%) were added to the solution. The mixture was refluxed for 20 h. Most of the solvent was evaporated and the crude product was purified by flash chromatography (silica gel, 0−40% EtOAc in petroleum ether). Fractions containing product were pooled and the solvent was evaporated. The oily residue was treated with petroleum ether yielding compound **S10** as white needles (4.23 g, 15.3 mmol, 61%).

^{1}H-NMR (400 MHz, DMSO-d_6, 300 K): δ (ppm) = 7.40 – 7.22 (m, 5H, Ar-*H*), 4.97 (d, *J* = 5.7 Hz, 1H, O*H*), 4.50 (dd, *J* = 26.3, 12.6 Hz, 2H, CH_2), 4.36 (s, 1H, O*H*), 3.81 (dt, *J* = 10.1, 5.3 Hz, 1H, C*H*), 3.29 (dd, *J* = 43.8, 10.3 Hz, 2H, CH_2), 2.30 (dddd, *J* = 13.6, 9.3, 3.5, 2.0 Hz, 1H, C*H*), 2.13 – 2.01 (m, 2H, 2 × C*H*), 1.78 (tdd, *J* = 5.9, 3.6, 2.6 Hz, 1H,

C*H*), 1.54 (ddd, *J* = 13.8, 5.1, 2.5 Hz, 1H, C*H*), 1.38 (d, *J* = 9.0 Hz, 1H, C*H*), 1.17 (s, 3H, CH_3), 0.74 (s, 3H, CH_3).

^{13}C-NMR (101 MHz, DMSO-d_6, 300 K): δ (ppm) = 138.9 (C_{Ar}), 128.1 (C_{Ar}), 127.4 (C_{Ar}), 127.3 (C_{Ar}), 76.8 (CH_2), 74.6 (C_{quart}), 72.4 (CH_2), 63.8 (*C*H), 48.4 (*C*H), 40.1 (*C*H), 38.1 (C_{quart}), 37.8 (CH_2), 27.7 (CH_3), 27.2 (CH_2), 23.9 (CH_2).

IR (diamond ATR, neat): $\tilde{\nu}$ (cm^{-1}) = 3364, 2991, 2955, 2910, 2859, 2161, 2032, 1496, 1455, 1439, 1389, 1366, 1331, 1296, 1227, 1200, 1116, 1087, 1038, 1016, 986, 960, 940, 903, 850, 810, 742, 698, 647.

m.p.: 83.6 °C.

HRMS (ESI+): *m/z* for $C_{17}H_{24}O_3Na$ $[M+Na]^+$: calculated: 299.1618; found: 299.1616.

(1R)-(−)-Myrtenoldiol benzylether acetonide (17)

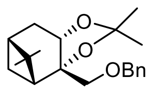

Compound **16** (2.00 g, 7.24 mmol, 1.00 equiv.) was dissolved in 75 mL of acetone. 2,2-dimethoxypropane (2.7 mL, 21.7 mmol, 3.00 equiv.) and pyridinium *p*-toluenesulfonate (273 mg, 1.09 mmol, 0.15 equiv.) were added and the mixture was stirred for 16 h at room temperature. The solvent was evaporated and the residue was suspended in 10 mL of a mixture of petroleum ether and EtOAc (8:2, v/v). The solid was removed by filtration and washed with 5 mL of petroleum ether/EtOAc (8:2, v/v) twice. The combined filtrates were evaporated to afford compound **17** as pale yellow oil (2.27 g, 7.19 mmol, 99%).

^{1}H-NMR (400 MHz, DMSO-d_6, 300 K): δ (ppm) = 7.54 – 7.10 (m, 5H, Ar-*H*), 4.50 (dd, *J* = 33.1, 11.8 Hz, 2H, CH_2OBn), 4.19 (d, *J* = 6.9 Hz, 1H, C*H*), 3.64 – 3.55 (m, 2H, CH_2), 2.18 – 2.11 (m, 1H, C*H*), 2.11 – 2.02 (m, 2H, 2 × CH_2), 1.90 – 1.77 (m, 2H, 2 × CH_2), 1.59 (d, J = 10.0 Hz, 1H, CH_2), 1.37 (s, 3H, CH_3), 1.30 (s, 3H, CH_3), 1.21 (s, 3H, CH_3), 0.71 (s, 3H, CH_3).

^{13}C-NMR (101 MHz, DMSO-d_6, 300 K): δ (ppm) = 138.3 (C_{Ar}), 128.1 (C_{Ar}), 127.4 (C_{Ar}), 127.3 (C_{Ar}), 107.1 (C_{quart}), 86.2 (C_{quart}), 75.3 (CH_2), 72.2 (CH_2), 71.2 (*C*H), 46.1 (C*H*), 37.6 (C_{quart}), 31.5 (CH_2), 27.4 (CH_3), 27.0 (CH_3), 26.0 (CH_3), 24.1 (CH_2) 22.6 (CH_3).

IR (silicone, neat): $\tilde{\nu}$ (cm^{-1}) = 2984, 2936, 2869, 2361, 2160, 2028, 1722, 1454, 1368, 1315, 1258, 1206, 1141, 1096, 1057, 1036, 1000, 980, 926, 893, 865, 851, 822, 736, 712, 697, 645.

HRMS (ESI+): *m/z* for $C_{20}H_{28}O_3Na$ $[M+Na]^+$: calculated: 339.1931; found: 339.1931.

1H NMR (400 MHz, DMSO-d6) δ 7.54 – 7.10 (m, 5H), 4.50 (dd, J = 33.1, 11.8 Hz, 2H), 4.19 (d, J = 6.9 Hz, 1H), 3.64 – 3.55 (m, 2H), 1.90 – 1.77 (m, 2H), 1.59 (d, J = 10.0 Hz, 1H), 1.37 (s, 3H), 1.30 (s, 3H), 1.21 (s, 3H), 0.71 (s, 3H).

(1R)-(−)-Myrtenoldiol acetonide (18)

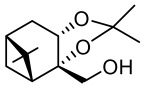

To a stirred solution of compound **17** (2.00 g, 6.32 mmol, 1.00 equiv.) in methanol (60 mL) was added palladium on carbon (loading 10% wt, 673 mg, 0.63 mmol, 10 mol-%). The suspension was purged with nitrogen for 10 min, purged with hydrogen for another 10 min and stirred under hydrogen atmosphere for 1 h. The mixture was filtered through a plug of celite and the solvent was removed *in vacuo* yielding compound **18** as colorless oil (1.38 g, 6.23 mmol, 96%).

^{1}H-NMR (400 MHz, $CDCl_3$, 300 K): δ (ppm) = 4.08 (d, J = 7.2 Hz, 1H, CH), 3.75 – 3.61 (m, 1H, CH_2), 2.46 (br s, 1H, OH), 2.26 (dd, J = 6.1, 5.0 Hz, 1H, CH), 2.18 – 2.05 (m, 2H, CH_2), 1.97 – 1.84 (m, 2H, CH_2 + CH), 1.59 (d, J = 10.4 Hz, 1H, CH_2), 1.46 (s, 1H, CH_3), 1.36 (s, 1H, CH_3), 1.24 (s, 1H, CH_3), 0.78 (s, 1H, CH_3).

^{13}C-NMR (101 MHz, $CDCl_3$, 300 K): δ (ppm) = 108.0 (C_{quart}), 87.5 (C_{quart}), 73.4 (CH), 68.1 (CH$_2$), 45.7 (CH), 40.2 (CH), 38.1 (C_{quart}), 32.4 (CH$_2$), 27.6 (CH$_3$), 27.1 (CH$_3$), 27.0 (CH$_3$), 24.8 (CH$_2$), 23.7 (CH$_3$).

IR (diamond ATR, neat): $\tilde{\nu}$ (cm^{-1}) = 3461, 2985, 2936, 2160, 1456, 1369, 1242, 1206, 1187, 1141, 1095, 1045, 999, 976, 918, 892, 862, 822, 766, 688, 645.

HRMS (ESI+): *m/z* for $C_{13}H_{22}O_3Na$ [M+Na]$^+$: calculated: 249.1461; found: 249.1464.

Fluorodeoxy-(1R)-(−)-myrtenoldiol acetonide (19)

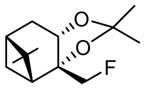

Alcohol **18** (300 mg, 1.33 mmol, 1.00 equiv.) was dissolved in dry toluene (15 mL). Triethylamine (0.55 mL, 3.98 mmol, 3.00 equiv.) and DAST (0.53 mL, 3.98 mmol, 3.00 equiv.) were added and the solution was heated to 75°C for 20 h. The mixture was cooled to room temperature and 1 mL of methanol was added. The solvent was evaporated and the crude product was purified by flash chromatography (silica gel, 0−20% EtOAc in petroleum ether) affording compound **19** as yellow oil (220 mg, 0.96 mmol, 73%).

^{1}H-NMR (400 MHz, $CDCl_3$, 300 K): δ (ppm) = 4.58 (dd, *J* = 24.4, 10.1 Hz, 1H, CH_2), 4.46 (dd, *J* = 23.1, 10.1 Hz, 1H, CH_2), 4.28 (d, *J* = 7.0 Hz, 1H, C*H*), 2.23 – 2.14 (m, 2H, CH_2), 2.11 (t, *J* = 5.6 Hz, 1H, C*H*), 2.02 (dd, *J* = 14.7, 4.1 Hz, 1H, CH_2), 1.97 –1.90 (m, 1H, C*H*), 1.72 (d, *J* = 10.3 Hz, 1H, CH_2), 1.51 (s, 1H, CH_3), 1.40 (s, 1H, CH_3), 1.29 (s, 1H, CH_3), 0.85 (s, 1H, CH_3).

^{13}C-NMR (101 MHz, $CDCl_3$, 300 K): δ (ppm) = 108.6 (C_{quart}), 87.4 (d, *J* = 175.9 Hz, CH_2F), 86.4 (d, *J* = 16.7 Hz, C_{quart}), 70.8 (d, *J* = 6.3 Hz, *C*H), 45.9 (d, *J* = 5.9 Hz, *C*H), 40.1 (*C*H), 38.1 (C_{quart}), 32.2 (CH_2), 27.7 (CH_3), 27.3 (CH_3), 26.32 (d, *J* = 5.4 Hz, CH_3), 24.6 (CH_2), 23.6 (CH_3).

^{19}F-NMR (376 MHz, $CDCl_3$, 300 K) δ (ppm) = –225.76 (t, *J* = 47.6 Hz).

IR (diamond ATR, neat): $\tilde{\nu}$ (cm^{-1}) = 2994, 2957, 2932, 2885, 2161, 2031, 1461, 1377, 1366, 1281, 1253, 1203, 1178, 1144, 1123, 1099, 1057, 1038, 1012, 972, 936, 919, 894, 863, 853, 820, 768, 692, 648.

m.p.: 41.3 °C.

HRMS (ESI+): *m/z* for $C_{13}H_{21}O_2FNa$ $[M+Na]^+$: calculated: 251.1418; found: 251.1419.

Fluorodeoxy-(1R)-(−)-myrtenoldiol (4)

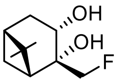

To a solution of acetonide **19** (230 mg, 1.01 mmol, 1.00 equiv.) in ethanol (25 mL) and water (25 mL) was added Amberlyst® 15 strong cation exchange resin (300 mg). The mixture was refluxed for two days, filtered through a plug of celite and purified by flash chromatography (silica gel, 0-60% EtOAc in petroleum ether) to yield fluorinated diol **4** as pale yellow oil (141 mg, 0.75 mmol, 74%).

^{1}H-NMR (400 MHz, DMSO-d_6, 300 K): δ (ppm) = 5.26 (d, J = 6.3 Hz, 1H, OH), 4.52 (s, 1H, OH), 4.24 (dd, J = 23.8, 9.4 Hz, 1H, CH_2F), 4.12 (dd, J = 24.3, 9.4 Hz, 1H, CH_2F), 3.87 (dddd, J = 9.4, 6.3, 5.1, 1.2 Hz, 1H, CH), 2.35 (dddd, J = 13.9, 9.4, 3.6, 2.3 Hz, 1H, CH_2), 2.11 (dtdd, J = 9.6, 6.0, 2.3, 0.9 Hz, 1H, CH_2), 2.00 (t, J = 6.2 Hz, 1H, CH), 1.82 (tdd, J = 5.9, 3.6, 2.6 Hz, 1H, CH), 1.57 (ddd, J = 13.8, 5.1, 2.5 Hz, 1H, CH_2), 1.40 (d, J = 9.9 Hz, 1H, CH_2), 1.21 (s, 3H, CH_3), 0.88 (s, 3H, CH_3).

^{13}C-NMR (101 MHz, DMSO-d_6, 300 K): δ (ppm) = 88.4 (d, J = 173.2 Hz, CH$_2$F), 73.8 (d, J = 16.0 Hz, C_{quart}), 62.2 (d, J = 5.6 Hz, CH), 47.6 (d, J = 4.3 Hz, CH), 40.1 (CH), 38.1 (C_{quart}), 37.5 (CH$_2$), 27.6 (CH$_3$), 27.1 (CH$_2$), 24.0 (CH$_3$).

^{19}F-NMR (376 MHz, DMSO-d_6, 300 K) δ (ppm) = – 228.40 (t, J = 48.0 Hz).

IR (diamond ATR, neat): $\tilde{\nu}$ (cm^{-1}) = 3362, 2925, 2361, 2161, 2031, 1455, 1386, 1368, 1335, 1273, 1216, 1154, 1127, 1066, 1048, 1023, 956, 933, 904, 847, 830, 743, 671, 625.

HRMS (ESI+): m/z for $C_{10}H_{17}O_2FNa$ $[M+Na]^+$: calculated: 211.1105; found: 211.1105.

3.1.4.4 2-Deoxy-2-fluoro-D-galactose (FDGal) (6)

1,3,4,6-tetra-*O*-acetyl-2-deoxy-2-fluoro-D-galactopyranose (20)

Compound **20** was synthesized following a known literature procedure.[14]

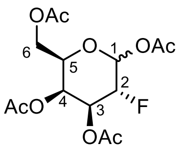

3,4,6-Tri-*O*-acetyl-D-galactal (1.04 g, 3.82 mmol, 1.00 equiv.) and SelectFluor (2.03 g, 5.73 mmol, 1.50 equiv.) were dissolved in a mixture of MeCN (40 mL) and AcOH (20 mL). The solution was heated to 70°C for 3 hours. The solvent was evaporated, and the residue was dissolved in 100 mL of EtOAc, washed with water (100 mL), saturated $NaHCO_3$ solution (100 mL) and brine (100 mL) and the solvent was evaporated. The crude residue was purified by flash chromatography (10-100% EtOAc in *n*-heptane) to yield the title compound as a white solid (302 mg, 0.86 mmol, 23%).

^{1}H-NMR (400 MHz, $CDCl_3$, 300 K): δ (ppm) = 6.47 (d, *J* = 4.0 Hz, 0.6H, H-C1α), 5.79 (dd, *J* = 8.0, 4.1 Hz, 1H, H-C1β), 5.52 (td, *J* = 3.4, 1.4 Hz, 0.6H, H-C4α), 5.49 – 5.43 (m, 1H, H-C4β), 5.44 (ddd, *J* = 11.0, 10.2, 3.5 Hz, 0.6H, H-C3α), 5.18 (ddd, *J* = 13.2, 9.8, 3.6 Hz, 1H, H-C3β), 4.89 (ddd, *J* = 49.2, 10.2, 4.0 Hz, 0.6H, H-C2α), 4.65 (ddd, *J* = 51.5, 9.9, 8.1 Hz, 1H, H-C2β), 4.31 (td, *J* = 6.9, 1.3 Hz, 0.6H, H-C5α), 4.20 – 4.04 (m, 4.2H, 2 × H-C6α + 2 × H-C6β + H-C5β), 2.19 (2 × s, 4.8H, CH_3α + CH_3β), 2.15 (s, 4.8H, CH_3α + CH_3β), 2.07 (s, 3H, CH_3β), 2.06 (s, 1.8H, CH_3α), (2 × s, 4.8H, CH_3α + CH_3β).

^{19}F-NMR (376 MHz, $CDCl_3$, 300 K) δ (ppm) = –208.10 (dddd, *J* = 51.4, 13.1, 4.1, 2.6 Hz, β-anomer), –209.12 (ddd, *J* = 49.2, 11.1, 3.6 Hz, α-anomer).

The spectroscopic data are in accordance with known literature data for **20**.[14]

2-Deoxy-2-fluoro-D-galactopyranose (6)

1,3,4,6-Tetra-O-acetyl-2-deoxy-2-fluoro-D-galactopyranose (**20**) (100 mg, 0.29 mmol, 1.00 equiv.) was dissolved in 2 mL of MeOH. 66 µL (28.5 µmol, 10 mol-%) of a solution of sodium metal (10 mg) in MeOH (1 mL) were added. The mixture was stirred for 1 h at room temperature before being neutralized by addition of Dowex 50WX8 strongly acidic cation exchange resin (15 mg). The mixture was stirred for an additional 10 min, the resin was filtered off and the solvent was evaporated to yield a sticky oil which was dissolved in 1 mL of water. The solution was lyophilized to afford the title compound (**6**) as a colorless powder (52 mg, 0.29 mmol, quantitative yield).

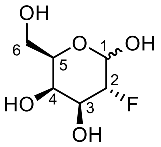

^{1}H-NMR (400 MHz, D_2O, 300 K): δ (ppm) = 5.51 (d, *J* = 4.0 Hz, 0.6H, H-C1α), 4.88 (dd, *J* = 7.8, 3.4 Hz, 1H, H-C1β), 4.77 – 4.62 (m, 0.6H, H-C2α), 4.37 (ddd, *J* = 52.0, 9.5, 7.8 Hz, 1H, H-C2β), 4.19 – 4.11 (m, 1.2H, H-C5α + H-C3α), 4.10 (td, *J* = 3.7, 1.2 Hz, 0.6H, H-C4α), 4.04 (app t, *J* = 3.4 Hz, 1H, H-C4β), 3.98 (ddd, *J* = 14.3, 9.5, 3.6 Hz, 1H, H-C3β), 3.82 – 3.76 (m, 4.2H, 2 × H-C2α + 2 × H-C2β + H-C5β).

^{19}F-NMR (376 MHz, $CDCl_3$, 300 K) δ (ppm) = –207.36 (ddt, *J* = 52.1, 14.5, 3.0 Hz, β-anomer), –207.53 (ddd, *J* = 49.7, 12.5, 3.8 Hz, α-anomer).

The spectroscopic data are in accordance with known literature data for compound **6**.[15,16]

3.1.4.5 Triol

(1R)-(−)-Myrtenoldiol (3)

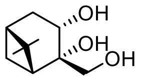

(1R)-(−)-Myrtenol (3.2 mL, 20.0 mmol, 1.00 equiv.), $K_2OsO_4·2H_2O$ (147 mg, 0.40 mmol, 2 mol-%) and 4-picoline (2.5 mL, 26.0 mmol, 1.30 equiv.) were dissolved in 40 mL of iPrOH. A solution of 4-Methylmorpholine *N*-oxide monohydrate (3.51 g, 26.0 mmol, 1.30 equiv.) in 3 mL of H_2O was added and the mixture was refluxed for 24 h. Most of the solvent was evaporated and the residue was diluted with 5 mL of EtOAc. The crude compound was purified by flash chromatography (silica gel, 20−100% EtOAc in petroleum ether) to yield (1R)-(−)-Myrtenoldiol (**3**) as a white solid (3.06 g, 16.4 mmol, 82%).

^{1}H-NMR (400 MHz, DMSO-d_6, 300 K): δ (ppm) = 4.86 (br s, 1H, O*H*), 4.30 (br s, 1H, O*H*), 4.20 (br s, 1H, O*H*), 3.82 (dd, *J* = 9.5, 5.1 Hz, 1H, C*H*), 3.24 (dd, *J* = 55.9, 11.0 Hz, 2H, CH_2OH), 2.31 (dddd, *J* = 13.7, 9.4, 3.6, 2.3 Hz, 1H, CH_2), 2.06 (dtd, *J* = 9.7, 6.0, 2.3 Hz, 1H, CH_2), 1.99 (t, *J* = 5.9 Hz, 1H, C*H*), 1.79 (tdd, *J* = 5.9, 3.6, 2.6 Hz, 1H, C*H*), 1.54 (ddd, *J* = 13.7, 5.1, 2.6 Hz, 1H, CH_2), 1.37 (d, *J* = 9.7 Hz, 1H, CH_2), 1.19 (s, 3H, CH_3), 0.85 (s, 3H, CH_3).

^{13}C-NMR (101 MHz, DMSO-d_6, 300 K): δ (ppm) = 75.1 (C_{quart}), 68.6 (CH$_2$), 63.8 (*C*H), 48.1 (*C*H), 40.2 (*C*H), 38.1 (C_{quart}), 37.8 (CH$_2$), 27.7 (CH$_3$), 27.3 (CH$_2$), 24.1 (CH$_3$).

IR (diamond ATR, neat): $\tilde{\nu}$ (cm^{-1}) = 3385, 2983, 2947, 2920, 2863, 2361, 2161, 2029, 1413, 1383, 1367, 1320, 1269, 1224, 1148, 1125, 1067, 1028, 958, 929, 895, 860, 829, 738, 645.

m.p.: 61.7 °C.

HRMS (ESI+): *m/z* for $C_{10}H_{18}O_3Na$ $[M+Na]^+$: calculated: 209.1148; found: 209.1148.

3.1.4.6 Methyl 5-deoxy-5-fluoro-β,D-ribofuranoside (FDR-OMe) (5)

Methyl 2,3-*O*-isopropylidene-5-*O*-(toluenesulfonyl)-β,D-ribofuranoside (21)

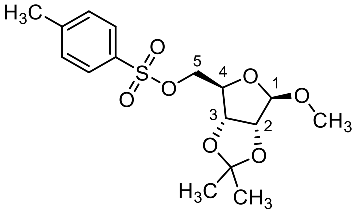

Following a literature procedure[17], methyl 2,3-*O*-isopropylidene-5-*O*-β,D-ribofuranoside (1.50 g, 7.35 mmol, 1.00 equiv.) was dissolved in dry pyridine (4.5 mL) and cooled to 0°C. *p*-Toluenesulfonyl chloride (2.10 g, 11.0 mmol, 1.50 equiv.) was added in portions. The mixture was stirred for 15 min at 0°C and an additional 2 h at room temperature, before being poured into 20 mL of ice water. The white precipitate was collected by filtration, washed with ice water (3 × 10 mL) and dried *in vacuo* to afford tosylate **21** as a white solid (2.56 g, 7.14 mmol, 97%).

^{1}H-NMR (400 MHz, $CDCl_3$, 300 K): δ (ppm) = 7.82 – 7.79 (m, 2H, Ar-*H*), 7.36 (d, *J* = 7.9 Hz, 2H, Ar-*H*), 4.93 (app s, 1H, *H*-C1), 4.60 (dd, *J* = 5.9, 1.0 Hz, 1H, *H*-C3), 4.53 (d, *J* = 5.9 Hz, 1H, *H*-C2), 4.31 (td, *J* = 7.1, 1.0 Hz, 1H, *H*-C4), 4.02 (app dd, *J* = 7.2, 1.9 Hz, 2H, *H*-C5), 3.24 (s, 3H, OCH_3), 2.46 (s, 1H, Ar-CH_3), 1.45 (s, 3H, CH_3), 1.29 (s, 3H, CH_3).

The spectroscopic data are consistent with the literature.[17]

Methyl 2,3-*O*-isopropylidene-5-deoxy-5-fluoro-β,D-ribofuranoside (22)

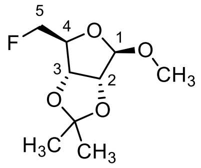

Tosylate **21** (2.00 g, 5.58 mmol, 1.00 equiv.) was dissolved in TBAF solution (20 mL, 1M in THF) under nitrogen atmosphere. The solution was heated to 100°C in a sealed vessel for 15 h. The solvent was evaporated, and the crude oil was purified by flash chromatography (0-10% EtOAc in *n*-heptane) yielding the title compound as a colorless oil (872 mg, 4.23 mmol, 76%).

^{1}H-NMR (400 MHz, $CDCl_3$, 300 K): δ (ppm) = 4.99 (d, *J* = 2.4 Hz, 1H, *H*-C1), 4.70 (d, *J* = 6.0, 1H, *H*-C3), 4.59 (d, *J* = 5.9 Hz, 1H, *H*-C2), 4.47 – 4.27 (m, 3H, *H*-C4 + *H*-C5), 3.33 (s, 3H, OCH_3), 1.49 (s, 3H, CH_3), 1.33 (s, 3H, CH_3).

^{19}F{^{1}H}-NMR (376 MHz, $CDCl_3$, 300 K) δ (ppm) = –225.01.

The spectroscopic data are consistent with the literature.[17]

Methyl 5-deoxy-5-fluoro-β,D-ribofuranoside (FDRibOMe) (5)

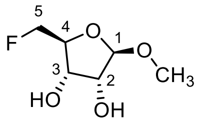

Acetonide **22** (49 mg, 0.238 mmol, 1.00 equiv) was dissolved in a mixture of acetic acid (700 µL) and distilled water (300 µL) and heated at 75°C in a sealed tube for 2 h. The solvent was evaporated and the mixture was purified by flash chromatography (0-10% MeOH in DCM) to yield FDRibOMe (20 mg, 0.12 mmol, 51%) as a colorless oil.

^{1}H-NMR (400 MHz, D_2O, 300 K): δ (ppm) = 4.98 (app s, 1H, *H*-C1), 4.77 – 4.49 (m, 2H, H-C2 + H-C3), 4.31 (dd, *J* = 7.2, 4.6 Hz, 1H), 4.21 (dddd, *J* = 24.8, 7.4, 5.0, 2.5 Hz, 1H), 4.09 (dt, *J* = 4.7, 1.4 Hz, 1H) 3.41 (s, 3H, OCH_3).

^{13}C-NMR (101 MHz, D_2O, 300 K): δ (ppm) = 108.1 (*C*1), 83.5 (d, *J* = 168.5 Hz, *C*5), 80.8 (d, *J* = 18.0 Hz, *C*4), 73.9 (d, *J* = 1.8 Hz, *C*2), 69.6 (d, *J* = 7.2 Hz, *C*3), 55.2 (CH_3).

^{19}F-NMR (376 MHz, D_2O, 300 K) δ (ppm) = –228.21 (td, *J* = 47.2, 25.0 Hz).

The spectroscopic data are consistent with the literature.[18]

3.1.4.7 Synthesis of a Trifluoromethoxy-modified Myrthenoldiol as Chiral Shift Reagent (7)

Trifluoromethoxy-(1*R*)-(−)-myrtenoldiol acetonide (23)

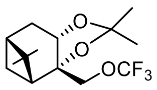

Following a literature procedure,[19] KF (581 mg, 10.0 mmol, 4.00 equiv) was dried by heating under high vacuum. Alcohol **18** (566 mg, 2.50 mmol, 1.00 equiv), AgOTf (1.93 g, 7.50 mmol, 3.00 equiv) and SelectFluor (1.33 g, 3.75 mmol, 1.50 equiv) were added and everything was dissolved in dry EtOAc (12.5 mL, dried over activated molecular sieves 4 Å) under nitrogen atmosphere. $TMSCF_3$ (1.1 mL, 7.50 mmol, 3.00 equiv) and 2-fluoropyridine (0.65 mL, 7.50 mmol, 3.00 equiv) were added and the mixture was stirred at room temperature for one day. The mixture was filtered through a plug of celite. The solvent was evaporated and the crude product was purified by flash chromatography (silica gel, 0−20% EtOAc in petroleum ether) affording the title compound as a colorless oil (260 mg, 0.88 mmol, 35%).

^{1}H-NMR (400 MHz, $CDCl_3$, 295 K): δ (ppm) = 4.22 (d, *J* = 7.1 Hz, 1H, C*H*), 4.11 (s, 2H, CH_2), 2.24 – 2.14 (m, 3H, 2 × CH_2 + C*H*), 2.07 – 1.98 (m, 1H, CH_2), 1.98 – 1.90 (m, 1H, C*H*), 1.75 – 1.65 (m, 1H, CH_2), 1.51 (d, *J* = 0.8 Hz, 3H, CH_3), 1.40 (d, *J* = 0.8 Hz, 3H, CH_3), 1.30 (s, 3H, CH_3), 0.86 (s, 3H, CH_3).

^{13}C-NMR (101 MHz, $CDCl_3$, 295 K): δ (ppm) = 121.8 (q, *J* = 255.0 Hz, *C*F_3), 108.9 (C_{quart}), 85.1 (C_{quart}), 71.9 (*C*H), 71.6 (q, *J* = 2.7 Hz, *C*H_2), 46.1 (*C*H), 40.0 (*C*H), 38.1 (C_{quart}), 32.2 (*C*H_2), 27.7 (*C*H_3), 27.2 (*C*H_3), 26.5 (*C*H_3), 24.6 (*C*H_2), 23.3 (*C*H_3).

^{19}F-NMR (376 MHz, $CDCl_3$, 295 K): δ (ppm) = −60.60 (OCF_3).

IR (diamond ATR, neat): $\tilde{\nu}$ (cm^{-1}) = 2988, 2941, 1460, 1405, 1382, 1370, 1269, 1211, 1132, 1055, 1028, 1000, 979, 926, 895, 866, 848, 822, 765, 633, 616.

Trifluoromethoxy-(1*R*)-(−)-myrtenoldiol (7)

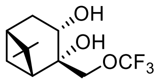

To a solution of acetonide **23** (200 mg, 0.68 mmol, 1.00 equiv) in ethanol (3 mL) and water (3 mL) was added Amberlyst 15 strong cation exchange resin (300 mg). The mixture was refluxed for four days, filtered through a syringe filter (pore size 0.45 µm) and purified by flash chromatography (silica gel, 0-20% EtOAc in petroleum ether). The resulting oil was dried *in vacuo* for a week to yield the title compound as white needles (145 mg, 0.57 mmol, 84%).

^{1}H-NMR (400 MHz, DMSO-d_6, 295 K): δ (ppm) = 5.38 (d, J = 6.2 Hz, 1H, OH), 4.59 (s, 1H, OH), 3.95 – 3.78 (m, 3H, C*H* + CH_2), 2.43 – 2.31 (m, 1H, CH_2), 2.13 (dtd, J = 9.9, 6.1, 2.3 Hz, 1H, CH_2), 2.06 (t, J = 5.9 Hz, 1H, C*H*), 1.84 (tdd, J = 5.9, 3.6, 2.6 Hz, 1H, C*H*), 1.59 (ddd, J = 13.8, 5.0, 2.5 Hz, 1H, CH_2), 1.38 (d, J = 9.9 Hz, 1H, CH_2), 1.22 (s, 3H, CH_3), 0.89 (s, 3H, CH_3).

^{13}C-NMR (101 MHz, DMSO-d_6, 295 K): δ (ppm) = 121.6 (q, J = 252.5 Hz, *C*F_3), 73.7 (q, J = 2.2 Hz, *C*H_2), 73.0 (C_{quart}), 62.8 (*C*H), 47.5 (*C*H), 39.9 (*C*H), 37.9 (C_{quart}), 37.4 (*C*H_2), 27.5 (*C*H_2), 27.0 (*C*H_3), 23.6 (*C*H_3).

^{19}F-NMR (376 MHz, DMSO-d_6, 295 K): δ (ppm) = −58.47 (OCF_3).

IR (diamond ATR, neat): $\tilde{\nu}$ (cm^{-1}) = 3266, 2993, 2917, 2876, 1457, 1411, 1256, 1218, 1132, 1064, 1037, 1015, 934, 906, 875, 827, 698, 611.

m.p.: 34−37°C.

HRMS (ESI+): *m/z* for $C_{11}H_{17}O_3F_3Na$ [M+Na]$^+$: calculated: 277.1022; found: 277.1024.

3.1.5 NMR Spectra

1 (^{1}H and ^{13}C NMR, DMSO-*d6*)

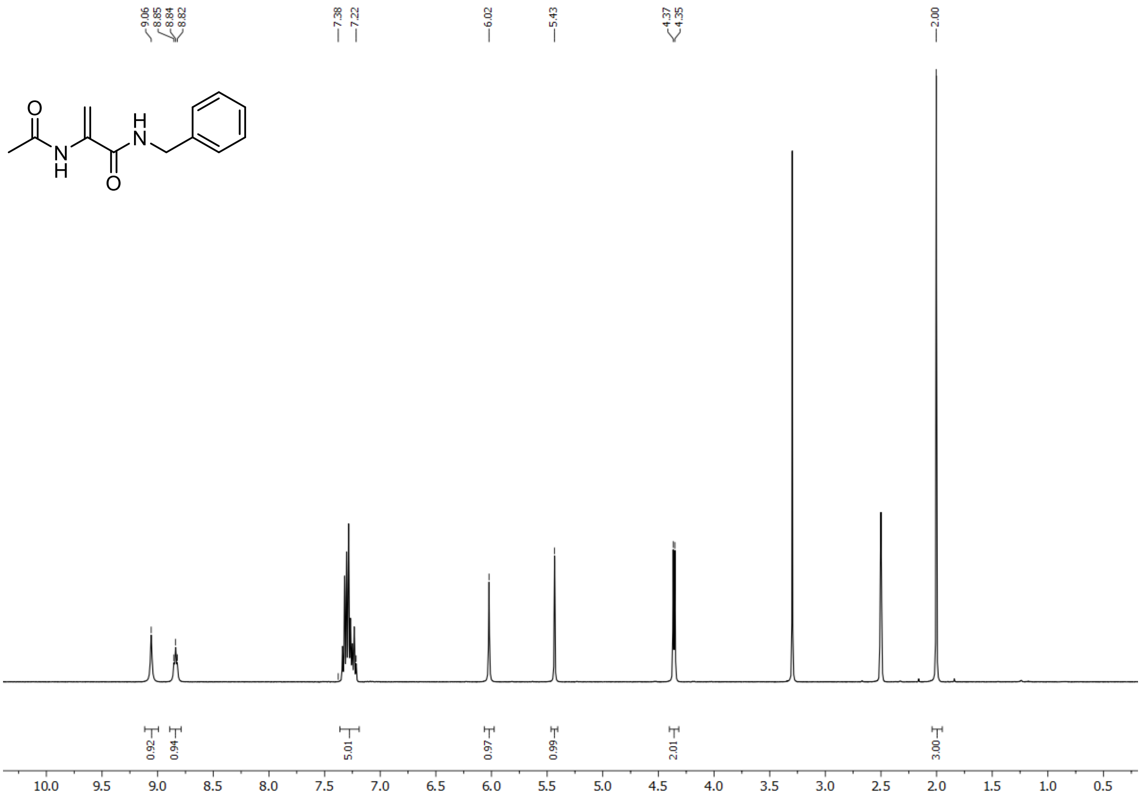

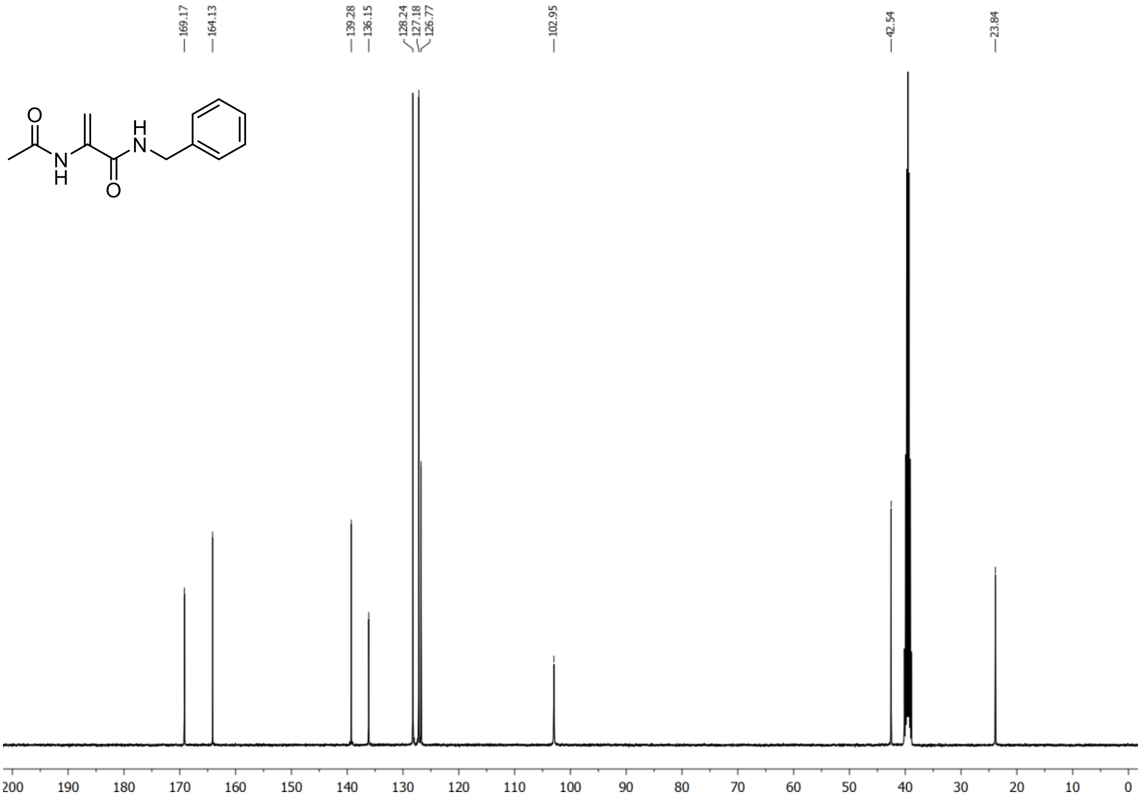

2 (^{1}H, ^{13}C and ^{11}B NMR, NaP_i (pH 1.5) in D_2O)

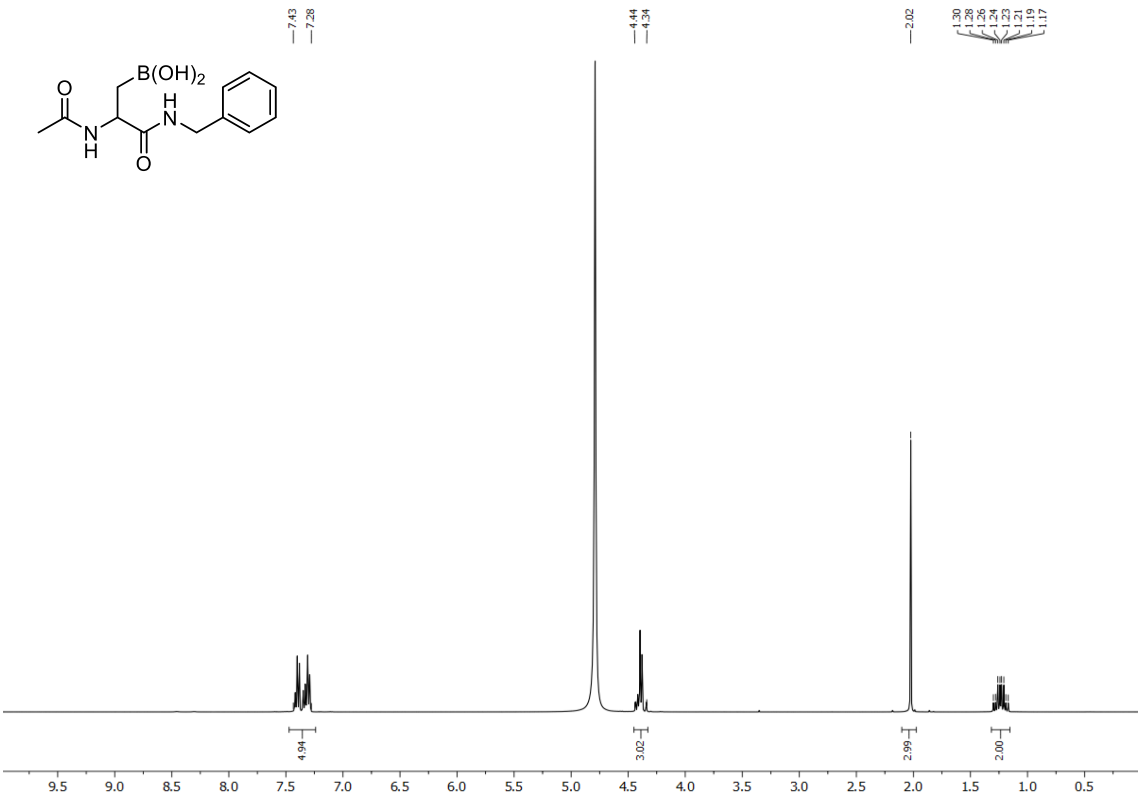

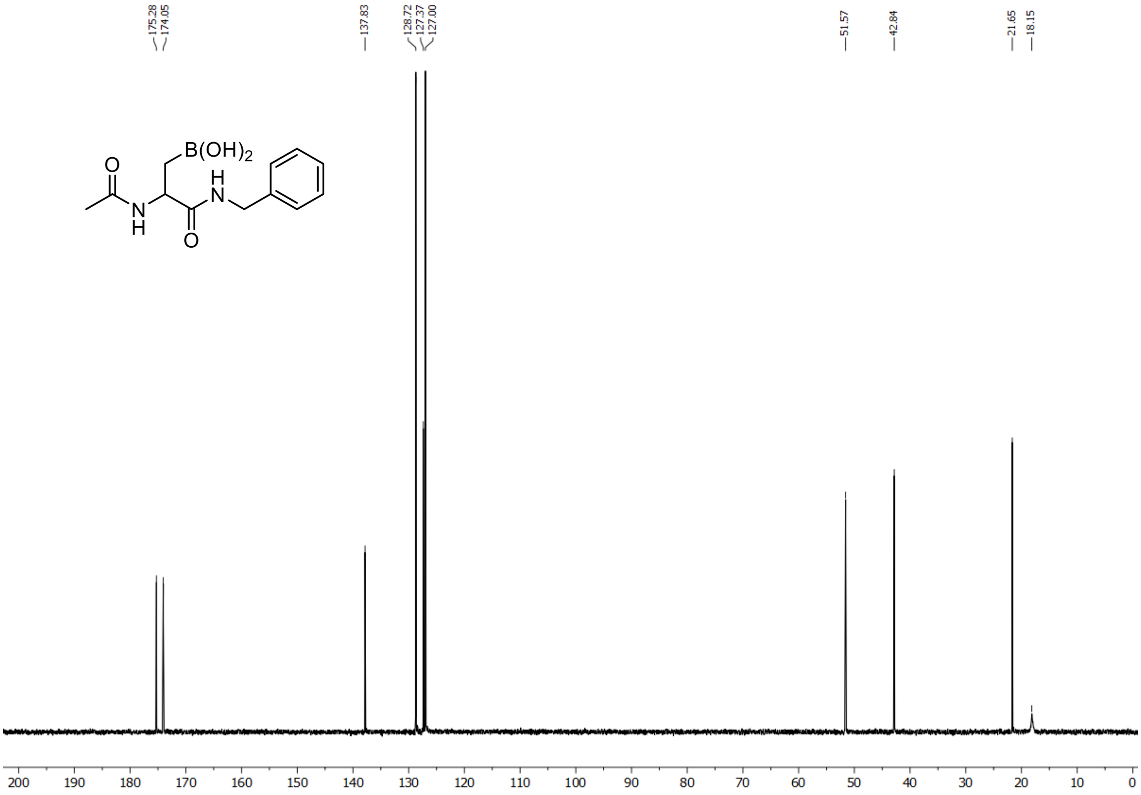

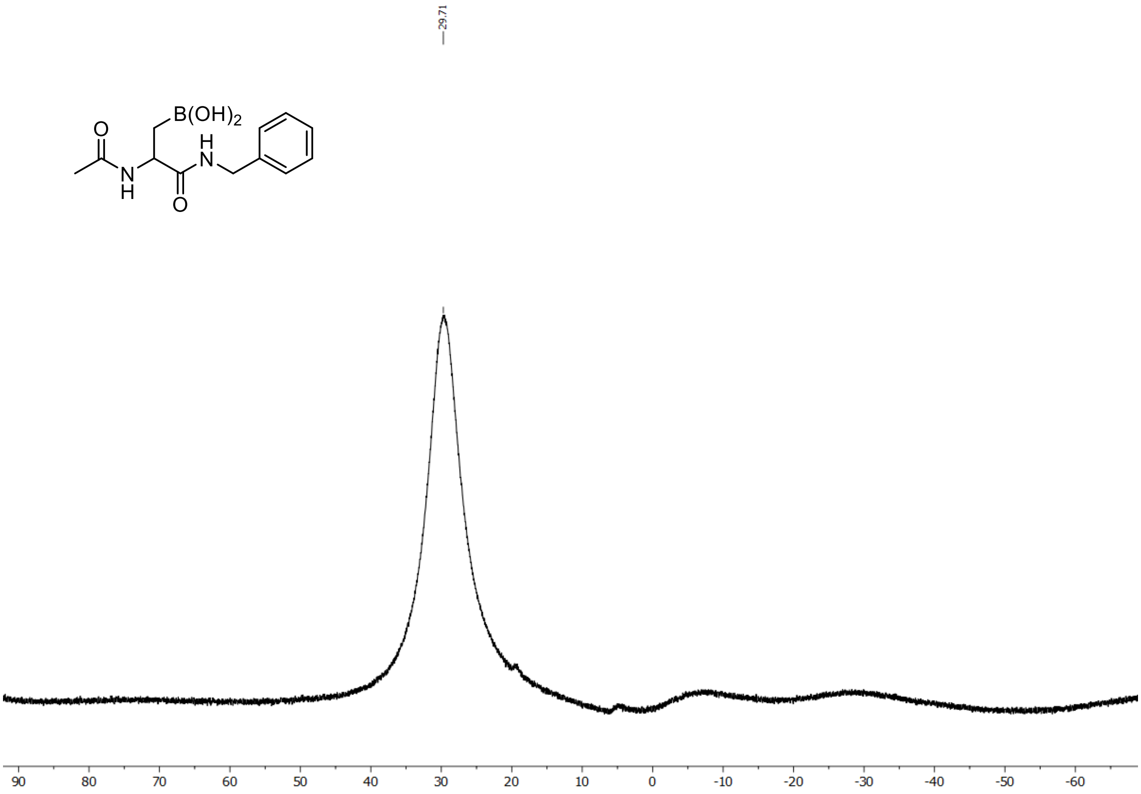
29.71
90 80 70 60 50 40 30 20 10 0 -10 -20 -30 -40 -50 -60

12 (^{1}H and ^{13}C-NMR, $CDCl_3$)

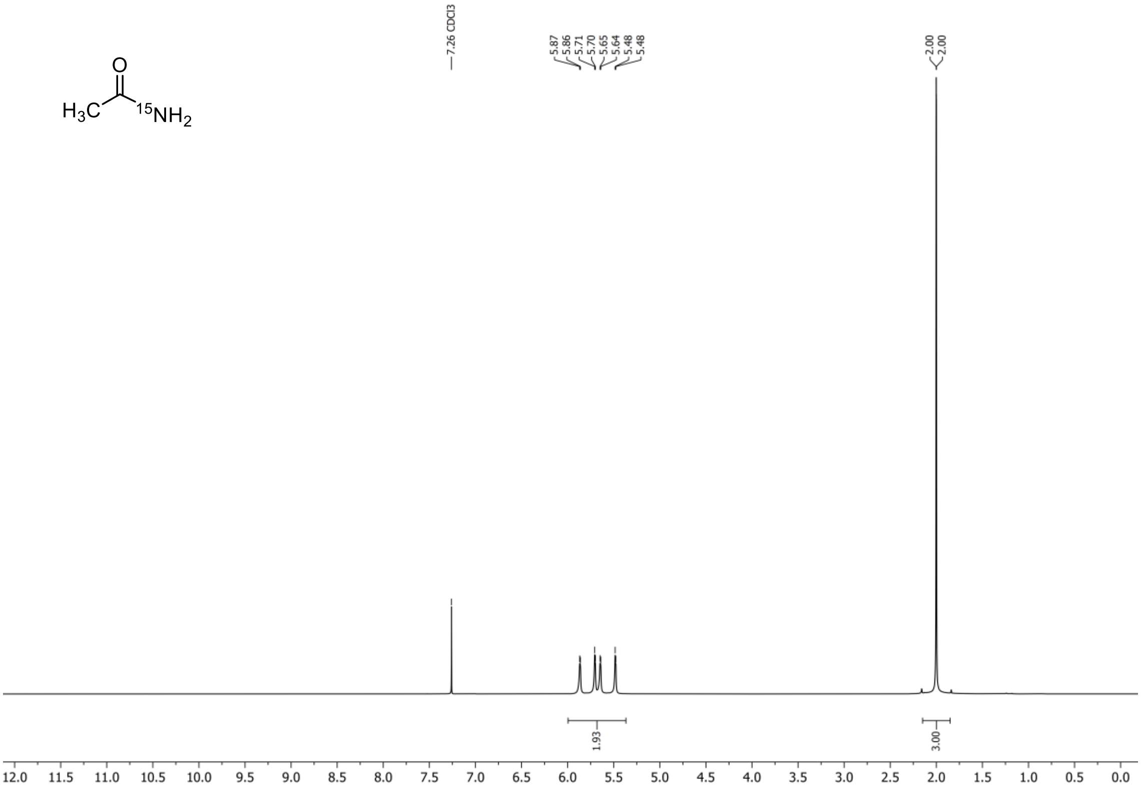

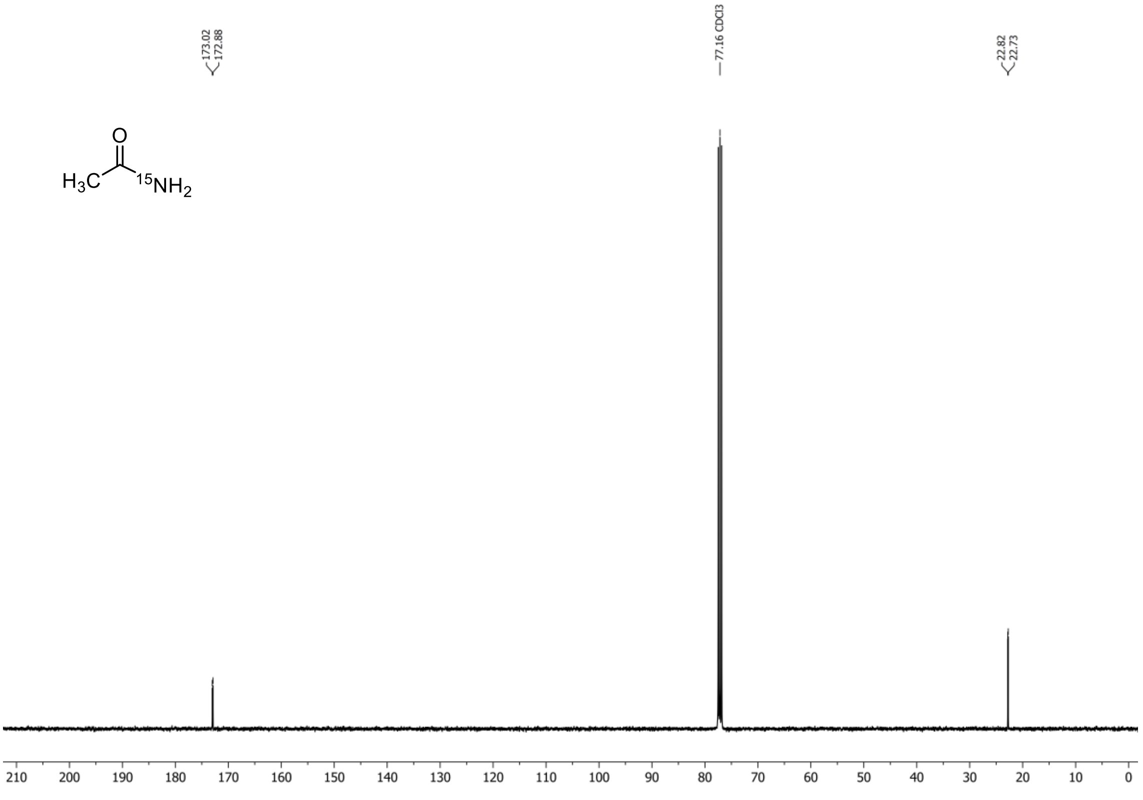

13 (^{1}H and ^{13}C-NMR, DMSO-*d6*)

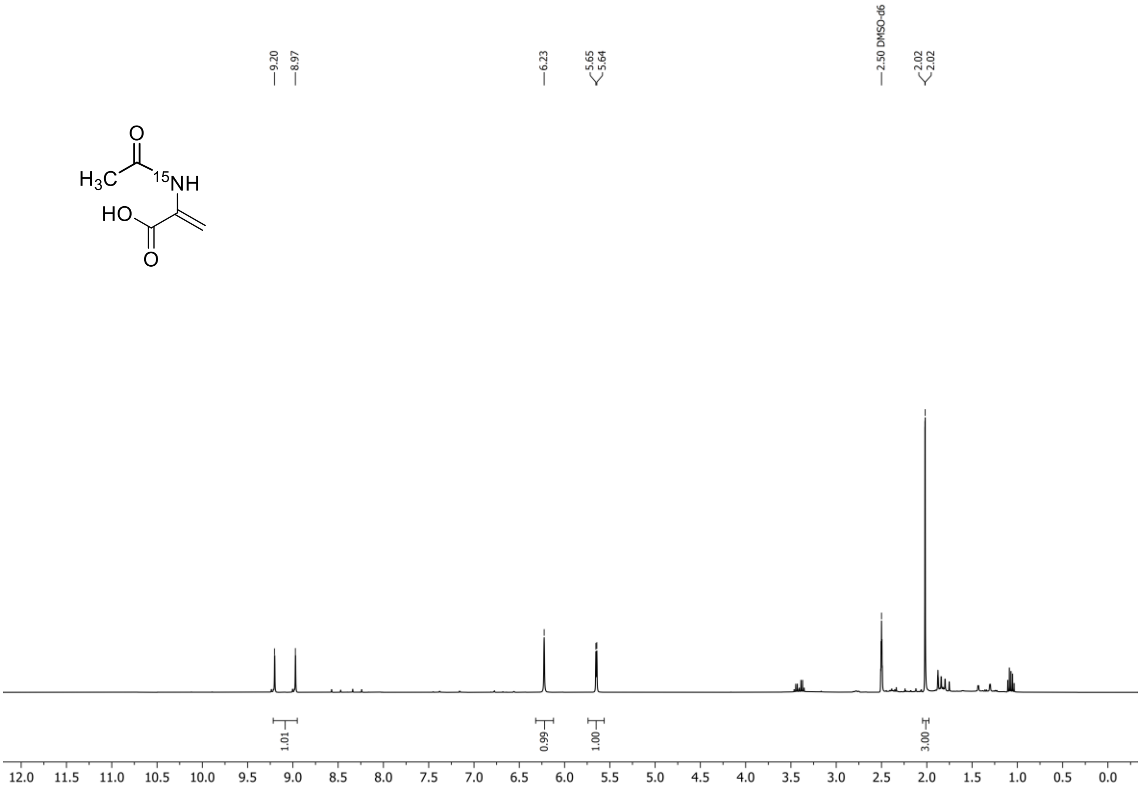

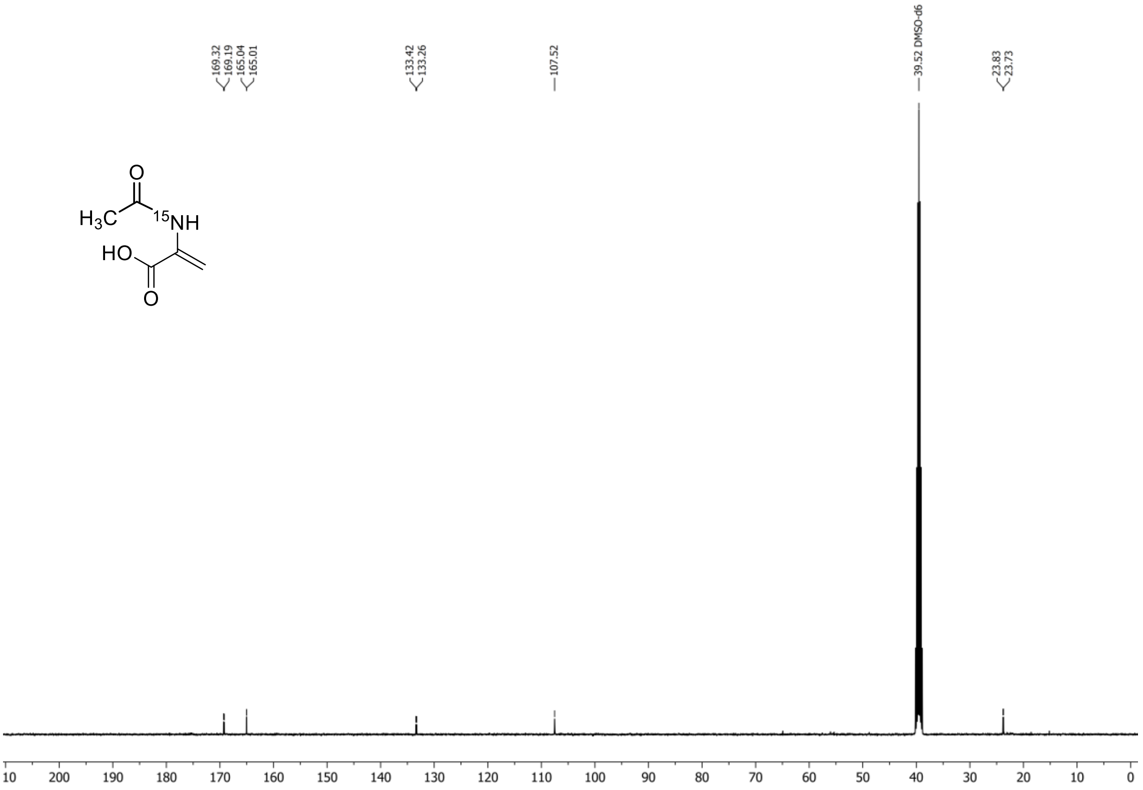

14 (^{1}H and ^{13}C-NMR, DMSO-*d6*)

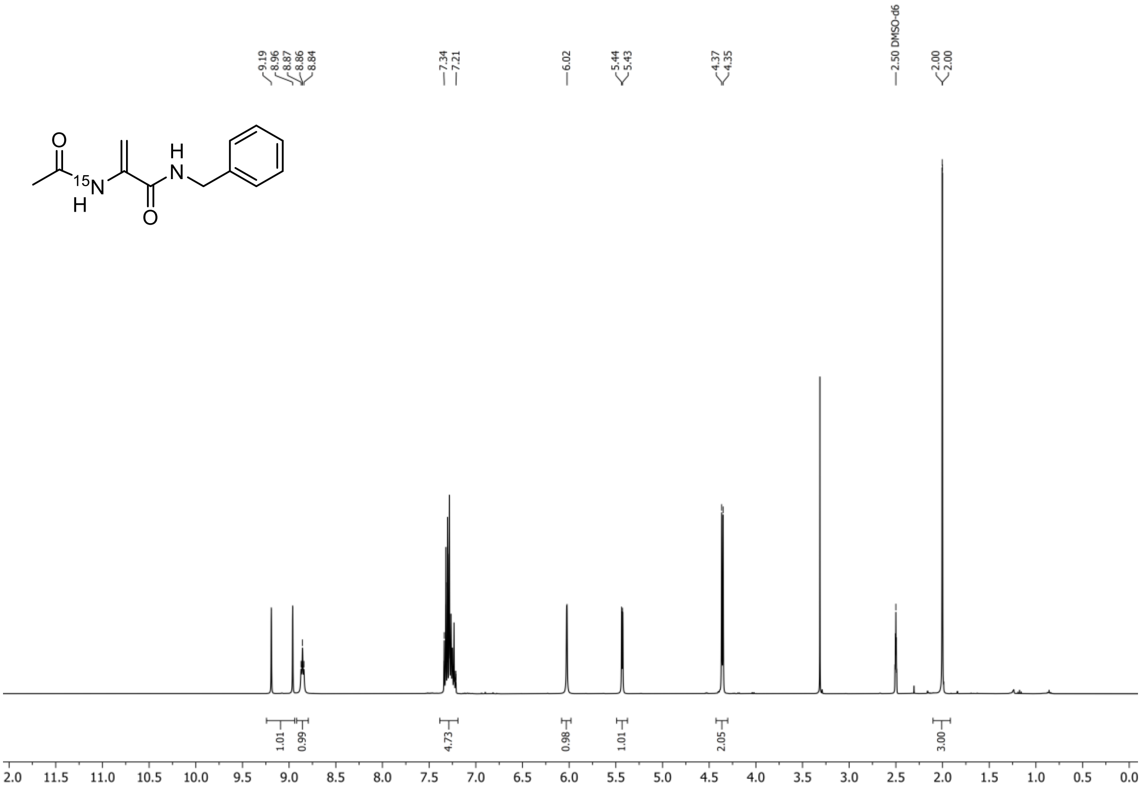

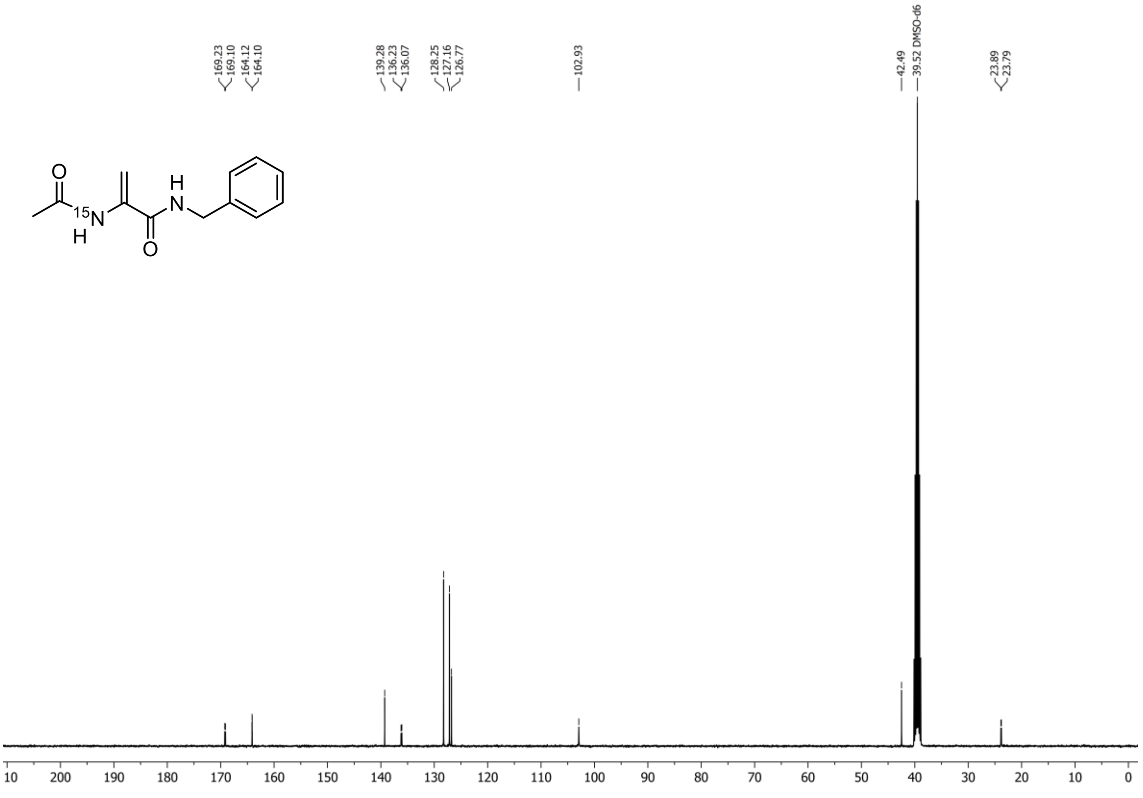

[^{15}N]-Ac-Bal-NHBn (^{1}H, ^{13}C and ^{11}B NMR, D_2O + 5% conc. DCl) **(15)**

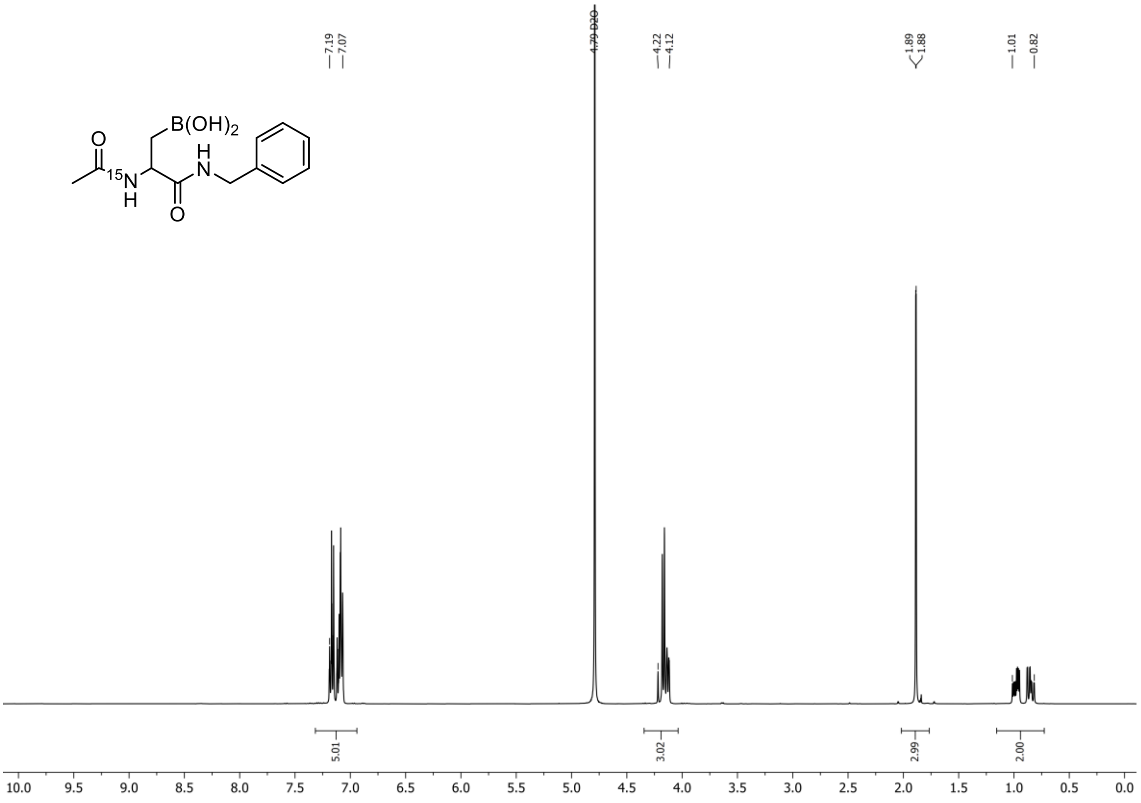

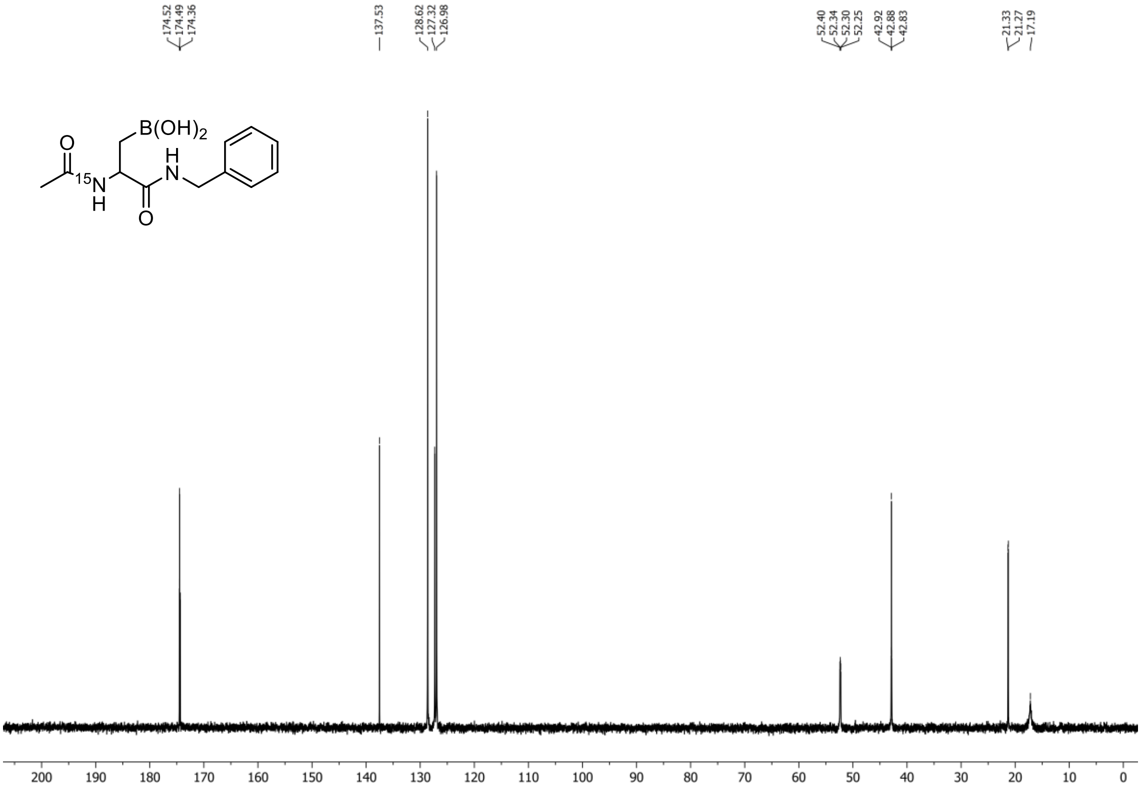

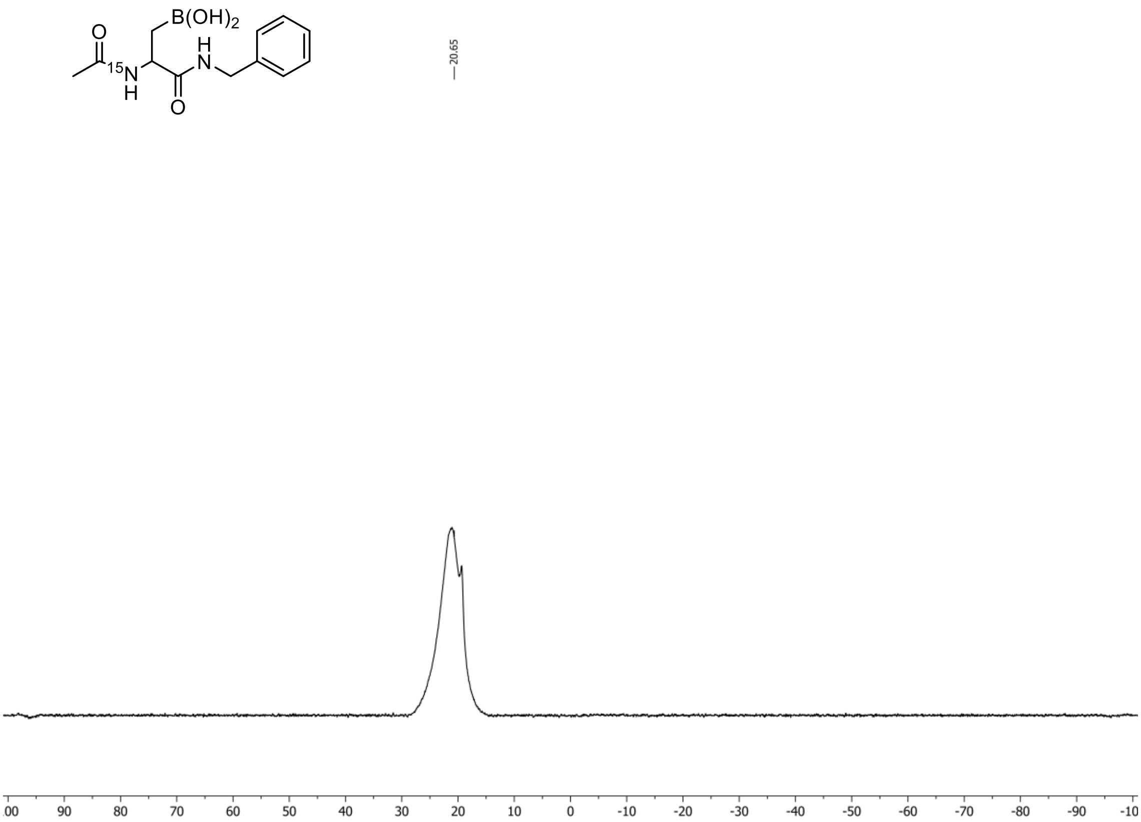
B(OH)2
O
15N
H
H
N
O
20.65
00
90
80
70
60
50
40
30
20
10
0
-10
-20
-30
-40
-50
-60
-70
-80
-90
-10

8 (^{1}H and ^{13}C-NMR, $CDCl_3$)

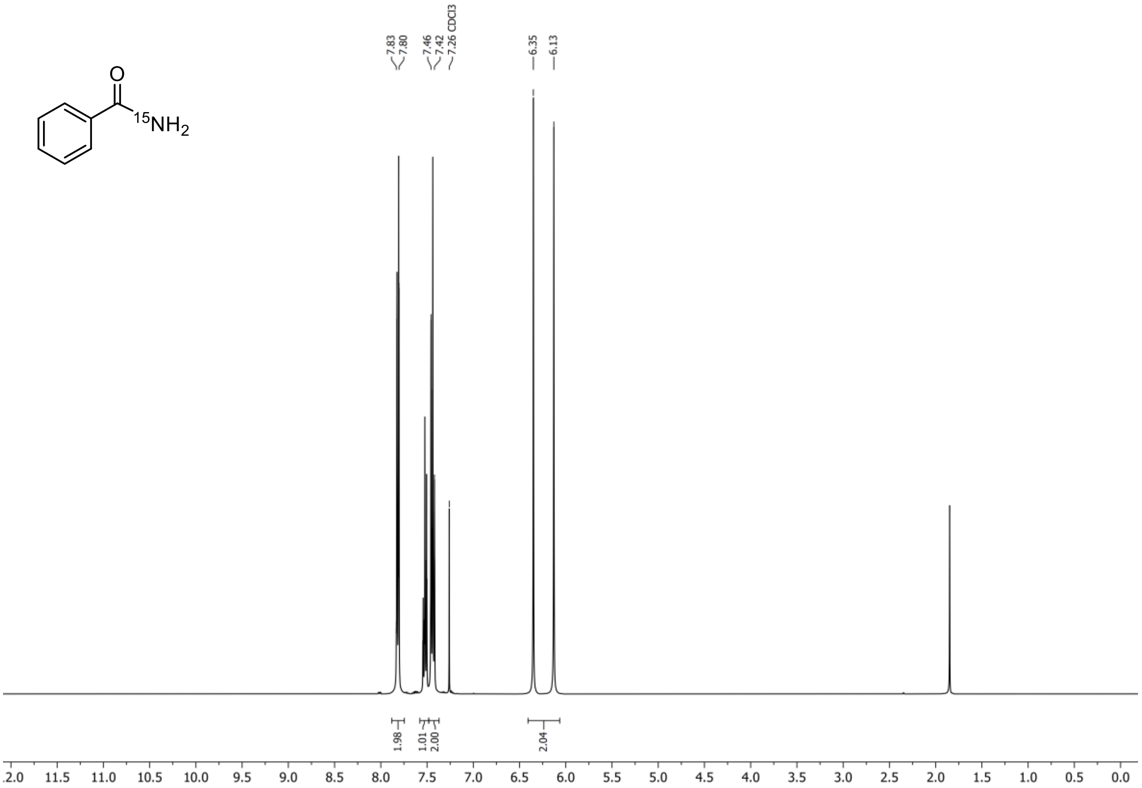

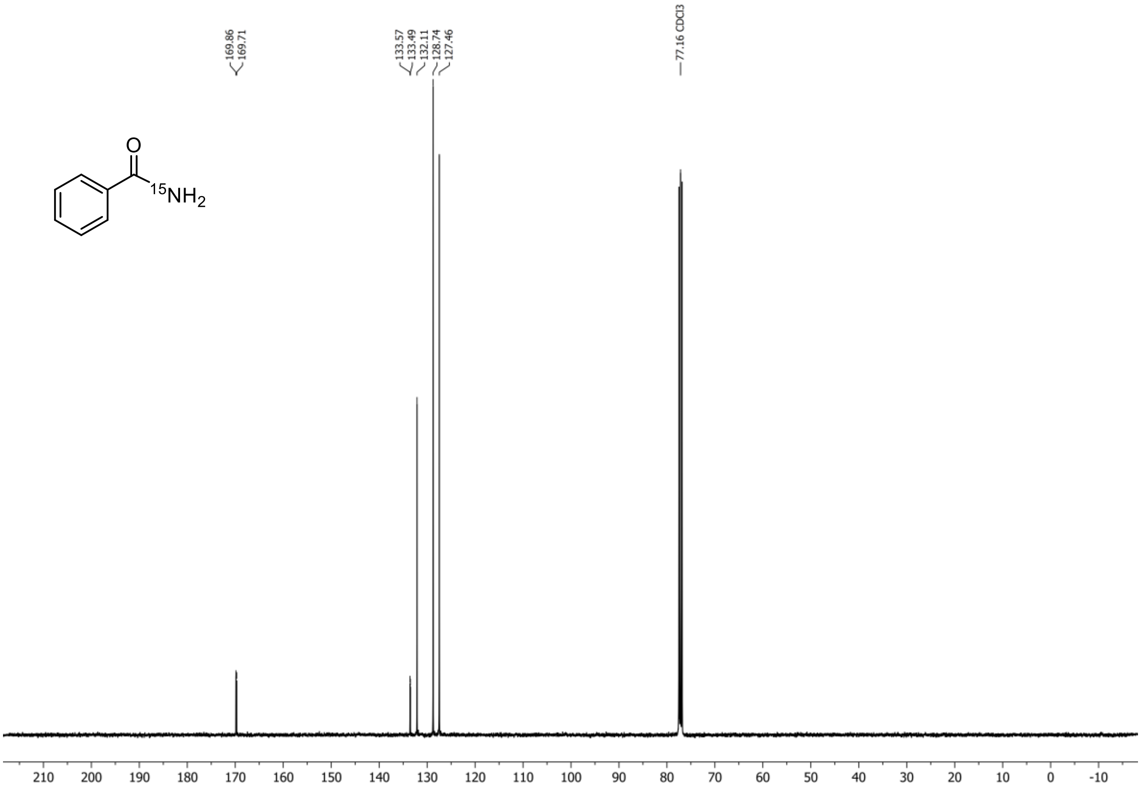

9 (^{1}H and ^{13}C-NMR, $CDCl_3$)

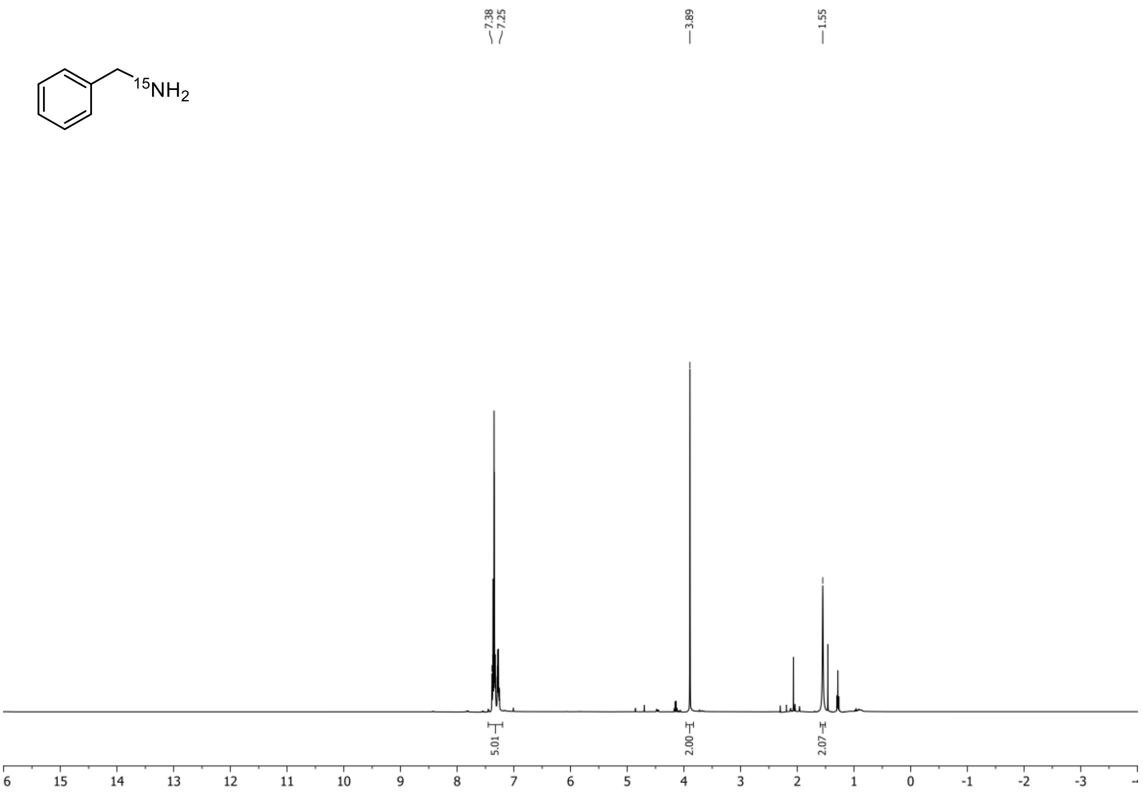

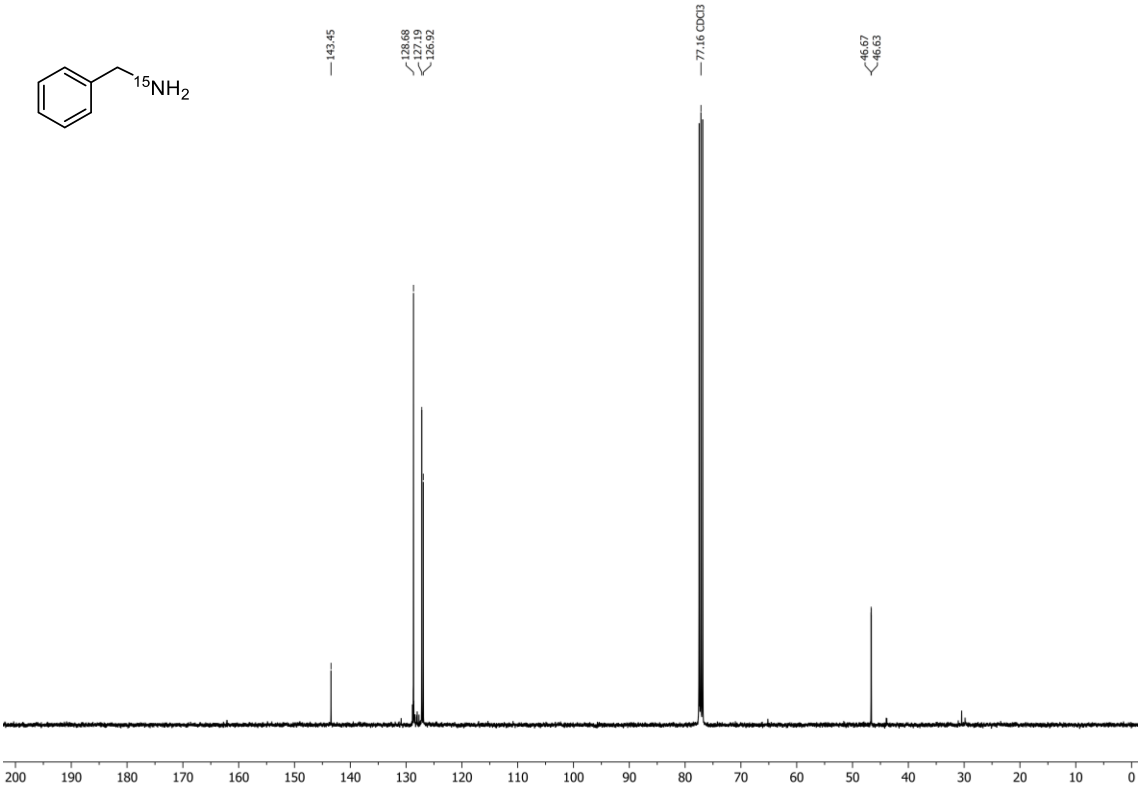

10 (^{1}H and ^{13}C-NMR, DMSO-*d6*)

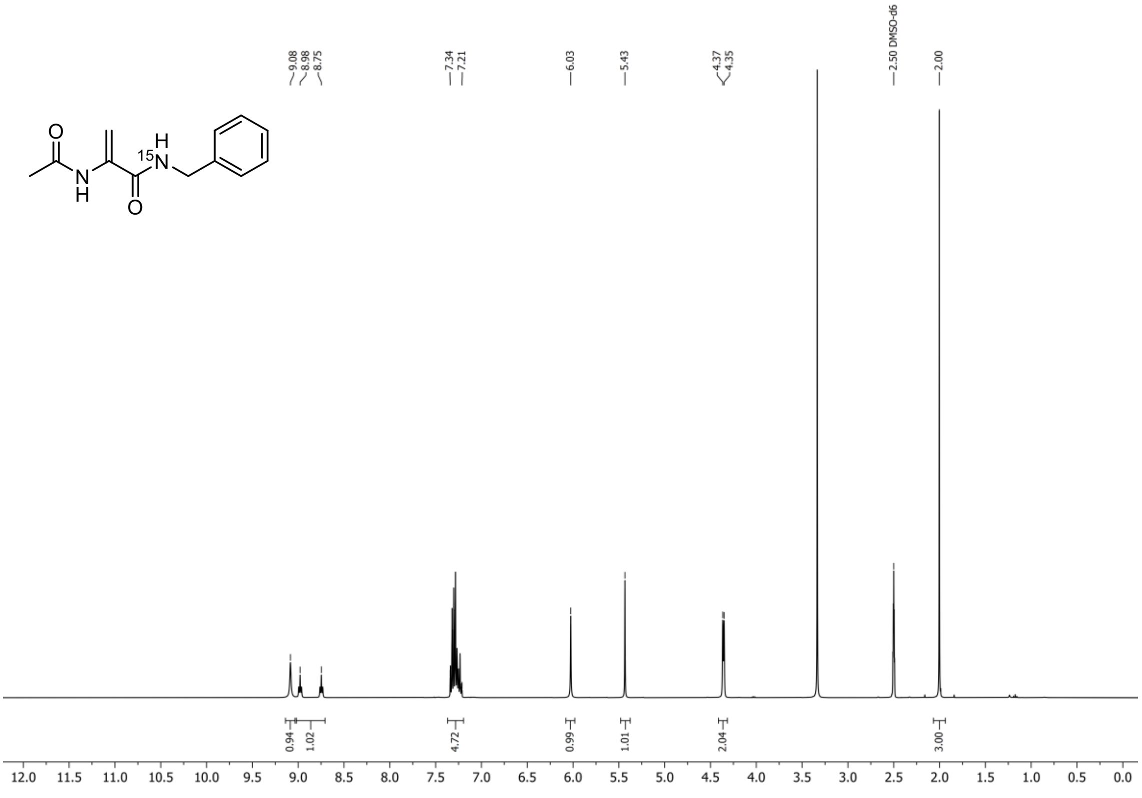

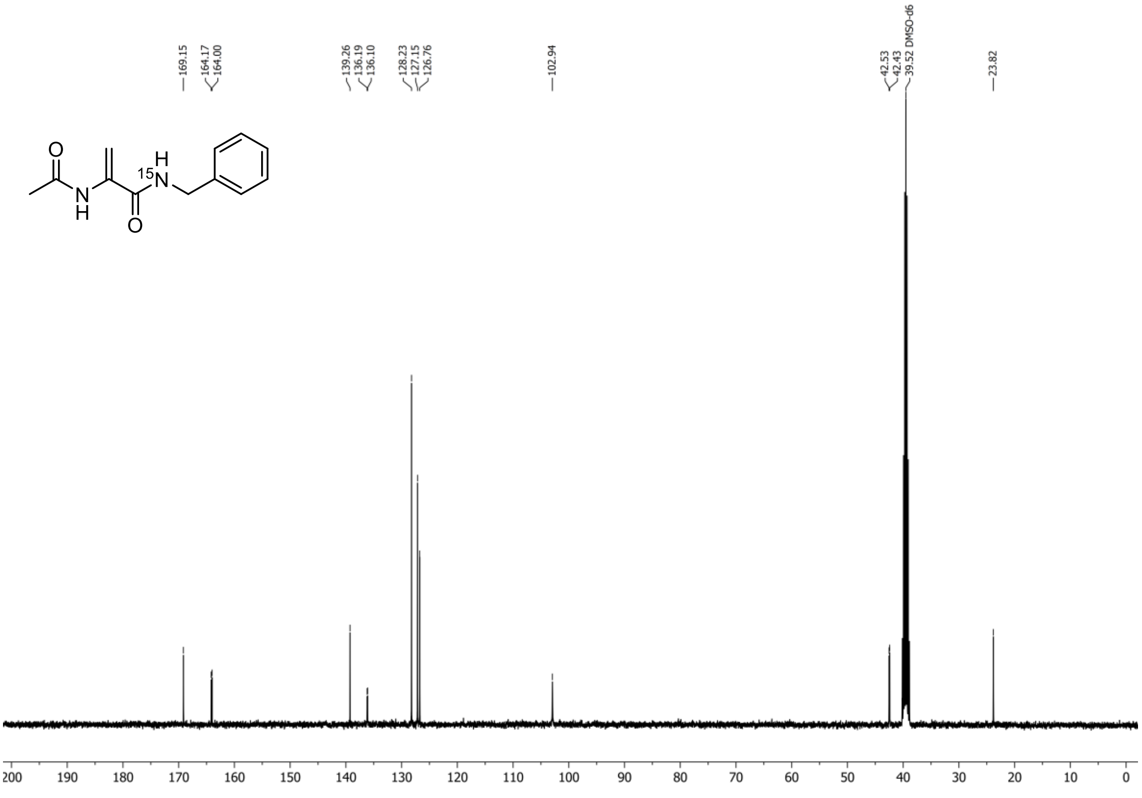

Ac-Bal-[15N]-NHBn (^{1}H, ^{13}C and ^{11}B NMR, D_2O + 5% conc. DCl) **(11)**

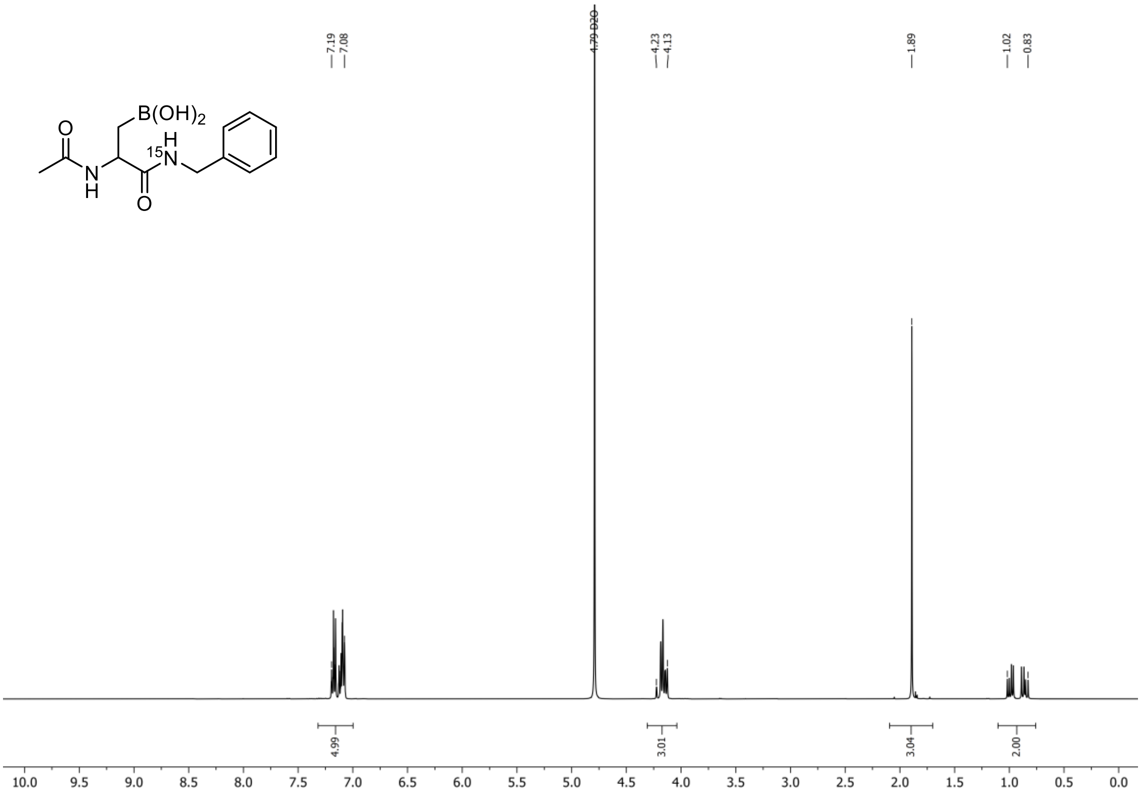

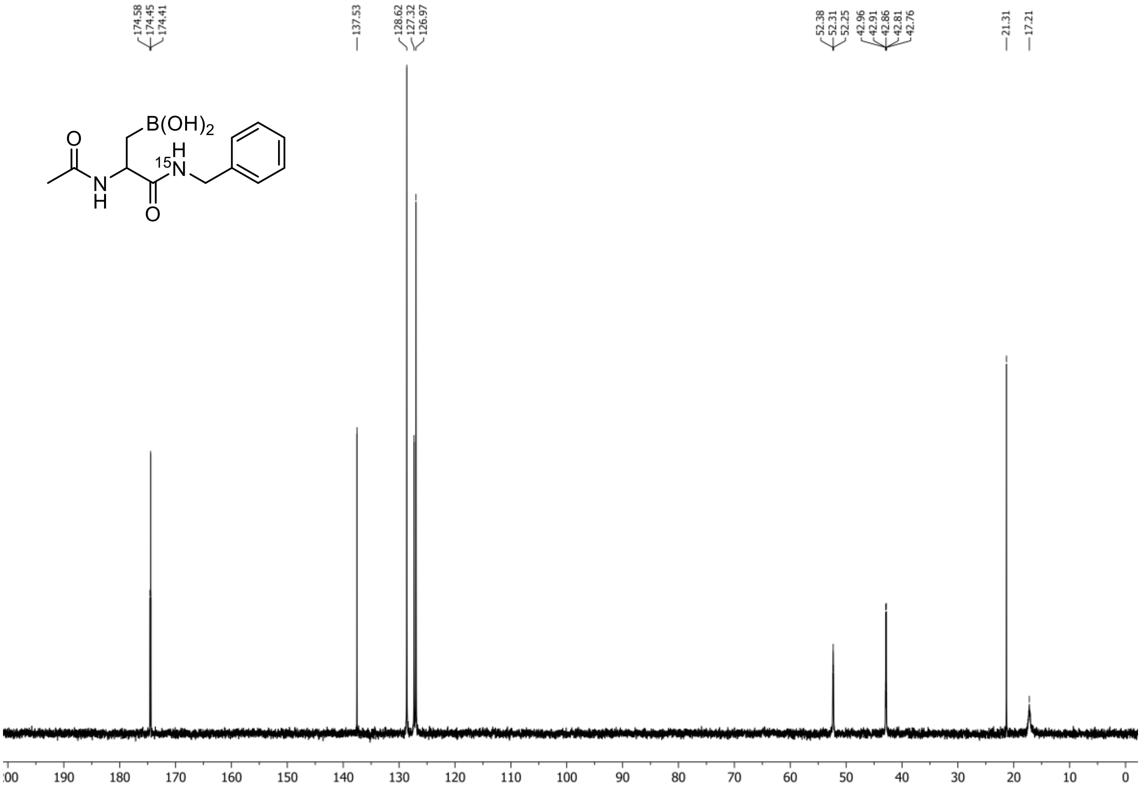

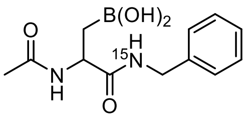
B(OH)2
O
15
H
N
N
H
O

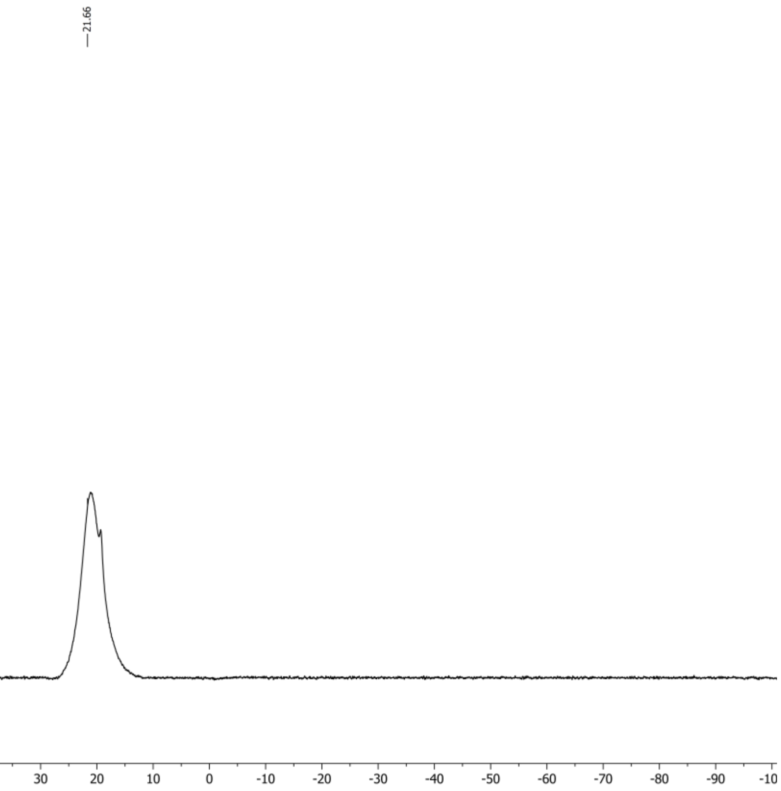
21.66
00
90
80
70
60
50
40
30
20
10
0
-10
-20
-30
-40
-50
-60
-70
-80
-90
-10

16 (^{1}H and ^{13}C NMR, DMSO-*d6*)

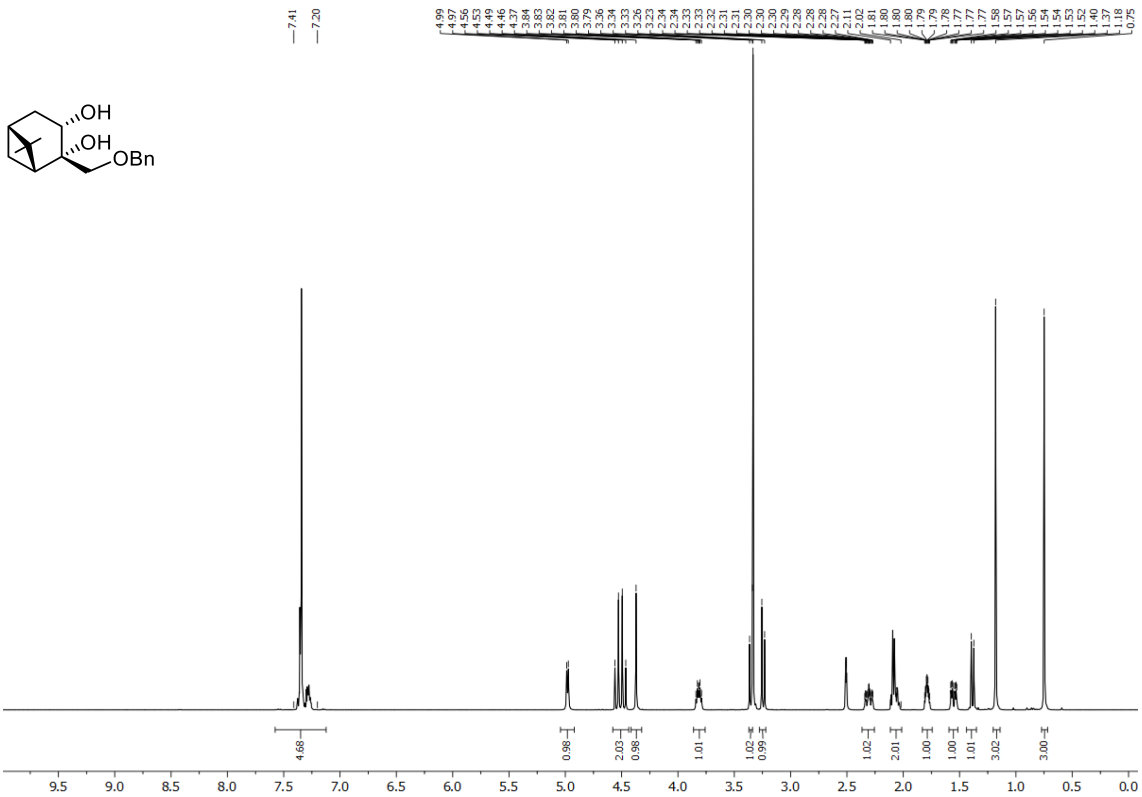

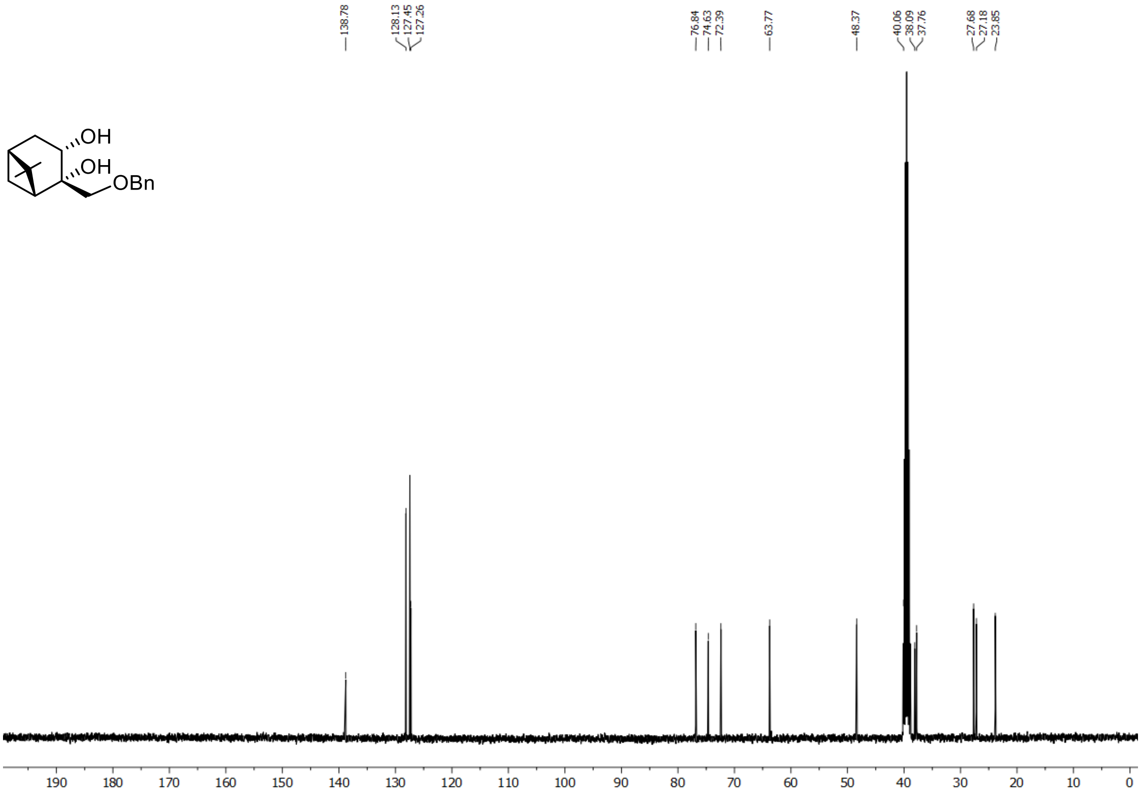

17 (^{1}H and ^{13}C NMR, DMSO-*d6*)

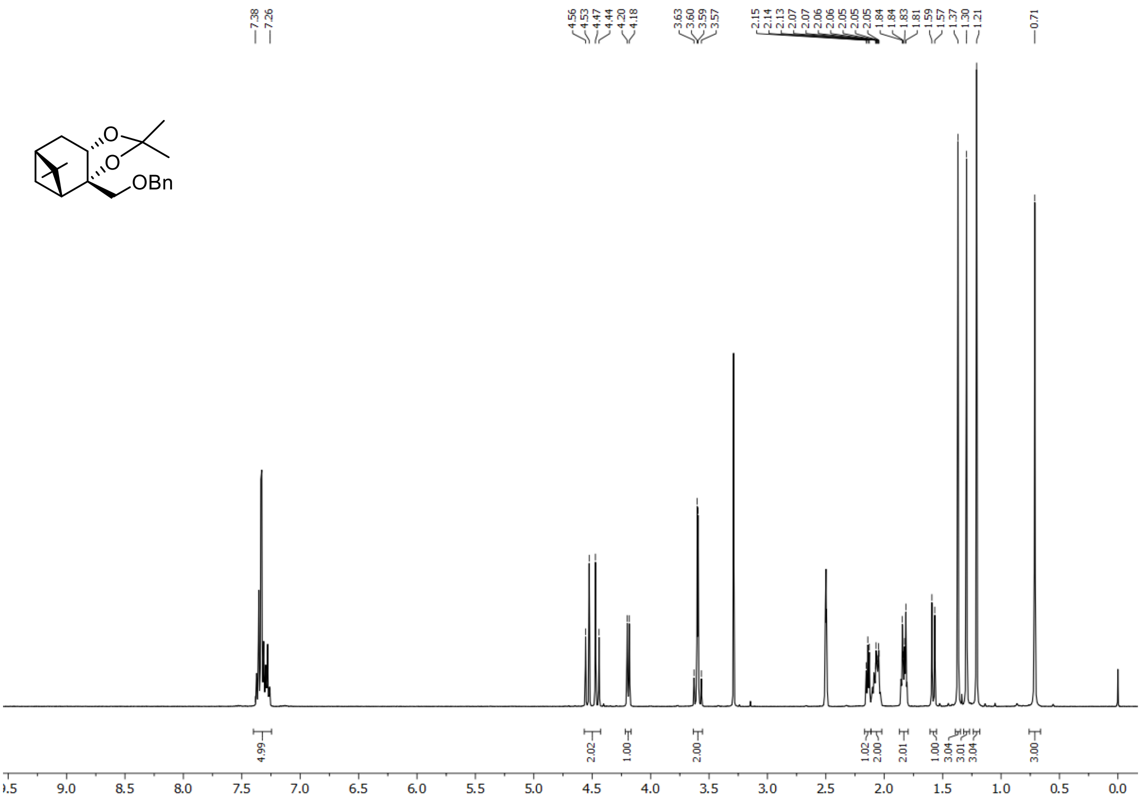

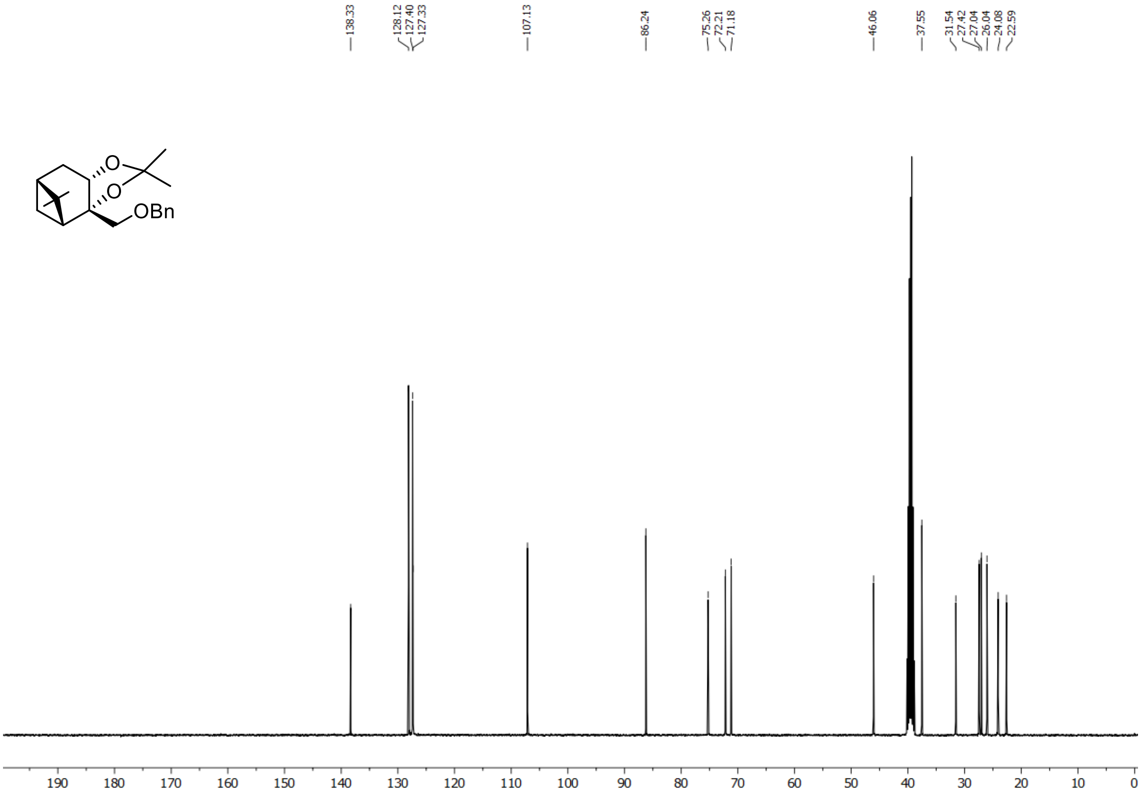

18 (^{1}H and ^{13}C NMR, DMSO-*d6*)

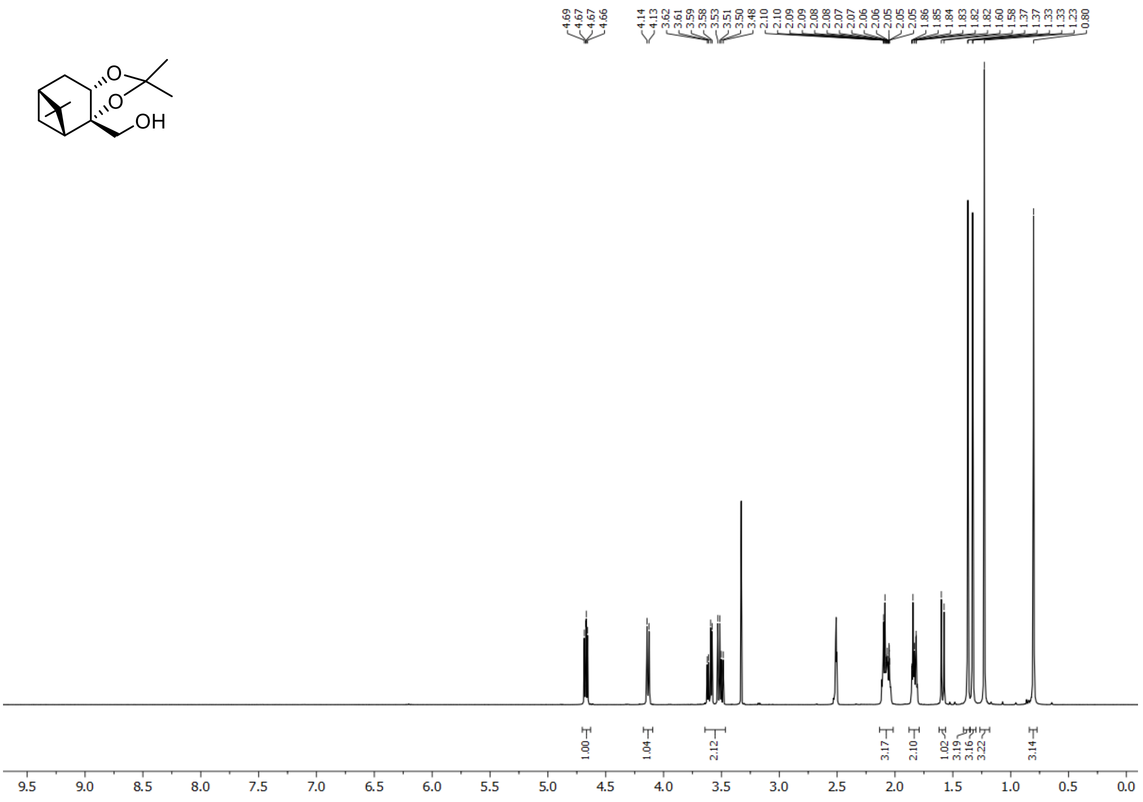

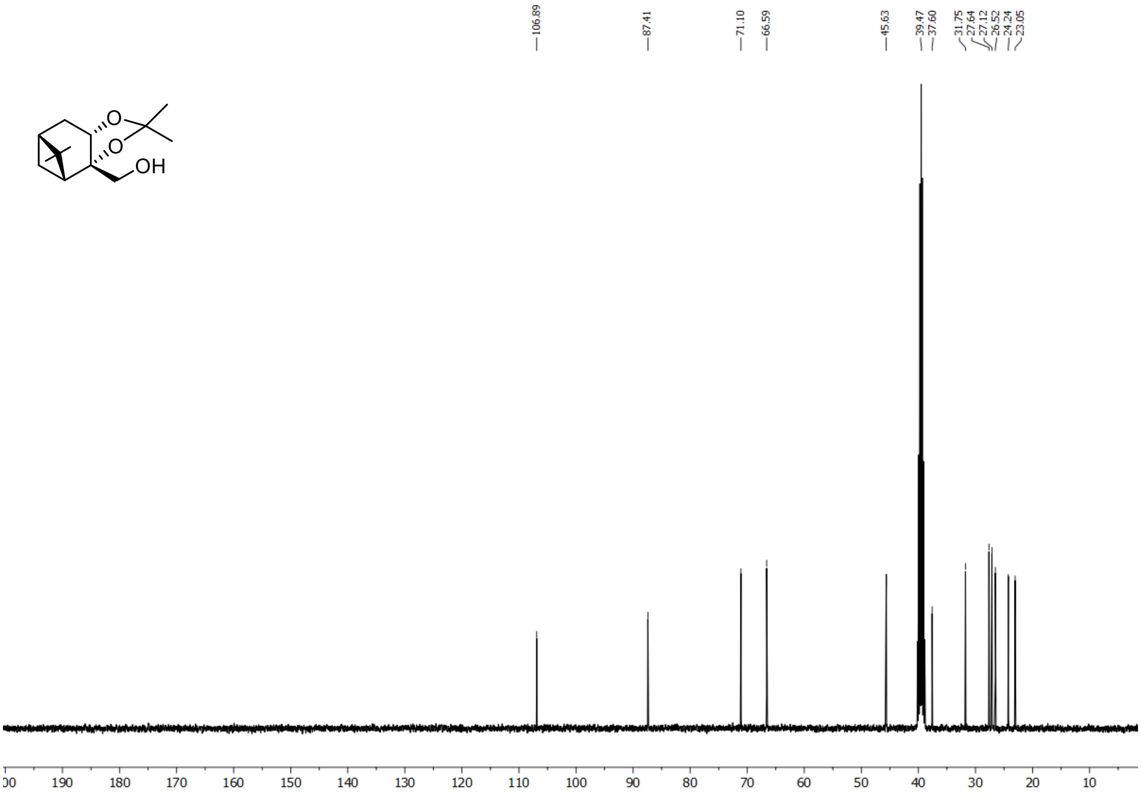

19 (^{1}H, ^{19}F and ^{13}C NMR, $CDCl_3$)

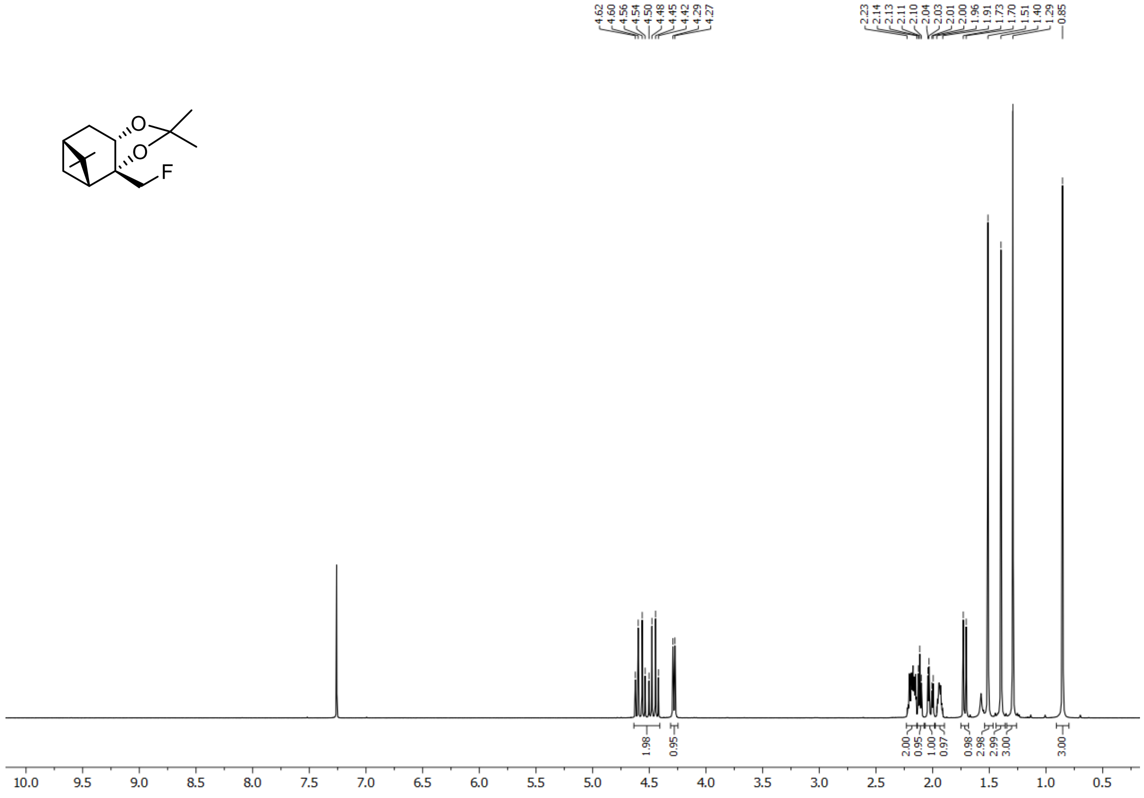

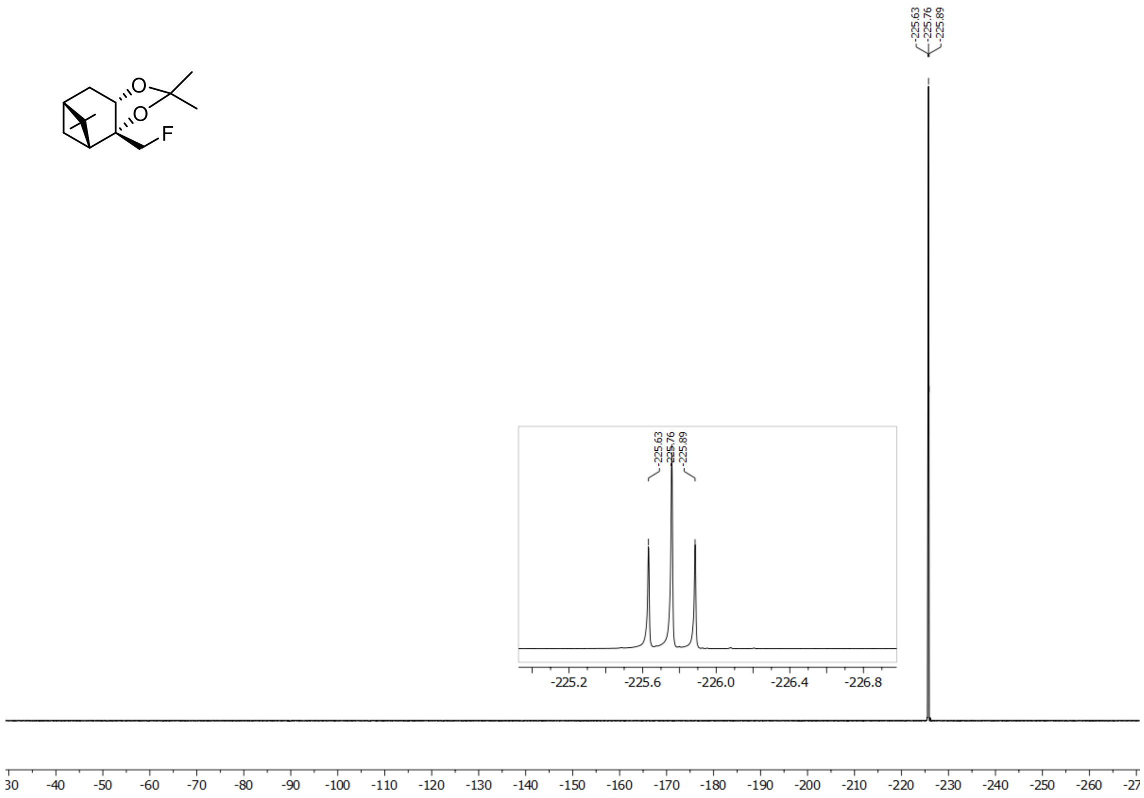

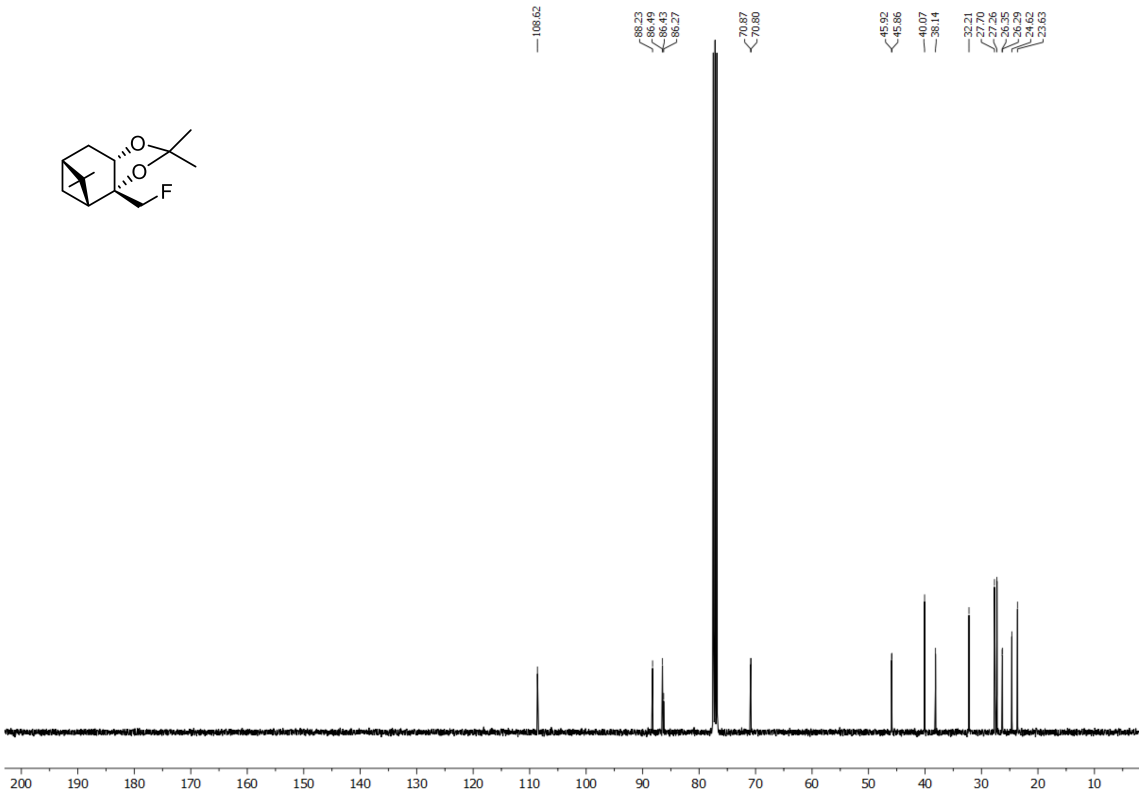
108.62
88.23
86.49
86.43
86.27
70.87
70.80
45.92
45.86
40.07
38.14
32.21
27.70
27.26
26.35
26.29
24.62
23.63
O
O
F
200
190
180
170
160
150
140
130
120
110
100
90
80
70
60
50
40
30
20
10

4 (^{1}H, ^{19}F and ^{13}C NMR, DMSO-*d6*)

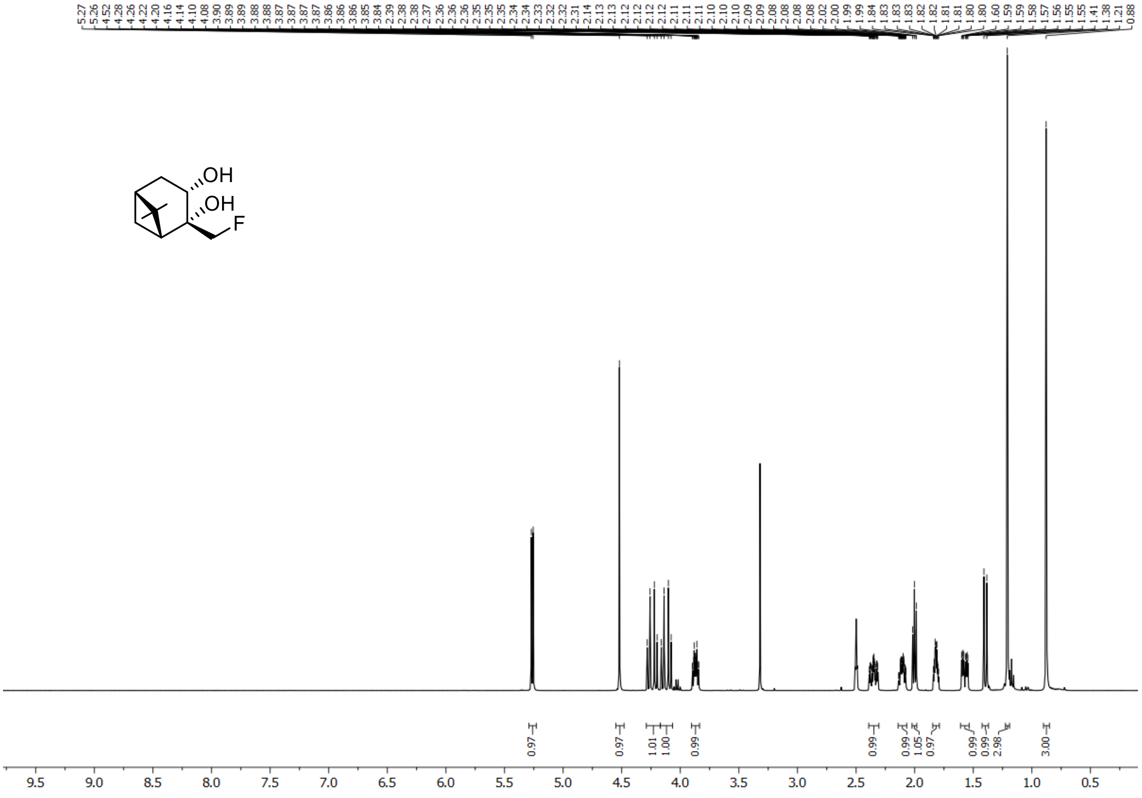

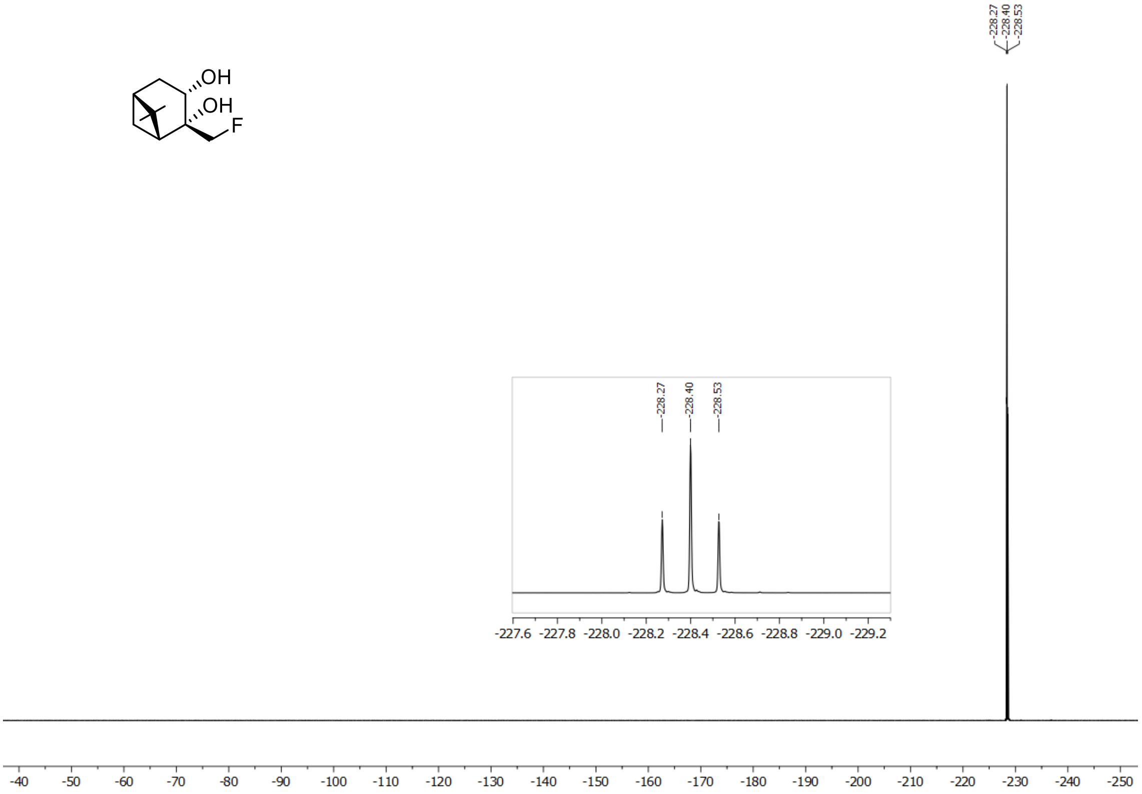

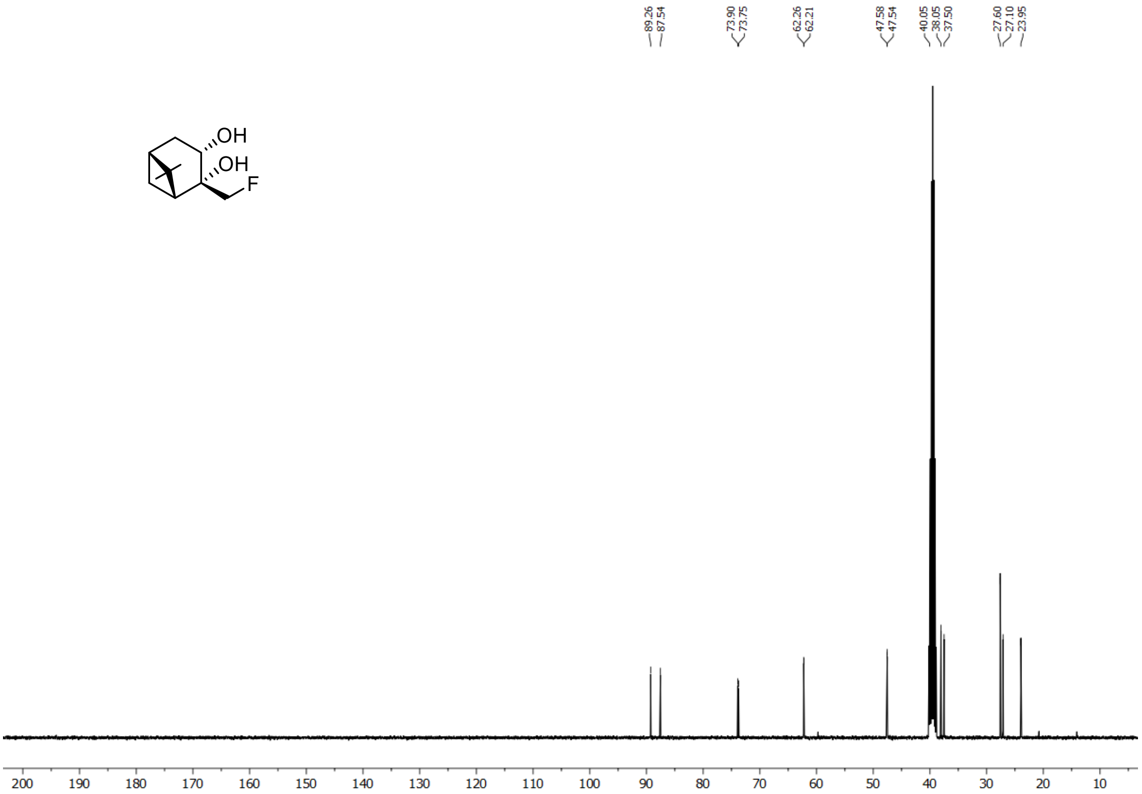
89.26
87.54
73.90
73.75
62.26
62.21
47.58
47.54
40.05
38.05
37.50
27.60
27.10
23.95
OH
OH
F
200
190
180
170
160
150
140
130
120
110
100
90
80
70
60
50
40
30
20
10

20 (^{1}H and ^{19}F, $CDCl_3$)

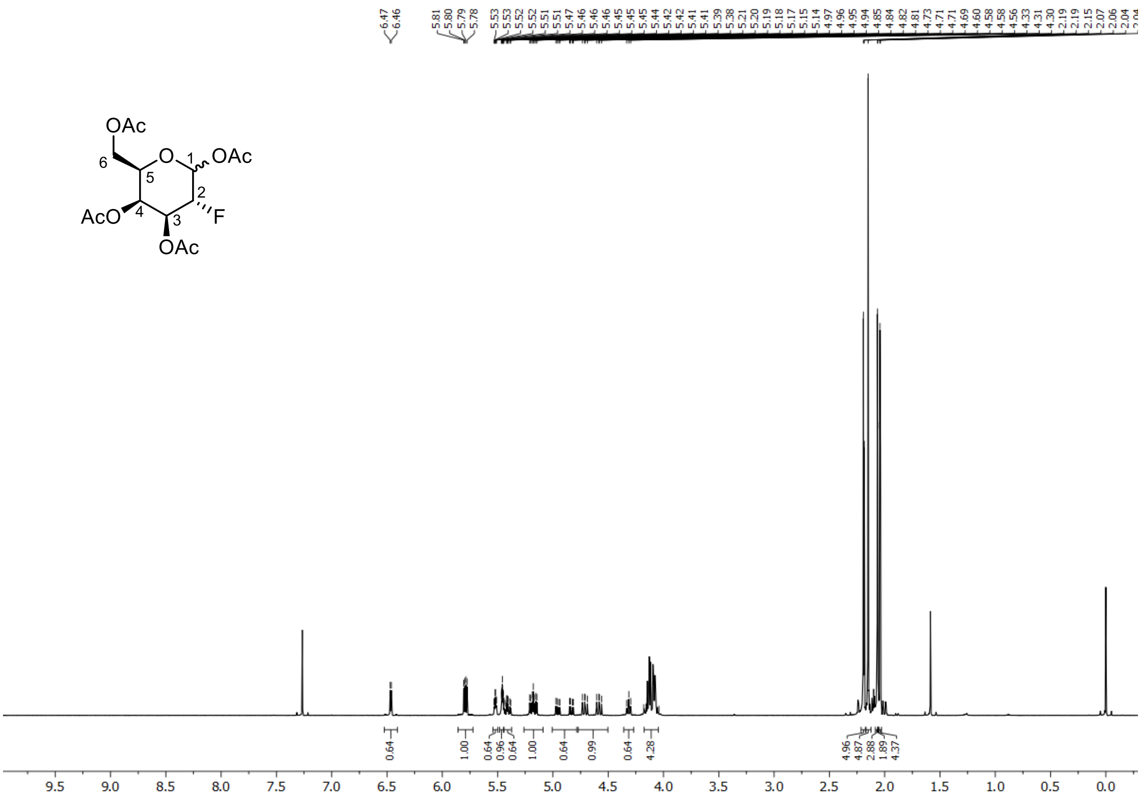

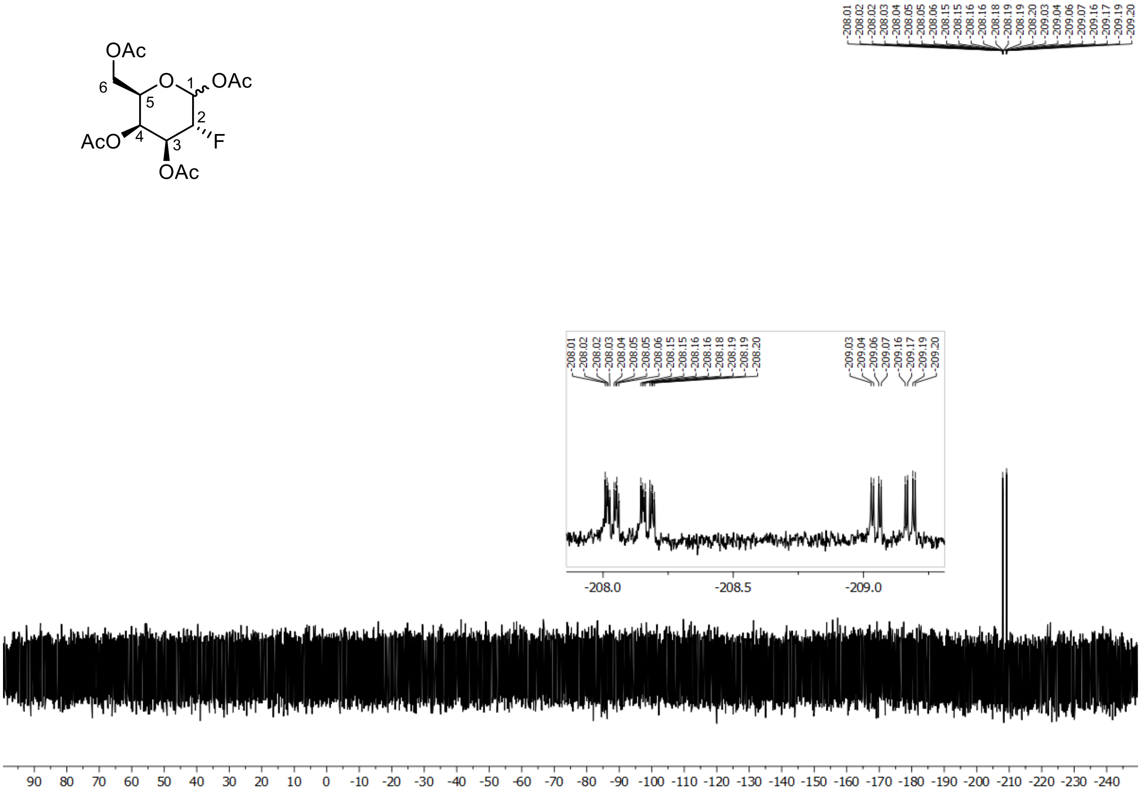

6 (^{1}H and ^{19}F, D_2O)

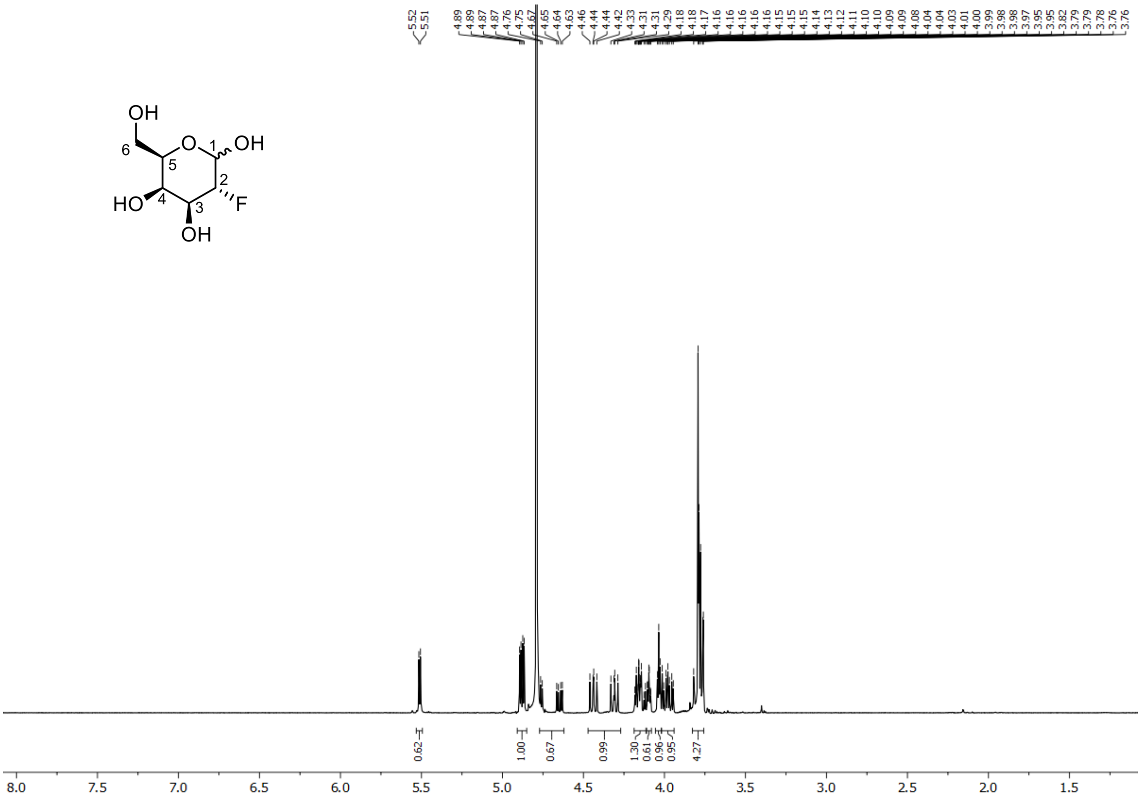

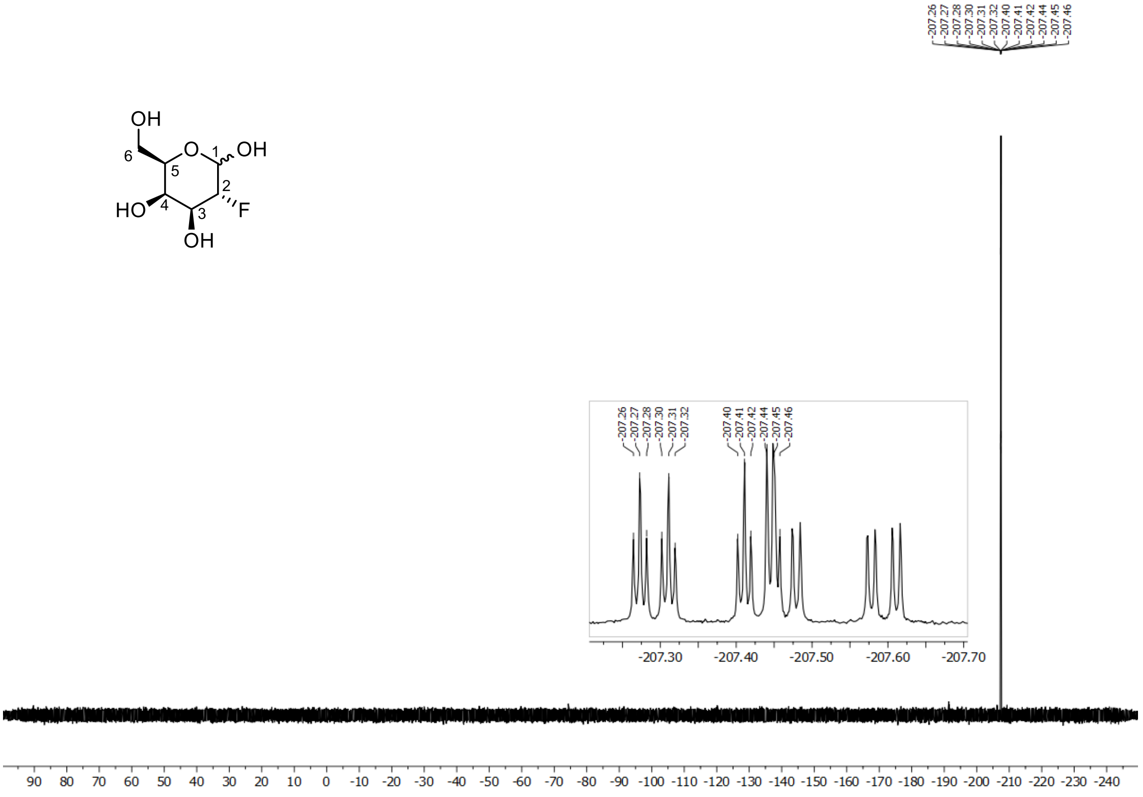

3 (^{1}H and ^{19}F, DMSO-*d6*)

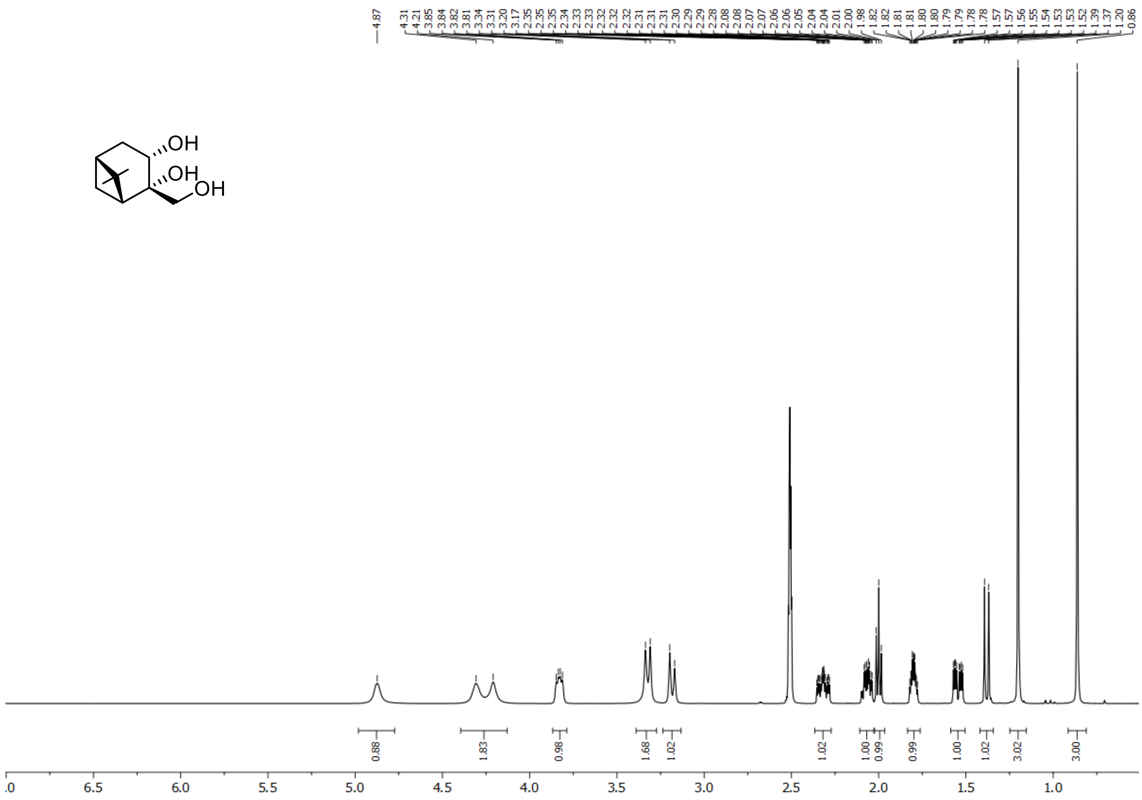

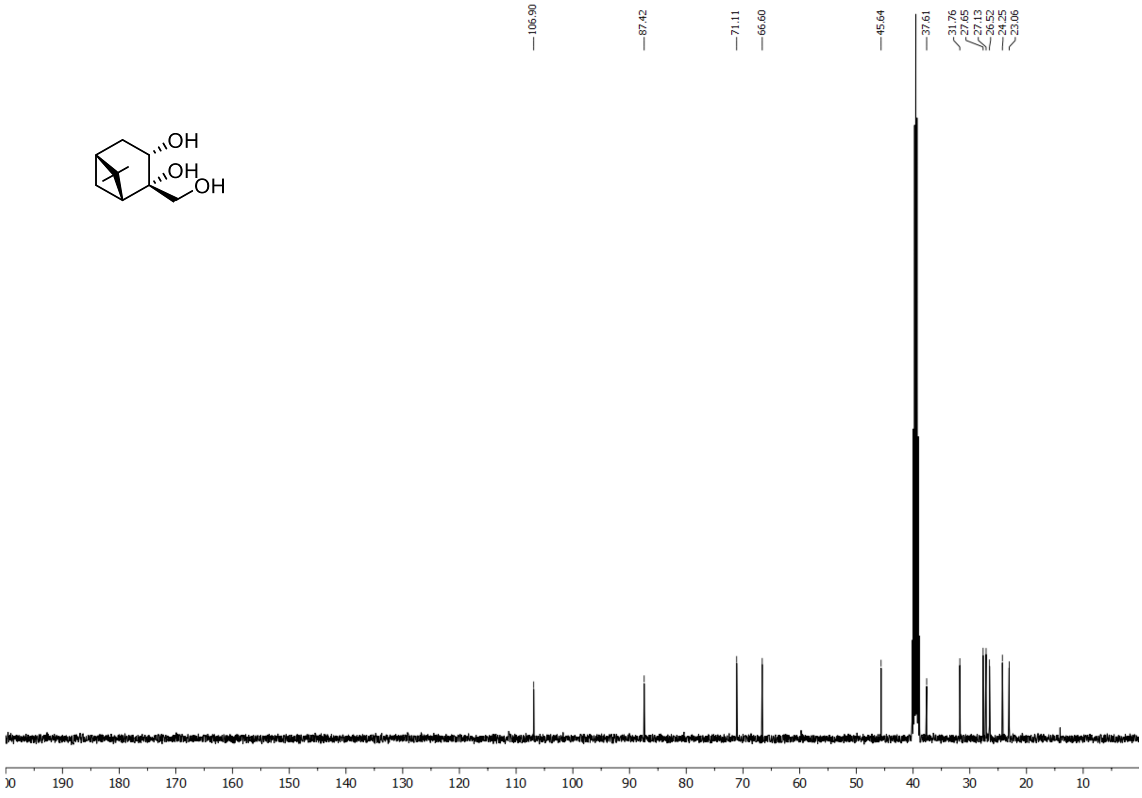

21 (^{1}H, $CDCl_3$)

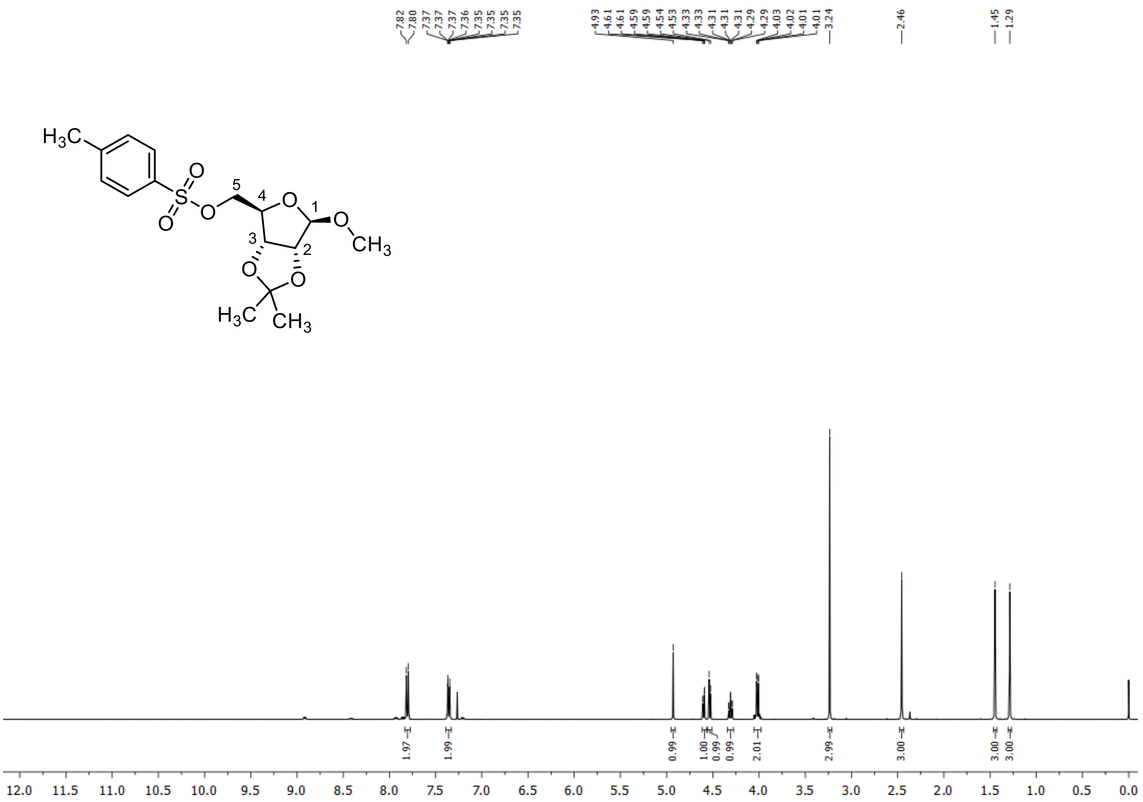

22 (^{1}H and ^{19}F{^{1}H}, $CDCl_3$)

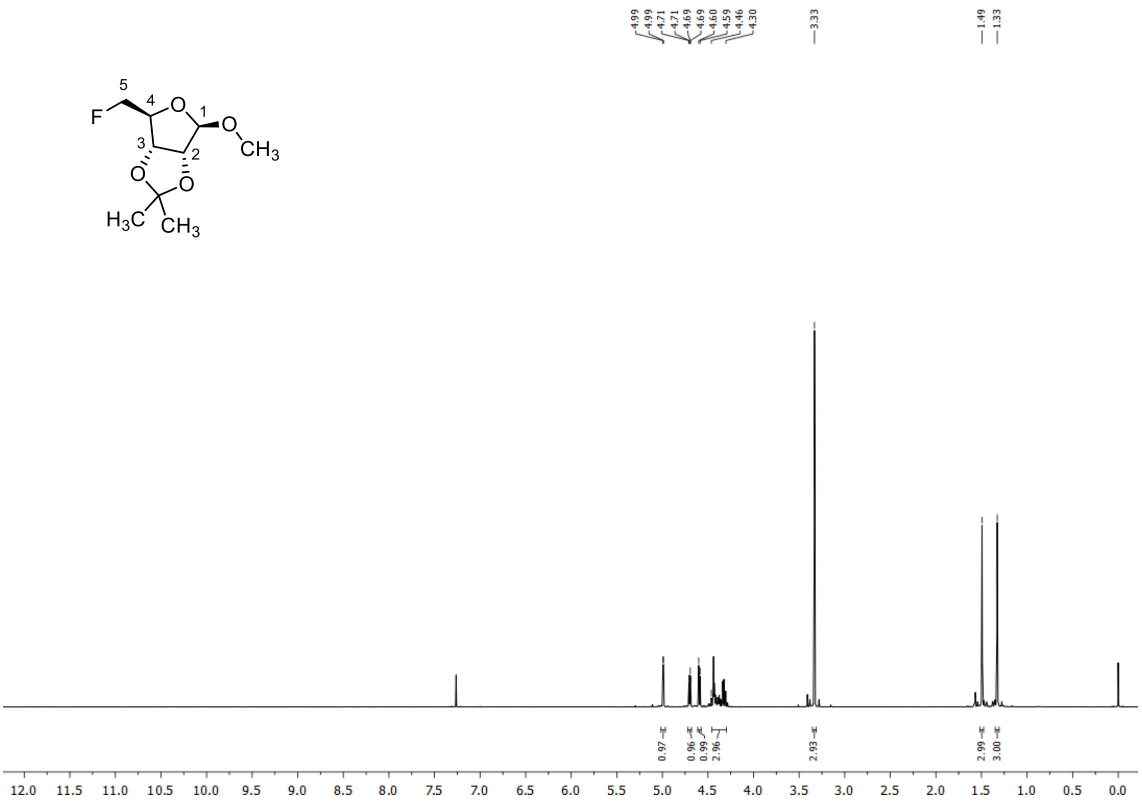

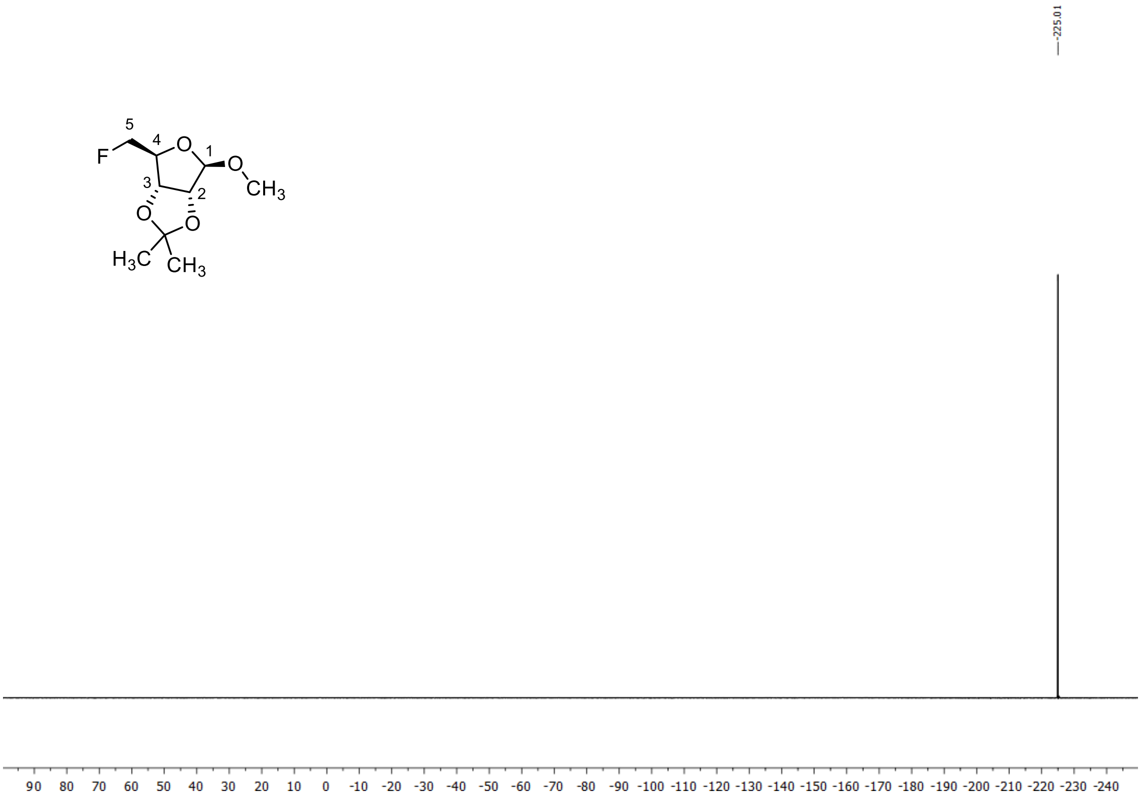

5 (^{1}H, ^{13}C and ^{19}F, D_2O)

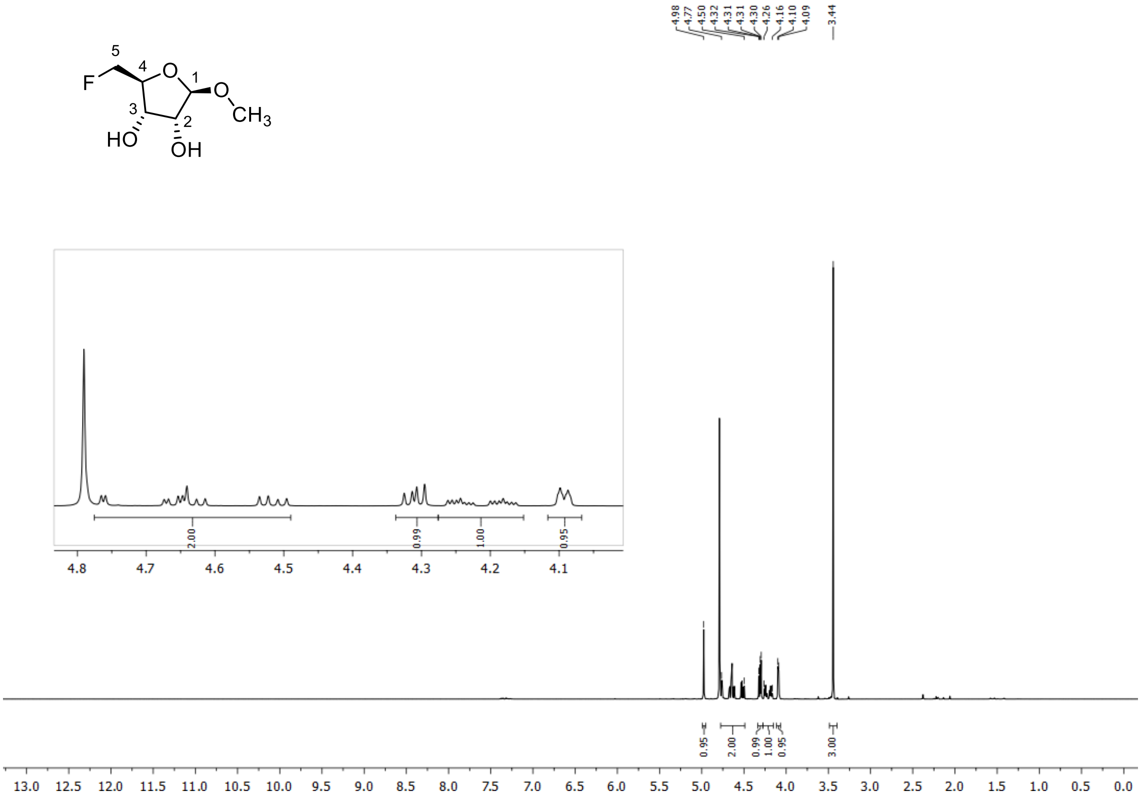

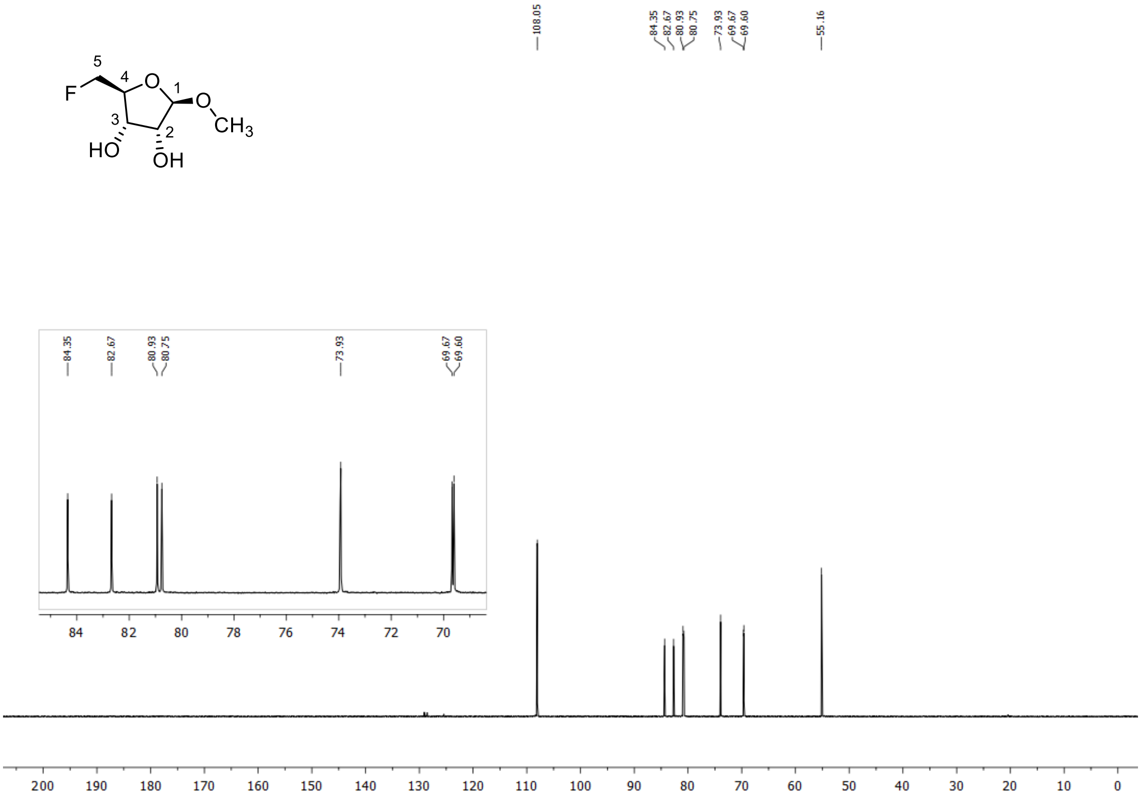

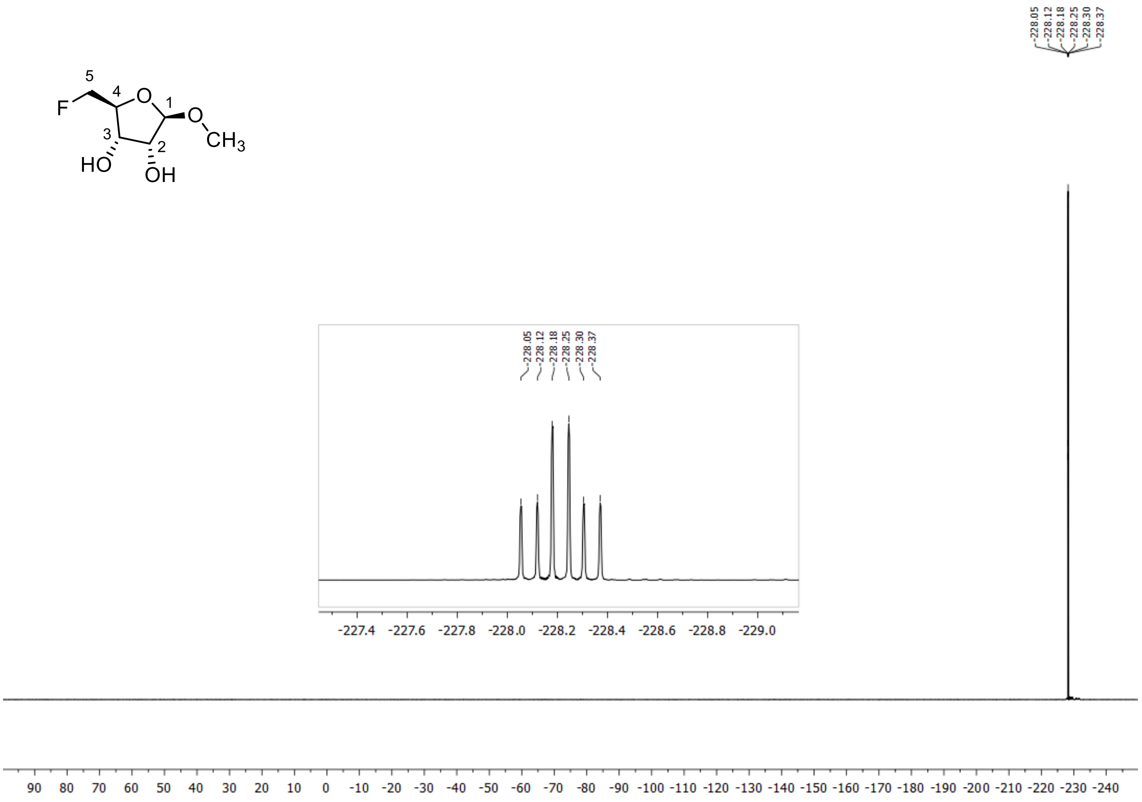
5
4
O
1
F
O
CH3
3
2
HO
OH
-228.05
-228.12
-228.18
-228.25
-228.30
-228.37
-227.4 -227.6 -227.8 -228.0 -228.2 -228.4 -228.6 -228.8 -229.0
90 80 70 60 50 40 30 20 10 0 -10 -20 -30 -40 -50 -60 -70 -80 -90 -100 -110 -120 -130 -140 -150 -160 -170 -180 -190 -200 -210 -220 -230 -240

23 (^{1}H, ^{13}C and ^{19}F, $CDCl_3$)

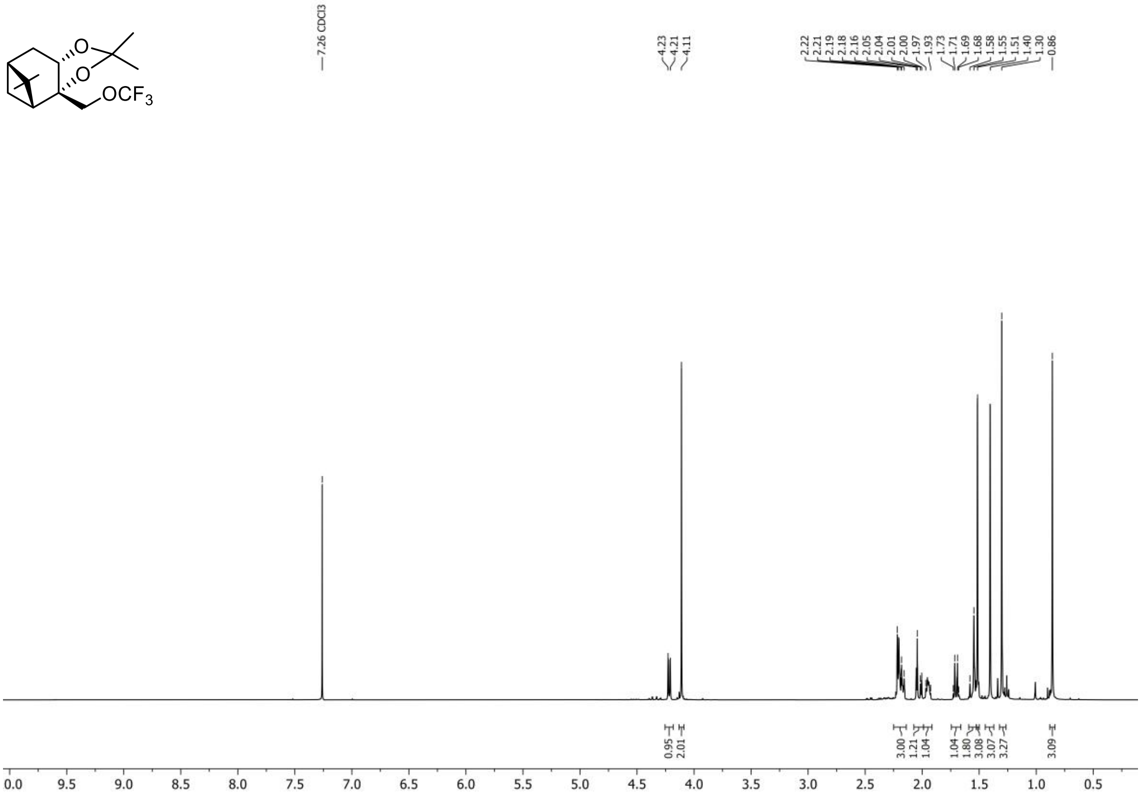

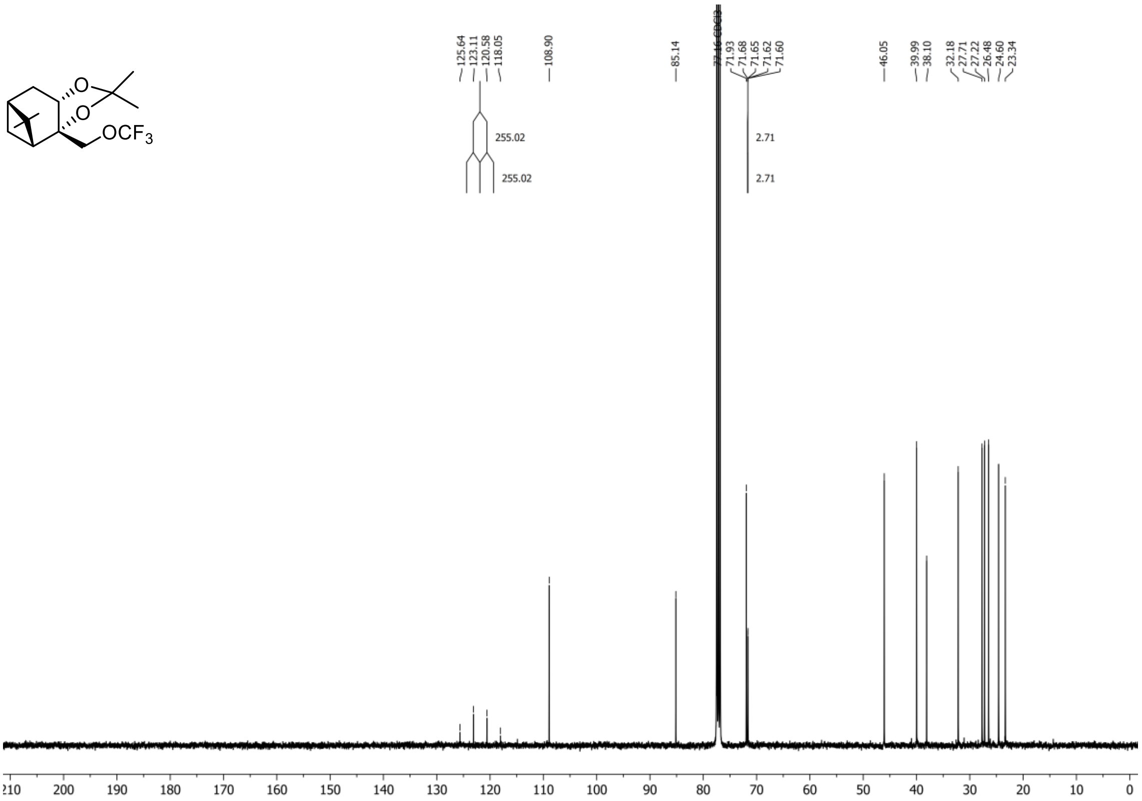

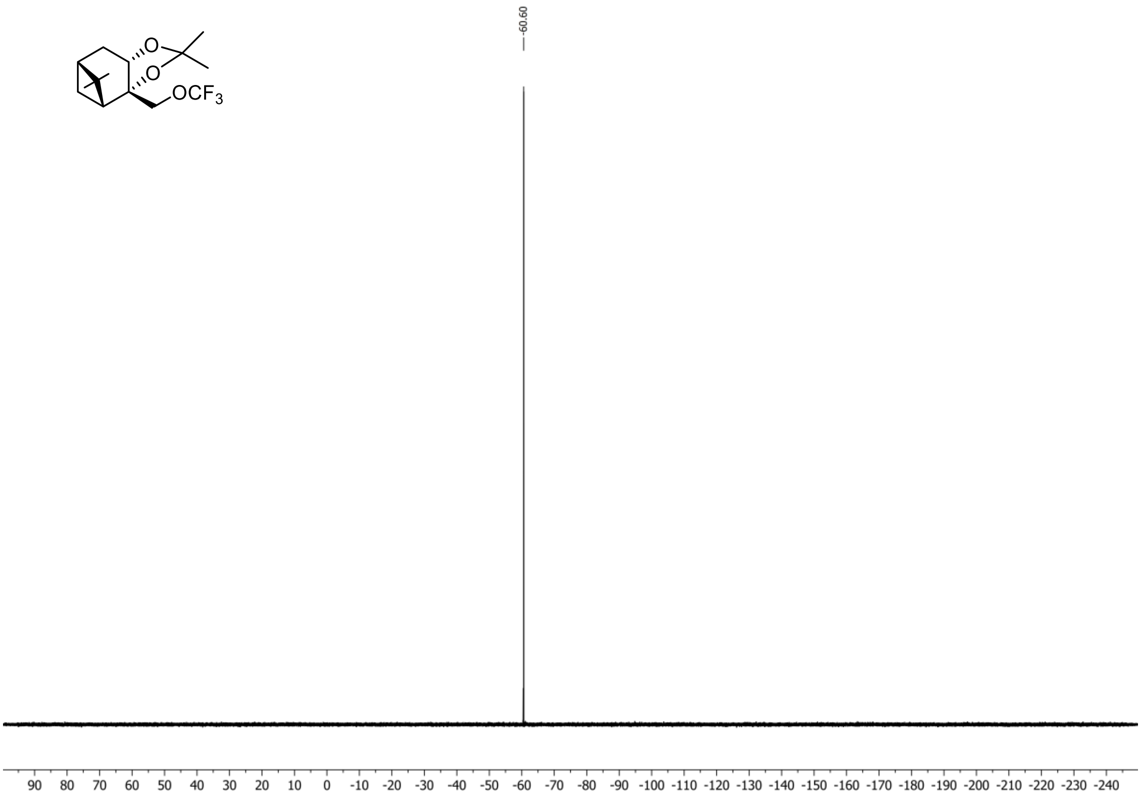
O
O
OCF3
-60.60
90 80 70 60 50 40 30 20 10 0 -10 -20 -30 -40 -50 -60 -70 -80 -90 -100 -110 -120 -130 -140 -150 -160 -170 -180 -190 -200 -210 -220 -230 -240

7 (^{1}H, ^{13}C and ^{19}F, DMSO-*d6*)

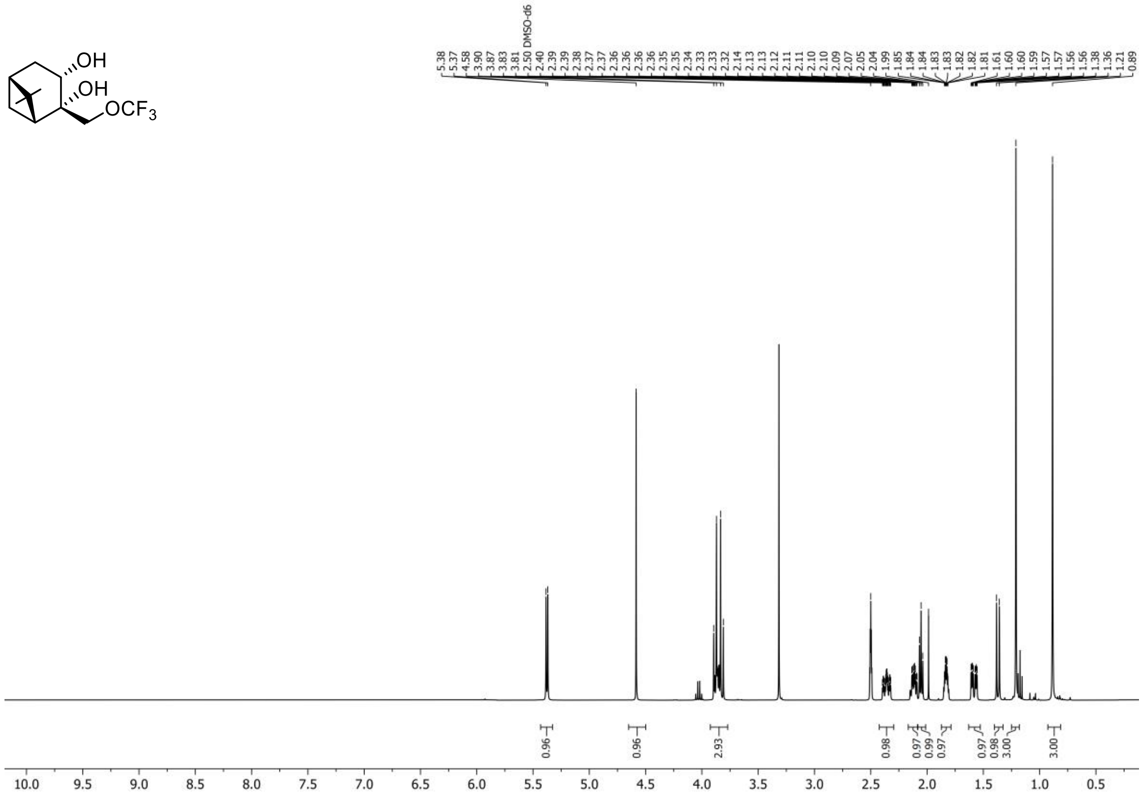

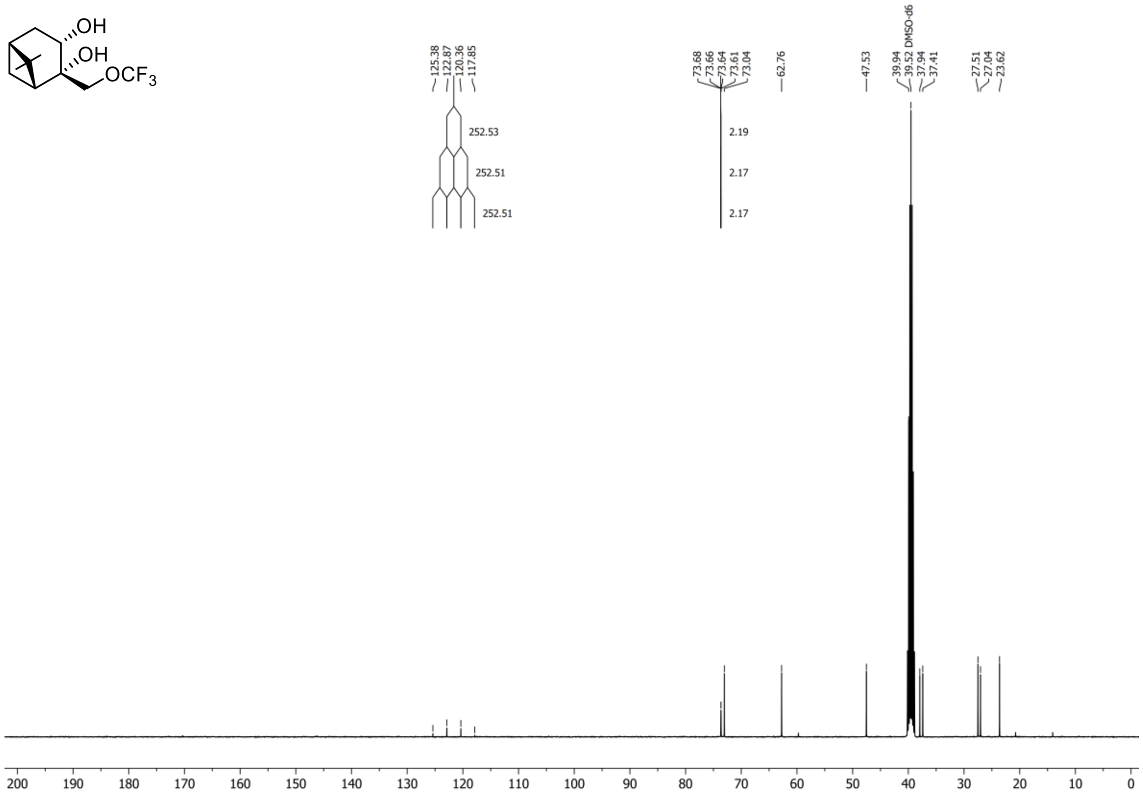

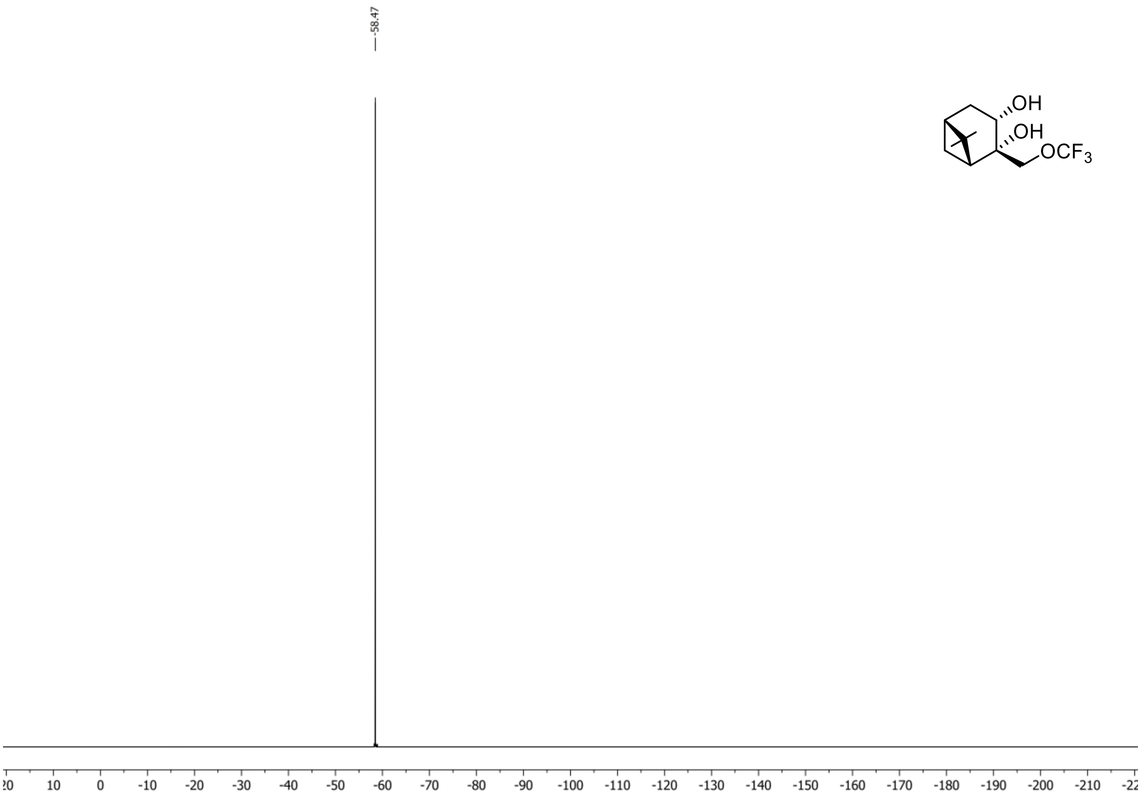
-58.47
OH
OH
OCF3
20
10
0
-10
-20
-30
-40
-50
-60
-70
-80
-90
-100
-110
-120
-130
-140
-150
-160
-170
-180
-190
-200
-210

3.2 Supplementary Note 2: Protein Sequences

3.2.1 Histone H3-Cys10

Protein Sequence

```
1          11         21         31         41         51
ARTKQTARKC TGGKAPRKQL ATKAARKSAP ATGGVKKPHR YRPGTVALRE IRRYQKSTEL
61         71         81         91         101        111
LIRKLPFQRL VREIAQDFKT DLRFQSSAVM ALQEASEAYL VALFEDTNLA AIHAKRVTIM
121        131
PKDIQLARRI RGERA
```

Formula: $C_{670}H_{1131}N_{215}O_{185}S_3$

Calculated molecular weight: 15254.9 g/mol

Extinction coefficient ε = 4470 M^{-1} cm^{-1}

3.2.2 Histone H3-Cys9

Protein Sequence

1	11	21	31	41	51
**ARTKQTARCS**	**TGGKAPRKQL**	**ATKAARKSAP**	**ATGGVKKPHR**	**YRPGTVALRE**	**IRRYQKSTEL**
61	71	81	91	101	111
**LIRKLPFQRL**	**VREIAQDFKT**	**DLRFQSSAVM**	**ALQEASEAYL**	**VALFEDTNLA**	**AIHAKRVTIM**
121	131				
**PKDIQLARRI**	**RGERA**				

Formula: $C_{667}H_{1124}N_{214}O_{186}S_3$

Calculated molecular weight: 15213.8 g/mol

Extinction coefficient ε = 4470 M^{-1} cm^{-1}

3.2.3 Annexin V-Cys316

Protein Sequence

1	11	21	31	41	51
**AQVLRGTVTD**	**FPGFDERADA**	**ETLRKAMKGL**	**GTDEESILTL**	**LTSRSNAQRQ**	**EISAAFKTLF**
61	71	81	91	101	111
**GRDLLDDLKS**	**ELTGKFEKLI**	**VALMKPSRLY**	**DAYELKHALK**	**GAGTNEKVLT**	**EIIASRTPEE**
121	131	141	151	161	171
**LRAIKQVYEE**	**EYGSSLEDDV**	**VGDTSGYYQR**	**MLVVLLQANR**	**DPDAGIDEAQ**	**VEQDAQALFQ**
181	191	201	211	221	231
**AGELKWGTDE**	**EKFITIFGTR**	**SVSHLRKVFD**	**KYMTISGFQI**	**EETIDRETSG**	**NLEQLLLAVV**
241	251	261	271	281	291
**KSIRSIPAYL**	**AETLYYAMKG**	**AGTDDHTLIR**	**VMVSRSEIDL**	**FNIRKEFRKN**	**FATSLYSMIK**
301	311				
**GDTSGDYKKA**	**LLLLCGEDD**				

Formula: $C_{1585}H_{2535}N_{423}O_{502}S_8$

Calculated molecular weight: 35805.6 g/mol

Extinction coefficient ε = 23380 M^{-1} cm^{-1}

3.2.4 Npβ-Cys61

Protein Sequence

1	11	21	31	41	51
**MFSSHHHHHH**	**SSGLVPRGSH**	**IDVGKLRQLY**	**AAGERDFSIV**	**DLRGAVLENI**	**NLSGAILHGA**
61	71	81	91	101	111
**CLDEANLQQA**	**NLSRADLSGA**	**TLNGADLRGA**	**NLSKADLSDA**	**ILDNAILEGA**	**ILDEAVLNQA**
121	131	141	151	161	171
**NLKAANLEQA**	**ILSHANIREA**	**DLSEANLEAA**	**DLSGADLAIA**	**DLHQANLHQA**	**ALERANLTGA**
181	191	201			
**NLEDANLEGT**	**ILEGGNNNLA**	**T**			

Formula: $C_{898}H_{1456}N_{276}O_{303}S_2$

Calculated molecular weight: 21031.2 g/mol

Extinction coefficient $\varepsilon = 1490\ M^{-1}\ cm^{-1}$

3.2.5 preSUMO1-Cys51

Protein Sequence

```
1          11         21         31         41         51
ADQEAKPSTE DLGDKKEGEY IKLKVIGQDS SEIHFKVKMT THLKKLKESY CQRQGVPMNS
61         71         81         91         101
LRFLFEGQRI ADNHTPKELG MEEEDVIEVY QEQTGGHSTV LEHHHHHH
```

Formula: $C_{543}H_{851}N_{157}O_{173}S_4$

Calculated molecular weight: 12474.9 g/mol

Extinction coefficient ε = 4470 M^{-1} cm^{-1}

3.2.6 mCherry-Cys131

Protein Sequence

```
1          11         21         31         41         51
MRGSHHHHHH GSMVSKGEED NMAIIKEFMR FKVHMEGSVN GHEFEIEGEG EGRPYEGTQT
61         71         81         91         101        111
AKLKVTKGGP LPFAWDILSP QFMYGSKAYV KHPADIPDYL KLSFPEGFKW ERVMNFEDGG
121        131        141        151        161        171
VVTVTQDSSL QDGEFIYKVK LRGTNFPCDG PVMQKKTMGW EASSERMYPE DGALKGEIKQ
181        191        201        211        221        231
RLKLKDGGHY DAEVKTTYKA KKPVQLPGAY NVNIKLDITS HNEDYTIVEQ YERAEGRHST
241
GGMDELYK
```

Formula: $C_{1250}H_{1915}N_{341}O_{377}S_{12}$

Calculated molecular weight: 28136.7 g/mol

Extinction coefficient ε = 34380 M^{-1} cm^{-1}

3.2.7 PstS-Cys197

Protein Sequence

1	11	21	31	41	51
**MEASLTGAGA**	**TFPAPVYAKW**	**ADTYQKETGN**	**KVNYQGIGSS**	**GGVKQIIANT**	**VDFGASDAPL**
61	71	81	91	101	111
**SDEKLAQEGL**	**FQFPTVIGGV**	**VLAVNIPGLK**	**SGELVLDGKT**	**LGDIYLGKIK**	**KWDDEAIAKL**
121	131	141	151	161	171
**NPGLKLPSQN**	**IAVVRRADGS**	**GTSFVFTSYL**	**AKVNEEWKNN**	**VGTGSTVKWP**	**IGLGGKGNDG**
181	191	201	211	221	231
**IAAFVQRLPG**	**AIGYVEYCYA**	**KQNNLAYTKL**	**ISADGKPVSP**	**TEENFANAAK**	**GADWSKTFAQ**
241	251	261	271	281	291
**DLTNQKGEDA**	**WPITSTTFIL**	**IHKDQKKPEQ**	**GTEVLKFFDW**	**AYKTGAKQAN**	**DLDYASLPDS**
301	311	321			
**VVEQVRAAWK**	**TNIKDSSGKP**	**LY**			

Formula: $C_{1563}H_{2426}N_{406}O_{476}S_2$

Calculated molecular weight: 35585.0 g/mol

Extinction coefficient ε = 61880 M^{-1} cm^{-1}

3.2.8 Histone H4-Cys16

Protein Sequence

```
        10         20         30         40         50         60
SGRGKGGKGL GKGGACRHRK VLRDNIQGIT KPAIRRLARR GGVKRISGLI YEETRGVLKV

        70         80         90        100
FLENVIRDAV TYTEHAKRKT VTAMDVVYAL KRQGRTLYGF GG
```

Formula: $C_{491}H_{830}N_{162}O_{134}S_2$

Calculated molecular weight: 11211.1 g/mol

Extinction coefficient $\varepsilon = 5960\ M^{-1}\ cm^{-1}$

3.2.9 AcrA-Cys123

Protein Sequence

```
        10         20         30         40         50         60
SKEEAPKIQM PPQPVTTMSA KSEDLPLSFT YPAKLVSDYD VIIKPQVSGV IVNKLFKAGD
        70         80         90        100        110        120
KVKKGQTLFI IEQDKFKASV DSAYGQALMA KATFENASKD FCRSKALFSK SAISQKEYDS
       130        140        150        160        170        180
SLATFNNSKA SLASARAQLA NARIDLDHTE IKAPFDGTIG DALVNIGDYV SASTTELVRV
       190        200        210        220        230        240
TNLNPIYADF FISDTDKLNL VRNTQSGKWD LDSIHANLNL NGETVQGKLY FIDSVIDANS
       250        260        270        280        290        300
GTVKAKAVFD NNNSTLLPGA FATITSEGFI QKNGFKVPQI GVKQDQNDVY VLLVKNGKVE
       310        320        330        340        350
KSSVHISYQN NEYAIIDKGL QNGDKIILDN FKKIQVGSEV KEIGAQLEHH HHHH
```

Formula: $C_{1732}H_{2743}N_{465}O_{538}S_4$

Calculated molecular weight: 38816.9 g/mol

Extinction coefficient ε = 20400 M^{-1} cm^{-1}

3.2.10 panC-Cys44

Protein Sequence

```
        10         20         30         40         50         60
TIPAFHPGEL NVYSAPGDVA DVSRALRLTG RRVMLVPTMG ALCEGHLALV RAAKRVPGSV
        70         80         90        100        110        120
VVVSIFVNPM QFGAGEDLDA YPRTPDDDLA QLRAEGVEIA FTPTTAAMYP DGLRTTVQPG
       130        140        150        160        170        180
PLAAELEGGP RPTHFAGVLT VVLKLLQIVR PDRVFFGEKD YQQLVLIRQL VADFNLDVAV
       190        200        210        220        230        240
VGVPTVREAD GLAMSSRNRY LDPAQRAAAV ALSAALTAAA HAATAGAQAA LDAARAVLDA
       250        260        270        280        290        300
APGVAVDYLE LRDIGLGPMP LNGSGRLLVA ARLGTTRLLD NIAIEIGTFA GTDRPDGYRA
       310        320
ILESHWRNKL AAALEHHHHH H
```

Formula: $C_{1513}H_{2427}N_{439}O_{440}S_{7}$

Calculated molecular weight: 34032.0 g/mol

Extinction coefficient $\varepsilon = 15930\ M^{-1}\ cm^{-1}$

3.2.11 panC-Cys47

Protein Sequence

```
        10         20         30         40         50         60
TIPAFHPGEL NVYSAPGDVA DVSRALRLTG RRVMLVPTMG ALHEGCLALV RAAKRVPGSV
        70         80         90        100        110        120
VVVSIFVNPM QFGAGEDLDA YPRTPDDDLA QLRAEGVEIA FTPTTAAMYP DGLRTTVQPG
       130        140        150        160        170        180
PLAAELEGGP RPTHFAGVLT VVLKLLQIVR PDRVFFGEKD YQQLVLIRQL VADFNLDVAV
       190        200        210        220        230        240
VGVPTVREAD GLAMSSRNRY LDPAQRAAAV ALSAALTAAA HAATAGAQAA LDAARAVLDA
       250        260        270        280        290        300
APGVAVDYLE LRDIGLGPMP LNGSGRLLVA ARLGTTRLLD NIAIEIGTFA GTDRPDGYRA
       310        320
ILESHWRNKL AAALEHHHHH H
```

Formula: $C_{1513}H_{2427}N_{439}O_{440}S_{7}$

Calculated molecular weight: 34032.0 g/mol

Extinction coefficient $\varepsilon = 15930\ M^{-1}\ cm^{-1}$

3.3 Supplementary Note 3: Protein Chemistry

3.4 Protein Expression and Purification

3.4.1 Histone H3-Cys10

Plasmid amplification

The plasmid was transformed into Agilent XL10-Gold® ultra-competent *E. coli* (Agilent) according to the manufacturer's transformation protocol and grown on agar plates (supplemented with ampicillin) over night. A single colony was selected and transferred to 5 mL of LB media containing 100 µg/mL ampicillin. The culture was incubated at 37 °C for 14 h and the cell pellet was harvested. Plasmid purification was conducted using a QIAprep Spin Miniprep kit and following the manufacturer's instructions.

Transformation and Expression

Protein Expression

BL21(DE3)pLysS competent *E. coli* (Agilent) were transformed with the plasmid following the manufacturer's instructions and grown on agar plates (supplemented with 100 µg/mL carbenicillin and 34 µg/mL chloramphenicol) over night. Single colonies were selected and transferred to 5 mL of LB media (containing 100 µg/mL carbenicillin and 34 µg/mL chloramphenicol) each. The starter cultures were incubated at 37 °C for 16 h. One starter culture was prepared for each 450 mL of expression media. Eight 2.5 L Erlenmeyer flasks charged with 450 mL of LB media supplemented with carbenicillin (at final concentration of 100 µg/mL) and chloramphenicol (at a final concentration of 34 µg/mL) were inoculated with the starter cultures. The bacterial cultures were incubated at 37 °C with vigorous shaking (250 rpm) an optical density value (OD_{600}) of 0.6 – 0.8 was observed. Protein expression was induced by addition of 1 mM IPTG and the culture was further incubated at 37 °C for 2 h. The cells were harvested by centrifugation (7,500 rpm, 20 min, 4 °C). The cell pellet was suspended in 25 mL of lysis buffer (50 mM TRIS base, 100 mM NaCl, 1 mM EDTA, 5 mM BME, pH 7.5) containing a tablet of cOmplete™ mini EDTA-free protease inhibitor cocktail. The sample was frozen in liquid nitrogen and stored at −80 °C until purification.

Protein Purification

The samples were thawed on ice and DNase I (1 mg) was added. The cell suspension was then sonicated on ice (5 cycles, 40% amplitude, 30 sec. on, 60 sec. off). The samples were centrifuged (20,000 rpm, 20 min, 4 °C) and the supernatant was discarded. The cell pellet was re-suspended in 20 mL of TW buffer (50 mM TRIS base, 100 mM NaCl, 1 mM EDTA, 5 mM BME, 1% Triton X-100, pH 7.5) using a mixer and sonicated on ice (2 cycles, 40% amplitude, 30 sec. on, 60 sec. off). The mixture was centrifuged (20,000 rpm, 10 min, 4 °C) and the supernatant was discarded. The pellet was again suspended in 20 mL of TW buffer, centrifuged (20,000 rpm, 10 min, 4 °C) and the supernatant was discarded. Washingn was repeated one more time with TW buffer and one more time with wash buffer (50 mM TRIS base, 100 mM NaCl, 1 mM EDTA, 5 mM BME, pH 7.5). The pellet was incubated with 1 mL of DMSO at room temperature for 10 min before 10 mL of unfolding buffer (7 M Gdn·HCl, 10 mM TRIS base, 1 mM EDTA, 10 mM DTT, 1 mM benzamidine) were added. The mixture was shaken at room temperature for 1 h and centrifuged (20,000 rpm, 10 min, 4 °C). The supernatant was purified by FPLC using a size-exclusion column running with SAU-100 buffer (7 M urea, 20 mM NaOAc, 100 mM NaCl, 1 mM EDTA, 5 mM BME, pH 5.2). The Histone H3 containing fractions were further purified by anion exchange chromatography using a linear gradient from SAU-100 buffer to SAU-1000 buffer (7 M urea, 20 mM NaOAc, 1000 mM NaCl, 1 mM EDTA, 5 mM BME, pH 5.2). The clean fractions were pooled together and dialyzed four times against 4 L of water containing 2 mM BME. The protein solution was lyophilized yielding 230 mg of Histone H3-Cys10 (64 mg/L expression volume). The lyophilized protein stored at −20 °C until needed.

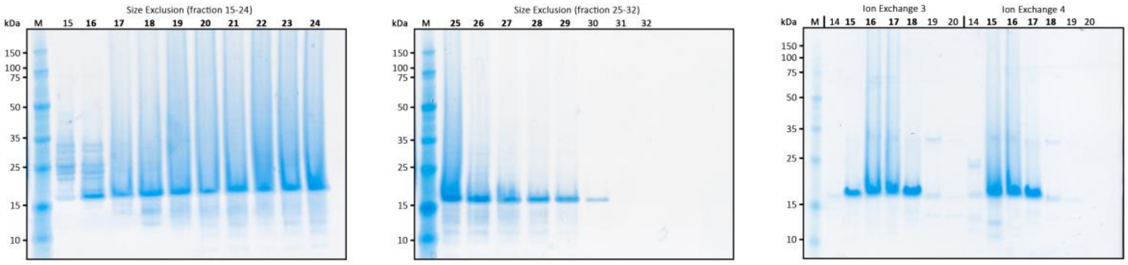

Purification and analysis of of purified fractions of Histone H3-Cys10 via SDS-PAGE and coomassie staining (M = marker). A single experiment was deemed sufficient, as no quantification was required.

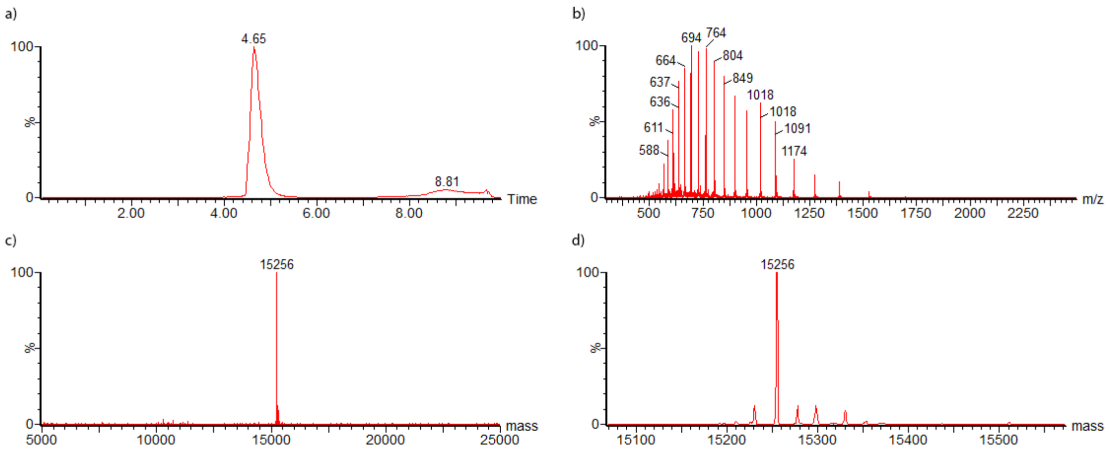

LCMS analysis of Histone H3-Cys10 a) total ion chromatogram b) ion series c) deconvoluted spectrum d) magnification of the major peak**;** calculated mass: 15255 g/mol; observed masses: 15256 g/mol.

3.4.2 Histone H3-Cys9

Plasmid amplification

The plasmid was transformed into Agilent XL10-Gold® ultra-competent *E. coli* (Agilent) according to the manufacturer's transformation protocol and grown on agar plates (supplemented with ampicillin) over night. A single colony was selected and transferred to 5 mL of LB media containing 100 µg/mL ampicillin. The culture was incubated at 37 °C for 14 h and the cell pellet was harvested. Plasmid purification was conducted using a QIAprep Spin Miniprep kit and following the manufacturer's instructions.

Transformation and Expression

Protein Expression

BL21(DE3)pLysS competent *E. coli* (Agilent) were transformed with the plasmid following the manufacturer's instructions and grown on agar plates (supplemented with 100 µg/mL carbenicillin and 34 µg/mL chloramphenicol) over night. Single colonies were selected and transferred to 5 mL of LB media (containing 100 µg/mL carbenicillin and 34 µg/mL chloramphenicol) each. The starter cultures were incubated at 37 °C for 16 h. One starter culture was prepared for each 450 mL of expression media. Eight 2.5 L Erlenmeyer flasks charged with 450 mL of LB media supplemented with carbenicillin (at final concentration of 100 µg/mL) and chloramphenicol (at a final concentration of 34 µg/mL) were inoculated with the starter cultures. The bacterial cultures were incubated at 37 °C with vigorous shaking (250 rpm) an optical density value (OD_{600}) of 0.6 – 0.8 was observed. Protein expression was induced by addition of 1 mM IPTG and the culture was further incubated at 37 °C for 2 h. The cells were harvested by centrifugation (7,500 rpm, 20 min, 4 °C). The cell pellet was suspended in 25 mL of lysis buffer (50 mM TRIS base, 100 mM NaCl, 1 mM EDTA, 5 mM BME, pH 7.5) containing a tablet of cOmplete™ mini EDTA-free protease inhibitor cocktail. The sample was frozen in liquid nitrogen and stored at −80 °C until purification.

Protein Purification

The samples were thawed on ice and DNase I (1 mg) was added. The cell suspension was then sonicated on ice (5 cycles, 40% amplitude, 30 sec. on, 60 sec. off). The samples were centrifuged (20,000 rpm, 20 min, 4 °C) and the supernatant was discarded. The cell pellet was re-suspended in 20 mL of TW buffer (50 mM TRIS base, 100 mM NaCl, 1 mM EDTA, 5 mM BME, 1% Triton X-100, pH 7.5) using a mixer and sonicated on ice (2 cycles, 40% amplitude, 30 sec. on, 60 sec. off). The mixture was centrifuged (20,000 rpm, 10 min, 4 °C) and the supernatant was discarded. The pellet was again suspended in 20 mL of TW buffer, centrifuged (20,000 rpm, 10 min, 4 °C) and the supernatant was discarded. Washingn was repeated one more time with TW buffer and one more time with wash buffer (50 mM TRIS base, 100 mM NaCl, 1 mM EDTA, 5 mM BME, pH 7.5). The pellet was incubated with 1 mL of DMSO at room temperature for 10 min before 10 mL of unfolding buffer (7 M Gdn·HCl, 10 mM TRIS base, 1 mM EDTA, 10 mM DTT, 1 mM benzamidine) were added. The mixture was shaken at room temperature for 1 h and centrifuged (20,000 rpm, 10 min, 4 °C). The supernatant was purified by FPLC using a size-exclusion column running with SAU-100 buffer (7 M urea, 20 mM NaOAc, 100 mM NaCl, 1 mM EDTA, 5 mM BME, pH 5.2). The Histone H3 containing fractions were further purified by anion exchange chromatography using a linear gradient from SAU-100 buffer to SAU-1000 buffer (7 M urea, 20 mM NaOAc, 1000 mM NaCl, 1 mM EDTA, 5 mM BME, pH 5.2). The clean fractions were pooled together and dialyzed four times against 4 L of water containing 2 mM BME. The protein solution was lyophilized yielding 187 mg of Histone H3-Cys9 (52 mg/L expression volume). The lyophilized protein stored at −20 °C until needed.

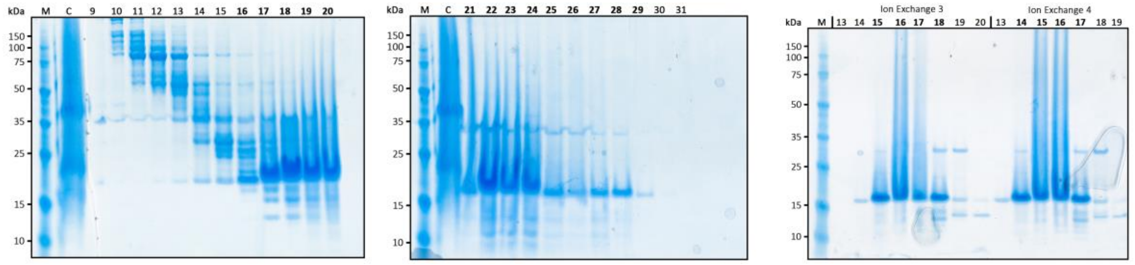

Purification and analysis of of purified fractions of Histone H3-Cys9 via SDS-PAGE and coomassie staining (M = marker). A single experiment was deemed sufficient, as no quantification was required.

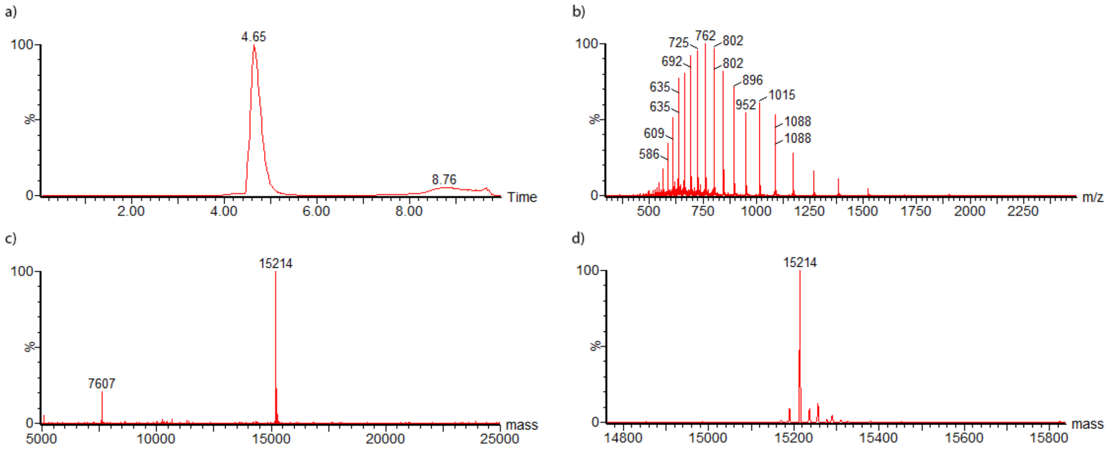

LCMS analysis of Histone H3-Cys9: a) total ion chromatogram b) ion series c) deconvoluted spectrum d) magnification of the major peak**;** calculated mass: 15214 g/mol; observed masses: 15214 g/mol.

3.4.3 [^{13}C-^{15}N]Histone H3-Cys10

Media Preparation

M9 medium was prepared by dissolving the following in 1 L of distilled water.

6.0 g	Na_2HPO_4
3.0 g	KH_2PO_4
0.5 g	NaCl
1.0 g	[^{15}N]NH_4Cl
1 mL	1.0 M $MgSO_4$
1 mL	100 mM $CaCl_2$
1 mL	Microsolution 1
0.1 mL	Microsolution 2

Microsolution 1

Microsolution 1 was prepared by dissolving the following in 10 mL of distilled water.

150 mg	$CaCl_2$
300 mg	Na_2EDTA
250 mg	$FeCl_3$

Microsolution 2

Microsolution 2 was prepared by dissolving the following in 10 mL of distilled water.

240 mg	$CuSO_4 \cdot 5H_2O$
180 mg	$MnSO_4 \cdot H_2O$
27 mg	$ZnSO_4 \cdot 7H_2O$
27 mg	$CoCl_2$

Microsolution 3

Microsolution 3 was prepared by dissolving the following in 10 mL of distilled water. The solution was sterile filtered.

100 mg	Thiamine
100 mg	Biotin

The pH was adjusted to 7.4 and the medium was sterilized and cooled to room temperature before [^{13}C]glucose (2.0 g), Microsolution 3 (1 mL) and antibiotics (final concentration: chloramphenicol: 34 µg/mL, carbenicillin: 100 µg/mL) were added.

Transformation and Expression

Protein Expression

BL21(DE3)pLysS competent *E. coli* (Agilent) were transformed with the plasmid following the manufacturer's instructions and grown on agar plates (supplemented with 100 µg/mL carbenicillin and 34 µg/mL chloramphenicol) over night. Single colonies were selected and transferred to 5 mL of LB media (containing 100 µg/mL carbenicillin and 34 µg/mL chloramphenicol) each. The starter cultures were incubated at 37 °C until an optical density value (OD_{600}) of 0.5 was observed. The cultures were pelleted by centrifugation (4,000 g, 10 min, 4°C) and resuspended in 10 mL M9 medium. One starter culture was prepared for each 500 mL of expression media. Two 2.5 L Erlenmeyer flasks charged with 500 mL of M9 media each were inoculated with the starter cultures. The bacterial cultures were incubated at 37 °C with vigorous shaking (250 rpm) an optical density value (OD_{600}) of 0.7 was observed. Protein expression was induced by addition of 1 mM IPTG and the culture was further incubated at 37 °C for 3 h. The cells were harvested by centrifugation (8,000 rpm, 20 min, 4 °C). The cell pellet was suspended in 25 mL of lysis buffer (50 mM TRIS base, 100 mM NaCl, 1 mM EDTA, 5 mM BME, pH 7.5) containing a tablet of cOmplete™ mini EDTA-free protease inhibitor cocktail. The sample was frozen in liquid nitrogen and stored at −80 °C until purification.

Protein Purification

Protein purification was conducted as previously described for Histone H3-Cys10 to yield a total of 2.5 mg of [^{13}C-^{15}N]Histone H3-Cys10 as a white solid. The molecular mass was determined by LCMS (M_w = 16117 g/mol) and corresponds to 97% heavy atom incorporation.

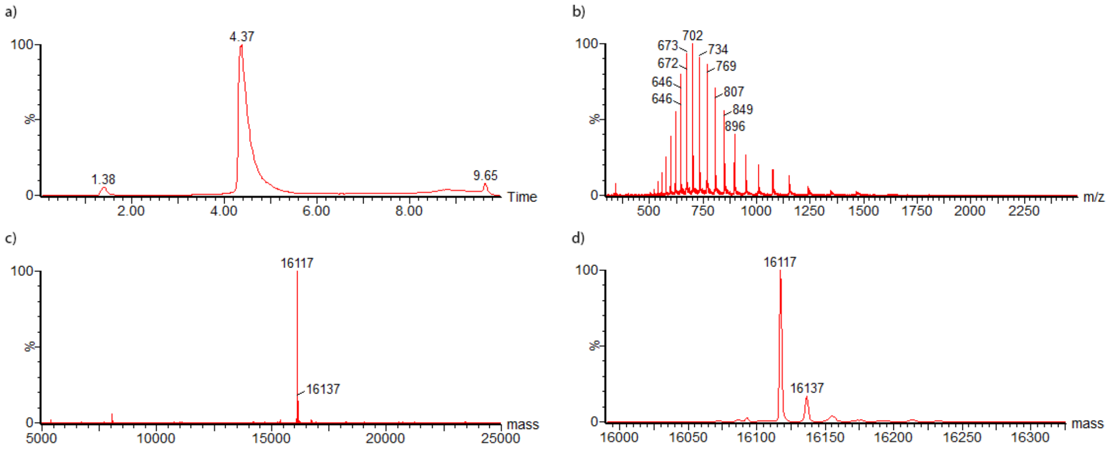

LCMS analysis of [^{13}C-^{15}N]Histone H3-Cys10: a) total ion chromatogram b) ion series c) deconvoluted spectrum d) magnification of the major peak**;** calculated mass: 16140 g/mol (full incorporation of ^{13}C and ^{15}N); observed mass: 16117 g/mol (97% heavy atom incorporation).

3.4.4 [15N]Histone H3-Cys9, [13C-15N] Histone H3-Cys9 and [15N]Histone H3-Cys10

Transformation and Expression

M9 Media Production

M9 media was produced following a modified literature procedure.[20]

In 1 L of milliQ H_2O were dissolved

6.0 g	Na_2HPO_4
3.0 g	KH_2PO_4
0.5 g	NaCl
0.5 g	$[^{15}N]NH_4Cl$

The pH was adjusted to 7.4 with HCl/NaOH as needed and the solution was autoclaved and cooled to room temperature. Before use,

11 mg	$CaCl_2$
120 mg	$MgSO_4$
3.0 g	glucose (or $[^{13}C]$glucose)
10 mg	biotin
10 mg	thiamine

and antibiotics (100 µg/mL carbenicillin and 34 µg/mL chloramphenicol) were added.

Protein Expression

BL21(DE3)pLysS competent *E. coli* (Agilent) were transformed with the plasmid encoding for Histone H3-Cys9 or Histone H3-Cys10 following the manufacturer's instructions and grown on agar plates (supplemented with 100 µg/mL carbenicillin and 34 µg/mL chloramphenicol) over night. Single colonies were selected and transferred to

2 × 15 mL LB medium (supplemented with 100 µg/mL carbenicillin and 34 µg/mL chloramphenicol). The starter cultures were grown at 37°C for 3 h, centrifuged (3,500 rpm, 10 min, 4°C) and the LB medium was replaced with M9 medium (35 mL each). The starter cultures were grown overnight at 30°C and used to inoculate 2 × 500 mL M9 medium. Expression cultures were grown at 37°C until OD600 = 0.7, induced with 0.5 mM IPTG and grown at 30°C for 14 h. The cell pellets was harvested and suspended in 25 mL of lysis buffer (50 mM TRIS base, 100 mM NaCl, 1 mM EDTA, 5 mM BME, pH 7.5) containing a tablet of cOmplete™ mini EDTA-free protease inhibitor cocktail.

Protein Purification

DNase I (1 mg) was added and the cell suspension was then sonicated on ice (5 cycles, 40% amplitude, 30 sec. on, 60 sec. off). The sample was centrifuged (20,000 rpm, 20 min, 4°C) and the supernatant was discarded. The cell pellet was re-suspended in 20 mL of TW buffer (50 mM TRIS base, 100 mM NaCl, 1 mM EDTA, 5 mM BME, 1% Triton X-100, pH 7.5) and sonicated on ice (2 cycles, 40% amplitude, 30 sec. on, 60 sec. off). The mixture was centrifuged (20,000 rpm, 10 min, 4 °C) and the supernatant was discarded. Washing was repeated wash buffer (50 mM TRIS base, 100 mM NaCl, 1 mM EDTA, 5 mM BME, pH 7.5). The pellet was incubated with 1 mL of DMSO at room temperature for 10 min before 10 mL of unfolding buffer (7 M Gdn·HCl, 10 mM TRIS base, 1 mM EDTA, 10 mM DTT, 1 mM benzamidine) were added. The mixture was shaken at room temperature for 1 h and centrifuged (20,000 rpm, 10 min, 4°C). The supernatant was concentrated to approximately 1 mL using a VivaSpin concentrator (5,000 Da MWCO) and purified by FPLC using a size-exclusion column running with SAU-100 buffer (7 M urea, 20 mM NaOAc, 100 mM NaCl, 1 mM EDTA, 5 mM BME, pH 5.2). The clean Histone H3 containing fractions were pooled together and dialyzed four times against 4 L of water containing 2 mM BME. The protein solution was lyophilized yielding 28 mg of [^{15}N]Histone H3-Cys9, 84 mg of [^{13}C-^{15}N]Histone H3-Cys9, or 38 mg of Histone H3 Cys10, respectively. The lyophilized protein stored at −80°C until needed. LCMS analysis showed 99% ^{15}N incorporation.

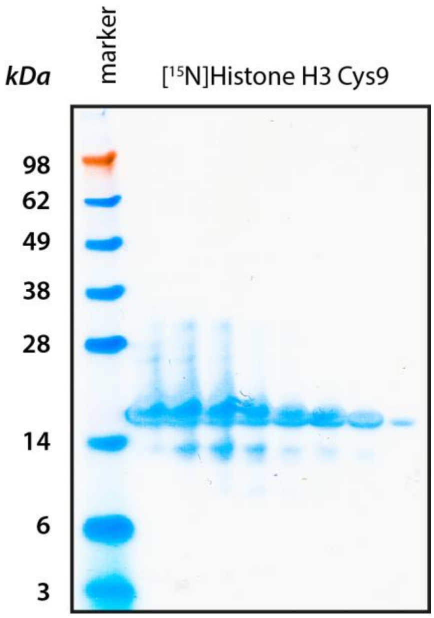

Analysis of purified fractions of [^{15}N]Histone H3-Cys9 via SDS-PAGE (10% Bis-TRIS gel, MES buffer, 200 V, 40 min) and coomassie staining (marker = SeeBlue™ Plus2 Pre-stained Protein Standard). A single experiment was deemed sufficient, as no quantification was required.

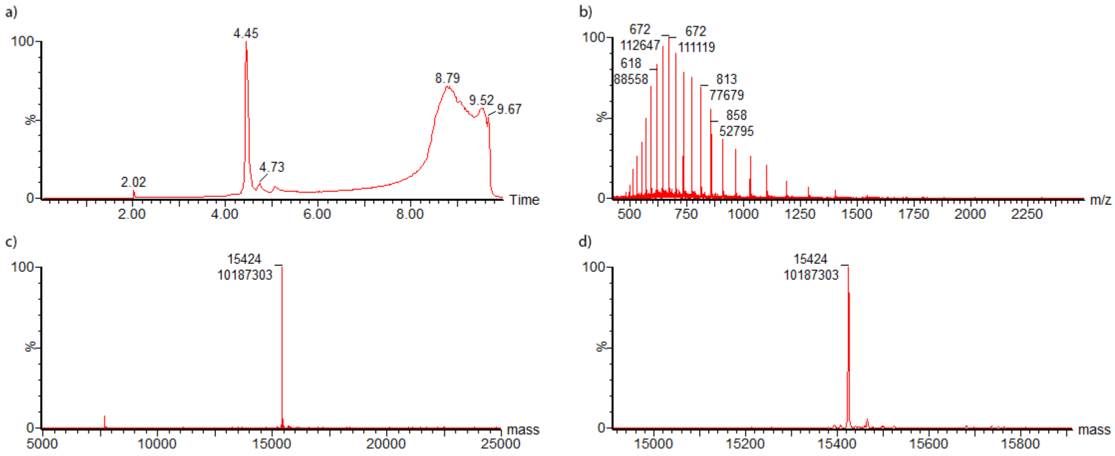

LCMS analysis of [^{15}N]Histone H3-Cys9: a) total ion chromatogram b) ion series c) deconvoluted spectrum d) magnification of the major peak**;** calculated mass: 15426 g/mol; observed mass: 15424 g/mol.

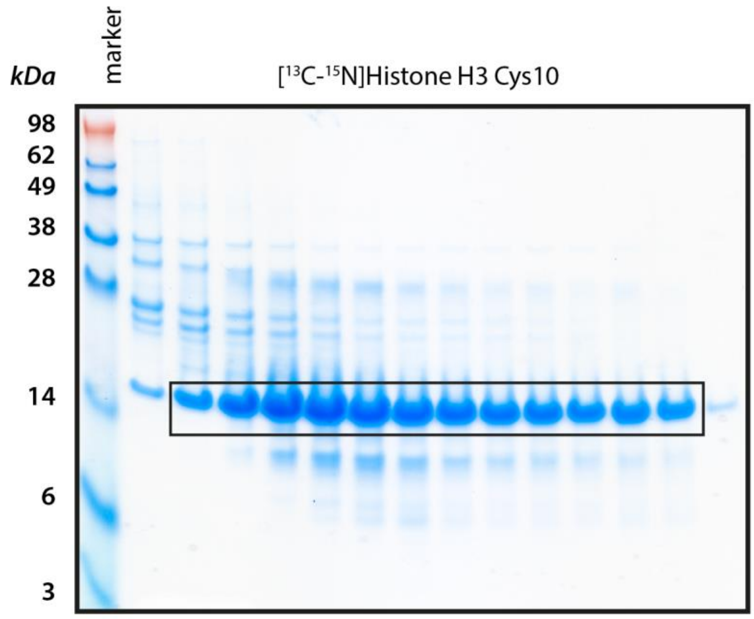

Analysis of purified fractions of [^{13}C-^{15}N]Histone H3-Cys9 via SDS-PAGE (10% Bis-TRIS gel, MES buffer, 200 V, 40 min) and coomassie staining (marker = SeeBlue™ Plus2 Pre-stained Protein Standard). A single experiment was deemed sufficient, as no quantification was required.

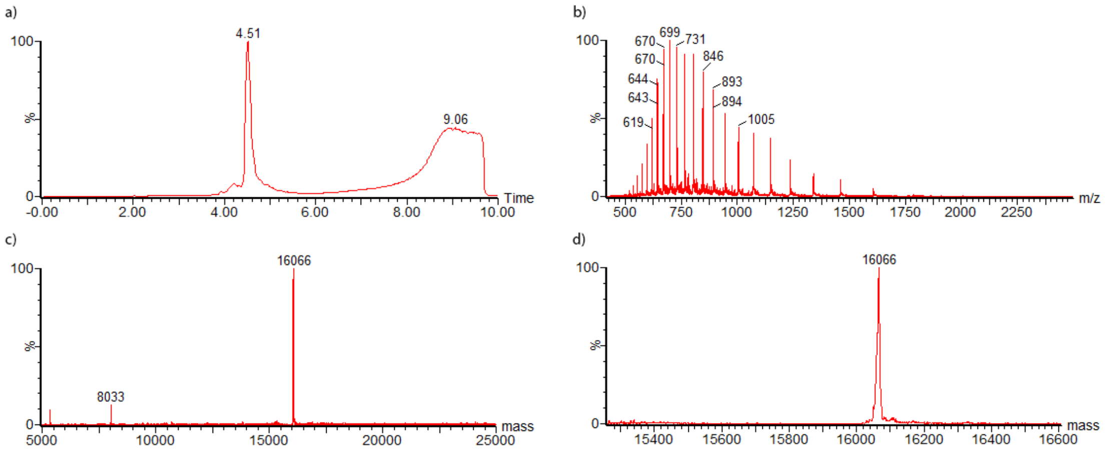

LCMS analysis of [^{13}C-^{15}N]Histone H3-Cys9: a) total ion chromatogram b) ion series c) deconvoluted spectrum d) magnification of the major peak.

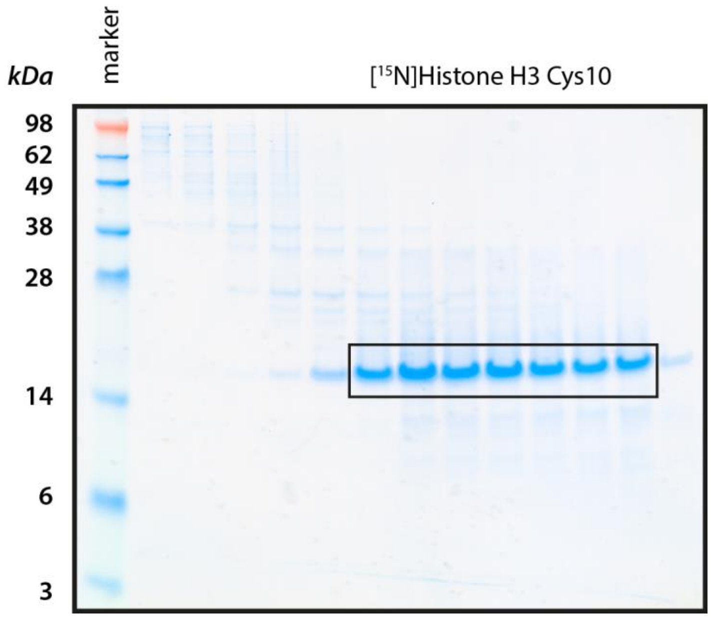

Analysis of purified fractions of [^{15}N]Histone H3-Cys10 via SDS-PAGE (10% Bis-TRIS gel, MES buffer, 200 V, 40 min) and coomassie staining (marker = SeeBlue™ Plus2 Pre-stained Protein Standard). A single experiment was deemed sufficient, as no quantification was required.

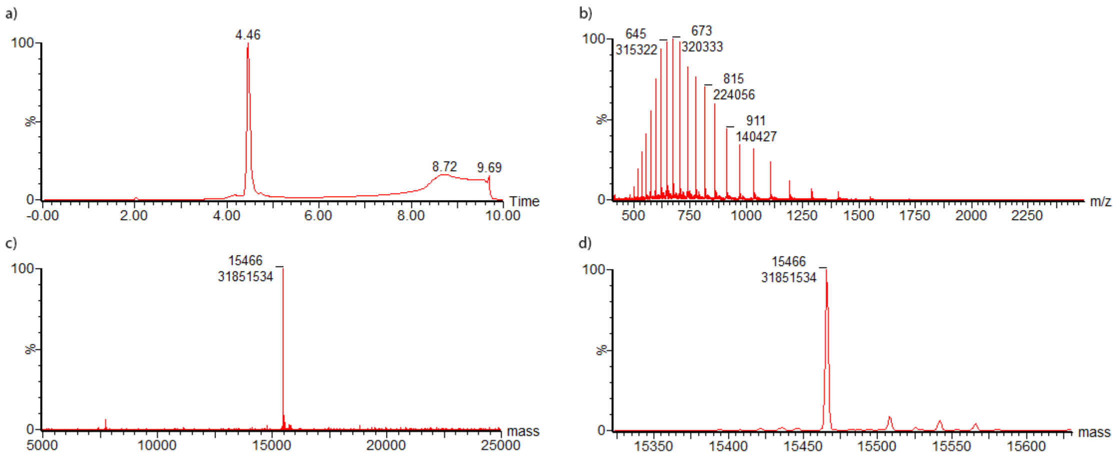

LCMS analysis of [^{15}N]Histone H3-Cys10: a) total ion chromatogram b) ion series c) deconvoluted spectrum d) magnification of the major peak**;** calculated mass: 15468 g/mol; observed mass: 15466 g/mol.

3.4.5 Annexin V-Cys316

Plasmid amplification

The plasmid was transformed into Invitrogen One Shot TOP10 chemically competent *E. coli* according to the manufacturer's transformation protocol and grown on agar plates (supplemented with ampicillin) over night. A single colony was selected and transferred to 5 mL of LB media containing 100 µg/mL ampicillin. The culture was incubated at 37 °C for 14 h and the cell pellet was harvested. Plasmid purification was conducted using a QIAprep Spin Miniprep kit and following the manufacturer's instructions.

Transformation and Expression

Protein Expression

T7 Express Competent *E. coli* (New England BioLabs) were transformed with the plasmid following the manufacturer's instructions and grown on agar plates (supplemented with 100 µg/mL ampicillin) over night. Single colonies were selected and transferred to 10 mL of LB media (containing 100 µg/mL ampicillin) each. The starter culture was incubated at 37 °C for 15 h. One starter culture was prepared for every 500 mL of expression media. Four 2.5 L Erlenmeyer flasks charged with 500 mL of LB media supplemented with carbenicillin (at final concentration of 100 µg/mL) were inoculated with the starter cultures. The bacterial cultures were incubated at 37 °C with vigorous shaking (250 rpm) an optical density value (OD_{600}) of 0.45 – 0.55 was observed. Protein expression was induced by addition of 1 mM IPTG and the culture was further incubated at 37 °C for 3 h. The cells were harvested by centrifugation (8,000 rpm, 10 min, 4 °C). The cell pellet was suspended in 60 mL of TBS buffer (50 mM TRIS base, 150 mM NaCl, pH 8.0) and re-pelleted by centrifugation (10,000 rpm, 10 min, 4 °C), then re-suspended in 60 mL of $CaCl_2$ buffer (50 mM TRIS base, 10 mM $CaCl_2$, pH 7.2) containing a tablet of cOmplete™ mini EDTA-free protease inhibitor cocktail. The samples were frozen in liquid nitrogen and stored at −80 °C until purification.

Protein Purification

The samples were thawed on ice and DNase I was added. The cell suspension was then sonicated on ice (5 cycles, 50% amplitude, 30 sec. on, 60 sec. off). The samples were centrifuged (18,000 rpm, 20 min, 4 °C) and the supernatant was discarded. The cell pellet was re-suspended in 40 mL of EDTA buffer (50 mM TRIS base, 20 mM EDTA, pH 7.2) and the cell debris was removed by centrifugation (18,000 rpm, 20 min, 4 °C). The annexin V containing supernatant was filtered through a 0.45 µm syringe filter and dialyzed against 3 × 4 L of TRIS buffer (20 mM TRIS base, pH 7.8). The dialyzed protein solution was purified by FPLC using an anion exchange column (10 mL) and running a gradient from TRIS buffer (20 mM TRIS base, pH 7.8) to NaCl buffer (20 mM TRIS base, 500 mM NaCl, pH 7.8). The purified protein was dialyzed against 3 × 4 L of NaP_i buffer (50 mM NaP_i, pH 8.0). The protein solution was concentrated using a VivaSpin 20 centrifugal concentrator (MWCO 10,000, 4,000 rpm, 10 min, 4 °C) to yield 10 mL of a protein solution. The protein concentration was determined to be 5.40 mg/mL using an A280 spectrophotometer. The expression yield was determined to be 27 mg/L. The protein solution was divided into aliquots, frozen in liquid nitrogen and stored at −80 °C until needed.

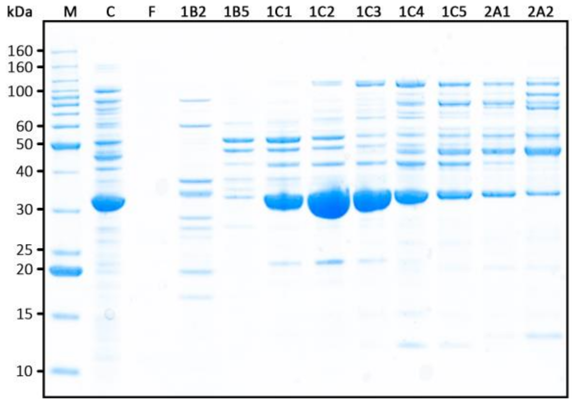

Analysis of purified fractions of Annexin V via SDS-PAGE and coomassie staining (M = BenchMark™ Unstained Protein Ladder, C = crude, F = flow through). A single experiment was deemed sufficient, as no quantification was required.

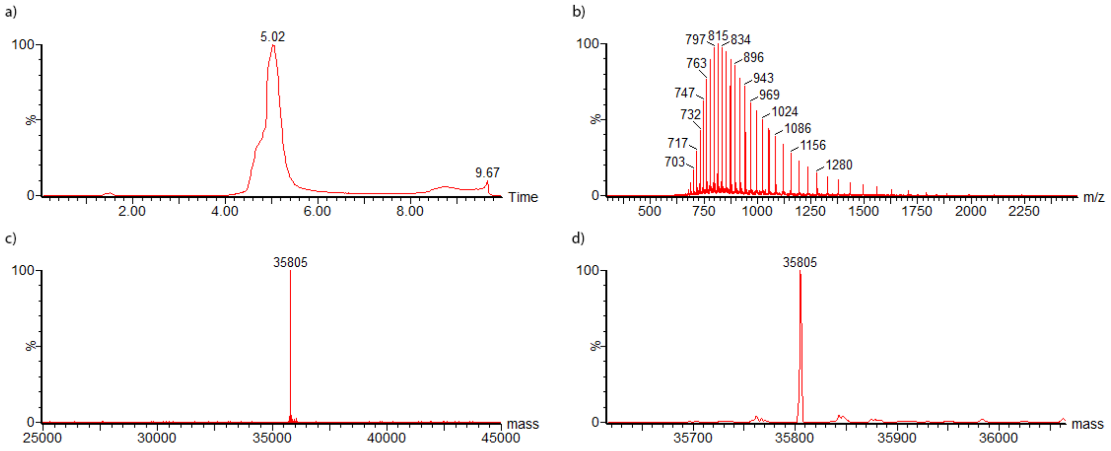

LCMS analysis of Annexin V: a) total ion chromatogram b) ion series c) deconvoluted spectrum d) magnification of the major peak**;** calculated mass: 35806 g/mol; observed mass: 35805 g/mol.

3.4.6 Npβ-Cys61

Plasmid amplification

The plasmid was transformed into Invitrogen One Shot TOP10 chemically competent *E. coli* according to the manufacturer's transformation protocol and grown on agar plates (supplemented with kanamycin) over night. A single colony was selected and transferred to 10 mL of LB media containing 50 µg/mL kanamycin. The culture was incubated at 37 °C for 14 h and the cell pellet was harvested. Plasmid purification was conducted using a QIAprep Spin Miniprep kit and following the manufacturer's instructions.

Transformation and Expression

Protein Expression

T7 Express Competent *E. coli* (New England BioLabs) were transformed with the plasmid following the manufacturer's instructions and grown on agar plates (supplemented with 50 µg/mL kanamycin) over night. Single colonies were selected and transferred to 10 mL of LB media (containing 50 µg/mL kanamycin) each. The starter culture was incubated at 37 °C for 15 h. One starter culture was prepared for every 900 mL of expression media. Two 2.5 L Erlenmeyer flasks charged with 900 mL of LB media supplemented with kanamycin (at final concentration of 30 µg/mL) were inoculated with the starter cultures. The bacterial cultures were incubated at 37 °C with vigorous shaking (250 rpm) an optical density value (OD_{600}) of 0.7 – 0.8 was observed. Protein expression was induced by addition of 1 mM IPTG and the culture was further incubated at 37 °C for 4 h. The cells were harvested by centrifugation (8,000 rpm, 15 min, 4 °C). The cell pellet was suspended in 25 mL of lysis buffer (20 mM imidazole, 20 mM TRIS base, 500 mM NaCl, 2 mM DTT, 5% glycerol, pH 7.8) containing half a tablet of cOmplete™ mini EDTA-free protease inhibitor cocktail. The sample was frozen in liquid nitrogen and stored at −80 °C until purification.

Protein Purification

The samples were thawed on ice and DNase I (2 mg) was added. The cell suspension was then sonicated on ice (10 cycles, 60% amplitude, 15 sec. on, 60 sec. off). The samples

were centrifuged (18,000 rpm, 10 min, 4 °C) and the supernatant was filtered through a 0.45 µm syringe filter and purified via FPLC. The sample was loaded onto a 5 mL GE HisTrap™ HP column at a flow rate of 1 mL/min. The column was washed with 20 CV of buffer A (40 mM imidazole, 20 mM TRIS base, 500 mM NaCl, 2 mM DTT, 5% glycerol, pH 7.8) and the protein was eluted running a linear gradient of 20 CV to 100% buffer B (500 mM imidazole, 20 mM TRIS base, 500 mM NaCl, 2 mM DTT, 5% glycerol, pH 7.8). The fractions were analyzed by SDS-PAGE and clean fractions containing Npβ were pooled together yielding 60 mL of protein solution/ The purified protein solution was dialyzed against 3 × 4 L of NaP_i buffer (50 mM NaP_i, pH 7.8) and concentrated using four VivaSpin 20 centrifugal concentrators (MWCO 10,000, 4,000 rpm, 15 min, 4 °C) to yield 10.3 mL of a protein solution. The protein concentration was determined to be 5.48 mg/mL using an A280 spectrophotometer. The expression yield was determined to be 31 mg/L. The protein solution was divided into aliquots, frozen in liquid nitrogen and stored at −80 °C until needed.

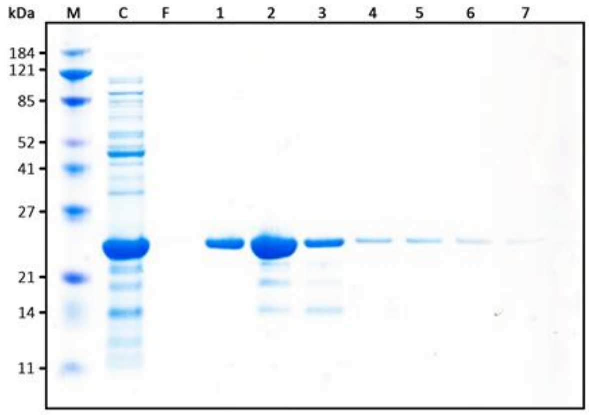

Analysis of purified fractions of Npβ via SDS-PAGE and coomassie staining (M = ProSieve Color Protein Marker, C = crude lysate, F = flow through). A single experiment was deemed sufficient, as no quantification was required.

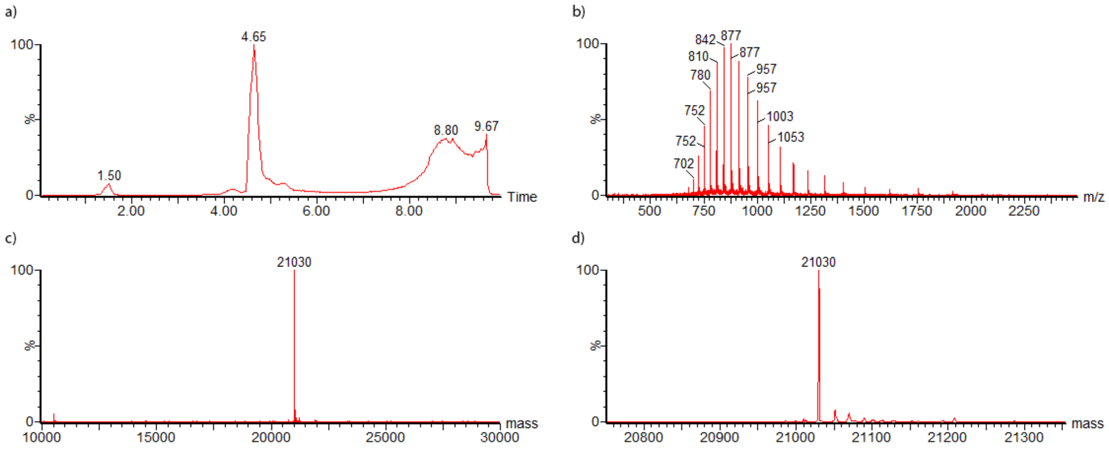

LCMS analysis of Npβ: a) total ion chromatogram b) ion series c) deconvoluted spectrum d) magnification of the major peak**;** calculated mass: 21031 g/mol; observed mass: 21030 g/mol.

3.4.7 pre-SUMO1-Cys51

Plasmid amplification

A pET28a plasmid encoding the gene for pre-SUMO1 was a gift from Guy Salvesen distributed by Addgene (Addgene plasmid #25101) as an agar stab. Bacteria were streaked and grown on agar plates (supplemented with kanamycin) over night. A single colony was selected and transferred to 5 mL of LB media containing 50 µg/mL kanamycin. The culture was incubated at 37°C overnight and the cell pellet was harvested. Plasmid purification was conducted using a QIAprep Spin Miniprep kit (Qiagen) and following the manufacturer's instructions.

Transformation and Expression

Protein Expression

Chemically competent *E. coli* BL21(DE3) pLysS (Agilent) were transformed with pET28a-pre-SUMO1 and plated on agar plates (supplemented with 50 µg/mL kanamycin and 34 µg/mL chloramphenicol) according to the manufacturer's instructions. After overnight incubation, single colonies were transferred to 2 × 10 mL of freshly prepared LB media (supplemented with 50 µg/mL kanamycin and 34 µg/mL chloramphenicol). The starter cultures were incubated overnight and used to inoculate 2 × 900 mL of LB media (supplemented with 50 µg/mL kanamycin and 34 µg/mL chloramphenicol). The cultures were shaken at 37°C (250 rpm) until an optical density value (OD_{600}) of 0.8 – 1.0 was reached. Protein expression was induced by addition of 1 mM IPTG and the cultures were shaken for an additional 3 hours. The cells were harvested by centrifugation (8,500 rpm, 30 min, 4°C). The cell pellets were resuspended in 15 mL of lysis buffer (50 mM NaH_2PO_4, 10 mM imidazole, 300 mM NaCl, 10 mM BME, pH 8.0, one tablet cOmplete™ Mini EDTA-free Protease Inhibitor Cocktail (Roche)), flash frozen in liquid nitrogen and stored at −80°C until purification.

Protein Purification

The samples were thawed and 1 mg of DNase I was added. The suspension was sonicated (10 cycles, 40% amplitude, 15 sec. on, 60 sec. off) and the cell debris was removed by centrifugation (20,000 rpm, 30 min, 4°C). The solution was loaded onto two HisTrap HP 5 mL columns (GE Healthcare) operated in series, the columns were washed with 20 CV of binding buffer (50 mM NaH_2PO_4, 20 mM imidazole, 500 mM NaCl, 10 mM BME, pH 8.0) and eluted with a 20 CV gradient 0-100% elution buffer (50 mM NaH_2PO_4, 500 mM imidazole, 500 mM NaCl, 10 mM BME, pH 8.0). The fractions were analysed by SDS-PAGE, clean fractions were pooled together and dialyzed twice against phosphate buffer supplemented with BME (50 mM NaH_2PO_4, 10 mM BME, pH 8.0) and once phosphate buffer (50 mM NaH_2PO_4, pH 8.0). The protein concentration was determined using an A280 spectrophotometer. The expression yield was determined to be 81.3 mg (45 mg/L expression volume). The protein solution was divided into aliquots, frozen in liquid nitrogen and stored at −80 °C until needed.

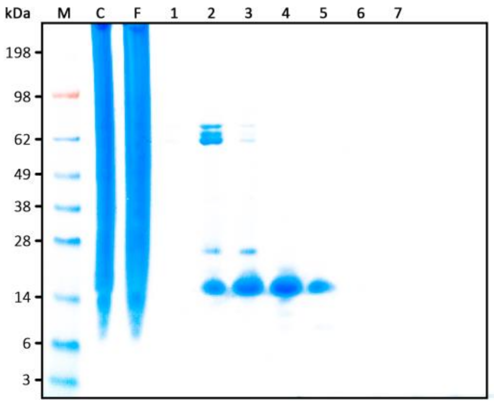

Analysis of purified fractions via SDS-PAGE (MES buffer, 200 V, 40 min, 10% Bis-TRIS gel) and coomassie staining (M = SeeBlue® Plus2 Protein Standard, C = crude lysate, F = flow through). A single experiment was deemed sufficient, as no quantification was required.

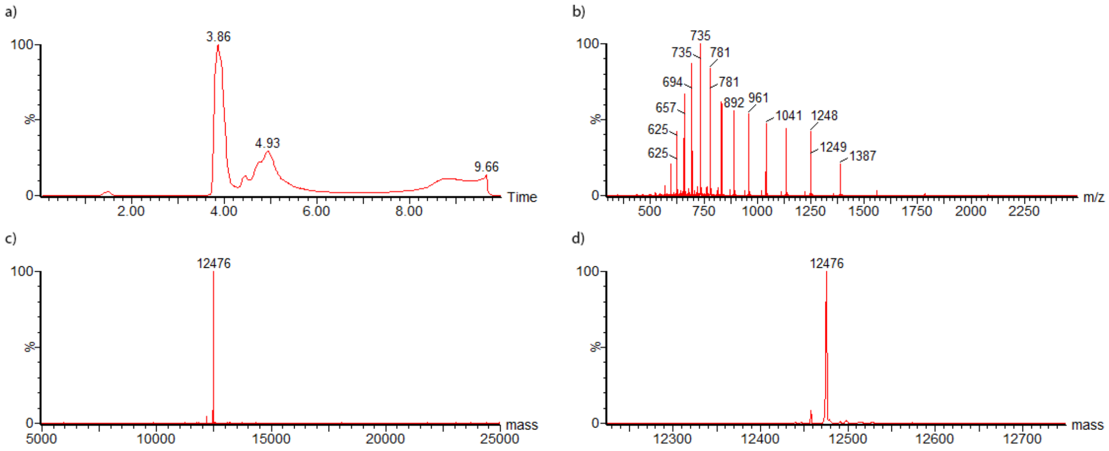

LCMS analysis of preSUMO1-Cys51: a) total ion chromatogram b) ion series c) deconvoluted spectrum d) magnification of the major peak**;** calculated mass: 12475 g/mol; observed masses: 12476 g/mol.

3.4.8 mCherry-Cys131

Transformation and Expression

Protein Expression

E. coli SG13009 (Qiagen) carrying the plasmid pQE-9-mCherryS131C was kindly donated by Bradley Olsen and distributed by Addgene (Addgene plasmid #48732). Bacteria were streaked on agar plates (supplemented with 50 µg/mL kanamycin and 100 µg/mL ampicillin) and grown overnight. Single colonies were transferred to 2 × 10 mL freshly prepared terrific broth (TB media) supplemented with 50 µg/mL kanamycin and 100 µg/mL ampicillin and incubated at 37°C (250 rpm) for 16 hours. 2 × 900 mL TB media (containing 50 µg/mL kanamycin, 100 µg/mL ampicillin) were inoculated with the previously prepared starter cultures. The cultures were shaken at 37°C (200 rpm) until an optical density value (OD_{600}) of 1.0 – 1.2 was reached. Protein expression was induced by addition of 1 mM ITPG and the cultures were shaken for another 5 hours. The cells were harvested by centrifugation (9,000 rpm, 20 min, 4°C), suspended in 12.5 mL of lysis buffer each (50 mM NaH_2PO_4, 10 mM imidazole, 300 mM NaCl, 10 mM BME, pH 8.0, one tablet cOmplete™ Mini EDTA-free Protease Inhibitor Cocktail (Roche)) and frozen in liquid nitrogen.

Protein Purification

The samples were thawed and 1 mg of DNase I was added. The suspension was sonicated (5 cycles, 40% amplitude, 30 sec. on, 60 sec. off) and the cell debris was removed by centrifugation (20,000 rpm, 20 min, 4°C). The solution was loaded onto two HisTrap HP 5 mL columns (GE Healthcare) operated in series, the columns were washed with 20 CV of binding buffer (50 mM NaH_2PO_4, 20 mM imidazole, 500 mM NaCl, 10 mM BME, pH 8.0) and eluted with a 20 CV gradient 0-100% elution buffer (50 mM NaH_2PO_4, 500 mM imidazole, 500 mM NaCl, 10 mM BME, pH 8.0). The fractions were analysed by SDS-PAGE, clean fractions were pooled together and dialyzed three times against phosphate buffer supplemented with BME (50 mM NaH_2PO_4, 10 mM BME, pH 8.0). The protein concentration was determined using an A280 spectrophotometer. The expression yield was determined to be 294 mg (163 mg/L expression volume). The protein solution was divided into aliquots, frozen in liquid nitrogen and stored at −80 °C until needed.

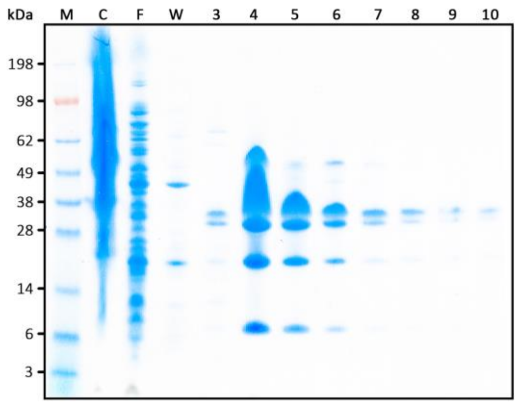

Analysis of purified fractions via SDS-PAGE (MES buffer, 200 V, 40 min, 10% Bis-TRIS gel) and coomassie staining (M = SeeBlue® Plus2 Protein Standard, C = crude lysate, F = flow through, W = wash). A single experiment was deemed sufficient, as no quantification was required.

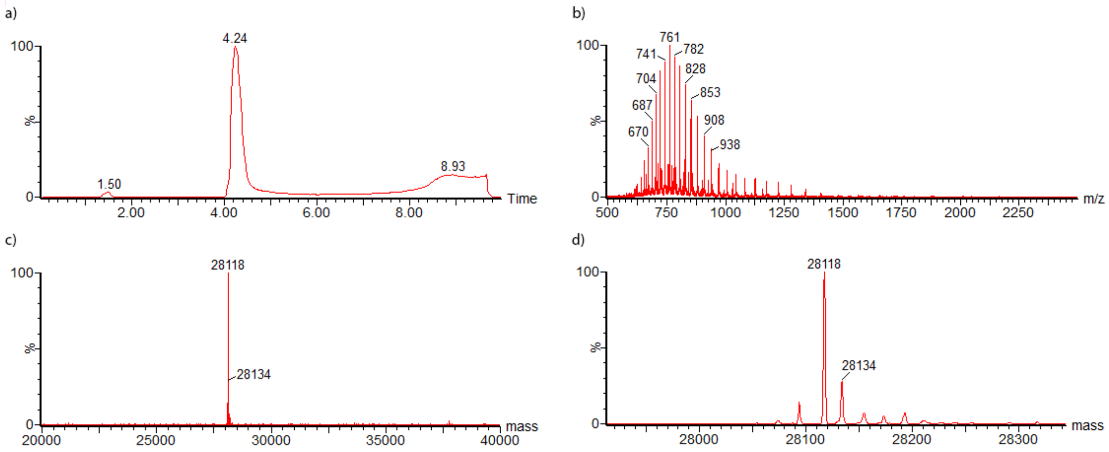

LCMS analysis of mCherry-Cys131: a) total ion chromatogram b) ion series c) deconvoluted spectrum d) magnification of the major peak**;** calculated masses: 28137 g/mol, 28117 g/mol (matured mCherry-Cys131); observed masses: 28134 g/mol, 28118 g/mol (matured mCherry-Cys131).

3.4.9 PstS-Cys197

Plasmid amplification

E. coli DH5α carrying the plasmid pET22b-PstSA197C was kindly donated by Martin Webb and distributed by Addgene (Addgene plasmid #78198) as an agar stab. Bacteria were streaked on agar plates (supplemented with 100 µg/mL ampicillin) and grown overnight. A single colony was selected and transferred to 5 mL of LB media containing 100 µg/mL ampicillin. The culture was incubated at 37°C overnight and the cell pellet was harvested. Plasmid purification was conducted using a QIAprep Spin Miniprep kit (Qiagen) and following the manufacturer's instructions.

Transformation and Expression

Protein Expression

Chemically competent *E. coli* BL21(DE3) (Agilent) were transformed with pET22b-PstS-A197C and plated on agar plates (supplemented with 100 µg/mL ampicillin) according to the manufacturer's instructions. Single colonies were transferred to 2 × 10 mL freshly prepared LB media supplemented with 100 µg/mL ampicillin and incubated at 37°C (250 rpm) for 16 hours. 2 × 900 mL LB media (containing 100 µg/mL ampicillin) were inoculated with the previously prepared starter cultures. The cultures were shaken at 37°C (200 rpm) until an optical density value (OD_{600}) of 0.6 – 0.8 was reached. Protein expression was induced by addition of 1 mM ITPG and the cultures were shaken for another 4 hours. The cells were harvested by centrifugation (9,000 rpm, 20 min, 4°C), suspended in 12.5 mL of lysis buffer each (10 mM TRIS base, 1 mM DTT, pH 8.0, one tablet cOmplete™ Mini EDTA-free Protease Inhibitor Cocktail (Roche)) and frozen in liquid nitrogen.

Protein Purification

The samples were thawed and 1 mg of DNase I was added. The suspension was sonicated (10 cycles, 40% amplitude, 15 sec. on, 60 sec. off) and the cell debris was removed by centrifugation (30,000 rpm, 30 min, 4°C). The solution was loaded onto a HiTrap Q HP column (5 mL) (GE Healthcare) and washed with 10 CV of binding buffer (10 mM TRIS

base, 1 mM DTT, pH 8.0) and eluted with a 20 CV gradient 0−100% elution buffer (10 mM TRIS base, 200 mM NaCl, 1 mM DTT, pH 8.0). The fractions were analysed by SDS-PAGE and clean fractions were pooled together. The protein concentration was determined using an A280 spectrophotometer. The expression yield was determined to be 13.7 mg (15.2 mg/L expression volume). The protein solution was divided into aliquots, frozen in liquid nitrogen and stored at −80 °C until needed.

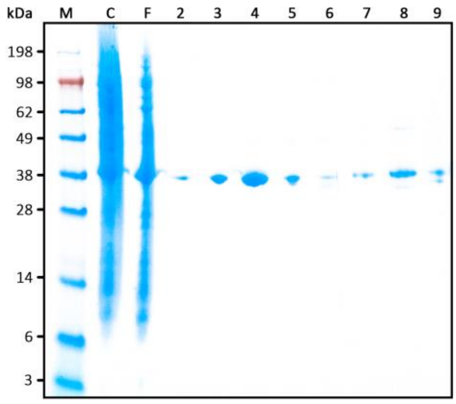

Analysis of purified fractions via SDS-PAGE (MES buffer, 200 V, 40 min, 10% Bis-TRIS gel) and coomassie staining (M = SeeBlue® Plus2 Protein Standard, C = crude lysate, F = flow through, W = wash). A single experiment was deemed sufficient, as no quantification was required.

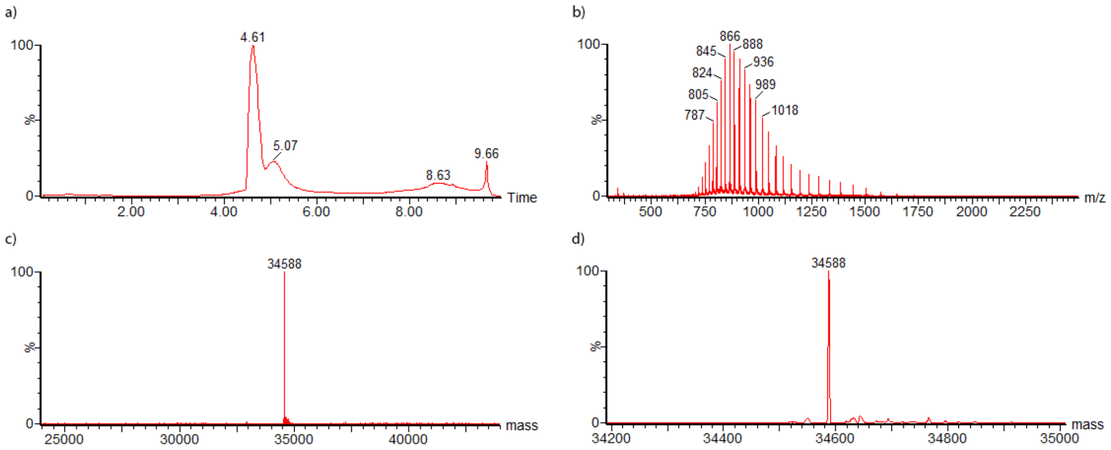

LCMS analysis of PstS-Cys197: a) total ion chromatogram b) ion series c) deconvoluted spectrum d) magnification of the major peak; calculated mass: 34585 g/mol; observed mass: 34588 g/mol.

3.4.10 panC

PanC-Cys44 and panC-Cys47 were expressed and purified following a previously published procedure.[21]

3.4.11 AcrA-Cys123

AcrA-Cys123 was expressed and purified following a previously published procedure.[22]

3.4.12 Histone H4-Cys16

Histone H4-Cys16 was expressed and purified following a previously published procedure.[22]

3.4.13 Histone H3-Cys10

3.4.13.1 Histone H3-Dha10

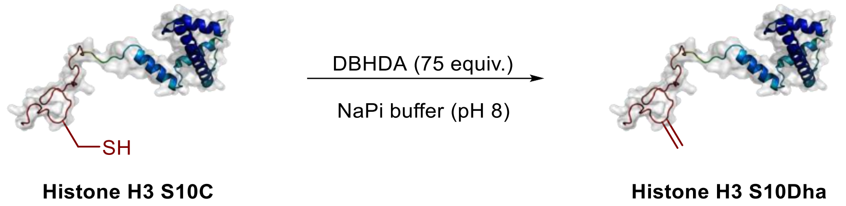

Histone H3-Cys10 (11.4 mg, 0.75 µmol, 1.00 equiv.) and DTT (30 mg, 194 µmol, 260 equiv.) were dissolved in 500 µL of NaP_i buffer (100 mM, 3 M Gdn·HCl, pH 8.0) by sonication. The solution was incubated at room temperature for 1 h, before it was desalted by passing through a GE MiniTrap G-25 desalting column pre-equilibrated with NaP_i buffer (100 mM, 3 M Gdn·HCl, pH 8.0) according to the manufacturer's instructions. To the solution was added a solution of DBHDA (18.0 mg, 59.8 µmol, 80 equiv.) in DMF (100 µL). The mixture was vortexed and gently shaken at room temperature for 75 min. until no starting material could be detected anymore by LCMS. The temperature was raised to 37 °C and the reaction mixture was shaken for an additional 2.5 h. The protein was desalted by passing through a GE MiniTrap G-25 column pre-equilibrated with NaP_i buffer (100 mM, 3 M Gdn·HCl, pH 8.0) to yield 2.00 mL of a protein solution with a concentration of 4.58 mg/mL as determined by A280 spectrophotometry (9.16 mg, 0.60 µmol, 80%).

The sample was aliquoted, frozen in liquid nitrogen and stored at −80°C.

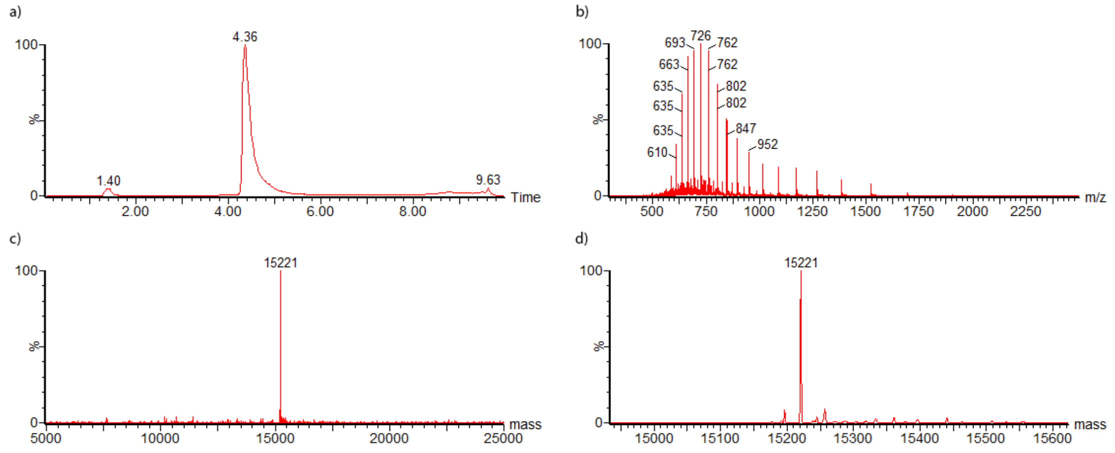

LCMS analysis of Histone H3-Dha10: a) total ion chromatogram b) ion series c) deconvoluted spectrum d) magnification of the major peak**;** calculated mass: 15221 g/mol, observed mass: 15221 g/mol.

3.4.13.2 Histone H3-Bal10

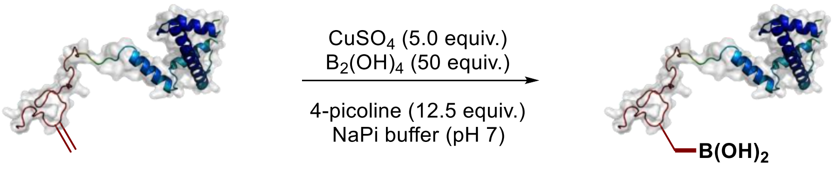

Histone H3 S10Dha **Histone H3 S10Bal**

Stock solutions of $CuSO_4 \cdot 5\ H_2O$ (11.8 mg in 1.00 mL of H_2O), 4-picoline (11.5 µL in 1.00 mL of H_2O) and $B_2(OH)_4$ (14.1 mg in 1.00 mL of H_2O) were freshly prepared before the reaction was conducted.

To a solution of Histone H3-Dha10 (500 µL, 2.87 mg/mL, 94.3 mnol, 1.00 equiv) NaP_i buffer (100 mM, 3 M Gdn·HCl, pH 7.2) were added previously prepared stock solutions of 4-picoline (10.0 µL, 1.18 µmol, 12.5 equiv.), $CuSO_4$ (10.0 µL, 471 nmol, 5.00 equiv.) and $B_2(OH)_4$ (30.0 µL, 4.71 µmol, 50.0 equiv.) subsequently. The mixture was vortexed and shaken for 30 min at room temperature. The protein was desalted by passing through a GE MiniTrap G-25 column pre-equilibrated with NaP_i buffer (100 mM, 3 M Gdn·HCl, pH 8.0) according to the manufacturer's instructions. The solution dialyzed against NaP_i buffer (100 mM, pH 8.0) to remove any residual boric acid yielding 1000 µL of a solution of Histone H3-Bal10 with a concentration of 1.31 mg/mL as determined by A280 spectrophotometry (1.31 mg, 85.8 nmol, 91% recovery, up to 98% conversion).

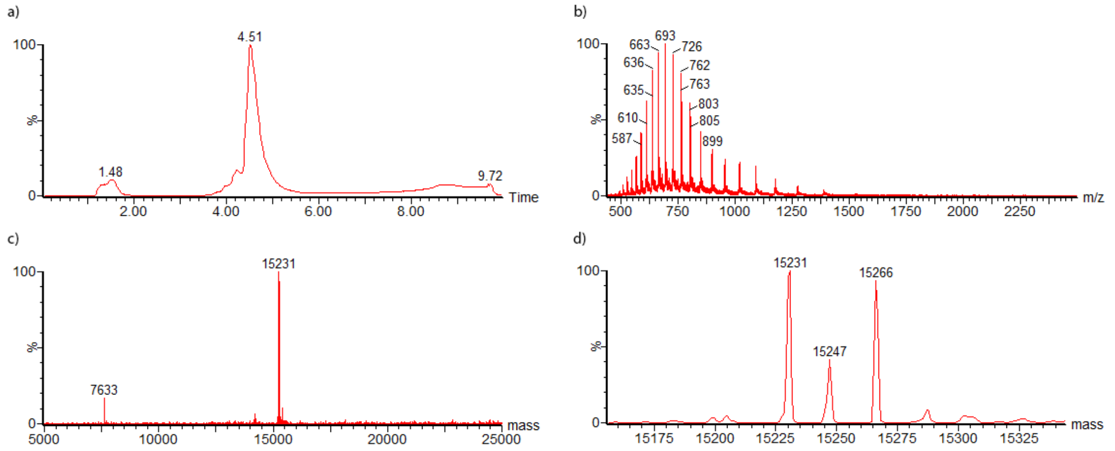

LCMS analysis of Histone H3-Bal10: a) total ion chromatogram b) ion series c) deconvoluted spectrum d) magnification of the major peak; calculated masses: 15267 g/mol (Bal), 15249 g/mol (Bal−H_2O), 15231 g/mol (Bal−$2H_2O$); observed masses: 15266 g/mol (Bal), 15247 g/mol (Bal−H_2O), 15231 g/mol (Bal−$2H_2O$).

3.4.13.3 Histone H3-Ser10

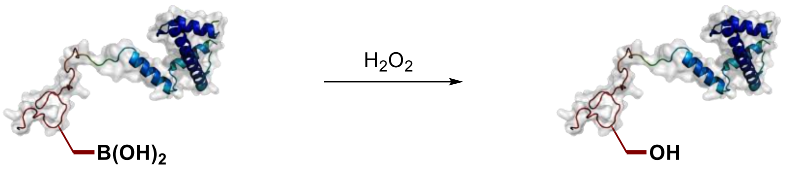

Histone H3 S10Bal **Histone H3 S10S**

To a solution of Histone H3-Bal10 (200 µL, 1.31 mg/mL, 261 µg, 17 nmol, 1.00 equiv.) in NaP_i buffer (100 mM, 3 M Gdn·HCl, pH 7.2) were added 10 µL of a solution of H_2O_2 in water (5.22 µmol, 20 equiv). The mixture was vortexed and shaken for 10 min at room temperature. The solution was dialyzed against NaP_i buffer (100 mM, pH 8.0) to remove any residual boric acid or H_2O_2 yielding 350 µL of a solution of Histone H3-Ser10 with a concentration of 0.70 mg/mL as determined by A280 spectrophotometry (245 µg, 16 nmol, 94%).

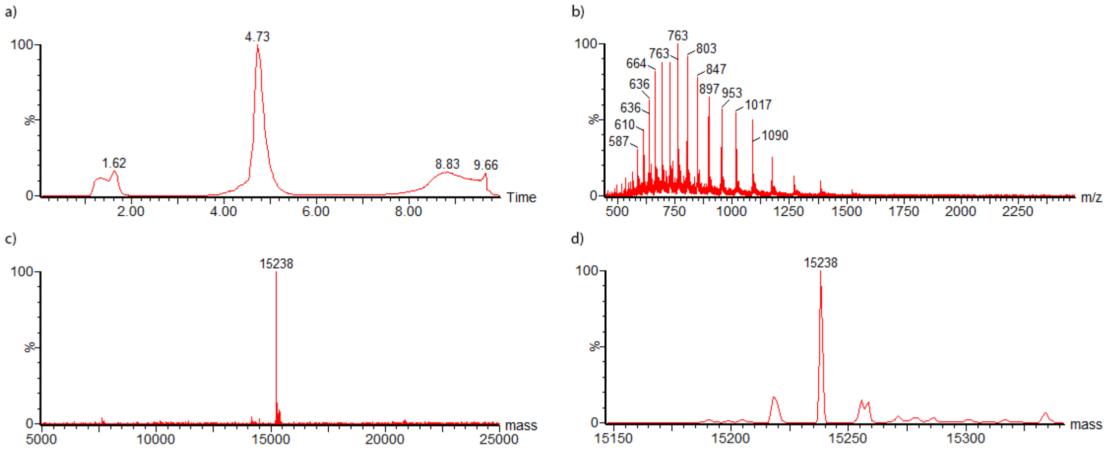

LCMS analysis of Histone H3-Ser10: a) total ion chromatogram b) ion series c) deconvoluted spectrum d) magnification of the major peak**;** calculated mass: 15239 g/mol; observed mass: 15238 g/mol.

3.4.13.4 Deuteration Study

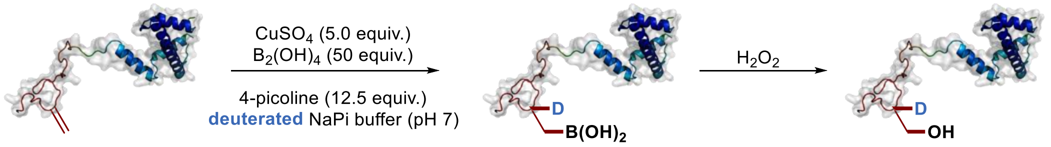

Histone H3 S10Dha **Histone H3 S10dBal** **Histone H3 S10dS**

A solution of histone H3-Dha10 (250 µL, 0.78 mg/mL) in NaP_i buffer (100 mM, 3 M Gdn·HCl, pH 7.0) was diluted with 750 µL D_2O and flash frozen in liquid N_2. The mixture was lyophilized, redissolved in 1000 µL of D_2O, shaken at room temperature for 10 min and flash frozen. This step was repeated twice before the protein solution was reconstituted with 250 µL of D_2O. Borylation was executed as described for Histone H3-Bal10, while all reactant solutions were prepared in D_2O. The reaction mixture was purified using a GE MiniTrap G-25 column and the purified protein was concentrated using VivaSpin 500 concentrators (5000 Da MWCO). The resulting solution was split in half and one aliquot was oxidized. Oxidation was performed with H_2O_2 (30 equiv.) as reported previously. Both aliquots were then dialyzed against NaP_i buffer (100 mM, pH 8.0).

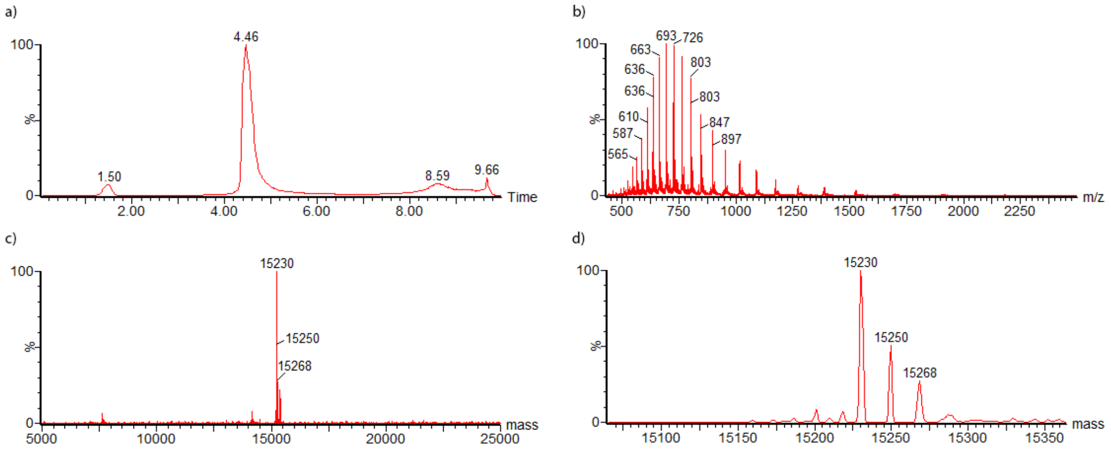

LCMS analysis of Histone H3-dBal10: a) total ion chromatogram b) ion series c) deconvoluted spectrum d) magnification of the major peak**;** calculated masses: 15268 g/mol (Bal), 15250 g/mol (Bal−H_2O), 15232 g/mol (Bal−2H_2O); observed masses: 15268 g/mol (Bal), 15250 g/mol (Bal−H_2O), 15230 g/mol (Bal−2H_2O).

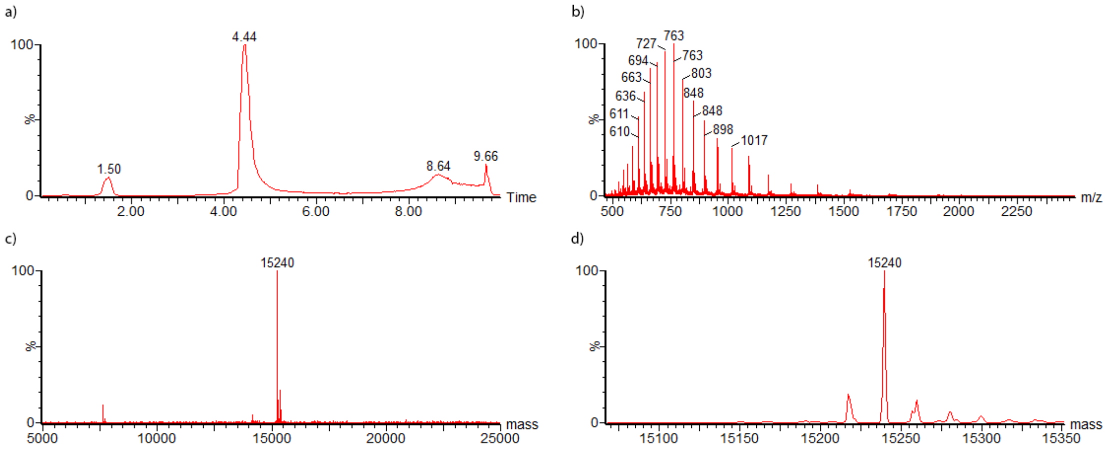

LCMS analysis of Histone H3-dSer10: a) total ion chromatogram b) ion series c) deconvoluted spectrum d) magnification of the major peak**;** calculated mass: 12240 g/mol; observed mass: 12240 g/mol.

3.4.14 [15N]Histone H3-Cys10

3.4.14.1 [15N]Histone H3-Dha10

[15N]Histone H3 Dha10 was synthesized following the procedure for Histone H3 Dha10 (see section 3.5.1.1). Full conversion was observed via LCMS analysis.

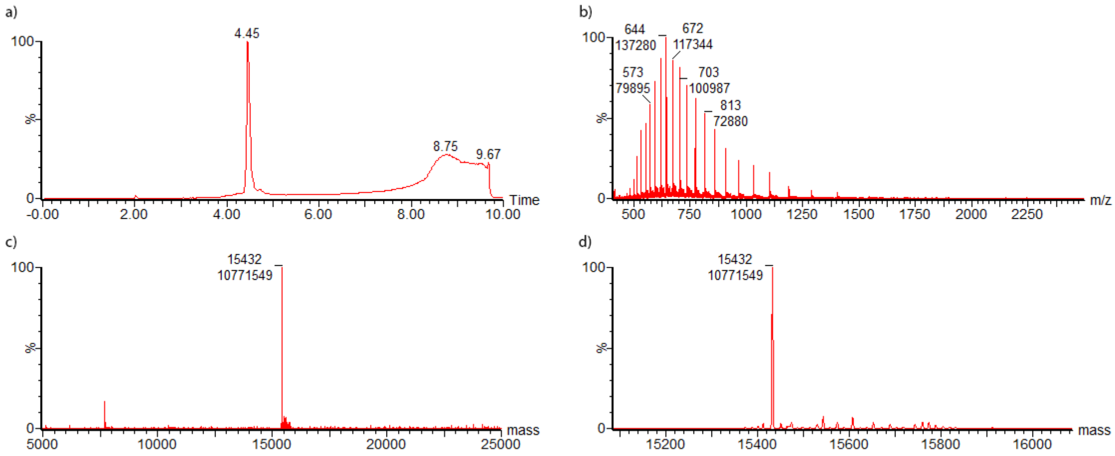

LCMS analysis of [15N]Histone H3-Dha10: a) total ion chromatogram b) ion series c) deconvoluted spectrum d) magnification of the major peak; calculated mass: 15434 g/mol; observed mass: 15432 g/mol.

3.4.14.2 [15N]Histone H3-Bal10

[15N]Histone H3 Bal10 was synthesized following the procedure for Histone H3 Bal10 (see section 3.5.1.2). To 3 × 1 mL of [15N]Histone H3 Dha10 (2.04 mg/mL, 402 nmol) in borylation buffer (100 mM NaP_i, 3 M Gdn·HCl, pH 7.0) were each added 4-picoline (12.5 equiv), $CuSO_4$ (5.0 equiv) and $B_2(OH)_4$ (50.0 equiv).

Full conversion was observed via LCMS analysis. 95% conversion was observed via LCMS analysis.

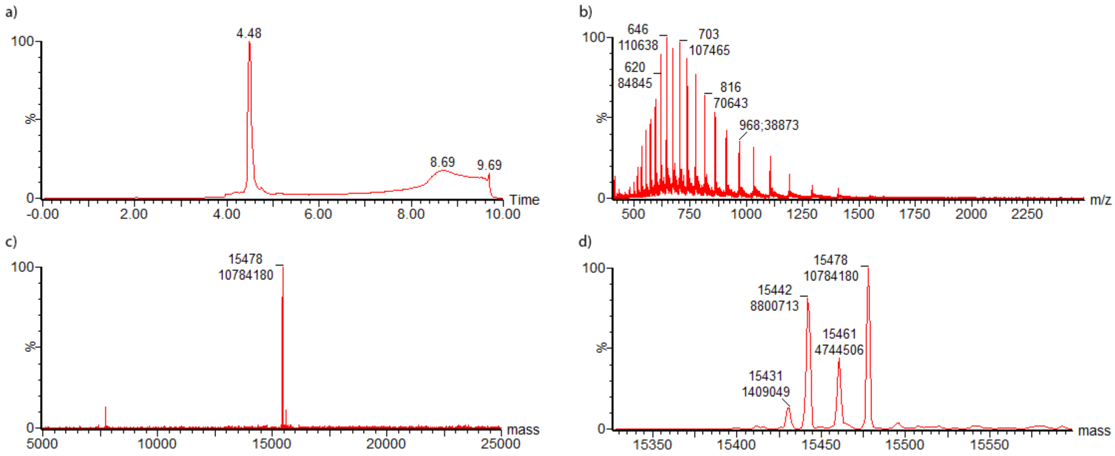

LCMS analysis of [15N]Histone H3-Bal10: a) total ion chromatogram b) ion series c) deconvoluted spectrum d) magnification of the major peak**;** calculated masses: 15480 g/mol (Bal), 15462 g/mol (Bal−H_2O), 15444 g/mol (Bal−2H_2O); observed masses: 15278 g/mol (Bal), 15461 g/mol (Bal−H_2O), 15442 g/mol (Bal−2H_2O).

3.4.14.3 [15N]Histone H3-Ser10

[15N]Histone H3 Ser10 was synthesized by incubation of Histone H3 Bal10 (approx. 4 mg/mL) with 10 mM H_2O_2 for 30 min in unfolding buffer (7 M Gdn·HCl, 10 mM TRIS base, 1 mM EDTA, 10 mM DTT, 1 mM benzamidine).

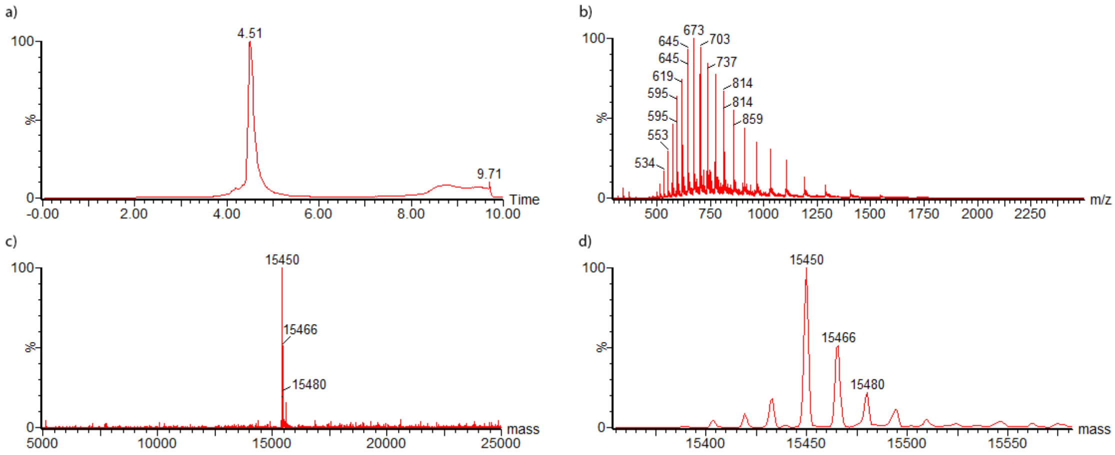

LCMS analysis of [15N]Histone H3-Ser10: a) total ion chromatogram b) ion series c) deconvoluted spectrum d) magnification of the major peak**;** calculated mass: 15452 g/mol; observed masses: 15450 g/mol, 15466 g/mol (+ 1 × oxidation), 15480 g/mol (+ 2 × oxidation).

3.4.15 [^{13}C-^{15}N]Histone H3-Cys10

3.4.15.1 [^{13}C-^{15}N]Histone H3-Dha10

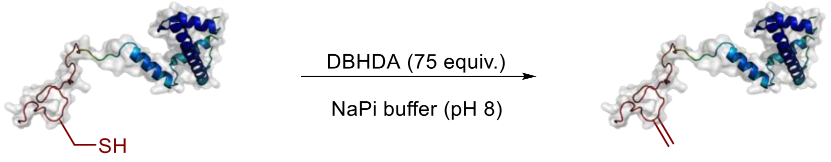

[^{13}C-^{15}N]Histone H3 S10C **[^{13}C-^{15}N]Histone H3 S10Dha**

[^{13}C-^{15}N]Histone H3-Cys10 (2.5 mg, 164 nmol) was dissolved in 330 µL of NMR buffer (50 mM NaP_i, 3 M Gdn·HCl, 5% D_2O). After completed NMR measurements, the sample was incubated with DTT (2.5 mg, 16.4 µmol, 100 equiv.) for 1 h. The sample was desalted by passing through a GE MidiTrap G-25 desalting column pre-equilibrated with NaP_i buffer (100 mM, 3 M Gdn·HCl, pH 8.0) according to the manufacturer's instructions. To the solution was added a solution of DBHDA (3.7 mg, 12.3 µmol, 75 equiv.) in DMF (80 µL), the mixture was vortexed and shaken (500 rpm) at room temperature for 45 min. The temperature was increased to 37°C and shaking was continued for 2 h. The reaction mixture was purified by passing through two GE MidiTrap G-25 desalting columns pre-equilibrated with NaP_i buffer (100 mM, 3 M Gdn·HCl, pH 8.0). The sample was then dialyzed against milli-Q water (3 × 4 L) using a dialysis cassette (MWCO 3,500 Da) and lyophilized. The lyophilized powder was redissolved in deuterated NaP_i buffer (1600 µL) and the concentration was determined as 1.20 mg/mL by A_{280} spectrophotometry (1.92 mg, 126 nmol, 77% recovery).

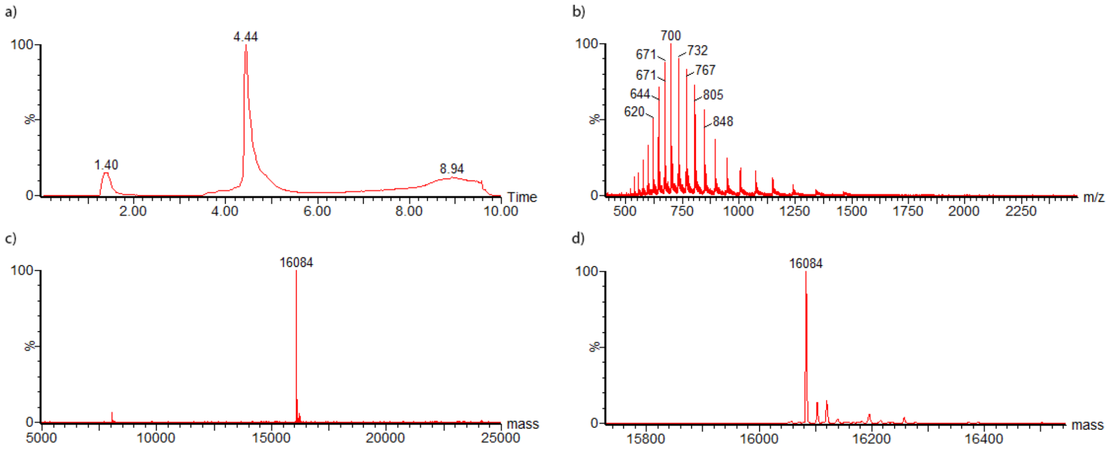

LCMS analysis of [^{13}C-^{15}N]Histone H3-Dha10: a) total ion chromatogram b) ion series c) deconvoluted spectrum d) magnification of the major peak; calculated mass: 16083 g/mol ([^{13}C-^{15}N]Histone H3-Dha10); observed mass: 16084 g/mol ([^{13}C-^{15}N]Histone H3-Dha10).

3.4.15.2 [^{13}C-^{15}N]Histone H3-dBal10

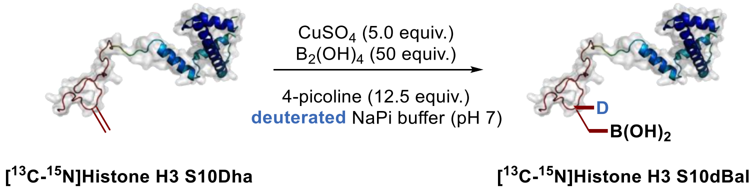

Deuterated NaP$_i$ buffer was produced using the following procedure: NaP$_i$ buffer (100 mM NaP$_i$, 3 M Gdn·HCl, pH 7.0) was frozen in liquid nitrogen and lyophilized. The lyophilized power was reconstituted with D_2O and frozen and lyophilized again. The lyophilized power was then reconstituted with D_2O and used to dissolve previously lyophilized [^{13}C-^{15}N]Histone H3-Dha10.

Stock solutions of $CuSO_4 \cdot 5\,H_2O$ (15.7 mg in 1.00 mL of H_2O), 4-picoline (15.4 µL in 1.00 mL of H_2O) and $B_2(OH)_4$ (3.4 mg in 1.00 mL of H_2O) were freshly prepared before the reaction was conducted.

To solution of [^{13}C-^{15}N]Histone H3-Dha10 in deuterated NaP$_i$ buffer (1600 µL, 1.20 mg/mL, 1.92 mg, 126 nmol) were added previously prepared stock solutions of 4-picoline (10 µL, 1.57 µmol, 12.5 equiv.), $CuSO_4$ (10 µL, 0.63 µmol, 5.00 equiv.) and $B_2(OH)_4$ (130 µL, 6.30 µmol, 50 equiv.). The mixture was incubated at room temperature

for 30 min before being purified using two GE MidiTrap G-25 columns pre-equilibrated with NaP_i buffer (100 mM, 3 M Gdn·HCl, pH 8.0) according to the manufacturer's instructions. The sample was then dialyzed against milli-Q water (3 × 4 L) using a dialysis cassette (MWCO 3,500 Da) and lyophilized. The lyophilized power was dissolved in 330 µL of NMR buffer (50 mM NaP_i, 3 M Gdn·HCl, 5% D_2O). The concentration of the sample was determined at 5.04 mg/mL by A_{280} photospectrometry (1.66 mg, 109 nmol, 86% recovery).

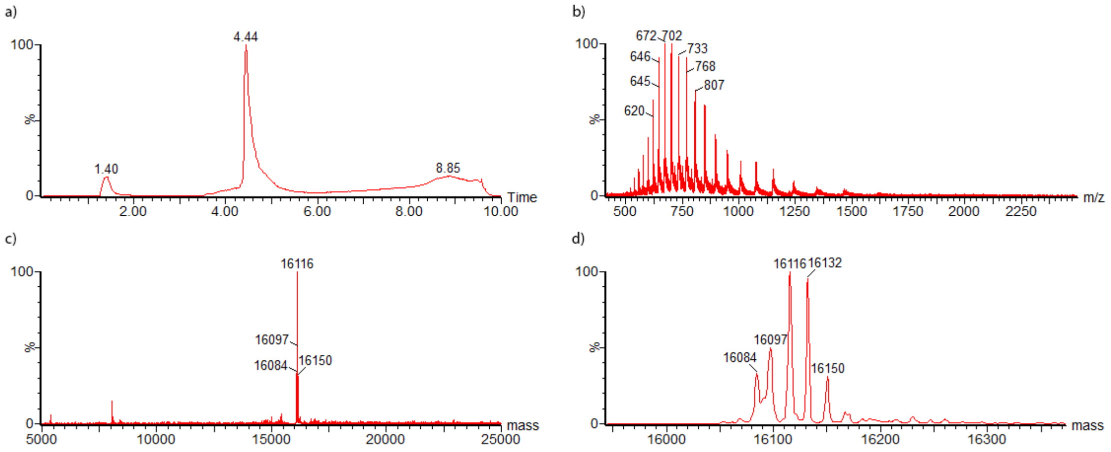

LCMS analysis of [^{13}C-^{15}N]Histone H3-dBal10: a) total ion chromatogram b) ion series c) deconvoluted spectrum d) magnification of the major peak**;** calculated masses: 16083 g/mol ([^{13}C-^{15}N]Histone H3-Dha10), 16094 g/mol ([^{13}C-^{15}N]Histone H3-dBal10−2H_2O), 16112 g/mol ([^{13}C-^{15}N]Histone H3-dBal10−H_2O), 16130 g/mol ([^{13}C-^{15}N]Histone H3-dBal10); observed masses: 16084 g/mol ([^{13}C-^{15}N]Histone H3-Dha10), 16097 g/mol ([^{13}C-^{15}N]Histone H3-dBal10−2H_2O), 16116 g/mol ([^{13}C-^{15}N]Histone H3-dBal10−H_2O), 16132 g/mol ([^{13}C-^{15}N]Histone H3-dBal10).

3.4.16 Histone H3-Cys9

3.4.16.1 Histone H3-Dha9

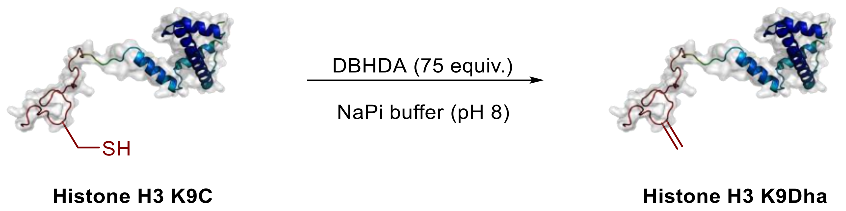

Histone H3-Cys9 (15.0 mg, 0.99 µmol, 1.00 equiv.) and DTT (37.5 mg, 243 µmol, 250 equiv.) were dissolved in 1000 µL of NaP_i buffer (100 mM, 3 M Gdn·HCl, pH 8.0) by repeated vortexing and sonication. The solution was incubated at room temperature for 1 h before being desalted using GE MiniTrap G-25 desalting columns preconditioned with NaP_i buffer (100 mM, 3 M Gdn·HCl, pH 8.0) according to the manufacturer's specifications. A solution of DBHDA (23.8 mg, 78.9 µmol, 80 equiv.) in 90 µL of DMF was added and the mixture was briefly vortexed and shaken at room temperature for 2 h. The temperature was raised to 37°C and shaking was continued for an additional 2.5 h until completion of the reaction was determined by LCMS. The solution was purified using GE MiniTrap G-25 columns preconditioned with NaP_i buffer (100 mM, 3 M Gdn·HCl, pH 8.0) to yield 4000 µL of a protein solution with a concentration of 2.54 mg/mL as determined by A280 spectrophotometry (10.16 mg, 0.67 µmol, 68%).

The sample was aliquoted, frozen in liquid nitrogen and stored at −80°C.

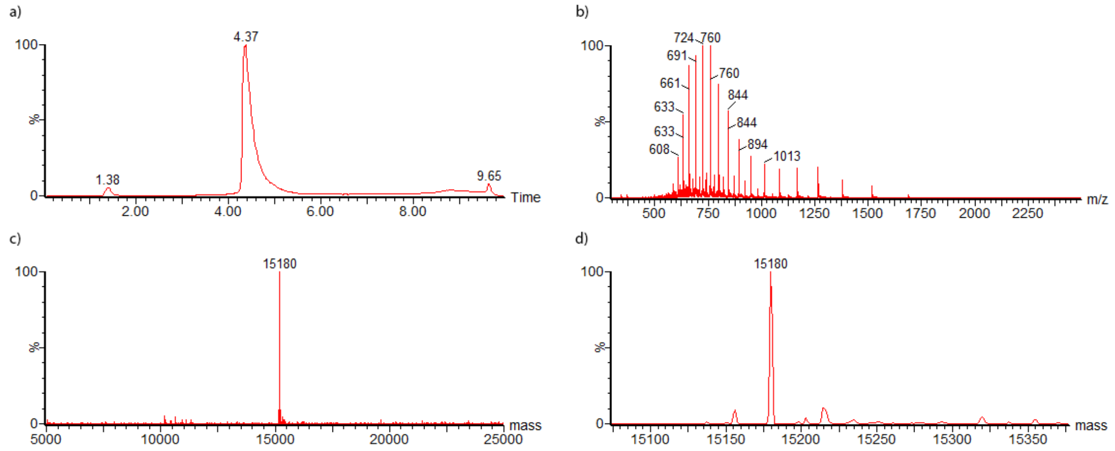

LCMS analysis of Histone H3-Dha9: a) total ion chromatogram b) ion series c) deconvoluted spectrum d) magnification of the major peak**;** calculated mass: 15180 g/mol; observed mass: 15180 g/mol.

3.4.16.2 Histone H3-Bal9

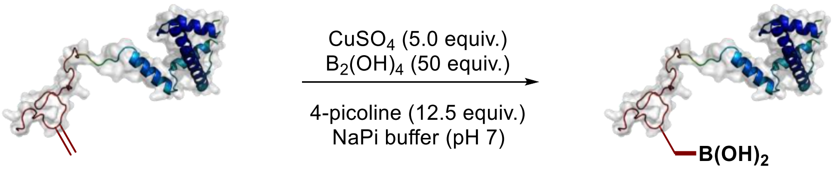

Histone H3 K9Dha **Histone H3 K9Bal**

Stock solutions of $CuSO_4 \cdot 5\ H_2O$ (2.35 mg in 1.00 mL of H_2O), 4-picoline (2.29 µL in 1.00 mL of H_2O) and $B_2(OH)_4$ (2.81 mg in 1.00 mL of H_2O) were freshly prepared before the reaction was conducted.

To a solution of Histone H3-Dha9 (500 µL, 2.54 mg/mL, 1.27 mg, 84 nmol, 1.00 equiv.) in NaP_i buffer (100 mM, 3 M Gdn·HCl, pH 7.2) were added previously prepared stock solutions of 4-picoline (10.0 µL, 1.05 µmol, 12.5 equiv.), $CuSO_4$ (10.0 µL, 418 nmol, 5.00 equiv.) and $B_2(OH)_4$ (30.0 µL, 4.18 µmol, 50.0 equiv.) subsequently. The mixture was vortexed and shaken for 30 min at room temperature. The protein was desalted by passing through a GE MiniTrap G-25 column pre-equilibrated with NaP_i buffer (100 mM, 3 M Gdn·HCl, pH 8.0) according to the manufacturer's instructions. The solution dialyzed against NaP_i buffer (100 mM, pH 8.0) to remove any residual boric acid yielding 1000 µL of a solution of Histone H3-Bal9 with a concentration of 1.11 mg/mL as determined by A280 spectrophotometry (0.89 mg, 72 nmol, 86% recovery, up to 73% conversion).

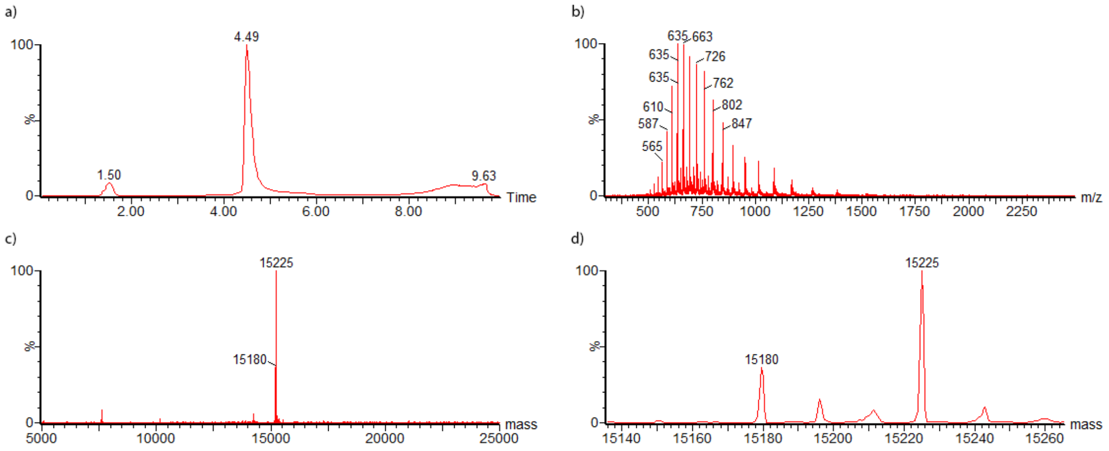

LCMS analysis of Histone H3-Bal9: a) total ion chromatogram b) ion series c) deconvoluted spectrum d) magnification of the major peak**;** calculated mass: 15226 g/mol (Bal); observed mass: 15225 g/mol (Bal).

3.4.17 [^{15}N]Histone H3-Cys9

3.4.17.1 [^{15}N]Histone H3-Dha9

[^{15}N]Histone H3 Dha9 was synthesized following the procedure for Histone H3 Dha9 (see section 3.5.4.1). Full conversion was observed via LCMS analysis.

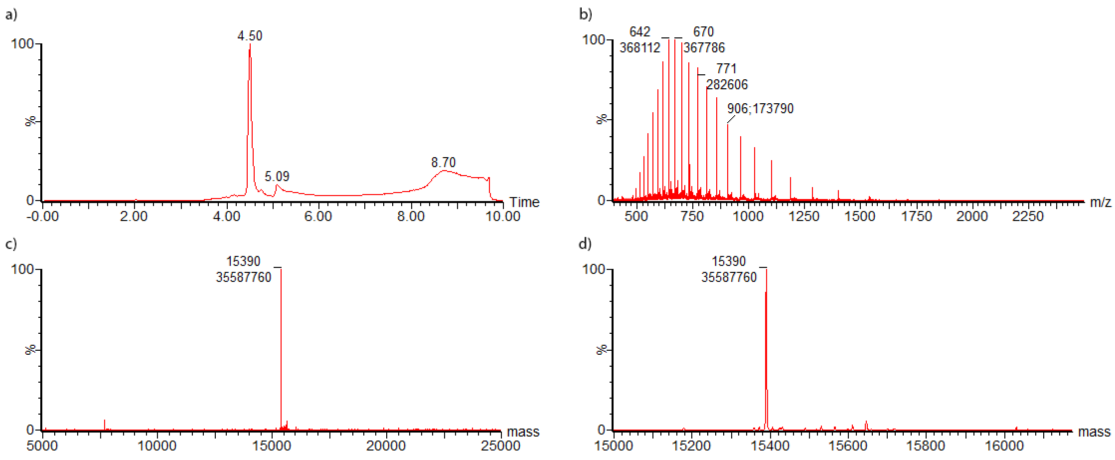

LCMS analysis of [^{15}N]Histone H3-Dha9: a) total ion chromatogram b) ion series c) deconvoluted spectrum d) magnification of the major peak; calculated mass: 15392 g/mol; observed mass: 15390 g/mol.

3.4.17.2 [^{15}N]Histone H3-Bal9

[^{15}N]Histone H3 Bal9 was synthesized following the procedure for Histone H3 Bal9 (see section 3.5.4.2). To 3 × 1 mL of [^{15}N]Histone H3 Dha9 (1.75 mg/mL, 346 nmol) in borylation buffer (100 mM NaP_i, 3 M Gdn·HCl, pH 7.0) were each added 4-picoline (12.5 equiv), $CuSO_4$ (5.0 equiv) and $B_2(OH)_4$ (50.0 equiv).

Full conversion was observed via LCMS analysis.

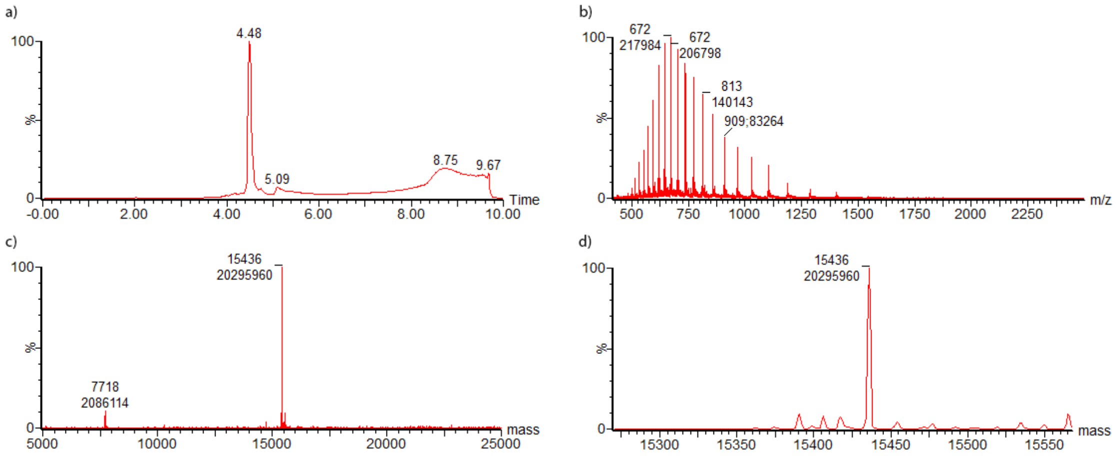

LCMS analysis of [^{15}N]Histone H3-Bal9: a) total ion chromatogram b) ion series c) deconvoluted spectrum d) magnification of the major peak**;** calculated mass: 15438 g/mol; observed mass: 15436 g/mol.

3.4.17.3 [^{15}N]Histone H3-Ser9er

[^{15}N]Histone H3 Ser9 was synthesized by incubation of Histone H3 Bal9 (approx. 4 mg/mL) with 10 mM H_2O_2 for 30 min in unfolding buffer (7 M Gdn·HCl, 10 mM TRIS base, 1 mM EDTA, 10 mM DTT, 1 mM benzamidine).

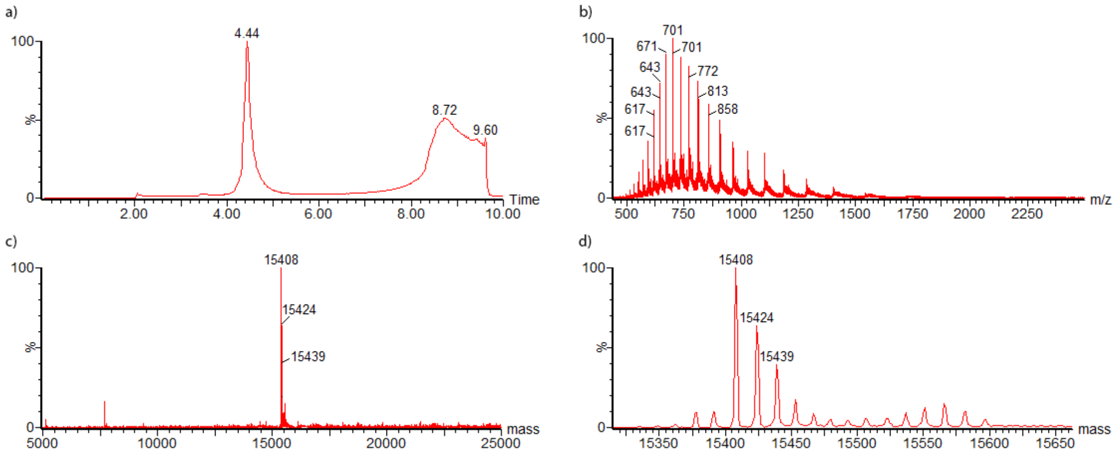

LCMS analysis of [^{15}N]Histone H3-Ser9er: a) total ion chromatogram b) ion series c) deconvoluted spectrum d) magnification of the major peak**;** calculated mass: 15410 g/mol; observed masses: 15408 g/mol, 15424 g/mol (+ 1 × oxidation), 15439 g/mol (+ 2 × oxidation).

3.4.18 Annexin V

3.4.18.1 Annexin V-Dha316

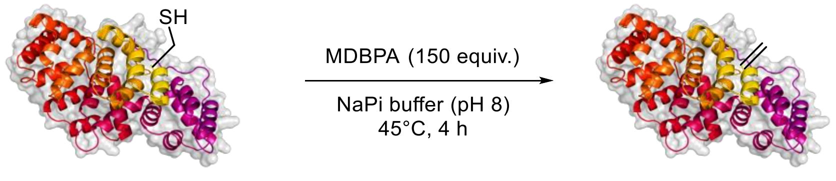

Annexin V **Annexin V C316Dha**

To 450 µL of a solution of Annexin V (2.00 mg/mL, 25.1 nmol, 1.00 equiv.) in NaP_i buffer (50 mM, pH 7.8) was added DTT (0.19 mg, 1.26 µmol, 50 equiv.). The mixture was incubated at room temperature for 20 min. and desalted by passing through a GE MiniTrap G-25 column pre-equilibrated with NaP_i buffer (100 mM, pH 8.0) according to the manufacturer's instructions. A solution of MDBPA (0.69 mg, 2.51 µmol, 100 equiv.) in 45 µL of DMSO was added and the mixture was shaken at 45ºC for 3.5 h. Another 50 equiv. of MDBPA in DMSO were added and the mixture was shaken for another 25 min. The mixture was passed through a GE MiniTrap G-25 column pre-equilibrated with NaP_i buffer (100 mM, pH 7.0) and yielding 1.5 mL of a solution of Annexin V-Dha316 with a concentration of 0.60 mg/mL (900 µg, 25.1 nmol, quantitative recovery). The product contained 15% double alkylated product.

The sample was aliquoted, frozen in liquid nitrogen and stored at −80°C.

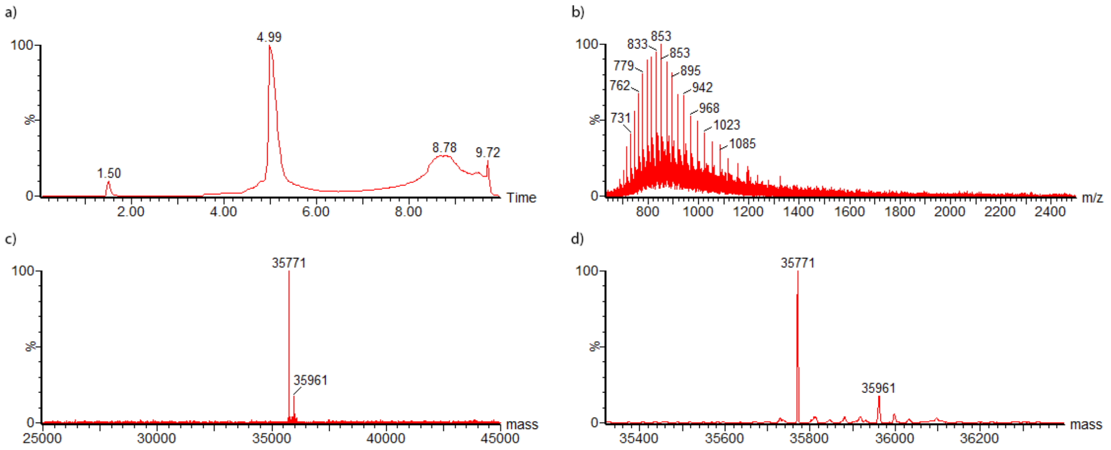

LCMS analysis of Annexin V-Dha316: a) total ion chromatogram b) ion series c) deconvoluted spectrum d) magnification of the major peak**;** calculated mass: 34772 g/mol; observed mass: 34771 g/mol.

3.4.18.2 Annexin V-Bal316

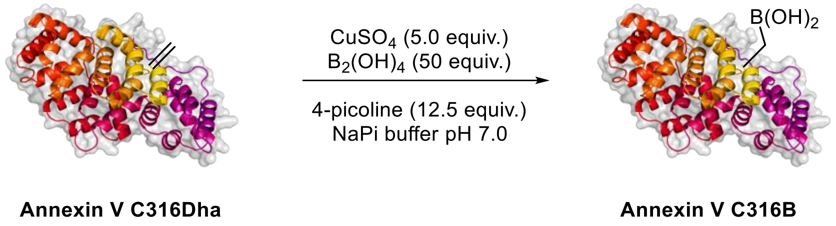

Stock solutions of $CuSO_4 \cdot 5\ H_2O$ (5.2 mg in 1.00 mL of H_2O), 4-picoline (5.10 µL in 1.00 mL of H_2O) and $B_2(OH)_4$ (7.5 mg in 1.00 mL of H_2O) were freshly prepared before the reaction was conducted.

To a solution of Annexin V-Dha316 (40 µL, 0.20 mg/mL, 0.56 nmol, 1.00 equiv.) in NaP_i buffer (100 mM, 3 M Gdn·HCl, pH 7.0) pre-warmed to 45°C were added previously prepared and pre-warmed stock solutions of 4-picoline (2.00 µL, 104.8 nmol, 187.5 equiv.), $CuSO_4$ (2.00 µL, 41.9 nmol, 75.0 equiv.) and $B_2(OH)_4$ (5.00 µL, 419 nmol, 750 equiv.) subsequently. The mixture was vortexed and shaken for 10 min at 45°C. LCMS indicated full conversion (59% Bal−H_2O, 41% Bal−$2H_2O$).

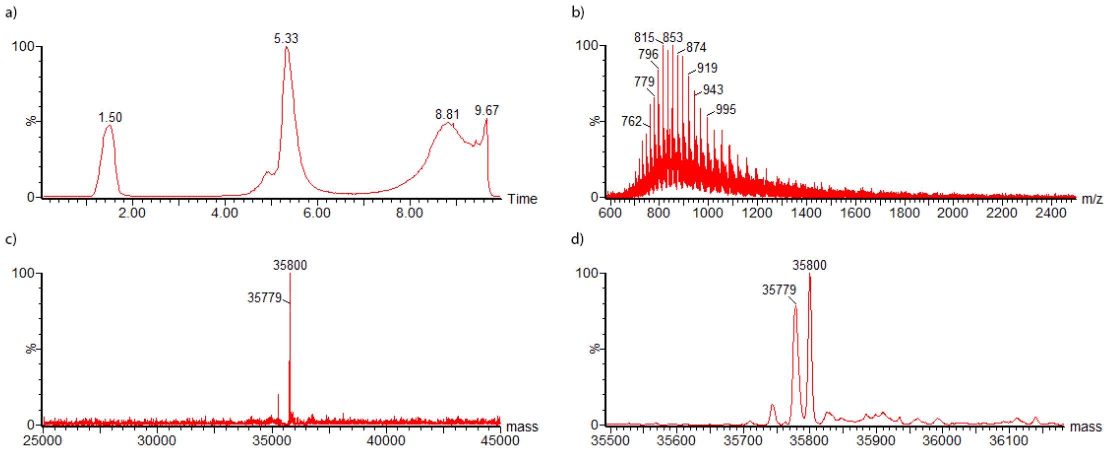

LCMS analysis of Annexin V-Bal316: a) total ion chromatogram b) ion series c) deconvoluted spectrum d) magnification of the major peak**;** calculated masses: 35799 g/mol (Bal−H_2O), 35781 g/mol (Bal−$2H_2O$); observed masses: 35800 g/mol (Bal−H_2O), 35779 g/mol (Bal−$2H_2O$).

3.4.19 Npβ

3.4.19.1 Npβ-Dha61

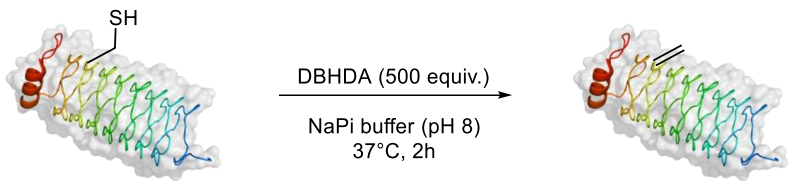

To a solution of Npβ (2.74 mg, 130 nmol, 1.00 equiv.) in 500 µL of NaP_i buffer (50 mM, pH 7.8) was added DTT (1.00 mg, 6.51 µmol, 50 equiv.). The mixture was vortexed and incubated at room temperature for 30 min before it was purified using a GE PD MiniTrap G-25 which was previously equilibrated with NaP_i buffer (100 mM,

pH 8.0) according to the manufacturer's instructions. A solution of DBHDA (19.7 mg, 65.1 µmol, 500 equiv.) in 160 µL of DMF was added to the purified protein and the reaction mixture was shaken at 37°C and 500 rpm for 2 h. The protein was purified using a GE PD MiniTrap G-25 which was previously equilibrated with NaP_i buffer (100 mM, pH 7.0) to yield 2000 µL of protein solution with a concentration of 0.92 mg/mL as determined by A280 spectrophotometry (1.84 mg, 87.6 nmol, 67% recovery).

The sample was aliquoted, frozen in liquid nitrogen and stored at −80°C.

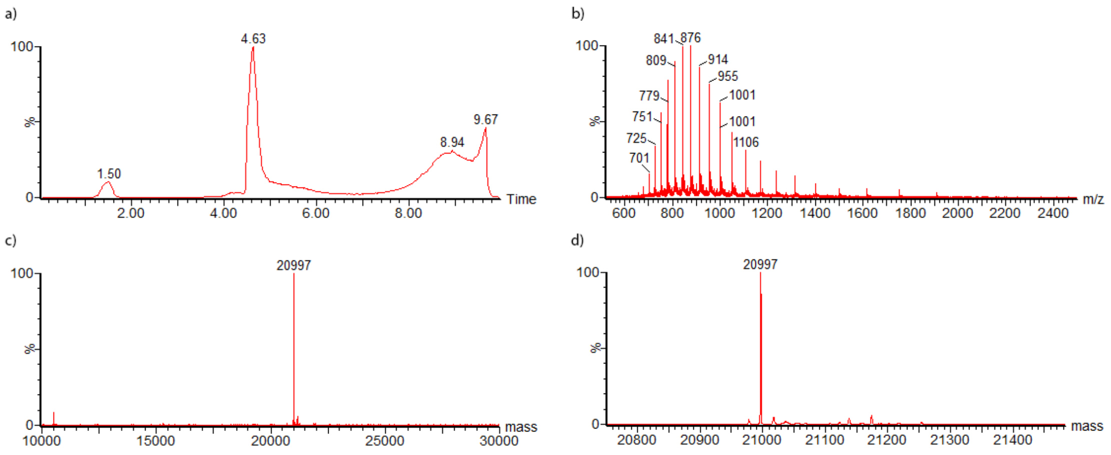

LCMS analysis of Npβ-Dha61: a) total ion chromatogram b) ion series c) deconvoluted spectrum d) magnification of the major peak; calculated mass: 20997 g/mol; observed mass: 20997 g/mol.

3.4.19.2 Npβ-Bal61

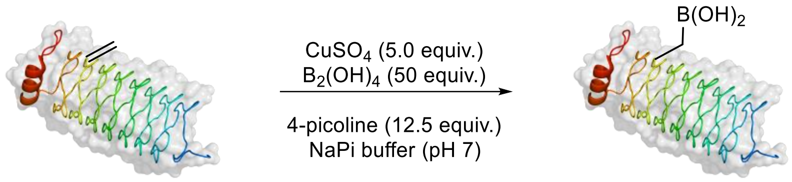

Stock solutions of $CuSO_4 \cdot 5\ H_2O$ (3.5 mg in 1.00 mL of H_2O), 4-picoline (1.71 µL in 1.00 mL of H_2O) and $B_2(OH)_4$ (6.3 mg in 1.00 mL of H_2O) were freshly prepared before the reaction was conducted.

To a solution of Npβ-Dha61 (50 µL, 1.12 mg/mL, 2.67 nmol, 1.00 equiv.) in NaP_i buffer (100 mM, 3 M Gdn·HCl, pH 7.0) were subsequently added stock solutions of 4-picoline (1.90 µL, 13.4 nmol, 12.5 equiv), CuSO4 (0.95 µL, 13.4 nmol, 5.00 equiv.) and $B_2(OH)_4$ (1.90 µL, 134 nmol, 50 equiv.). The mixture was briefly vortexed and incubated at room temperature for 10 min (>99% conversion, 12% boronic acid, 57% mono-ester, 31% di-ester).

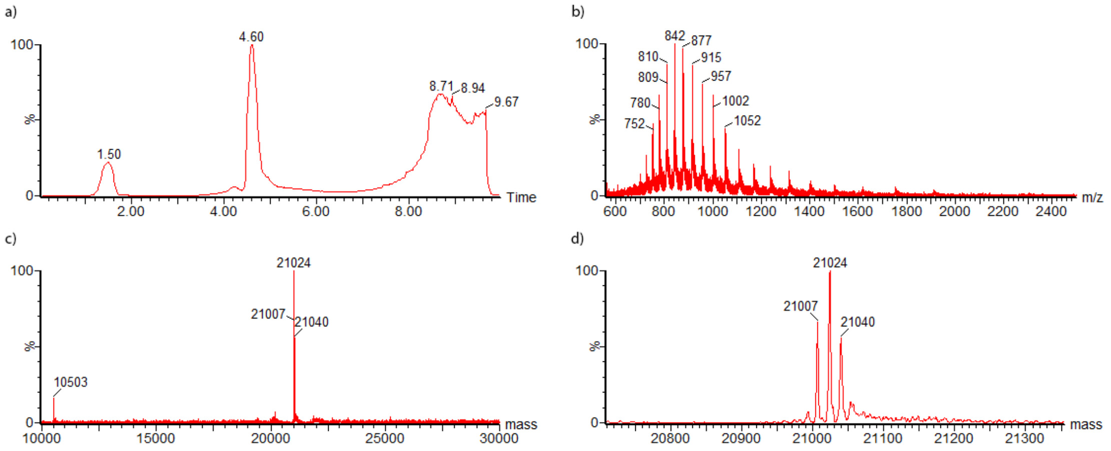

LCMS analysis of Npβ-Bal61: a) total ion chromatogram b) ion series c) deconvoluted spectrum d) magnification of the major peak**;** calculated masses: 21043 g/mol (Bal), 21025 g/mol (Bal−H_2O), 21007 g/mol (Bal−$2H_2O$); observed masses: 21040 g/mol (Bal), 21024 g/mol (Bal−H_2O), 21007 g/mol (Bal−$2H_2O$).

3.4.20 PanC

3.4.20.1 PanC-Dha44 / PanC-Dha47

PanC-Dha44 and PanC-Dha47 were formed following a previously published procedure.[21]

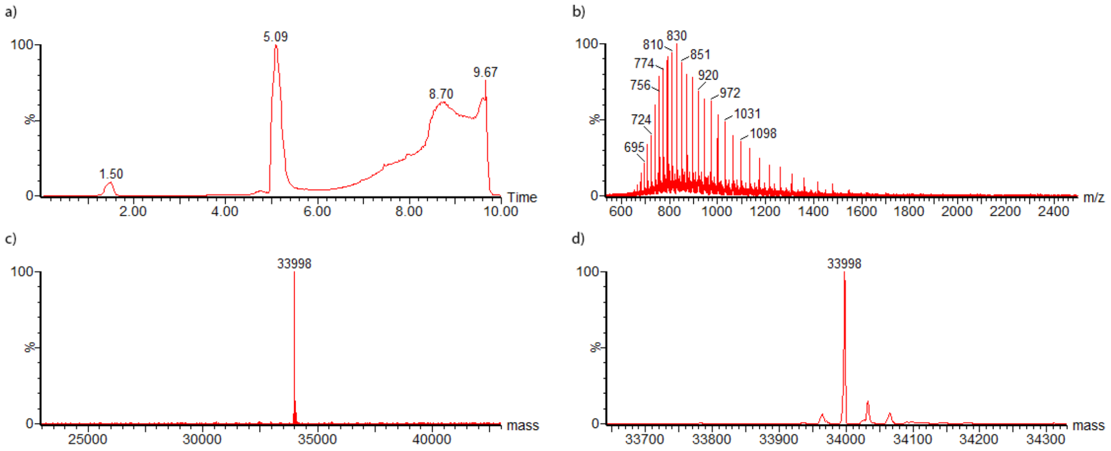

LCMS analysis of Panc-Dha44: a) total ion chromatogram b) ion series c) deconvoluted spectrum d) magnification of the major peak; calculated mass: 33998 g/mol; observed mass: 33998 g/mol.

3.4.20.2 PanC-Bal44

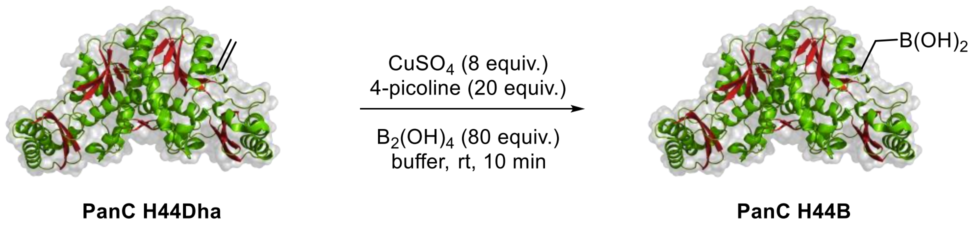

Stock solutions of $CuSO_4 \cdot 5\ H_2O$ (3.5 mg in 1.00 mL of H_2O), 4-picoline (1.71 µL in 1.00 mL of H_2O) and $B_2(OH)_4$ (6.3 mg in 1.00 mL of H_2O) were freshly prepared before the reaction was conducted.

To a solution of PanC-Dha44 (gratefully received from Dr. P. Isenegger) (50 µL, 0.99 mg/mL, 1.46 nmol, 1.00 equiv.) in NaP_i buffer (100 mM, 3 M Gdn·HCl, pH 7.0) were subsequently added stock solutions of 4-picoline (1.70 µL, 29.2 nmol, 20 equiv), CuSO4 (0.85 µL, 11.7 nmol, 8.00 equiv.) and $B_2(OH)_4$ (1.70 µL, 117 nmol, 80 equiv.). The mixture was briefly vortexed and incubated at room temperature for 10 min (71% conversion, 56% mono-ester, 15% oxidized product).

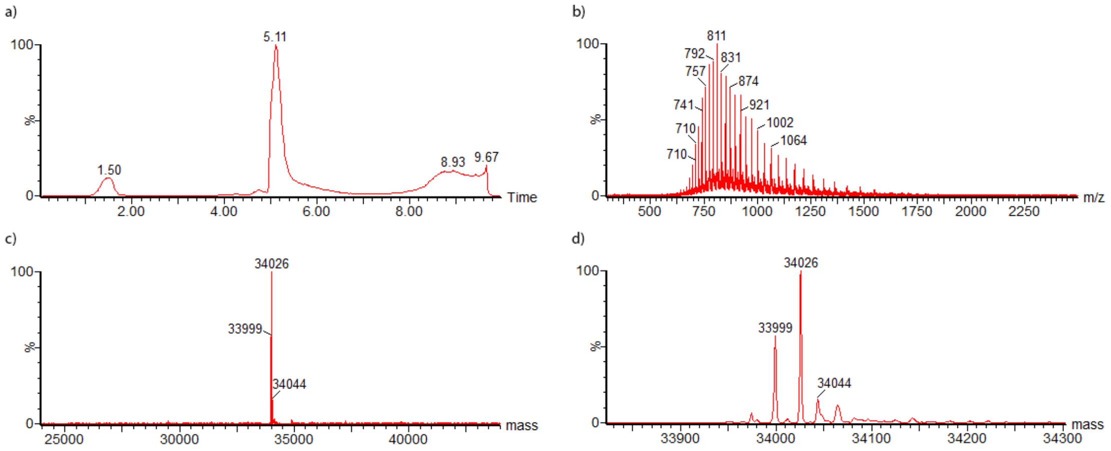

LCMS analysis of PanC-Bal44: a) total ion chromatogram b) ion series c) deconvoluted spectrum d) magnification of the major peak**;** calculated masses: 34044 g/mol (Bal), 34026 g/mol (Bal−H_2O); observed masses: 34044 g/mol (Bal), 34026 g/mol (Bal−H_2O).

3.4.20.3 PanC-Bal47

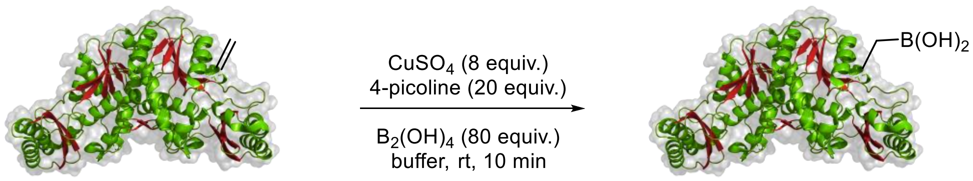

PanC H47Dha **PanC H47B**

Stock solutions of $CuSO_4 \cdot 5\,H_2O$ (3.5 mg in 1.00 mL of H_2O), 4-picoline (1.71 µL in 1.00 mL of H_2O) and $B_2(OH)_4$ (6.3 mg in 1.00 mL of H_2O) were freshly prepared before the reaction was conducted.

To a solution of PanC-Dha47 (gratefully received from Dr. P. Isenegger) (50 µL, 1.16 mg/mL, 1.71 nmol, 1.00 equiv.) in NaP$_i$ buffer (100 mM, 3 M Gdn·HCl, pH 7.0) were subsequently added stock solutions of 4-picoline (2.00 µL, 34.1 nmol, 20 equiv), CuSO4 (1.00 µL, 13.7 nmol, 8.00 equiv.) and $B_2(OH)_4$ (2.00 µL, 137 nmol, 80 equiv.). The mixture was briefly vortexed and incubated at room temperature for 10 min (60% conversion, 13% boronic acid, 42% mono-ester, 5% oxidized product).

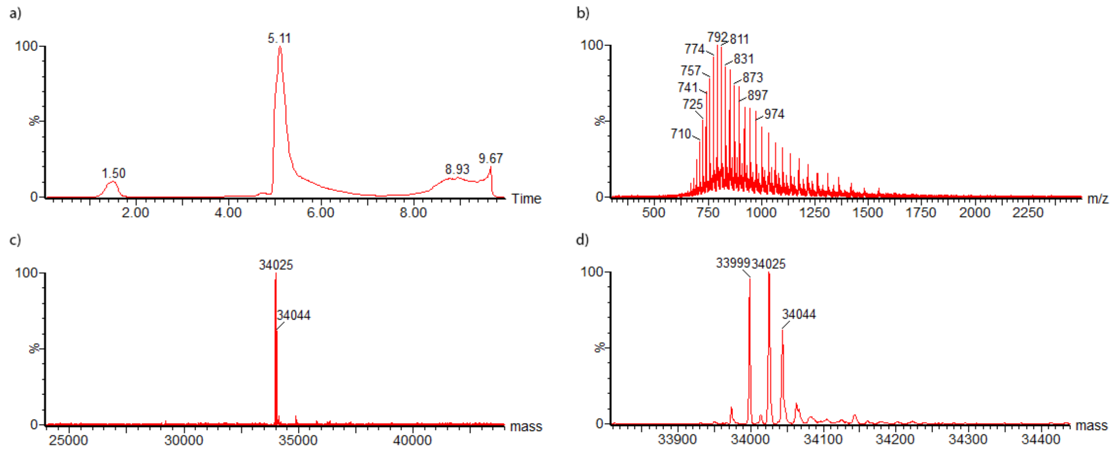

LCMS analysis of PanC-Bal47: a) total ion chromatogram b) ion series c) deconvoluted spectrum d) magnification of the major peak**;** calculated masses: 34044 g/mol (Bal), 34025 g/mol (Bal−H_2O); observed masses: 34044 g/mol (Bal), 34025 g/mol (Bal−H_2O).

3.4.21 pre-SUMO1

3.4.21.1 pre-SUMO1-Dha51

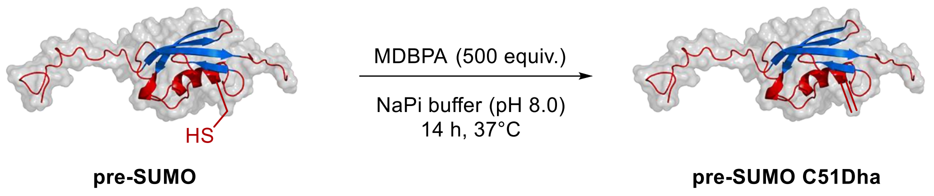

A solution of pre-SUMO1 (4000 µL, 2.30 mg/mL, 737 nmol, 1.00 equiv.) was incubated with DTT (5.7 mg, 36.9 µmol, 50.0 equiv.) for 30 min at room temperature. The protein was purified using GE PD MidiTraps G-25 which were previously equilibrated with NaP_i buffer (100 mM, pH 8.0) according to the manufacturer's instructions. To the purified protein solution was added a solution of MDBPA (57.4 µL, 369 µmol, 500 equiv.) in DMSO (142.6 µL), the mixture was vortexed and shaken at 37°C and 600 rpm for 14 h. The solution was concentrated to 2500 µL using a VivaSpin 20 concentrator (MWCO 3,000 Da). 2000 µL of this solution were purified using GE PD MidiTraps G-25 which were previously equilibrated with NaP_i buffer (100 mM, 3 M Gdn·HCl, pH 7.0) yielding 3000 µL of a solution of pre-SUMO1-Dha51 (2.01 mg, 199 nmol, 27% recovery).

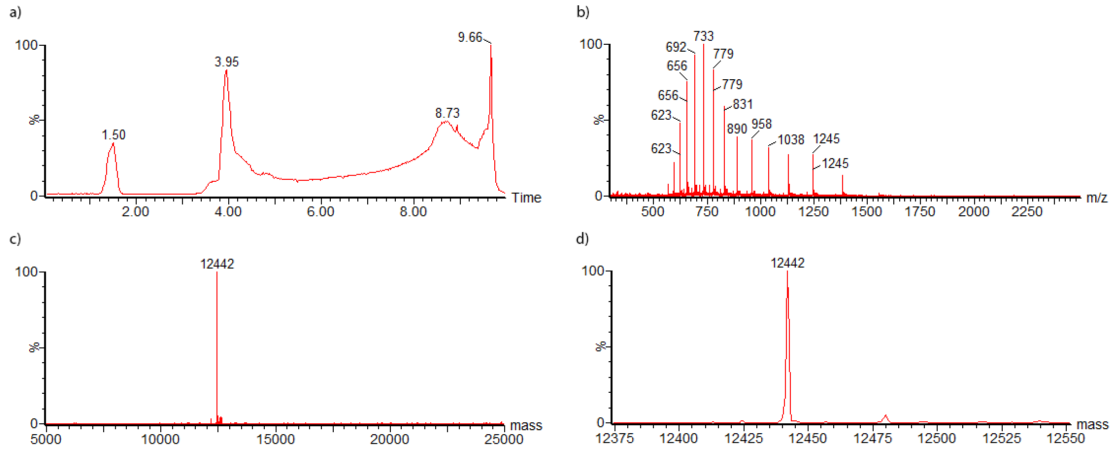

LCMS analysis of pre-SUMO1-Dha51: a) total ion chromatogram b) ion series c) deconvoluted spectrum d) magnification of the major peak; calculated mass: 12441 g/mol; observed mass: 12442 g/mol.

3.4.21.2 pre-SUMO1-Bal51

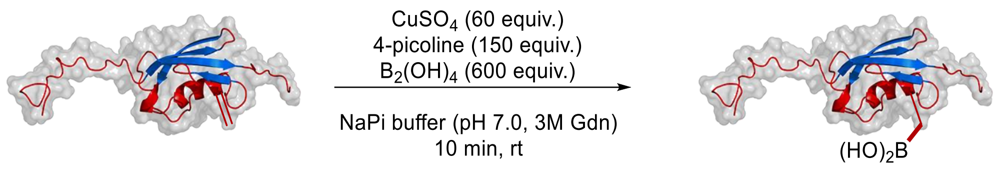

pre-SUMO C51Dha **pre-SUMO C51Bal**

Stock solutions of $CuSO_4 \cdot 5\ H_2O$ (40.3 mg in 1.00 mL of H_2O), 4-picoline (39.3 µL in 1.00 mL of H_2O) and $B_2(OH)_4$ (8.1 mg in 1.00 mL of H_2O) were freshly prepared before the reaction was conducted.

To a solution of pre-SUMO1-Dha51 (500 µL, 0.67 mg/mL, 26.9 nmol, 1.00 equiv.) in NaP_i buffer (100 mM, 3 M Gdn·HCl, pH 7.0) were subsequently added stock solutions of 4-picoline (10.0 µL, 4.04 µmol, 150 equiv), $CuSO_4$ (10.0 µL, 1.62 µmol, 60 equiv.) and $B_2(OH)_4$ (180 µL, 16.2 µmol, 600 equiv.). The mixture was briefly vortexed and incubated at room temperature for 10 min (full conversion). The mixture was dialyzed against SUMO maturation buffer (50 mM TRIS base, 20 mM NaCl, 2 mM DTT).

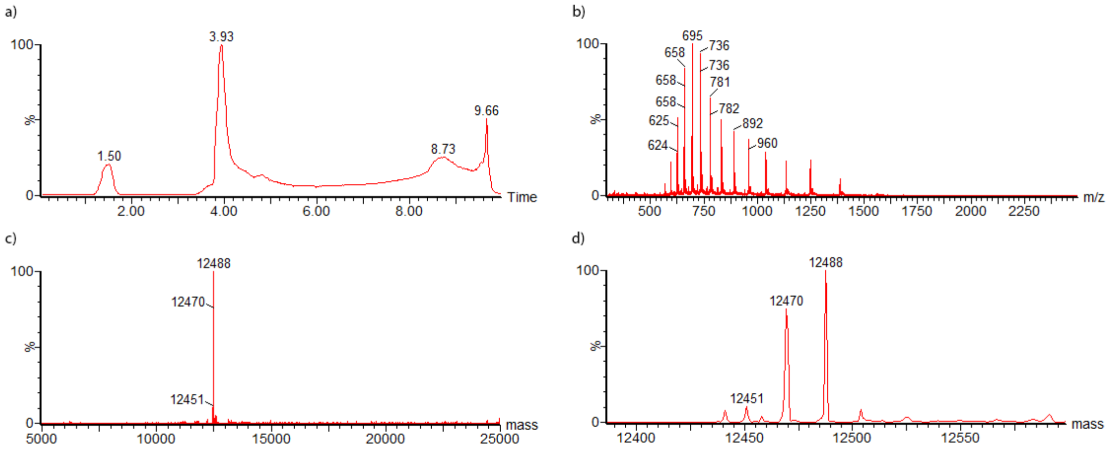

LCMS analysis of pre-SUMO1-Bal51: a) total ion chromatogram b) ion series c) deconvoluted spectrum d) magnification of the major peak**;** calculated masses: 12487 g/mol (Bal), 12469 g/mol (Bal−H_2O), 12451 g/mol (Bal−$2H_2O$); observed masses: 12488 g/mol (Bal), 12470 g/mol (Bal−H_2O), 12451 g/mol (Bal−$2H_2O$).

3.4.22 mCherry

3.4.22.1 mCherry-Sulfonium131

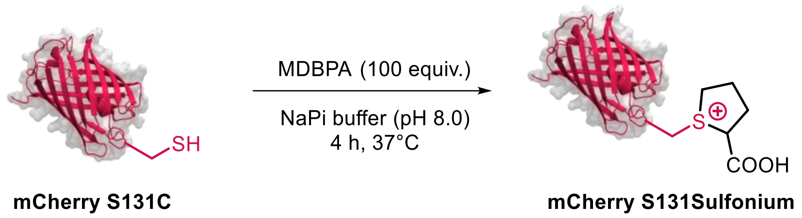

A solution of mCherry-Cys131 (1000 µL, 4.56 mg/mL, 366 nmol) in storage buffer was transferred to a glove box and degassed for 14 h at 4°C. The protein was purified using a GE PD MidiTrap G-25 which was previously equilibrated with NaP$_i$ buffer (100 mM, pH 8.0) according to the manufacturer's instructions. A solution of MDBPA (6.20 µL, 36.6 µmol, 100 equiv.) in 50 µL of MeCN was added and the mixture was shaken at 37°C and 500 rpm for 4 h. The mixture was transferred out of the glove box, concentrated to 1000 µL using VivaSpin 500 concentrators (MWCO 5,000 Da) and purified using a GE PD MidiTrap G-25 which was previously equilibrated with NaP$_i$ buffer (100 mM, pH 7.0) according to the manufacturer's instructions to yield 1500 µL of a solution of mCherry-Sulfonium131 (2.41 mg/mL, 3.62 mg, 289 nmol, 79% recovery).

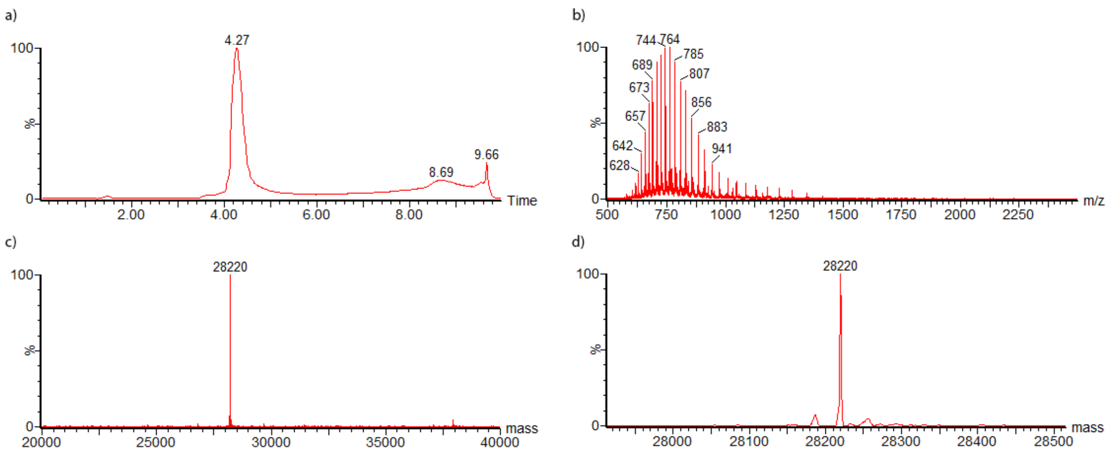

LCMS analysis of mCherry-Sulfonium131: a) total ion chromatogram b) ion series c) deconvoluted spectrum d) magnification of the major peak**;** calculated mass: 28215 g/mol; observed mass: 28220 g/mol.

3.4.22.2 mCherry-Bal131

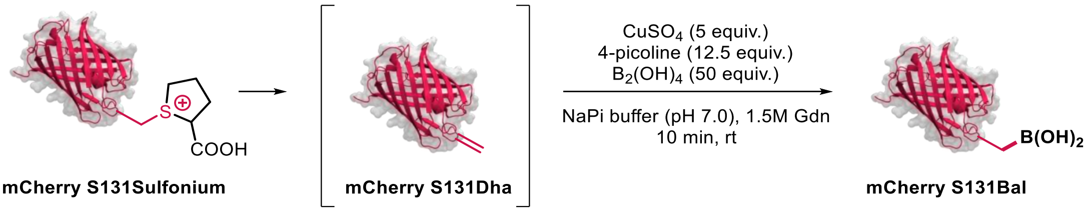

Stock solutions of $CuSO_4 \cdot 5\ H_2O$ (5.3 mg in 1.00 mL of H_2O), 4-picoline (5.19 µL in 1.00 mL of H_2O) and $B_2(OH)_4$ (4.8 mg in 1.00 mL of H_2O) were freshly prepared before the reaction was conducted.

A solution of mCherry-Sulfonium131 (100 µL, 2.41 mg/mL, 8.54 nmol, 1.00 equiv.) in NaP_i buffer (100 mM, pH 7.0) was diluted with 100 µL of NaP_i buffer (100 mM, 3 M Gdn·HCl, pH 7.0). Stock solutions of 4-picoline (2.0 µL, 107 nmol, 12.5 equiv), $CuSO_4$ (2.0 µL, 42.7 nmol, 5.0 equiv.) and $B_2(OH)_4$ (8.0 µL, 427 nmol, 50 equiv.) were added subsequently. The mixture was briefly vortexed and incubated at room temperature for 10 min (35% conversion).

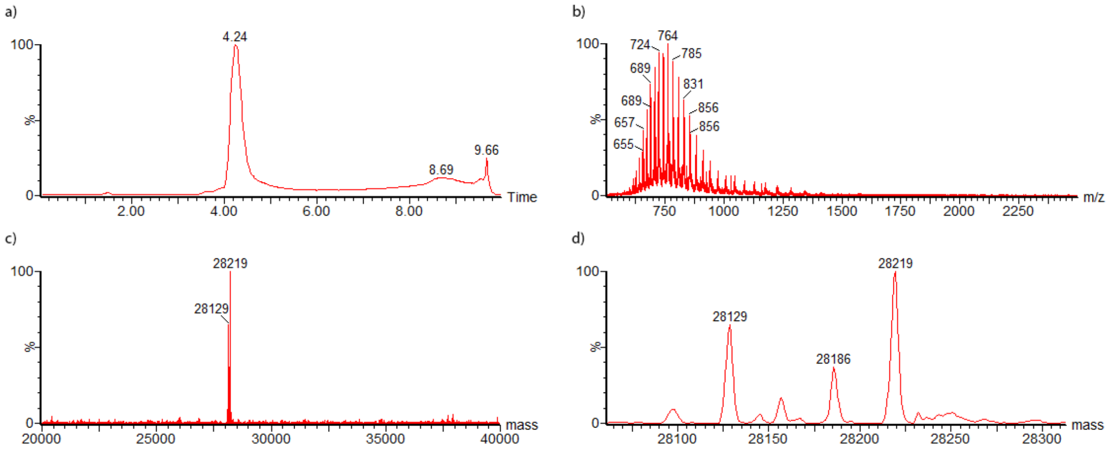

LCMS analysis of mCherry-Bal131: a) total ion chromatogram b) ion series c) deconvoluted spectrum d) magnification of the major peak**;** calculated mass: 28128 g/mol (Bal); observed masses: 28129 g/mol (Bal).

3.4.23 PstS

3.4.23.1 PstS-Dha197

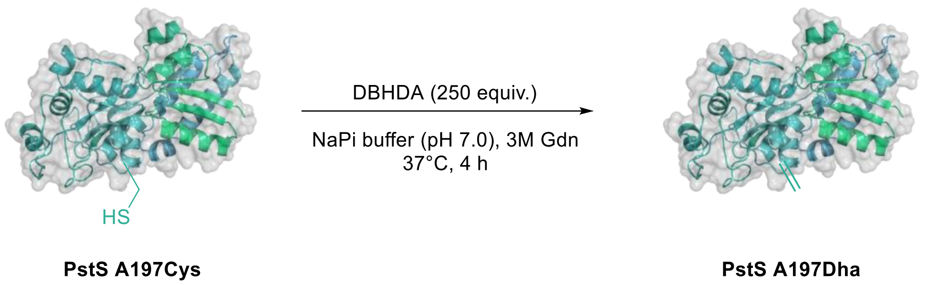

A solution of PstS-Cys197 (500 µL, 2.07 mg/mL, 1.04 mg, 29.9 nmol) was incubated with DTT (0.23 mg, 1.50 µmol, 50 equiv.) for 30 min at room temperature. The protein solution was purified using a GE PD MidiTrap G-25 which was previously equilibrated with NaP_i buffer (100 mM, pH 8.0, 3 M Gdn·HCl) according to the manufacturer's instructions. To the purified protein was added a solution of DBHDA (2.3 mg, 7.48 µmol, 250 equiv.) in 50 µL of DMF. The mixture was shaken at 37°C for 4 h (500 rpm) before being purified using a GE PD MidiTrap G-25 which was previously equilibrated with NaP_i buffer (100 mM, pH 7.0, 3 M Gdn·HCl) to yield 1500 µL of a solution of PstS-Dha197 with a concentration of 0.44 mg/mL (32% recovery).

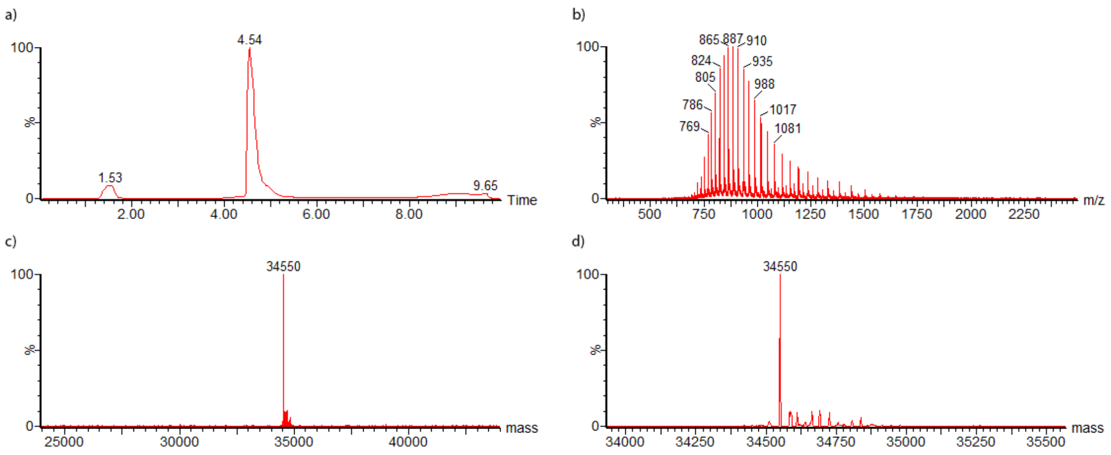

LCMS analysis of PstS-Dha197: a) total ion chromatogram b) ion series c) deconvoluted spectrum d) magnification of the major peak**;** calculated mass: 34551 g/mol; observed mass: 34550 g/mol.

3.4.23.2 PstS-Bal197

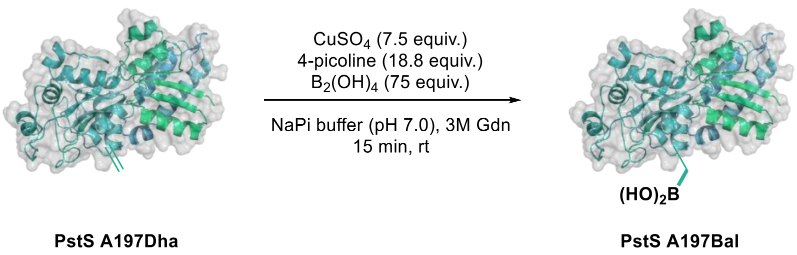

Stock solutions of $CuSO_4 \cdot 5\,H_2O$ (3.0 mg in 1.00 mL of H_2O), 4-picoline (2.90 µL in 1.00 mL of H_2O) and $B_2(OH)_4$ (5.4 mg in 1.00 mL of H_2O) were freshly prepared before the reaction was conducted.

To a solution of PstS-Bal197 (500 µL, 0.44 mg/mL, 220 µg, 6.37 nmol) in NaP_i buffer (100 mM, pH 7.0, 3 M Gdn·HCl) were added the previously prepared stock solutions of 4-picoline (4.00 µL, 119 nmol, 18.8 equiv.), $CuSO_4$ (4.00 µL, 47.8 nmol, 7.50 equiv) and $B_2(OH)_4$ (8.00 µL, 478 nmol, 75.0 equiv.). The sample was vortexed and incubated at room temperature for 15 min. 79% conversion was indicated by LCMS.

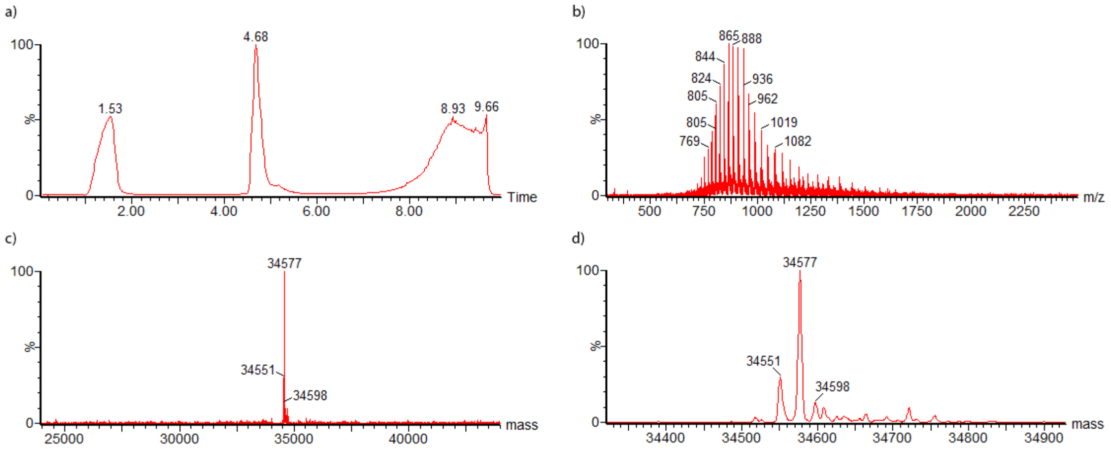

LCMS analysis of PstS-Bal197: a) total ion chromatogram b) ion series c) deconvoluted spectrum d) magnification of the major peak**;** calculated masses: 34597 g/mol (Bal), 34579 g/mol (Bal−H_2O); observed masses: 34598 g/mol (Bal), 34577 g/mol (Bal−H_2O).

3.4.24 AcrA

3.4.24.1 AcrA-Dha123

AcrA-Dha123 was synthesized following a previously published procedure.[22]

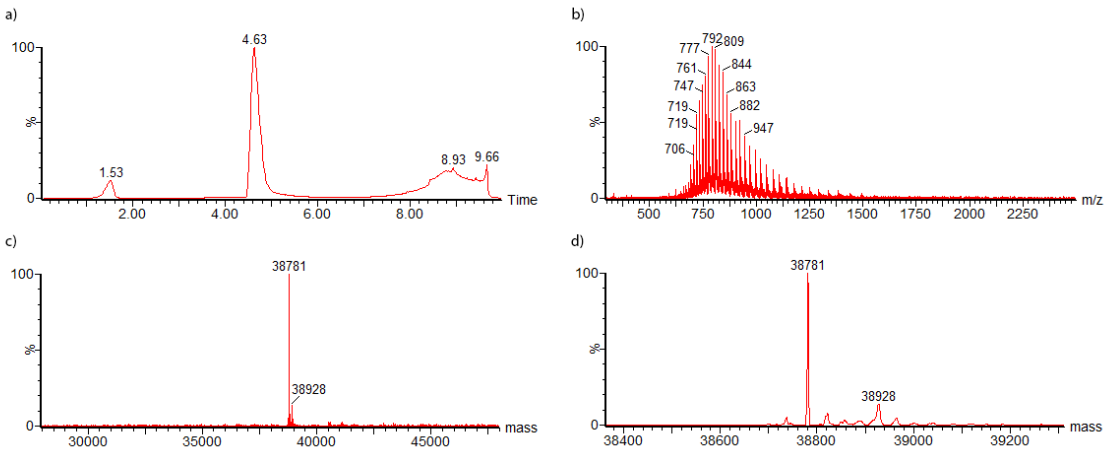

LCMS analysis of AcrA-Dha123: a) total ion chromatogram b) ion series c) deconvoluted spectrum d) magnification of the major peak**;** calculated mass: 38783 g/mol; observed mass: 38781 g/mol.

3.4.24.2 AcrA-Bal123

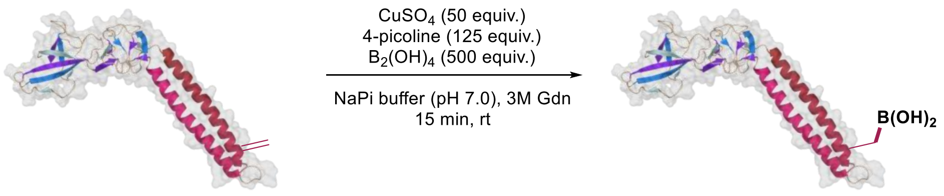

Stock solutions of $CuSO_4 \cdot 5\ H_2O$ (2.2 mg in 1.00 mL of H_2O), 4-picoline (2.09 µL in 1.00 mL of H_2O) and $B_2(OH)_4$ (3.9 mg in 1.00 mL of H_2O) were freshly prepared before the reaction was conducted.

To 50 µL of a solution of AcrA-Dha123 (0.13 mg/mL, 0.17 nmol) in PBS were added 50 µL of NaP$_i$ buffer (100 mM, pH 7.0, 6 M Gdn·HCl). The previously prepared stock solutions of 4-picoline (1.00 µL, 21.7 nmol, 125 equiv.), $CuSO_4$ (1.00 µL, 8.70 nmol, 50 equiv.) and $B_2(OH)_4$ (2.00 µL, 87.0 nmol, 500 equiv.) were added, the sample was briefly vortexed and incubated for 15 min. 77% conversion was determined by LCMS.

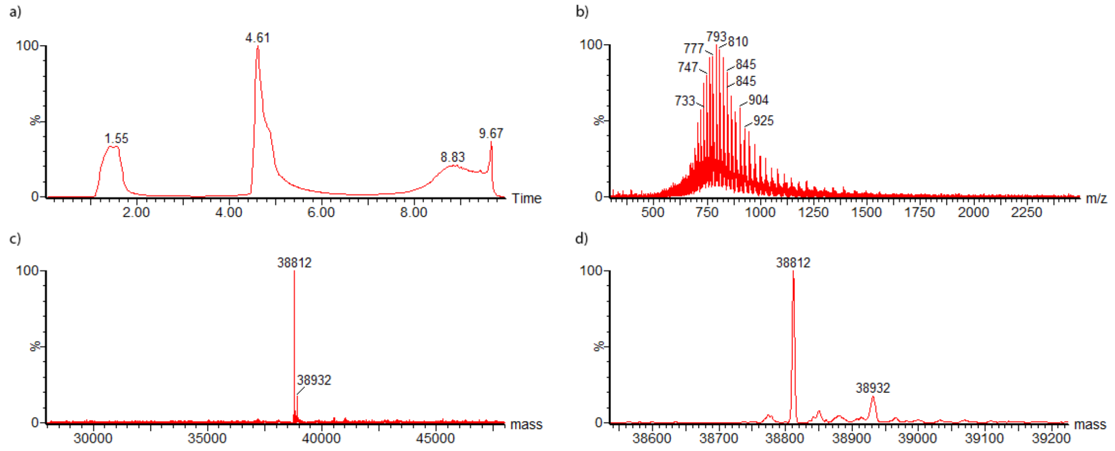

LCMS analysis of AcrA-Bal123: a) total ion chromatogram b) ion series c) deconvoluted spectrum d) magnification of the major peak**;** calculated mass: 38811 g/mol (Bal−H_2O); observed mass: 38812 g/mol (Bal−H_2O).

3.4.25 Histone H4

3.4.25.1 Histone H4-Dha16

Histone H4-Dha16 was synthesized following a previously published procedure.[22]

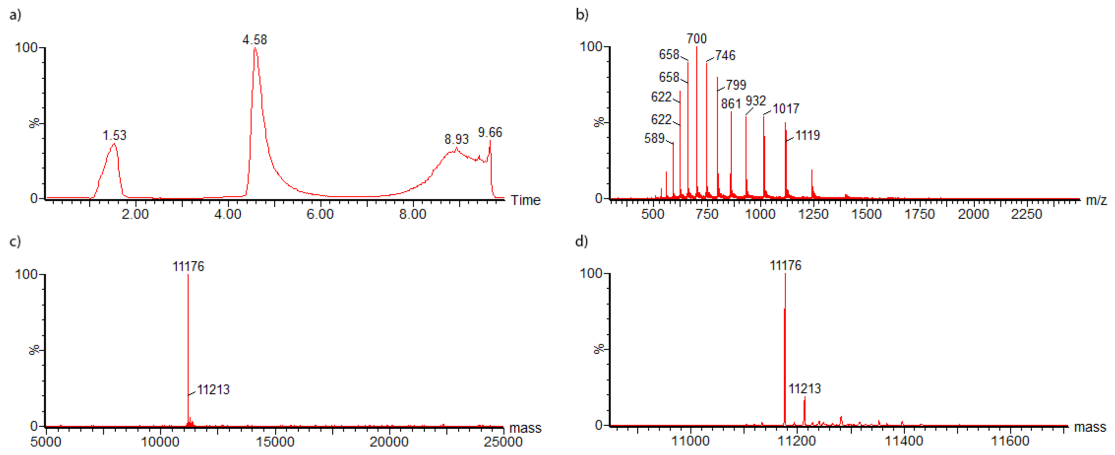

LCMS analysis of Histone H4-Dha16: a) total ion chromatogram b) ion series c) deconvoluted spectrum d) magnification of the major peak**;** calculated mass: 11177 g/mol; observed mass: 11176 g/mol.

3.4.25.2 Histone H4-Bal16

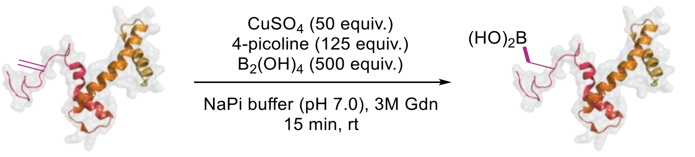

Histone H4 K16C **Histone H4 K16Bal**

Stock solutions of $CuSO_4 \cdot 5\ H_2O$ (2.1 mg in 1.00 mL of H_2O), 4-picoline (2.02 µL in 1.00 mL of H_2O) and $B_2(OH)_4$ (3.7 mg in 1.00 mL of H_2O) were freshly prepared before the reaction was conducted.

To 50 µL of a solution of Histone H4-Bal16 (0.37 mg/mL, 1.66 nmol) in NaP_i buffer (100 mM, pH 7.0, 6 M Gdn·HCl) were added the previously prepared stock solutions of 4-picoline (1.00 µL, 20.7 nmol, 12.5 equiv.), $CuSO_4$ (1.00 µL, 8.28 nmol, 5.00 equiv.) and $B_2(OH)_4$ (2.00 µL, 82.8 nmol, 50 equiv.) were added, the sample was briefly vortexed and incubated for 15 min. full conversion was determined by LCMS.

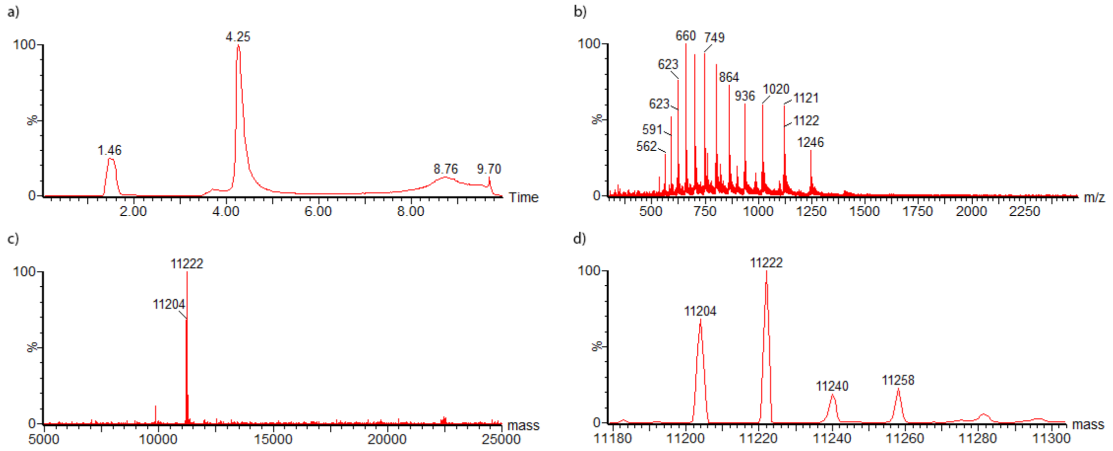

LCMS analysis of Histone H4-Bal16: a) total ion chromatogram b) ion series c) deconvoluted spectrum d) magnification of the major peak**;** calculated masses: 11223 g/mol (Bal), 11205 g/mol (Bal−H_2O); observed masses: 11222 g/mol (Bal), 11204 g/mol (Bal−H_2O).

4 References

[1] C. Louis-Jeune, M. A. Andrade-Navarro, C. Perez-Iratxeta, *Proteins Struct. Funct. Bioinforma.* **2012**, *80,* 374–381.

[2] L. A. Compton, W. C. Johnson, *Anal. Biochem.* **1986**, *155*, 155–167.

[3] P. Manavalan, W. C. Johnson, *Anal. Biochem.* **1987**, *167*, 76–85.

[4] N. Sreerama, R. W. Woody, *Anal. Biochem.* **2000**, *287*, 252–260.

[5] A. Micsonai, F. Wien, L. Kernya, Y.-H. Lee, Y. Goto, M. Réfrégiers, J. Kardos, *Proc. Natl. Acad. Sci.* **2015**, *112*, E3095–E3103.

[6] D. P. Klose, B. A. Wallace, R. W. Janes, *Bioinformatics* **2010**, *26*, 2624–2625.

[7] M. J. F. and G. W. T. and H. B. S. and G. E. S. and M. A. R. and J. R. C. and G. S. and V. B. and B. M. and G. A. P. and H. N. and M. C. and X. L. and H. P. H. and A. F. I. and Fox, *Gaussian 09, Gaussian, Inc., Wallingford CT* **2009**.

[8] G. Karunanithy, A. Cnossen, H. Müller, M. D. Peeks, N. H. Rees, T. D. W. Claridge, H. L. Anderson, A. J. Baldwin, *Chem. Commun.* **2016**, *52*, 7450–7453.

[9] M. W. Lodewyk, C. Soldi, P. B. Jones, M. M. Olmstead, J. Rita, J. T. Shaw, D. J. Tantillo, *J. Am. Chem. Soc.* **2012**, *134*, 18550–18553.

[10] B. N. Naidu, M. E. Sorenson, T. P. Connolly, Y. Ueda, *J. Org. Chem.* **2003**, *68,* 10098–10102.

[11] H. J. Reich, W. S. Goldenberg, B. Ö. Gudmundsson, A. W. Sanders, K. J. Kulicke, K. Simon, I. A. Guzei, *J. Am. Chem. Soc.* **2001**, *123*, 8067–8079.

[12] D. R. Morgan, H. C. Dorn, *J. Label. Compd. Radiopharm.* **1991**, *29*, 777–779.

[13] B. Dedeoğlu, I. Uğur, I. Değirmenci, V. Aviyente, B. Barçin, G. Çayli, H. Y. Acar, *Polymer* **2013**, *54*, 5122–5132.

[14] B. P. Rempel, S. G. Withers, *Org. Biomol. Chem.* **2014**, *12*, 2592–2595.

[15] C. C. Geilen, N. Loch, W. Reutter, K. Seppelt, F. Oberdorfer, *Tetrahedron Lett.* **1992**, *33*, 2435–2438.

[16] J. N. Barlow, J. S. Blanchard, *Carbohydr. Res.* **2000**, *328*, 473–480.

[17] X. G. Li, S. Dall'Angelo, L. F. Schweiger, M. Zanda, D. O'Hagan, *Chem. Commun.* **2012**, *48*, 5247–5249.

[18] M. Sharma, Y. X. Li, M. Ledvina, M. Bobek, *Nucleosides Nucleotides Nucleic Acids* **1995**, *14*, 1831–1852.

[19] J. B. Liu, X. H. Xu, F. L. Qing, *Org. Lett.* **2015**, *17*, 5048–5051.

[20] P. Lundström, P. Vallurupalli, D. F. Hansen, L. E. Kay, *Nat. Protoc.* **2009**, *4*, 1641–1648.

[21] J. Dadová, K.-J. Wu, P. G. Isenegger, J. C. Errey, G. J. L. Bernardes, J. M. Chalker, L. Raich, C. Rovira, B. G. Davis, *ACS Cent. Sci.* **2017**, *3*, 1168–1173.

[22] T. H. Wright, B. J. Bower, J. M. Chalker, G. J. L. Bernardes, R. Wiewiora, W.-L. Ng, R. Raj, S. Faulkner, M. R. J. Vallee, A. Phanumartwiwath, et al., *Science* **2016**, *354*, 597.
