# Supplementary material for: Post-translational insertion of boron in proteins to probe and modulate function
Source: Nat Chem Biol. 2021 Nov 1;17(12):1245–61. doi: 10.1038/s41589-021-00883-7 (PMC8604732; doi:10.1038/s41589-021-00883-7)
Supplement: Supplementary file 2 — Reporting Summary [file 41589_2021_883_MOESM48_ESM.pdf]

nature research

Corresponding author(s): Davis

Last updated by author(s): Jul 26, 2021

Reporting Summary

Nature Research wishes to improve the reproducibility of the work that we publish. This form provides structure for consistency and transparency in reporting. For further information on Nature Research policies, see our Editorial Policies and the Editorial Policy Checklist.

Statistics

For all statistical analyses, confirm that the following items are present in the figure legend, table legend, main text, or Methods section.

n/a	Confirmed	
☐	☒	The exact sample size (n) for each experimental group/condition, given as a discrete number and unit of measurement
☐	☒	A statement on whether measurements were taken from distinct samples or whether the same sample was measured repeatedly
☒	☐	The statistical test(s) used AND whether they are one- or two-sided<br>*Only common tests should be described solely by name; describe more complex techniques in the Methods section.*
☒	☐	A description of all covariates tested
☒	☐	A description of any assumptions or corrections, such as tests of normality and adjustment for multiple comparisons
☐	☒	A full description of the statistical parameters including central tendency (e.g. means) or other basic estimates (e.g. regression coefficient) AND variation (e.g. standard deviation) or associated estimates of uncertainty (e.g. confidence intervals)
☒	☐	For null hypothesis testing, the test statistic (e.g. F, t, r) with confidence intervals, effect sizes, degrees of freedom and P value noted<br>*Give P values as exact values whenever suitable.*
☒	☐	For Bayesian analysis, information on the choice of priors and Markov chain Monte Carlo settings
☒	☐	For hierarchical and complex designs, identification of the appropriate level for tests and full reporting of outcomes
☒	☐	Estimates of effect sizes (e.g. Cohen's d, Pearson's r), indicating how they were calculated

Our web collection on statistics for biologists contains articles on many of the points above.

Software and code

Policy information about availability of computer code

Data collection	Data was collected using the software provided by the respective instrument vendor and is specified in the Methods section.
Data analysis	Data was analysed with MassLynx 4.1 (Waters), GraphPad Prism 8 (GraphPad Software Inc.), PEAKS Studio 8.5 and PEAKS Studio X (Bioinformatics Solutions Inc.), MestReNova 14 (Mestrelab Research S.L.), TopSpin 4 (Bruker BioSpin GmbH), FlowJo 10 (FlowJo LLC), Microsoft Excel 2016, FreeSASA (no version number), K2D3 (no version number), CDSSTR (no version number), BeStSel (no version number), 2Struct (no version number), PR.Stability Analysis 1 (NanoTemper Technologies), nmPIPE v. 10.9 rev. 2020.119.13.27 64-bit, SPARKY v 3.115, SMILE v. 2.1 rev. 2019.337.11.19 64-bit.

For manuscripts utilizing custom algorithms or software that are central to the research but not yet described in published literature, software must be made available to editors and reviewers. We strongly encourage code deposition in a community repository (e.g. GitHub). See the Nature Research guidelines for submitting code & software for further information.

Data

Policy information about availability of data

All manuscripts must include a data availability statement. This statement should provide the following information, where applicable:

- Accession codes, unique identifiers, or web links for publicly available datasets
- A list of figures that have associated raw data
- A description of any restrictions on data availability

Raw protein LC-MS, raw protein MSMS, and raw NMR data (protein and small molecule), raw nucleosome NMR data and primary numerical data for all graphical plots is deposited in the open-access depositories ORA-data (partial) (https://ora.ox.ac.uk/objects/uuid:ca409cd6-36d0-4788-a3c8-083e32bf0e18) and Zenodo (full) (DOI: 10.5281/zenodo.4900115).

The following publically available protein structures were used: acrA (PDB ID 2FMA), Annexin V (PDB ID 1HVD), nucleosome, Histone H3 and Histone H4 (PDB ID 1KX5), mCherry (PDB ID 4ZIN), Npβ (PDB ID 2J8K), panC (PDB ID 1N2E), preSUMO1 (PDB ID 1A5R) and PstS (PDB ID 1A40).

Field-specific reporting

Please select the one below that is the best fit for your research. If you are not sure, read the appropriate sections before making your selection.

☒ Life sciences ☐ Behavioural & social sciences ☐ Ecological, evolutionary & environmental sciences

For a reference copy of the document with all sections, see nature.com/documents/nr-reporting-summary-flat.pdf

Life sciences study design

All studies must disclose on these points even when the disclosure is negative.

Sample size	Diol binding studies, protein melting, PstS assay and boronate distribution (except for AcrA, n=1) were conducted in triplicate (n=3). Chemical synthesis and protein modification were carried out as single experiments (n=1) and reported values (conversions and yields) should be regarded as semi-quantitative. Single reactions were deemed appropriate for synthetic reactions as these procedures were performed independently multiple times and proved reliable and consistent.
Data exclusions	No data was excluded
Replication	Diol binding studies, protein melting, PstS assay and boronate distribution (except for AcrA, n=1) were conducted in triplicate (n=3). Chemical synthesis and protein modification were carried out as single experiments (n=1) and reported values (conversions and yields) should be regarded as semi-quantitative. Single reactions were deemed appropriate for synthetic reactions as these procedures were performed independently multiple times and proved reliable and consistent. No statistical methods were used to determine sample size. Single experiments were deemed appropriate for synthetic procedures as they were viewed in the context of hundreds of comparable experiments conducted throughout this study. All attempts at replication were successful for synthetic reactions, protein melting, PstS assay and boronate distribution determination. Protein borylation was repeated constantly during this study and all attempts at replication were successful.
Randomization	Not applicable; No experimental groups were involved.
Blinding	Not applicable; No group allocation was conducted.

Reporting for specific materials, systems and methods

We require information from authors about some types of materials, experimental systems and methods used in many studies. Here, indicate whether each material, system or method listed is relevant to your study. If you are not sure if a list item applies to your research, read the appropriate section before selecting a response.

Materials & experimental systems

n/a	Involved in the study	
☐	☒	Antibodies
☐	☒	Eukaryotic cell lines
☒	☐	Palaeontology and archaeology
☒	☐	Animals and other organisms
☒	☐	Human research participants
☒	☐	Clinical data
☒	☐	Dual use research of concern

Methods

n/a	Involved in the study	
☒	☐	ChIP-seq
☐	☒	Flow cytometry
☒	☐	MRI-based neuroimaging

Antibodies

Antibodies used	Anti-SUMO1 Polyclonal, Rabbit (Abcam, ab139470, Lot: GR3240564-4); Anti-Rabbit IgG–Alkaline Phosphatase (goat) (Sigma Aldrich, A3687); Anti-Mouse IgG–Alkaline Phosphatase (goat) (Sigma Aldrich, A3562, Lot: SLCB8722); Anti-Histone H3 pSer10 Monoclonal, Mouse (GeneTex, GTX630185, clone GT921, Lot: 41505). Dilutions used were 1:1000.
Validation	Anti-SUMO1 antibody was supplied as part of a kit (SUMOylation assay kit, Abcam, ab139470) and val<br>The Anti-SUMO1 antibody was received as part of a SUMOylation assay kit (Abcam, ab139470). The antibody was validated by the manufacturer via Western Blot (https://www.abcam.com/ps/products/139/ab139470/documents/SUMOylation-Assay-Kit-v3-ab139470%20%20(website).pdf). Positive controls were included.idated by the manufacturer. Additionally, control experiments were conducted. Anti-Histone H3 pSer10 antibody was validated by the manufacturer via Western Blot, IHC-P and ICC.<br>Anti-SUMO1 Polyclonal, Rabbit (Abcam, ab139470, Lot: GR3240564-4): Available from: https://www.abcam.com/sumoylation-assay-kit-ab139470.html. Antibody validated by the manufacturer via Western Blot (https://www.abcam.com/ps/products/139/ab139470/documents/SUMOylation-Assay-Kit-v3-ab139470%20%20(website).pdf)<br>Anti-Histone H3 pSer10 Monoclonal, Mouse (GeneTex, GTX630185, Lot: 41505): Available from: https://www.genetex.com/Product/

Detail/Histone-H3S10ph-phospho-Ser10-antibody-GT921/GTX630185. Antibody validated by the manufacturer through orthogonal validation using Western Blot, IHC-P and ICC. See https://www.genetex.com/PDF/Download?catno=GTX630185 for details.
The Anti-SUMO1 antibody was received as part of a SUMOylation assay kit (Abcam, ab139470). The antibody was validated by the manufacturer via Western Blot (https://www.abcam.com/ps/products/139/ab139470/documents/SUMOylation-Assay-Kit-v3-ab139470%20%20(website).pdf). Positive controls were included.

Eukaryotic cell lines

Policy information about cell lines

Cell line source(s)
CHO cells (wt) were received from Prof. Paul Crocker (University of Dundee); Jurkat cells (clone E6.1) were received from Prof. Quentin Sattentau (Sir William Dunn School of Pathology, University of Oxford) and are available from the European Collection of Authenticated Cell Cultures (Catalogue No.: 88042803)

Authentication
Cell lines were not re-authenticated prior to use.

Mycoplasma contamination
All cell lines tested negative for Mycoplasma contamination.

Commonly misidentified lines (See ICLAC register)
No commonly misidentified cell lines were used.

Flow Cytometry

Plots

Confirm that:

- [x] The axis labels state the marker and fluorochrome used (e.g. CD4-FITC).
- [x] The axis scales are clearly visible. Include numbers along axes only for bottom left plot of group (a 'group' is an analysis of identical markers).
- [x] All plots are contour plots with outliers or pseudocolor plots.
- [x] A numerical value for number of cells or percentage (with statistics) is provided.

Methodology

Sample preparation
To 100 µL of CHO wt cells (approx. 106 cells) in FACS buffer (Dulbecco's phosphate-buffered saline, pH 8.0, 2% FBS) were added 300 µL of mCherry mutant (0.28 mg/mL, 10 µM) or 300 µL of FACS buffer (control). The cells where shaken on ice for 20 min at 300 rpm. The samples were centrifuged at 400 g for 3 min at 4°C. The liquid was removed and the cell pellet was resuspended in 1000 µL of FACS buffer, centrifuged at 400 g for 3 min at 4°C and the buffer was removed. The washing step was repeated once before the cell pellet was suspended in 400 µL of FACS buffer.
Flow cytometry was performed on a BD LSRFortessa™ X-20 cell analyser using BD FACSDiva 8.0 software. A minimum of 10,000 cells per sample was analysed using the 561 nm laser and a 610/20 nm bandpass filter. The 640 nm laser in combination with a 780/60 nm bandpass filter was used as a reference channel. The data was analysed using FlowJo Version 10 software. Histograms depict mCherry fluorescence versus cell count, dot plots depict mCherry fluorescence versus the reference channel.

Instrument
BD LSRFortessa™ X-20, BD FACSCalibur™

Software
FACSDiva (data collection), FlowJo (data analysis)

Cell population abundance
All cell lines were homogenous containing only one population. A minimum of 10,000 cells were analysed each run.

Gating strategy
Cells were gated using forward and side scatter.

- [x] Tick this box to confirm that a figure exemplifying the gating strategy is provided in the Supplementary Information.
